# The genomic diversity of Taiwanese Austronesian groups: Implications for the “Into- and Out-of-Taiwan” models

**DOI:** 10.1093/pnasnexus/pgad122

**Published:** 2023-05-16

**Authors:** Dang Liu, Albert Min-Shan Ko, Mark Stoneking

**Affiliations:** Department of Evolutionary Genetics, Max Planck Institute for Evolutionary Anthropology, Leipzig 04103, Germany; Human Evolutionary Genetics Unit, Institut Pasteur, UMR 2000, CNRS, Paris 75015, France; Department and Graduate Institute of Biomedical Sciences, Chang Gung University, Taoyuan 333, Taiwan; Cardiovascular Department, Chang Gung Memorial Hospital, Taoyuan 333, Taiwan; Healthy Aging Research Center, Chang Gung University, Taoyuan 333, Taiwan; Department of Evolutionary Genetics, Max Planck Institute for Evolutionary Anthropology, Leipzig 04103, Germany; Laboratoire de Biométrie et Biologie Evolutive, Université Lyon 1, CNRS, UMR 5558, Villeurbanne 69622, France

**Keywords:** Taiwan, Austronesian, Into-Taiwan, Out-of-Taiwan, genome-wide data

## Abstract

The origin and dispersal of the Austronesian language family, one of the largest and most widespread in the world, have long attracted the attention of linguists, archaeologists, and geneticists. Even though there is a growing consensus that Taiwan is the source of the spread of Austronesian languages, little is known about the migration patterns of the early Austronesians who settled in and left Taiwan, i.e. the “Into-Taiwan” and “out-of-Taiwan” events. In particular, the genetic diversity and structure within Taiwan and how this relates to the into-/out-of-Taiwan events are largely unexplored, primarily because most genomic studies have largely utilized data from just two of the 16 recognized Highland Austronesian groups in Taiwan. In this study, we generated the largest genome-wide data set of Taiwanese Austronesians to date, including six Highland groups and one Lowland group from across the island and two Taiwanese Han groups. We identified fine-scale genomic structure in Taiwan, inferred the ancestry profile of the ancestors of Austronesians, and found that the southern Taiwanese Austronesians show excess genetic affinities with the Austronesians outside of Taiwan. Our findings thus shed new light on the Into- and Out-of-Taiwan dispersals.

**Significance Statement**

The expansion of Austronesian-speaking people probably originated from Taiwan and spread from there throughout Southeast Asia and the Pacific. Yet, despite the considerable linguistic diversity of Taiwanese Austronesian groups, genomic studies of the Austronesian expansion typically include only one or two of them. To study how Taiwanese diversity influences inferences about the Into- and Out-of-Taiwan migrations, we generated the largest genomic data set of Taiwanese Austronesians to date. We find considerable genetic structure between northern and southern Highland groups, increased northern East Asian-related ancestry in the ancestors of the Out-of-Taiwan migration, and closer relationships between southern Highland groups and Austronesians outside Taiwan, illustrating the important impact of Taiwanese genomic diversity on inferences about the Austronesian expansion.

## Introduction

Austronesian is one of the largest language families in the world, with more than 1,200 languages spoken by almost 400 million people, and being spread from Madagascar in the west to Hawaii and Easter Island in the east (1). Linguistic analyses strongly support a Taiwanese origin for Austronesian languages (2, 3), and archaeological and genetic evidence further supports an expansion of people from Taiwan associated with the spread of Austronesian languages (4–7). For this reason, the migration events “Into-Taiwan,” which detail the arrival of the ancestors of Taiwanese Austronesians on the island, and “Out-of-Taiwan,” which detail the departure of the same ancestors for other Austronesian groups, are of great importance.

Regarding “Into-Taiwan,” based on the distribution of millet and rice in ancient sites, archeologists estimate that the ancestors of Taiwanese Austronesian arrived in Taiwan from the southeastern coast of mainland China ∼4.8 thousand years ago (kya) at the latest (8). Linguistically, these people are considered “proto-Austronesians,” and their language shares features with the Tai-Kadai and Sino-Tibetan languages spoken in southeastern coast of China (9). Recent ancient DNA studies also find strong genetic links between the Taiwanese Austronesians and the ancient individuals from southern China (associated with the Neolithic agricultural culture) (10, 11).

As for “Out-of-Taiwan,” archaeological evidence suggests that the agricultural complex associated with Austronesian ancestors began expanding from Taiwan into the Philippines ∼ 4.2 kya (12) and then rapidly throughout Indonesia, west to Madagascar, and east across the Pacific (5, 13). Among the 10 divisions of the Austronesian language family recognized by linguists, 9 (Formosan branches) are found only in Taiwan, while the remaining Austronesian languages outside Taiwan are grouped under the Malayo-Polynesian branch (14). Phylogenetic analyses of Austronesian languages have also supported an origin in Taiwan and estimated that the Formosan and Malayo-Polynesian branches diverged ∼5 kya (3).

Genetic studies have found evidence for an Out-of-Taiwan migration, although with estimated dates ranging from 4 to 8 kya (6, 15–17). Additionally, the expansion of Austronesian people is associated with the development of the Lapita culture (3.5–2.5 kya) in Near Oceania and the spread into Remote Oceania (13, 18). Surprisingly, genomes from ancient individuals of Remote Oceania related to the Lapita have revealed a strong genetic link to the Taiwanese Austronesian groups (19–21), suggesting these individuals were among the earliest Out-of-Taiwan groups.

While there has been much investigation of the Into- and Out-of-Taiwan migrations, the diversity and structure of Austronesian groups within Taiwan remain relatively unexplored. Most genome-wide studies have used data from only two groups, the Amis and Atayal (15, 16, 21), when in fact there are over 20 recognized indigenous (Austronesian) groups in Taiwan according to the Council of Indigenous People (CIP) in Taiwan (https://www.cip.gov.tw; last accessed 2022 December 12). These are broadly classified as the “Highland” or “Lowland” (called “Gaoshan” or “Pingpu” in Mandarin, respectively) groups based on where they live, although some of the Highland groups do not reside in the mountainous area. Presently, there are 16 officially defined indigenous Highland groups (the Atayal, Saysiyat, Truku, Sediq, Sakizaya, Thao, Tsou, Kavalan, Bunun, Hla’alua, Kanakanavu, Amis, Rukai, Puyuma, Paiwan, and the Tao). The Tao (or Yami) actually reside on Orchid Island, which is located off the southeastern coast of Taiwan. And while all Highland groups speak Formosan languages, the Yami language is part of the Malayo-Polynesian branch (1, 14). There are another 12 or so identified Lowland groups (the Ketagalan, Kavalan, Taokas, Kaxabu, Pazeh, Papora, Babuza, Lloa, Arikun, Siraya, Taivoan, and the Makatao). Extensive contact with Han people (primarily the Minnan and Hakka) who migrated from mainland China in the past 500 years has led to the extinction or endangerment of Lowland Austronesian languages (described on the CIP website).

The Highland groups of Taiwan have been the primary focus of genetic studies, while the Lowland groups have received much less attention. There is evidence of genetic diversity among Taiwanese Austronesians, as shown by studies of the Highland groups using uniparental genetic makers (6, 22–25), although few of these studies included the Lowland groups (6, 24, 25). Furthermore, genome-wide studies, which can provide much more detailed insights into population histories, are very limited. To our knowledge, genome-wide data have only been generated for the Atayal, Amis, Paiwan, and Tao (Yami) (17, 26–28). Moreover, a systematic genomic assessment of the diversity and relationships among Taiwanese groups and exploration as to how these data relate to the Into- and Out-of-Taiwan migrations are still lacking.

To remedy this gap in knowledge, we generated new genome-wide data for seven Taiwanese Austronesian groups from across the island (three with no genome-wide data reported before, including one Lowland group) and two Taiwanese Han groups to investigate fine scale structure within Taiwan. We used the CIP's definition of Taiwan Highland/Taiwan Orchid Island (THI/TOI) to categorize the Highlands’ various groups. Combing our new data with published comparative modern and ancient genomes, we leverage the diversity of Taiwanese Austronesians to gain new insights for the Into- and Out-of-Taiwan events.

## Results

### Taiwanese groups in the light of genetic variation in Asia and Oceania

**Fig. 1.**
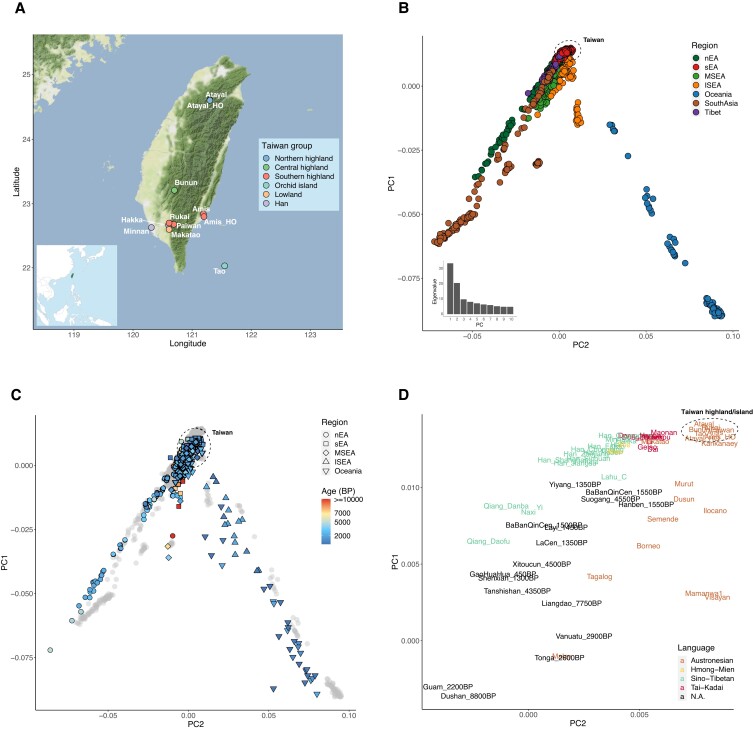
Map of the sampled Taiwanese groups and PCA of the modern/ancient individuals from South Asia, East Asia, and Oceania. A) Sampling locations of the Taiwanese populations. Map tiles by Stamen Design (CC BY 3.0), data by OpenStreetMap (ODbL). The Atayal_HO and Amis_HO are from published Human Origins data. The small map panel on the bottom-left indicates Taiwan (green) in the geographical context of East Asia. B) PCA of the modern individuals from South Asia, East Asia, and Oceania in the merged data set, colored by regions. The dashed circle indicates where the Taiwanese individuals fall. The eigenvalues from PC1 to PC10 are shown on the bottom-left. C) PCA with the ancient individuals projected onto the PCA in B) with the modern individuals in gray. Symbol shapes indicate region and colors indicate age (D). Zoom-in of C) with a focus on the modern groups from sEA and ISEA and the ancient groups from sEA and Lapita-related Oceania, colored by languages. The position of a group is the median of the positions of individuals from the group.

We generated genome-wide single-nucleotide polymorphism (SNP) array data on the Affymetrix Human Origins array for 55 individuals from seven Austronesian (five Highland, one Lowland, and one Orchid Island) and two Han groups from Taiwan (Fig. 1A). We merged the new data with comparative modern and ancient genomes from Asia and Oceania (Fig. S1).

We first investigated overall patterns in the data set using principal component analysis (PCA) and ADMIXTURE (29)/DyStruct (30) analyses. In the PCA, the East Asians (southern East Asians, sEA; northern East Asians, nEA; Mainland Southeast Asians, MSEA; and Island Southeast Asians, ISEA) were separated from the South Asians and Oceanians. For the sEA/nEA, southern/northern East Asia was defined by the south/north of the Qinling–Huaihe (10). Along PC1, the sEA were located at the extreme of the East Asian pole, the ISEA were positioned on the cline toward the Oceanians, and the remaining East Asian groups fell on the cline toward South Asians. The genetic profiles of all Taiwanese individuals fell within the genetic variation of the sEA (Fig. 1B and C), with the Taiwan Austronesian groups appearing on the extreme of the sEA pole together with the Filipino Austronesian group Kankanaey (Fig. 1D). Ancient individuals closest to the Taiwanese groups were the sEA and early (∼2–3 kya) Oceanian individuals (Fig. 1D).

**Fig. 2.**
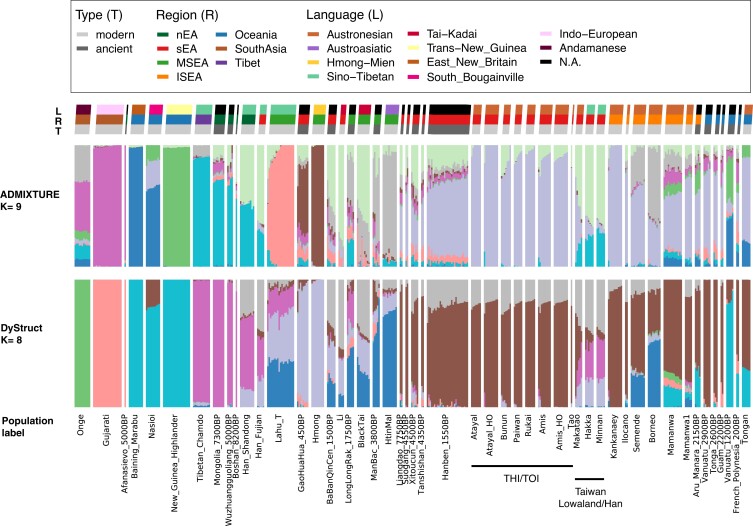
*K* = 9 of ADMIXTURE and *K* = 8 of DyStruct for selected representative groups. Both number of *K* are the best-fitting *K* for ADMIXTURE/DyStruct (Fig. S2). From the top to the bottom, there is a legend for the following three rows of keys indicating the language (L), the region (R), and the type of ancient/modern genomes (T) for the populations; below the keys, there are two rows showing the results of ADMIXTURE and DyStruct, respectively; the last row indicates the specific population label. Each vertical thin bar represents an individual, and different populations are separated with gaps. Representative groups are selected based on their enrichment of a source component, e.g. Andamanese, Indo-European, Papuan (Trans-New Guinea, East New Britain, South Bougainville), Sinto-Tibetan/nEA, Hmong-Mien, Austroasiatic, or their relevance to the into-/out-of-Taiwan events, e.g. Tai-Kadai, sEA, Taiwan, ISEA, and Oceania. Results of the full data set are presented in Fig. S3.

For the best-fitting *K* value of the ADMIXTURE analysis (Fig. S2A), the Atayal and Kankanaey had the highest frequencies of a purple component which is also enriched in the THI/TOI groups and ancient sEA (e.g. the ∼4.5 kya Suogang and ∼4.3 kya Tanshishan) and Oceanian (e.g. the ∼2.9 kya Vanuatu and ∼2.6 kya Tonga) individuals (Figs. 2 and S3A). In contrast, the Taiwanese Han groups were similar to the Han from Fujian in southern China, in having two major components: light green and turquoise. In comparison with the Han from Shandong of northern China, the Taiwanese and Fujian Han groups showed small amounts of the purple Austronesian-related component, suggesting potential interactions between the sEA Han and Austronesian groups. Similarly, the Taiwanese Lowland Austronesian group, Makatao, had a similar profile to the Taiwanese Han groups but with more of the purple Austronesian-related component.

The best-fitting *K* value for the DyStruct analysis, an ADMIXTURE-like clustering algorithm that incorporates time transects and is hence more suitable for ancient DNA, indicated similar results for the THI/TOI groups being close to the Kankanaey and ancient sEA individuals. They shared a relatively homogenous pattern of two components (Figs. 2 and S3B). The brown one occurred at the highest frequency in the ∼4.5 kya sEA Suogang (likely proto-Austronesians) and ∼2.9 kya Vanuatu (likely early Austronesians), which is associated with all modern Austronesian groups in ISEA and Oceania, suggesting a link between all of them. The gray one occurred at the highest frequency in the Tai-Kadai-speaking Li and was widespread among present­-day East Asians.

To investigate this gray component, we noticed that it occurred at a higher frequency in the Taiwan Lowland/Han groups, as did the light/dark purple components, which is similar to the pattern of the light green and turquoise components in ADMIXTURE. Furthermore, all of these were enriched in the Han groups (Fig. 2). We therefore compared the proportions of these components in the Taiwanese groups (ADMIXTURE light green vs. turquoise; DyStruct gray + light purple vs. dark purple) and found a correlation between them (*r*^2^ = 0.913 and 0.926, respectively; both *P* = 0), suggesting they might have been introduced via a major Han admixture event (Fig. S4). Moreover, the ratio of these components was correlated with the geographical distribution of the Han groups (northern groups showed more turquoise/dark purple compared with southern groups). In this regard, the ratio in the Taiwanese Han was close to that of the southern Chinese Han groups, while the cline of the Taiwanese Austronesian groups pointed toward the Taiwanese Han (Fig. S4).

### Genetic structure within Taiwan

**Fig. 3.**
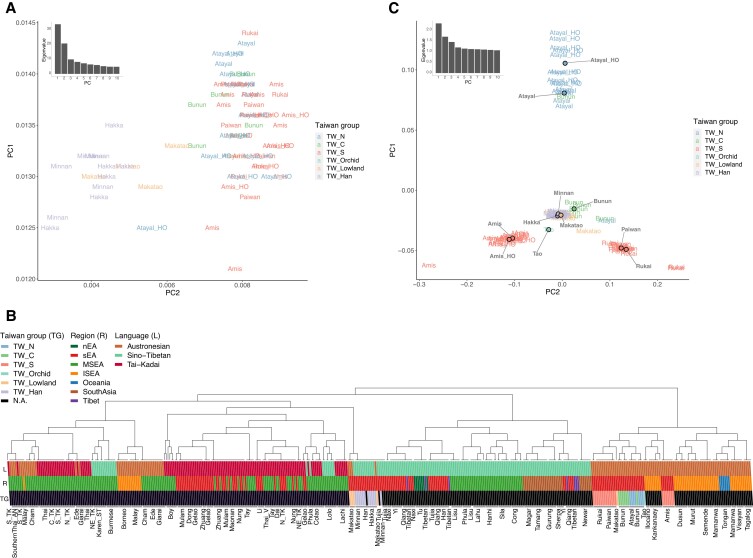
Structure within Taiwan. A) Zoom-in of the PCA plot in Fig. 1B with a focus on the Taiwanese individuals, labeled by populations and colored by groups. B) FineStructure clustering of the Sino-Tibetan, Tai-Kadai, and Austronesian groups based on haplotype painting profiles from ChromoPainter. Each vertical line represents an individual, colored according to regions (R), Taiwan groups (TG), and languages (L). Populations are labeled at the bottom. Note that the clustering does not necessarily imply phylogenetic structure. C) PCA for which eigenvalues were computed using only the THI/TOI individuals, with the Han and Lowland individuals projected. Individuals are labeled by groups and the median positions of individuals of each group are plotted with dots colored by groups. The eigenvalues from PC1 to PC10 are shown on the top-left. TW_N, TW_C, and TW_S denote northern, central, and southern Taiwanese Highland groups, respectively.

In the context of Asian and Oceanian genetic variation, the genetic profile of the THI/TOI was relatively homogenous with little structure revealed, except that they were separated from the Han groups, with the Lowland group falling in between them (Fig. 3A). To further study the structure within Taiwan, we applied the haplotype-based method fineSTRUCTURE (31). To provide haplotype sources from neighboring groups, we carried out chromosome painting on all of the Austronesian, Tai-Kadai, and Sino-Tibetan groups in our data set (Fig. 3B), as these language families are proposed to be related (9). The fineSTRUCTURE results generally supported clustering according to language family except that groups from MSEA showed a heterogenous pattern, which is consistent with previous studies suggesting complex histories involving extensive admixture and probable cases of language shift (32, 33). Within Taiwan, there was a clear separation between the THI/TOI and Lowland/Han groups; the former clustered with other Austronesian groups, while the latter clustered with the Sino-Tibetan and Tai-Kadai groups (Fig. 3B). With respect to the THI/TOI groups, the northern (Atayal) and central (Bunun) groups clustered in a clade. There was a division in the southern groups (Rukai, Paiwan, and Amis): the Rukai and Paiwan grouped together, while the Amis grouped together in another clade with the Orchid Island group (Tao) and the Filipino groups Kankanaey and Ilocano.

Given the structure revealed in the THI/TOI groups is distinct from the Lowland/Han groups, we performed a PCA with eigenvalues computed using only the THI/TOI individuals and projected the Lowland/Han individuals (Fig. 3C). PC1 separated the northern group Atayal from the southern groups, and PC2 separated the Amis from the Rukai and Paiwan. The central group Bunun fell toward the Atayal, the Orchid Island group Tao fell toward the Amis, and the Lowland group Makatao was heterogeneous, with most individuals grouping with the Han groups in the middle, although some were positioned toward the Rukai/Paiwan. Notably, the Atayal and Amis from the published data set grouped together with the Atayal and Amis genotyped in this study. The fact that the Atayal, Amis, and Rukai drive the poles of this PCA probably reflects their isolation and drift, as they showed high amounts of within-group identity-by-descent (IBD) sharing (Fig. S5).

Both ADMIXTURE and PCA results showed that the Lowland group Makatao had an intermediate genetic profile between the THI/TOI and Han groups (Figs. 2 and 3A), suggesting admixture. To test this possibility, we computed the *f*3 admixture value *f*3(Minnan, Rukai; Makatao) and obtained *Z* = −7, revealing admixture in the Makatao involving these two proxy sources. The Rukai were chosen as the Austronesian source proxy because the Makatao were projected toward the Rukai pole in the PCA of THI/TOI (Fig. 3C). This decision was further supported by a best-fitting admixture graph of the Mbuti (outgroup), Lowland Makatao, Han groups, and three representative THI groups (Atayal, Amis, and Rukai): the Makatao were modeled as having 42% ancestry from the ancestor of the THI group Rukai and 58% from the ancestor of the Han group Minnan (Fig. S6). Using GLOBETROTTER (34), we inferred that this admixture resulted from a major recent pulse event dating to ∼1.64 ± 0.93 generations ago (∼50 years ago assuming 30 years per generation). Overall, we observed three distinct genetic clusters in the THI/TOI groups and an admixture event between the THI and Han groups in the Lowland group.

### Genetic structure of Taiwanese Austronesian groups: implications for Into-Taiwan

With a better understanding of the genetic structure within Taiwan, we investigated the genetic profiles of the ancestors of Taiwanese Austronesians or proto-Austronesians. We first focused on ancient genomes from or near Taiwan: the Hanben from northeastern Taiwan, dated to ∼1.5 kya; the Suogang from Penghu Island offshore from southwestern Taiwan, dated to ∼4.5 kya; and the Liangdao from Liangdao Island offshore from northwestern Taiwan, dated to ∼7.7 kya (10, 11). Among all projected ancient genomes on the PCA of the THI/TOI, the Suogang and Liangdao individuals, who are thought to be related to proto-Austronesians (10), were positioned toward the Rukai pole, while the Hanben individuals were closer to the Atayal pole (Fig. S7).

Previous studies used qpAdm with ancient genomes as sources and outgroups to model present-day East Asians, including the Amis and Atayal (10, 11). However, Yang et al (10) modeled the Amis as a pure sEA source and the Atayal as an admixture of sEA and nEA sources, while Wang et al. (11) only included the Amis and modeled them as an admixture of sEA and nEA with different sources and outgroups. We investigated this framework and extended the analysis to the other Taiwanese groups in our study, using the sources and outgroups in Yang et al. (10) as these were more clearly defined. There was one modification (Fig. S8): we substituted the 7 kya Pha Faen genome (35) (which overlaps with the Onge in terms of Hoabinhian-related ancestry) with the 10 kya Longlin genome (36) to provide more distinct sEA outgroups.

**Fig. 4.**
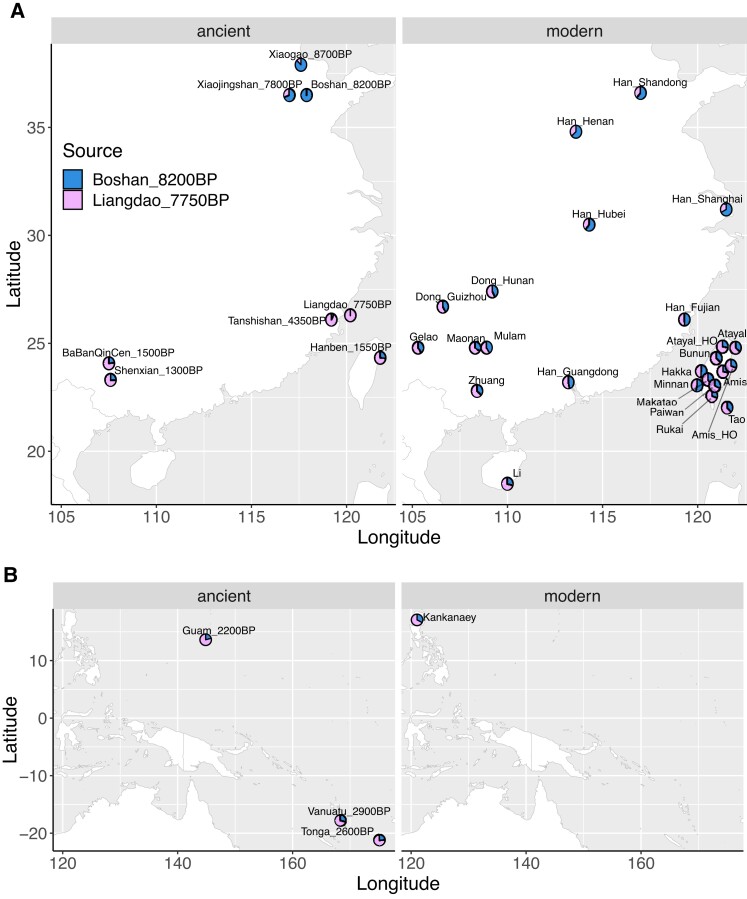
qpAdm modeling of the spatiotemporal dynamic of nEA versus sEA ancestries in East Asia and Oceania. Using outgroups shown in Fig. S8, selected groups from (A) nEA and sEA as well as (B) ISEA and Oceania (with a focus on those related to the early Out-of-Taiwan groups) are modeled with nEA (in blue, the ∼8.2 kya Boshan as proxy) and sEA (in pink, the ∼7.7 kya Liangdao as proxy) sources. The left panels show ancient groups while the right panels show modern groups. Bar plot visualization with the standard errors of the estimates is shown in Fig. S9.

Regarding the selected ancient groups, the ∼4.3 kya Tanshishan group from southern China, which is closely related to the ∼4.5 kya Suogang group (due to low coverage, we did not have enough power to model this group), was modeled as having mainly sEA (7.7 kya Liangdao) ancestry and a small proportion (∼8%) of nEA (8.2 kya Boshan) ancestry, while the younger sEA and Taiwan groups had more (∼23–26%) nEA ancestry (Figs. 4A and S9). Likewise, we modeled all selected modern groups (including Taiwanese groups) as an admixture of ancient nEA and sEA ancestries, with the nEA ancestry further increasing (∼28–50%) in the present-day sEA samples (Figs. 4A and S9). The sEA ancestry was also present at a low frequency in the ancient nEA groups and increased in the present-day samples, consistent with previous studies (10, 11). A best-fitting admixture graph had the ∼1.5 kya Hanben group modeled as an admixture of a nEA source (ancestor of the 8.3 kya Boshan) and a sEA source (ancestor of the 7.7 kya Liangdao). Similarly, the THI Austronesians (Atayal, Amis, and Rukai) and the Tai-Kadai speaking Li were modeled as an admixture of shared nEA (ancestor of the Han from Shandong) and sEA (ancestor of the 7.7 kya Liangdao) ancestries, while the Li received additional sEA-related ancestry from an unsampled group (Fig. S10).

### Genetic structure of Taiwanese Austronesian groups: implications for Out-of-Taiwan

We next investigated the relationships between Taiwanese Austronesian groups and groups in ISEA and Oceania, to gain insights into the Out-of-Taiwan event(s). The Lapita-related ancient groups (∼2–3 kya Guam, Vanuatu, and Tonga), who are thought to be related to the Out-of-Taiwan Austronesians, showed a similar profile to the younger (∼1.5 kya) sEA ancient groups, while the present-day Filipino Kankanaey, who are an isolated group often used as a modern proxy for the Out-of-Taiwan group, were more similar to the THI/TOI groups (Figs. 4B and S9). Together, these results suggested that, intriguingly, the early Out-of-Taiwan (Lapita) groups already possessed increased nEA ancestry compared with the early Into-Taiwan (sEA) groups (∼21–29% vs. ∼0–8%), and the nEA ancestry further slightly increased in the present-day THI/TOI (∼28–37%) and Kankanaey (∼33%) groups. Focusing on how these groups (as well as other ISEA and Oceanian groups) project on the THI/TOI PCA, we found that they were generally projected toward the southern groups Amis and Rukai and slightly more shifted to the Amis (Fig. S11).

**Fig. 5.**
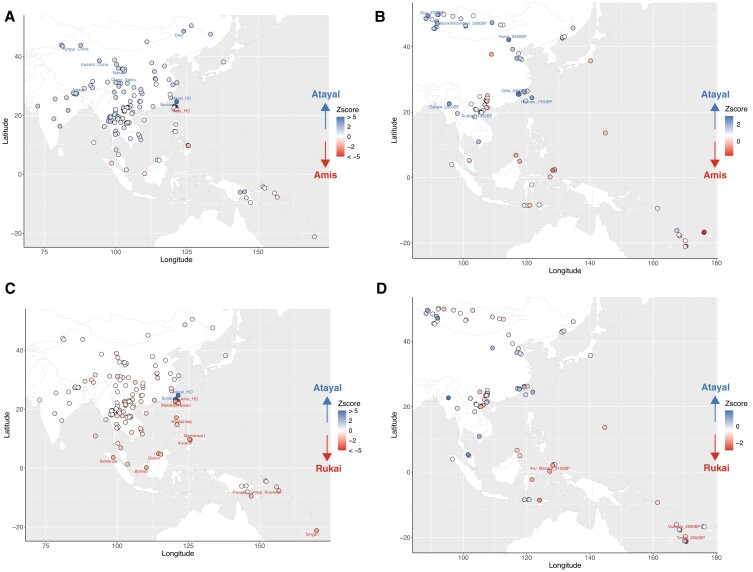
Differential allelic sharing to the modern/ancient groups from East Asia and Oceania between the Atayal and Rukai/Amis. Results of the form *f*4(Atayal, X; Y, Mbuti) where X is Amis (A and B)/Rukai (C and D) and Y are modern (A and C)/ancient (B and D) groups from East Asia and Oceania. The modern/ancient groups are plotted as dots on the map, colored in proportion to *Z* score. Positive values (in blue) indicate more sharing with the Atayal while negative values (in red) indicate more sharing with the Amis/Rukai. Significant values (absolute *Z* score value ≥ 2) are further labeled with population names. For the comparisons with ancient groups, additional tests using only transversions and the French as an outgroup are shown in Fig. S12, which reduce not only the potential for false positives caused by DNA damage and/or attraction to deep outgroups but also the statistical power due to the decreased number of SNPs.

Results of *f*4 statistics of the form *f*4(Atayal, Amis/Rukai; modern/ancient groups, Mbuti) further supported the observation that, compared with the northern group Atayal, the southern groups Amis and Rukai had excess sharing with the modern/ancient ISEA and Oceanian groups (Figs. 5 and S12). More precisely, the Atayal showed significant excess sharing with the mainland sEA modern/ancient groups compared with the Amis (Fig. 5A and B), while the Rukai showed significant excess sharing with the ISEA and Oceania groups compared with the Atayal (Fig. 5C and D). We finally tried to determine whether the ancestral Out-of-Taiwan groups were closer to the Amis or Rukai with an *f*4 statistic of the form *f*4(Amis, Rukai; modern/ancient groups, Mbuti). We found that the values were mostly negative (Fig. S13), suggesting that most populations shared excess ancestry with the Rukai. In keeping with these *f*4 results, a best-fitting admixture graph modeled the Atayal as being closest to the Into-Taiwan group (sEA Li) and the Rukai as closest to the Out-of-Taiwan group (ISEA Kakanaey), with the Amis falling in-between (Fig. S14).

**Fig. 6.**
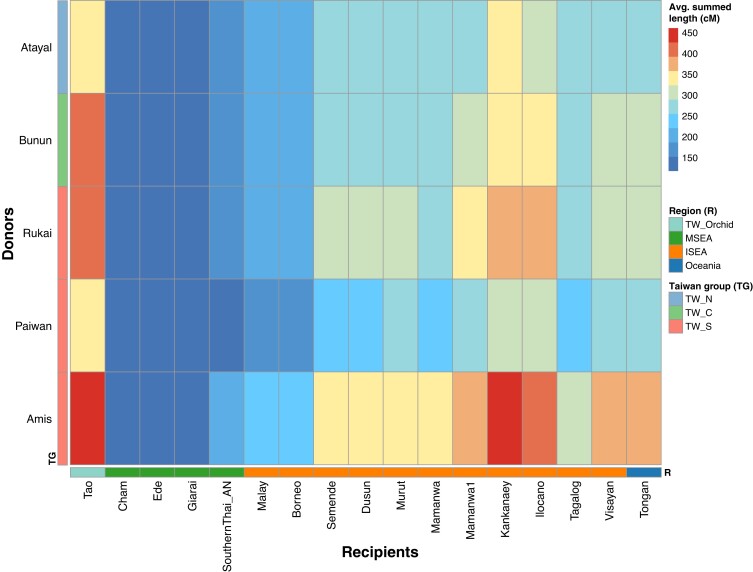
Chromosomal painting of the Malayo-Polynesian Austronesian recipients by the Formosan Austronesian donors. Results show the painting profile of recipients (in columns) by donors (in rows) colored in proportion to the average of the summed length (cm) of painted chromosomal regions, corresponding to the extent of haplotype sharing. The Taiwan group (TG) for the donors and the region (R) for the recipients are denoted by the colored keys. TW_N, TW_C, and TW_S indicate northern, central, and southern Taiwanese Highland groups, respectively.

We further applied haplotype-based approaches, which enrich the signal of recent genetic sharing, to investigate the affinities between Taiwanese Austronesian and Out-of-Taiwan-related groups. Although results of frequency-based methods (i.e. *f*4 and qpgraph) indicated that the Rukai might be the closest to the Out-of-Taiwan groups, the fineSTRUCTURE (haplotype-based) results clustered the Amis with the Tao and Kankanaey, suggesting the Amis might be the closest to the Out-of-Taiwan-related groups from a haplotype-based perspective. We therefore performed another ChromoPainter analysis using only the THI (Formosan branches) groups as the source to paint other Austronesians (Malayo-Polynesian branch) and indeed found that the Amis shared most with other Austronesian groups, followed by the Rukai (Fig. 6).

We also investigated the sharing of IBD genomic segments in three main size ranges: 1 to 5 cm, 5 to 10 cm, and over 10 cm, roughly corresponding to ∼2.7 kya, ∼675 ya, and ∼225 ya, respectively (37). Most of the signals were enriched in the size range of 1 to 5 cm (Fig. S15), while the THI groups showed strong within group sharing and regional structure in the longer size ranges (Figs. S16 and S17), as found in the previous analyses of the genetic structure within Taiwan (Figs. 3 and S5). Similarly, the Lowland Makatao also showed an intermediate sharing profile between the Han and THI/TOI groups in the size range of 1 to 5 cm (Fig. S15). In this shortest size range, the THI/TOI groups only showed notable sharing (the log value of average of the summed IBD length ≥ 1 cm) with the Austronesian groups, except that they also shared with the Taiwanese Han (Hakka) and Papuan groups (Nasioi, Papuan Central Province, Papuan Gulf Province, and Vella Lavella) with Austronesian admixture (Fig. S3). Finally, the IBD results indicated that the southern groups shared more with the Out-of-Taiwan groups than did the northern/central groups; e.g. the log value of the average summed IBD length for the Rukai/Amis versus Kankanaey was greater than 3 cm and for the Rukai/Amis versus Papuan Central Province greater than 2 cm, while for the Atayal/Bunun, these values were lower (Fig. S15).

## Discussion

In this study, we generated new genome-wide data for 55 Taiwanese individuals (43 Austronesians) to characterize the genetic structure of Taiwanese and address how this structure impacts questions regarding the Into- and Out-of-Taiwan migrations. Here, we highlight and discuss our most important findings concerning the genetic structure of THI/TOI groups, the genetic profile of Lowland groups, and the Into-Taiwan and Out-of-Taiwan events.

### Genetic structure of Taiwan

The linguistic diversity of the Formosan branches of the Austronesian language family (2, 38) and the distinct cultures of indigenous Taiwanese groups (CIP: https://www.cip.gov.tw) are suggestive of population structure within Taiwan, particularly for THI/TOI groups. Prior research into mitochondrial DNA (mtDNA) variation in the Taiwanese groups revealed a cline of decreasing genetic diversity from north to south, with the groups further subdivided into northern, central, and southern clusters based on patterns of mtDNA sequence sharing (6). Furthermore, the central group is more closely related to the northern group than the southern groups are, and the southern groups can be further subdivided, as shown by our genome-wide data (Fig. 3B and C). In fact, the mtDNA study found that the northern group Atayal and the central group Bunun shared some haplotypes while the southern groups Rukai/Paiwan and Amis had distinct haplotype profiles (6).

In addition, our PCA results for the THI/TOI groups suggest a major structure driven by northern versus southern groups (PC1) and a subdivision in southern groups (PC2). This is represented by the poles of the Atayal, Rukai, and Amis (Fig. 3C), which also represents three distinct Formosan linguistic branches (the Atayalic, Rukai, and East Formosan, respectively). The main factor shaping this structure is probably isolation between the groups, as shown by the high levels of IBD sharing within them (Fig. S5). The fact that the Atayal exhibit the highest levels of within-group IBD sharing (Fig. S5) may also explain why they are the most distinct of the THI/TOI groups in PCA and ADMIXTURE analyses (Figs. 2 and 3C). The Atayal have their own distinct haplotype network, according to a previous Y-chromosomal DNA study (24), suggesting they experienced founder/isolation events. The genetic structure observed within Taiwan could have formed (or strengthened) within the past 3 kya, as the signal can be identified in all size ranges of IBD sharing between/within the THI/TOI group (Figs. S15–S17), in line with the ∼1–3 kya time estimate, based on mtDNA, for the formation of different groups (6). However, not all THI/TOI groups are represented in our analyses, indicating that further studies including these unsampled groups are needed to verify and extend our observations.

### Genetic profile of a Taiwan Lowland group

There are around 12 identified Lowland Austronesian groups (belonging to the West Plains, Northwest Formosan, and East Formosan branches) (2), but only one is represented in our data. As the Lowland groups are even less studied than the THI/TOI groups, our data provide the first genome-wide characterization of a Lowland group. In contrast to the more isolated Highland groups, Lowland groups are known to have had extensive contact with Han groups (39). They share a similar demographic pattern of population size change with the Han that differs from the THI groups with respect to mtDNA (6) and larger population size and higher frequencies of haplogroup sharing with respect to the Y chromosome (24). Our data provide evidence of autosomal admixture between the THI and Han groups for the Lowland group Makatao (Figs. 2, 3A, and S6), with the Rukai as the best proxy for the THI source (Figs. 3C and S6). Although the Makatao language belongs to the East Formosan branch and the Rukai language belongs to the Rukai branch, the sampling locations of the two groups are in geographic proximity (Fig. 1A), which might explain their genetic similarity and reflect their recent local contact through the sharing of IBD in the size range of 5 to 10 cm (Fig. S16).

Evidence for ongoing gene-flow between the Han and Austronesian groups is suggested by the inferred admixture date of ∼50 years ago, which may be the most recent admixture date captured by our dating methods. Given that the sampling of the Makatao was done from 1998 to 2001 (6), this admixture date would correspond to ∼1950, which coincides with the timing of the retreat of the government of the Republic of China (and massive migration of the Han) to Taiwan (40). This was also one of the most recent historical events, after the Kingdom of Tungning (1661–1683) and rule of the Qing dynasty (1683–1895), that promoted intermarriage between Han and Austronesian groups (40–42), which could therefore account for the admixture signal.

In contrast to the dating results, we did not find any evidence of IBD sharing between the Makatao and Han groups in the longer size range. We speculate that our sample size may not have been large enough to detect IBD in expanding populations (like the Han). Since there were traceable amounts of an Austronesian-related component in the Taiwanese Han groups (particularly the Hakka), slightly more than in the Chinese Han groups, we speculate that this gene-flow was bidirectional (Figs. 2, S3, and S15). Yet, given the weakness of this signal, we cannot model this admixture in the Han groups using our admixture graph analyses. However, we do find that the Hakka cluster more closely with the THI groups than the Minnan do (Fig. S6). Furthermore, a recent study of Taiwanese Han from the Taiwan biobank found evidence of genetic sharing between the Taiwanese Han and Austronesian groups (43). Additional studies of Taiwan's Lowland groups are clearly needed to clarify these population relationships.

### Implications for Into-Taiwan

The Into-Taiwan event describes the ancestors of Taiwanese Austronesians from wherever they originated. Linguistic studies indicate that “proto-Austronesian” likely formed in the southeast coast of mainland China (44). Archeological studies of cultivated rice and millet show a link from southeastern China to Taiwan and then to Southeast Asia (8), which further supports a southeast Chinese origin of the ancestors of Austronesians under the “farming-language dispersal” model (4). mtDNA and Y chromosome studies also suggest links between southeastern China and Taiwan (6, 24). Ancient DNA studies have confirmed a genetic link between ancient groups from the southeast Chinese coast and Taiwanese Austronesians (10, 11). However, these ancient DNA studies only included the Amis/Atayal, resulting in inconsistent estimates of nEA versus sEA ancestry (10, 11).

Our results add context to the published ancient genomes from or near Taiwan by showing that the ∼1.5 kya Hanben individuals have genetic affinities with the northern group Atayal while the ∼7.7 kya Liangdao and ∼4.5 kya Suogang individuals are slightly closer to the southern group Rukai (Fig. S7). The Atayal's traditional territory overlaps with the Hanben archaeological site in Yilan County in northern Taiwan, suggesting a familial relationship between the two groups. In contrast, the Liangdao and Suogang archaeological sites, located on islands off western Taiwan, are far from the active area of the Rukai. One potential explanation for their affinities to the Rukai might be that the Rukai are more closely related to the early Into-Taiwan groups that swiftly moved from the north to the south, as suggested by the mtDNA study (6). In fact, linguistic studies indicate that the Rukai language is the earliest Formosan branch to split off (45, 46).

We also revisited the modeling of nEA versus sEA sources in the THI/TOI groups and surrounding ancient/modern groups and confirmed that all of the groups tested can be modeled as having nEA (8.3 kya Boshan) and sEA (7.7 kya Liangdao) ancestries (Figs. 4, S9, and S10). This finding indicates that the “proto-Austronesian” and Austronesian-related genetic components are both a mixture of nEA and sEA ancestries. Moreover, compared with the Into-Taiwan groups (7.7 kya Liangdao and 4.3 kya Tanshishan), the 1.5 kya Hanben and present-day THI/TOI groups show an increased amount of the nEA source, suggesting an additional influx of nEA ancestry Into-Taiwan after the Neolithic expansion, in line with ancient DNA studies showing post-Neolithic gene-flow between nEA and sEA (10).

Interestingly, the early Out-of-Taiwan groups (2.2 kya Guam, 2.6 kya Tonga, and 2.9 kya Vanuatu) show more nEA ancestry than the early Into-Taiwan groups but less than the THI/TOI groups and Kankanaey; the Atayal, Bunun, and Tao show the most nEA ancestry among these groups (Figs. 4 and S9). This pattern suggests that either more nEA gene-flow Into-Taiwan occurred prior to the Out-of-Taiwan expansion or there are unsampled Into-Taiwan groups with more nEA ancestry than the early Out-of-Taiwan groups. Moreover, the present-day northern THI/TOI groups have had additional contact with nEA-related groups.

Previous research has indicated a close linguistic and genetic relationship between Austronesian and Tai-Kadai speakers (9, 11, 32, 37). Linguistic studies raise two hypotheses for this relationship: a shared ancestor for “proto-Austronesian” and “proto-Tai-Kadai” or an Austronesian group from Taiwan that returned to mainland China and became the ancestors of Tai-Kadai (9, 47). Our admixture graph result favors the former hypothesis, as the Tai-Kadai speaking Li, who have been shown to be an unadmixed proxy for Tai-Kadai ancestry (48), share the same source of nEA ancestry with the Austronesian groups rather than nesting within them. Furthermore, the Li even independently received additional ancestry from the ancestral sEA branch (Fig. S10).

### Implications for Out-of-Taiwan

The Out-of-Taiwan event describes the migration of people from Taiwan to ISEA and Oceania, coinciding with the spread of Austronesian languages and agriculture. This migration and spread of culture have been confirmed by archeological, linguistic, and genetic evidence (3, 4, 6, 7, 49); in particular, mtDNA and Y chromosome studies have suggested that Taiwan is the source of some haplogroups in Austronesian groups in ISEA and Oceania, such as mtDNA haplogroup E1a and some subhaplogroups of Y chromosome haplogroup O1a2 (6, 24, 50, 51). However, the support for a major contribution in the uniparental markers from Taiwan and the mode/tempo of the migration remain debated (52–54), and the way it relates to the structure within Taiwan is largely unexplored. In line with uniparental marker data (6, 55), our results support a closer relationship between the Out-of-Taiwan and southern THI/TOI groups (Figs. 5, 6, S11, and S14). However, different results point to different southern groups as being the closest to the Out-of-Taiwan groups. The haplotype-based results favor the Amis being closer to the Out-of-Taiwan groups (Figs. 3B and 6), which is supported by a recent linguistic study suggesting that the East Formosan branch (which the Amis language belongs to) is the closest to the Out-of-Taiwan, Malayo-Polynesian branch (56). However, the allele-based *f*4 comparisons show that the Rukai share more ancestry with the Out-of-Taiwan groups than the Amis (Fig. 5), a result consistent with the allele-based admixture graph (Fig. S14). Because the haplotype-based analyses enrich the signal of recent contact while the allele-based analyses capture the average of overall sharing during population history, they suggest that, with the Out-of-Taiwan groups, the Amis received additional recent contact while the Rukai retained on average high genetic affinities throughout their history.

A further potential complication is that recent genetic studies have estimated an older divergence time between the THI and Filipino groups than the linguistic Out-of-Taiwan time estimate and suggested that the spread of Austronesian languages might not be associated with the migrations of people (16, 17). Given that the Amis and Paiwan (close to the Rukai) used in their models are southern groups, this older estimate is not due to using a more distant THI group to the Out-of-Taiwan groups. However, even a small amount of additional interactions between the THI and nEA-related groups compared with the Filipino groups could inflate the estimate, as the inclusion of a nEA source in the model expands the confidence interval to overlap with the linguistic Out-of-Taiwan time (17). In our study, we do see nEA sources modeled for all THI groups (Figs. 4 and S9).

In summary, the concise synthesis of our results would be as follows. First, the early Into-Taiwan groups rapidly moved to the south and became the Out-of-Taiwan groups. This conclusion is supported by our DyStruct results indicating that the early sEA groups share similar profiles with the early Oceanian (Lapita-related) groups (Fig. 2) and by the previous mtDNA study (6). Second, given that there was little divergence between the Into- and Out-of-Taiwan groups, our results of the southern group Rukai retaining excess allelic sharing with both Into- and Out-of-Taiwan groups (Figs. 4, S7, and S13) might reflect that their ancestors underwent less genetic drift than the ancestors of other present-day Taiwan groups. Alternatively, the ancestors of the Rukai might actually be the closest to the Into-Taiwan (which soon became out-of-Taiwan) groups, which is in line with the linguistic evidence that they are the earliest diverged groups (45, 46). Third, populations further diverged within Taiwan after these early events, as suggested by IBD results (Figs. S15–S17) and mtDNA (6), with the northern group Atayal experiencing the strongest bottleneck/isolation (Figs. 2, 3C, and S5). Fourth, the closer haplotype sharing (and perhaps the linguistic relationship (56)) between the southern group Amis and the Out-of-Taiwan groups might result from recent back migration and contact (Figs. 3B and 6). The East Formosan Amis are close to the Malayo-Polynesian Tao geographically and genetically, based on our results (Figs. 3 and 6) as well as previous findings (28). Moreover, it has been suggested that the Orchid Island group Tao might be derived from a back migration from ISEA (57). Finally, during even more recent times, the Lowland groups interacted extensively with the Han groups, resulting in an admixed genetic profile (Figs. 2, 3A, and S6). In any case, more whole genome sequencing of THI/TOI groups would improve the power of more sophisticated analyses to test these models, along with more ancient genomes from Into-Taiwan, Taiwan, and Out-of-Taiwan groups.

## Materials and methods

### Sample and data information (details in Supplementary Material, Extended Materials and Methods)

Sampling of Taiwanese individuals was done in Ko et al. (6); we selected a total of 43 Austronesian (37 highlanders from Atayal, Bunun, Rukai, Paiwan, Ami; 1 Tao; and 5 Makatao lowlanders) and 12 Taiwanese Han (Hakka; Minnan) individuals and generated their genome-wide data on the Affymetrix Human Origins array. The ethics committees of the China Medical University, the Taiwan National Health Research Institutes, and the University of Leipzig Medical Faculty have approved this study. Informed consent was obtained from all participants. We merged our newly generated data with published modern and ancient data and performed quality control on the merged data set (Supplementary Material, Extended Materials and Methods), resulting a filtered data set consisting of 540,691 SNPs and 1,958 modern and ancient individuals. Metadata are in Table S1.

### Population genetic analyses (details in Supplementary Material, Extended Materials and Methods)

PCA was done with smartpca v16000 (58). Model-based clustering was done by ADMIXTURE v1.3.0 (29) and DyStruct v1.1.0 (30). We used admixr v0.9.1 (59) to compute *f*3 and *f*4 statistics and qpAdm from ADMIXTOOLS v7.0.2 (26). Phasing was done by SHAPEIT version 4.1.3 (60) with the reference panel and the recombination map from the 1,000 Genomes Phase3 (61). We ran ChromoPainter v2 (31), fineSTRUCTURE v4.0.1 (31), and GLOBETROTTER (34) to infer the haplotype sharing, fine-scale structure, and admixture dates. We identified shared IBD blocks using RefinedIBD (62). We used ADMIXTOOLS 2 (63) to model admixture graphs.

## Supplementary Material

pgad122_Supplementary_Data

## Data Availability

The genome-wide SNP array data generated in this study are available from the European Genome-Phenome Archive (EGA; https://ega-archive.org), under accession code EGAS00001006911.
