# Supplementary material for: The genomic diversity of Taiwanese Austronesian groups: Implications for the “Into- and Out-of-Taiwan” models
Source: PNAS Nexus. 2023 May 16;2(5):pgad122. doi: 10.1093/pnasnexus/pgad122 (PMC10187666; doi:10.1093/pnasnexus/pgad122)
Supplement: pgad122_Supplementary_Data [file pgad122_supplementary_data.pdf]

PNAS nexus

Supplementary Information for

The genomic diversity of Taiwanese Austronesian groups: implications for the 'Into and Out of Taiwan' models

Dang Liu*, Albert Min-Shan Ko and Mark Stoneking*

*Corresponding authors: Dang Liu and Mark Stoneking
Email: dang.liu@pasteur.fr and stoneking@eva.mpg.de

This PDF file includes:

Supplementary text
SI References
Figures S1 to S17
Table S1

Supplementary Information Text

Extended Materials and Methods

Sample and data information

Sampling of Taiwanese individuals was done in Ko et al (1); we selected a total of 43 Austronesian (37 highlanders from Atayal, Bunun, Rukai, Paiwan, Ami; 1 Tao; and 5 Makatao lowlanders) and 12 Taiwanese Han (Hakka; Minnan) individuals and generated their genome-wide data on the Affymetrix Human Origins array. The ethics committees of the China Medical University, the Taiwan National Health Research Institutes, and the University of Leipzig Medical Faculty have approved this study. Informed consent was obtained from all participants. We first merged the new data with published data on the same array from comparative modern populations (2-9) and we kept only autosomal markers for our analyses. For quality control of the merged data of modern individuals, we excluded sites with more than 5% missing data in the entire dataset and sites with more than 50% missing data and/or Hardy-Weinberg equilibrium p values below 0.00005 within a group (except for groups with only one individual). We removed individuals with more than 5% missing data and we excluded one individual from 1st degree kinship pairs. Data missingness and Hardy-Weinberg equilibrium were calculated using PLINK v1.9 (10) while the individuals to be removed to avoid 1st degree relatedness (kinship coefficient >= 0.177) were inferred using KING (11), as implemented in PLINK v2 (12). This gives a filtered dataset of modern individuals consisting of 540,697 SNPs and 1,664 individuals. Next, we further merged the filtered dataset with comparative ancient genomes (5, 6, 9, 13-21). Six positions with more than two variants or that were inconsistent between the array and sequencing data were excluded. Ancient individuals with <15,000 informative sites were also removed. This gives a filtered dataset of modern and ancient individuals consisting of 540,691 SNPs and 1,958 individuals. For Principal Component Analysis (PCA), ADMIXTURE, and DyStruct analyses (described in below sections), variants were pruned beforehand for linkage disequilibrium using PLINK v1.9, excluding one variant from pairs with r2 > 0.4 within windows of 200 variants and a step size of 25 variants. Metadata are in Table S1.

PCA analyses

PCA was done with smartpca v16000 (22). Projection of groups on PCA was done with the “lsqproject” and “shrinkmode” options.

ADMIXTURE and DyStruct analyses

The clustering algorithm was first run by ADMIXTURE v1.3.0 (23). From K = 2 to K = 15, we performed 100 replicates for each K with random seeds. ADMIXTURE outliers with >0.01 of the turquoise (Han-related) component were excluded for the Amis (TA199, TA81), Bunun (bun55, bun32), Atayal_HO (NA13600), and Kankanaey (Igorot11) groups in the F-statistics based analyses (e.g., f3, f4, qpAdm, admixture graph, described in the below sections). We also performed DyStruct v1.1.0 (24) incorporating time-series data from ancient genomes for the model. We transformed the age of ancient genomes into generations using a generation time of 30 years (25) and binned every 50 generations into a time point using the script bin_sample_times.py. From K = 2 to K = 15, we performed 25 replicates for each K with random seeds. Then, we used the script plot_Q.py to plot the highest likelihood results for the best K of ADMIXTURE (K=9, with the lowest cross-validation error) and DyStruct (K=8, with the highest likelihood). Scripts bin_sample_times.py and plot_Q.py are provided by the authors of DyStruct (available here: https://github.com/tyjo/dystruct/tree/master/supp/scripts).

F3 and F4 statistics and qpAdm analyses

We used admixr v0.9.1 (26) to compute f3- and f4-statistics from ADMIXTOOLS v7.0.2 (2), with significance assessed through block jackknife resampling across the genome. We also used admixr to compute qpAdm from ADMIXTOOLS with option inbreed = "YES" (required if using a single individual as a source or outgroup). We merged additional outgroups (27-35) to follow Yang et al (18), with metadata available in Table S1. The target groups are all Taiwanese groups and selected Tai-Kadai (Dong_Guizhou, Dong_Hunan, Gelao, Li, Maonan, Mulam, Zhuang), Han (Han_Fujian, Han_Guangdong, Han_Shandong, Han_Shanghai, Han_Henan, Han_Hubei), ancient nEA (Xiaojingshan_7800BP, Xiaogao_8700BP, Wuzhuangguoliang_5000BP) and ancient sEA (Suogang_4550BP, Xitoucun_4500BP, Tanshishan_4350BP, Hanben_1550BP, Shenxian_1300BP, BaBanQinCen_1500BP) groups; the source groups are nEA Boshan_8200BP and sEA Liangdao_7750BP; the outgroups are Mota_4470BP, Ust_Ishim_44350BP, Kostenki_38050BP, Iran_10000BP, Yana_31850BP, Karelia_8450BP, Okunevo_4300BP, Indus_Periphery_4500BP, New_Guinea_Highlander, Onge, Upward_Sun_River_11400BP, Tianyuan_40000BP,

Longlin_10550BP, Kolyma_9750BP, Jomon_2800BP, Qihe_11550BP, Bianbian_9500BP, Mbuti, French, Australian, Tu, She, Ami, Kinh, and Baining. In contrast to the outgroups used in Yang et al, we substituted the 7 kya Pha Faen genome (14) (which overlaps with the Onge in terms of Hoabinhian-related ancestry) with the 10 kya Longlin genome (19) to provide more distinct sEA outgroups. We excluded results with negative values inferred for either one of the sources, p-values > 0.05, or the absolute values of the standard error > 0.25.

Data phasing

Phasing was done by SHAPEIT version 4.1.3 (36), with the East Asian (without the Kinh Vietnamese merged in our data set) and South Asian populations as a reference panel, and the recombination map from the 1000 Genomes Phase3 (37). To prepare the reference panel, we extracted the East and South Asian individuals as well as the overlapping sites with our data for each chromosome from the 1000 Genomes Phase3 data using bcftools version 1.4 (http://samtools.github.io/bcftools/; last accessed 10 July, 2020). The phasing accuracy of SHAPEIT4 can be enhanced by increasing the number of conditioning neighbors in the Positional Burrows–Wheeler Transform (PBWT) on which haplotype estimation is based (36). We ran phasing with the options –pbwt-depth 8 for 8 conditioning neighbors and left other parameters as default.

ChromoPainter, fineSTRUCTURE and GLOBETROTTER analyses

ChromoPainter v2 (38) was run on the phased dataset focusing only on the Austronesian, Tai-Kadai, and Sino-Tibetan speaking groups, with sample sizes for each population group randomly down-sampled to 7 (all individuals were used for groups with sample sizes below 7). We began with 10 iterations of the EM (expectation maximization) process to estimate the switch rate and global mutation probability using chromosomes 1, 5, 10, 15, and 20. With the estimated switch and global mutation rates, we ran the chromosomal painting process for all chromosomes, which then gave the output for downstream analyses. We first attempted to paint the chromosomes of each individual, using all of the samples in the dataset as both donors and recipients, via the -a argument. The EM estimation of switch rate and global mutation probability were ~409.33 and ~0.00048, respectively, which were then used as the starting values for these parameters for all donors in the painting process. To specifically study the contribution of the THI groups (Formosan branches) as sources for other Austronesians (Malayo-Polynesian branch), we also performed another run using all the samples except for the non-THI Austronesians as both donors and recipients; the non-THI Austronesians were used only as recipients. The EM estimation of switch rate and global mutation probability for this analysis were ~ 430.08 and ~ 0.00064, respectively.

fineSTRUCTURE v4.0.1 (38) was run on the first ChromoPainter output using all of the samples in the dataset as both donors and recipients. First, 1 million burn-in steps were used (-x) and 1 million further iterations were sampled (-y) keeping every 10000th sample (-z). To infer a tree (-m T), we ran an additional 100,000 burn-in steps (-x) and used the maximum number of 3000 tree comparisons for splitting/merging (-t). We processed and plotted the results in R with the assistance of fineSTRUCTURE R Tools (https://people.maths.bris.ac.uk/~madjl/finestructure/finestructureR.html).

To investigate the admixture of Austronesian and Han ancestries in the Lowland group Makatao, GLOBETROTTER (39) was run on the second ChromoPainter output using the THI/TOI Austronesian, Tai-Kadai, and Sino-Tibetan groups as surrogates. We first tested the certainty and potential waves of admixture events, and then estimated the major and minor sources as well as the dates of admixture. A single pulse of admixture was inferred for the Makatao. The standard error was computed from the distribution of admixture dates accessed through 100 bootstraps. A generation time of 30 years was used for transforming generations to years (25).

Identity by descent (IBD) analyses

We identified shared IBD blocks between each pair of individuals and homozygous-by-descent (HBD) blocks within each individual using RefinedIBD (40). Both identified IBD and HBD blocks are considered as IBD blocks in our analyses, which is analogous to pairwise shared coalescence (PSC) segments in a previous study (41). The IBD blocks within a 0.6 cM gap were merged using the program merge-ibd-segments from the RefinedIBD website (https://faculty.washington.edu/browning/refined-ibd.html), allowing only 1 inconsistent genotype between the gap and block regions. We used IBD blocks at least 2 cM in length shared by individuals within a population to investigate the demography of each population group. Then, we used IBD blocks in 1-5 cM, 5-10 cM, and over 10 cM ranges to investigate the sharing between individuals from different populations in different time periods (41). To visualize the sharing between populations, the pairs with an average of at least 0.5 shared IBD blocks (i.e., on average half of the pairs share IBD blocks) were kept to reduce noise and false positives. The average

of the summed IBD length/number of blocks between two groups was calculated by dividing the summed IBD length/number of blocks between all pairs of individuals from the two groups by the product of the number of individuals from one group and the number of individuals from the other group.

Admixture graph analyses

We selected groups to test our questions concerning the admixture profile of the Lowland group Makatao (Fig. S6), and the Into-Taiwan (Fig. S10) and Out-of-Taiwan (Fig. S14) events. For ancient groups, we removed individuals with more than 30% missing data except for individual TON002 from ~2.6 kya Tonga, who is the highest quality ancient Lapita individual and is needed for the Out-of-Taiwan model. We used ADMIXTOOLS 2 (42) to compute pairwise f2 statistics between the groups using extract_f2() with maxmiss=1 (no SNPs excluded) and then extracted the allele frequency products from the computed f2 blocks using f2_from_precomp() with afprod=TRUE (which will result in more precise f4-statistics when there are large amounts of missing data, as described on the tool website: https://uqrmaie1.github.io/admixtools/). Finally, for each of the scenarios, we searched for the best-fitting admixture graph by running ten independent runs of find_graphs() and selected the one with the lowest score (computed through the residuals between the expected and observed f-statistics given the data). We further confirmed a fitting graph by testing the graph with the lowest score using qpgraph() to check if the absolute value of the worst-fitting Z score is below 3. We started with no migrations (numadmix=0) and then added migrations until we found a fitting graph, which we denoted as the best-fitting graph for that scenario. We also added different admixture constraints for searching the graphs depending on our questions; for the admixture profile of the Makatao, at least 1 admixture for the Makatao, and no admixtures for the Atayal, Rukai, and Amis; for the Into-Taiwan event(s), no admixtures for the ~8.2 kya Boshan and ~7.7 kya Liangdao; for the Out-of-Taiwan event(s), no admixtures for the New Guinea Highlander, Atayal, and Rukai.

SI References

1. A. M. Ko *et al.*, Early Austronesians: into and out of Taiwan. *Am J Hum Genet* **94**, 426-436 (2014).
2. N. Patterson *et al.*, Ancient admixture in human history. *Genetics* **192**, 1065-1093 (2012).
3. I. Lazaridis *et al.*, Ancient human genomes suggest three ancestral populations for present-day Europeans. *Nature* **513**, 409-413 (2014).
4. P. Qin, M. Stoneking, Denisovan Ancestry in East Eurasian and Native American Populations. *Mol Biol Evol* **32**, 2665-2674 (2015).
5. P. Skoglund *et al.*, Genomic insights into the peopling of the Southwest Pacific. *Nature* **538**, 510-513 (2016).
6. M. Lipson *et al.*, Ancient genomes document multiple waves of migration in Southeast Asian prehistory. *Science* **361**, 92-95 (2018).
7. D. Liu *et al.*, Extensive Ethnolinguistic Diversity in Vietnam Reflects Multiple Sources of Genetic Diversity. *Mol Biol Evol* **37**, 2503-2519 (2020).
8. W. Kutanan *et al.*, Reconstructing the Human Genetic History of Mainland Southeast Asia: Insights from Genome-Wide Data from Thailand and Laos. *Mol Biol Evol* **38**, 3459-3477 (2021).
9. C. C. Wang *et al.*, Genomic insights into the formation of human populations in East Asia. *Nature* **591**, 413-+ (2021).
10. S. Purcell *et al.*, PLINK: A Tool Set for Whole-Genome Association and Population-Based Linkage Analyses. *The American Journal of Human Genetics* **81**, 559-575 (2007).
11. A. Manichaikul *et al.*, Robust relationship inference in genome-wide association studies. *Bioinformatics* **26**, 2867-2873 (2010).
12. C. C. Chang *et al.*, Second-generation PLINK: Rising to the challenge of larger and richer datasets. *GigaScience* **4**, 1-16 (2015).
13. M. A. Yang *et al.*, 40,000-Year-Old Individual from Asia Provides Insight into Early Population Structure in Eurasia. *Current Biology* **27**, 3202-3208.e3209 (2017).
14. H. McColl *et al.*, The prehistoric peopling of Southeast Asia. *Science* **361**, 88-92 (2018).
15. M. Lipson *et al.*, Population Turnover in Remote Oceania Shortly after Initial Settlement. *Current Biology* **28**, 1157-1165.e1157 (2018).
16. C. Posth *et al.*, Language continuity despite population replacement in Remote Oceania. *Nature Ecology and Evolution* **2**, 731-740 (2018).
17. M. Lipson *et al.*, Three Phases of Ancient Migration Shaped the Ancestry of Human Populations in Vanuatu. *Curr Biol* **30**, 4846-4856 e4846 (2020).

18. M. A. Yang *et al.*, Ancient DNA indicates human population shifts and admixture in northern and southern China. *Science* **369**, 282-+ (2020).
19. T. Y. Wang *et al.*, Human population history at the crossroads of East and Southeast Asia since 11,000 years ago. *Cell* **184**, 3829-+ (2021).
20. I. Pugach *et al.*, Ancient DNA from Guam and the peopling of the Pacific. *Proc Natl Acad Sci U S A* **118** (2021).
21. S. Oliveira *et al.*, Ancient genomes from the last three millennia support multiple human dispersals into Wallacea. *Nat Ecol Evol* **6**, 1024-1034 (2022).
22. N. Patterson, A. L. Price, D. Reich, Population Structure and Eigenanalysis. *PLoS genetics* **2**, e190 (2006).
23. D. H. Alexander, J. Novembre, K. Lange, Fast model-based estimation of ancestry in unrelated individuals. *Genome research* **19**, 1655-1664 (2009).
24. T. A. Joseph, I. Pe'er, Inference of Population Structure from Time-Series Genotype Data. *Am J Hum Genet* **105**, 317-333 (2019).
25. J. N. Fenner, Cross-cultural estimation of the human generation interval for use in genetics-based population divergence studies. *Am J Phys Anthropol* **128**, 415-423 (2005).
26. M. Petr, B. Vernot, J. Kelso, admixr —R package for reproducible analyses using ADMIXTOOLS *Bioinformatics* 10.1093/bioinformatics/btz030, 1-2 (2019).
27. Q. Fu *et al.*, Genome sequence of a 45,000-year-old modern human from western Siberia. *Nature* **514**, 445-449 (2014).
28. A. Seguin-Orlando *et al.*, Paleogenomics. Genomic structure in Europeans dating back at least 36,200 years. *Science* **346**, 1113-1118 (2014).
29. M. Gallego Llorente *et al.*, Ancient Ethiopian genome reveals extensive Eurasian admixture throughout the African continent. *Science* **350**, 820-822 (2015).
30. I. Lazaridis *et al.*, Genomic insights into the origin of farming in the ancient Near East. *Nature* **536**, 419-424 (2016).
31. Q. Fu *et al.*, The genetic history of Ice Age Europe. *Nature* **534**, 200-205 (2016).
32. J. V. Moreno-Mayar *et al.*, Early human dispersals within the Americas. *Science* **362** (2018).
33. P. B. Damgaard *et al.*, The first horse herders and the impact of early Bronze Age steppe expansions into Asia. *Science* **360** (2018).
34. V. M. Narasimhan *et al.*, The formation of human populations in South and Central Asia. *Science* **365** (2019).
35. M. Sikora *et al.*, The population history of northeastern Siberia since the Pleistocene. *Nature* **570**, 182-188 (2019).
36. O. Delaneau, J. F. Zagury, M. R. Robinson, J. L. Marchini, E. T. Dermitzakis, Accurate, scalable and integrative haplotype estimation. *Nat Commun* **10**, 5436 (2019).
37. C. Genomes Project *et al.*, A global reference for human genetic variation. *Nature* **526**, 68-74 (2015).
38. D. J. Lawson, G. Hellenthal, S. Myers, D. Falush, Inference of Population Structure using Dense Haplotype Data. *Plos Genetics* **8** (2012).
39. G. Hellenthal *et al.*, A Genetic Atlas of Human Admixture History. *Science* **343**, 747-751 (2014).
40. B. L. Browning, S. R. Browning, Improving the accuracy and efficiency of identity-by-descent detection in population data. *Genetics* **194**, 459-471 (2013).
41. H. Al-Asadi, D. Petkova, M. Stephens, J. Novembre, Estimating recent migration and population-size surfaces. *PLoS genetics* **15**, e1007908 (2019).
42. R. Maier, P. Flegontov, O. Flegontova, P. Changmai, D. Reich, On the limits of fitting complex models of population history to genetic data. *bioRxiv* 10.1101/2022.05.08.491072 (2022).

Supplementary Figures

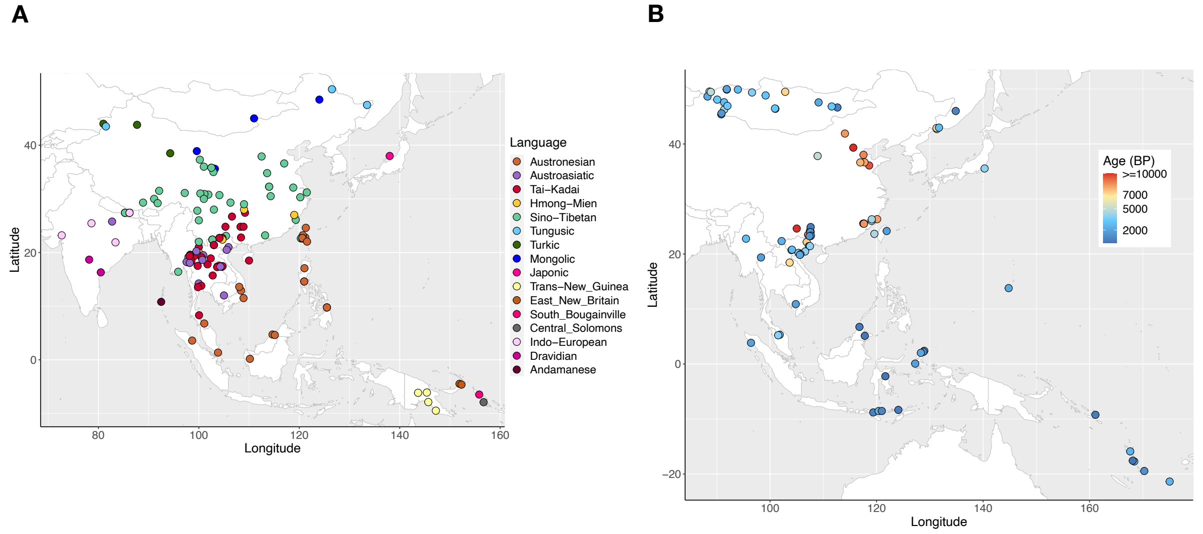

Fig. S1. Map of comparative data. Maps of comparative (A) modern and (B) ancient published data, colored by languages and sample ages, respectively. More details are in Table S1.

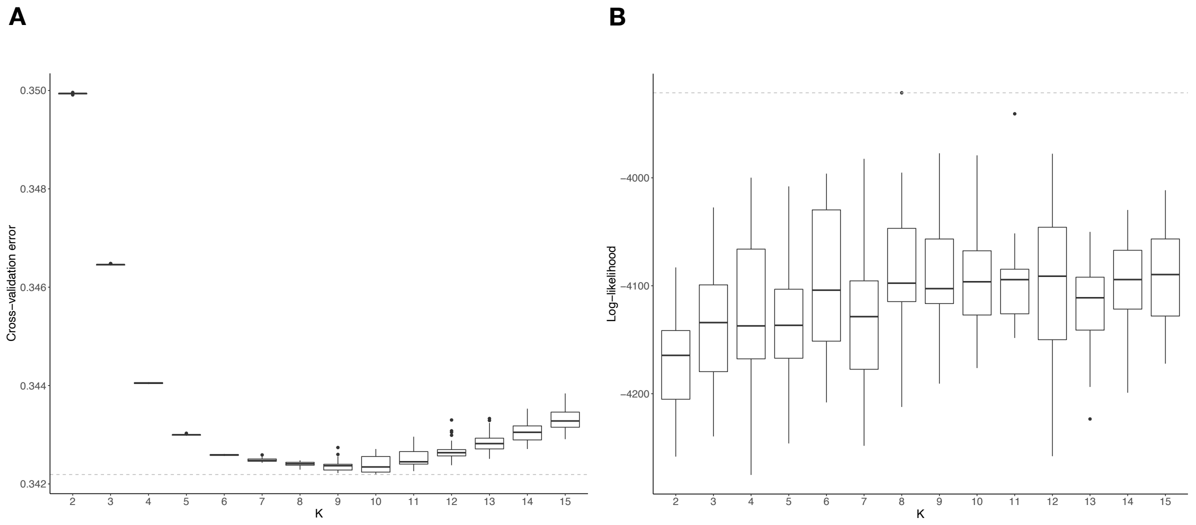

Fig. S2. Identifying the best-fitting K for ADMIXTURE and DyStruct analyses. (A) Cross-validation error plot for ADMIXTURE from K = 2 to K = 15. The best-fitting K (lowest cross-validation error) is K = 9. (B) Likelihood plot for DyStruct from K =2 to K = 15, indicating that the best-fitting K (highest likelihood) is K = 8. We ran 100 independent runs for ADMIXTURE (20 for DyStruct) for each K, and we show the runs with the highest likelihood for the best-fitting values of K in Fig. 2.

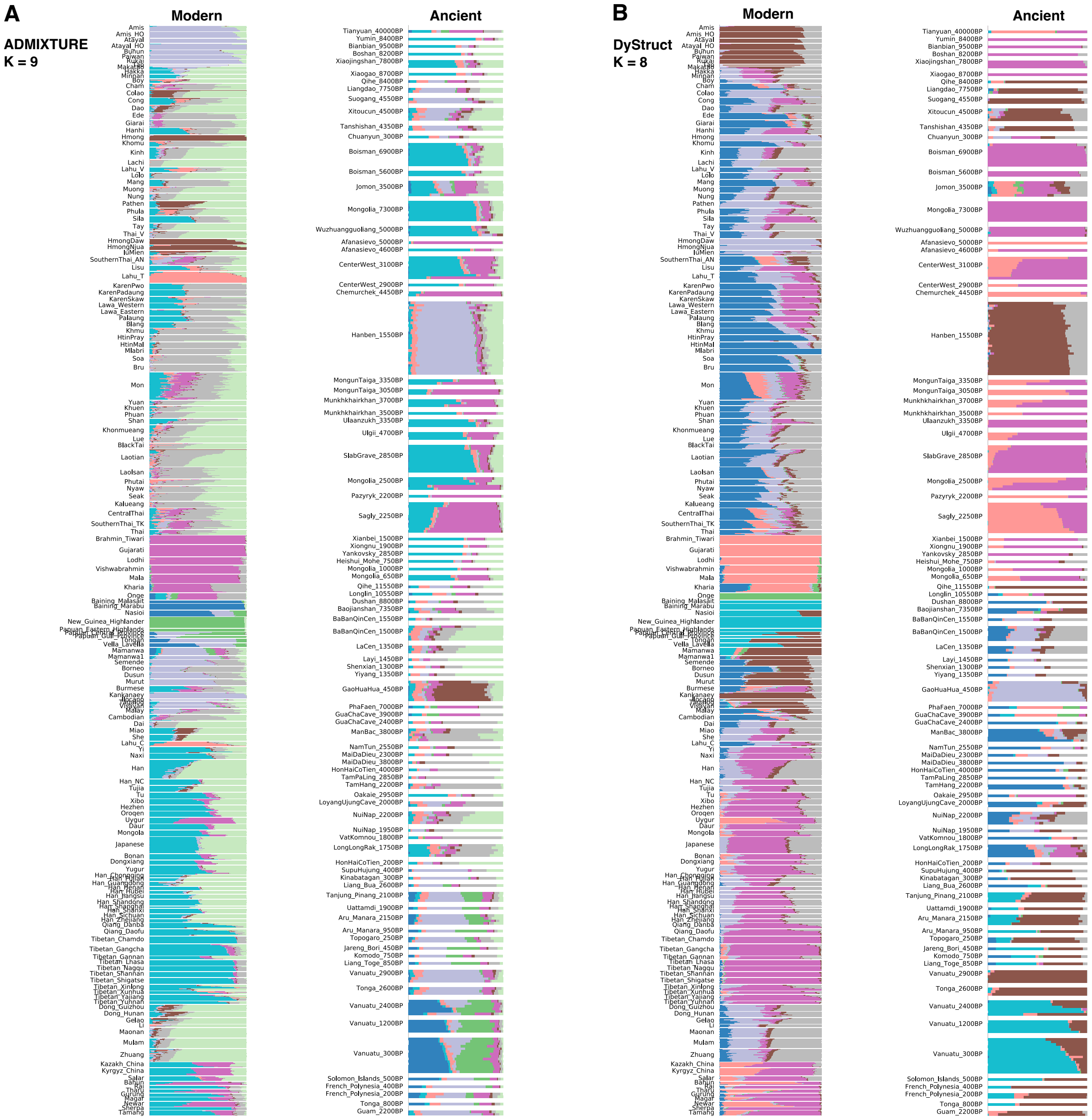

Fig. S3. Full results of the best-fitting K for the ADMIXTURE and DyStruct analyses. The highest likelihood run of (A) ADMIXTURE for K = 9 and (B) DyStruct for K = 8, with the modern groups listed on the left and the ancient samples on the right.

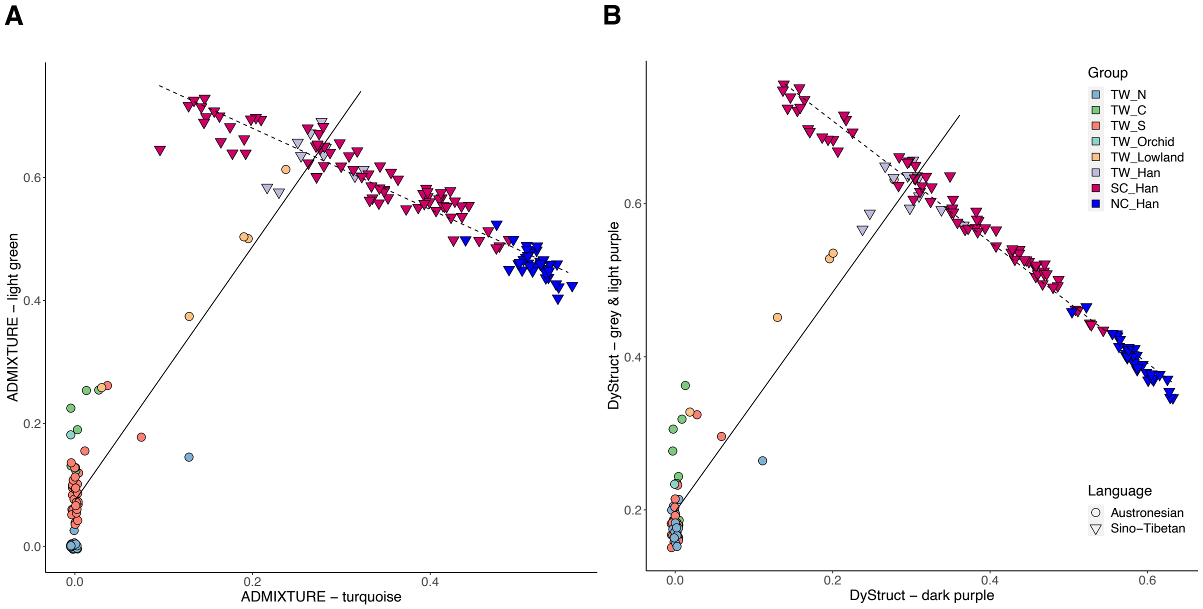

Fig. S4. ADMIXTURE and DyStruct Han-related components in the Taiwanese groups. We plotted the components that showed in some Taiwanese groups and were largely shared with the Han groups from China from the best-fitting K of (A) ADMIXTURE and (B) DyStruct results. Individuals were plotted as dots/triangles (according to language) and colored by groups. TW denotes Taiwan, and N, C, S denote northern, central, southern Highland groups, respectively. SC_Han/NC_Han denote the southern/northern Han groups from China. The light green ADMIXTURE (grey and light purple DyStruct) component is negatively correlated with the turquoise ADMIXTURE (dark purple DyStruct) component for the Chinese Han groups (r^2=0.921, p=0 for ADMIXTURE; r^2=0.983, p=0 for DyStruct), but they are positively correlated in the Taiwanese groups (r^2=0.913, p=0 for ADMIXTURE; r^2=0.926, p=0 for DyStruct).

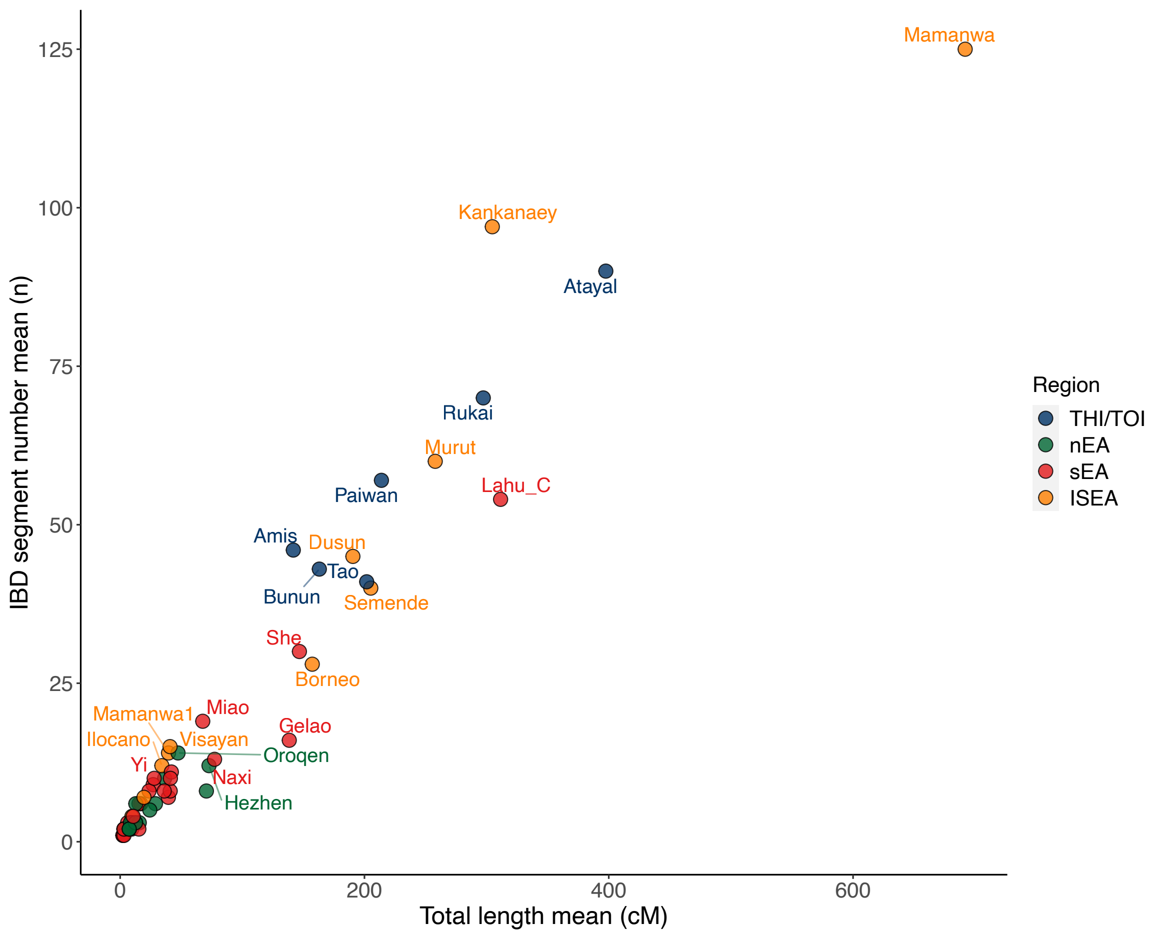

Fig. S5. Within group IBD sharing. We plotted on the x-axis the mean of the summed length of IBD segments, and on the y-axis the mean number of IBD segments for groups from nEA, sEA, and ISEA (colored accordingly). The THI/TOI groups are highlighted in another color.

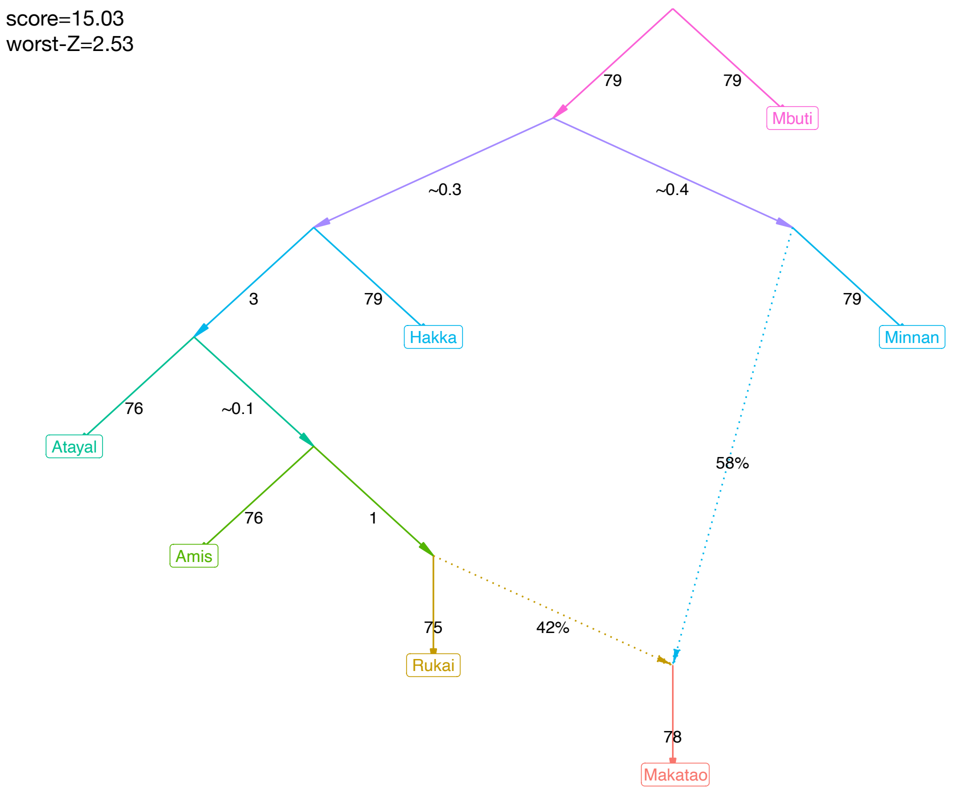

Fig. S6. Best-fitting admixture graph for investigating admixture in the Lowland group Makatao. The numbers on the solid branch lines are in genetic drift units while those on the dashed lines indicate ancestry proportions. The ADMIXTOOLS 2 graph score and the worst-fitting Z score are shown on the top-left.

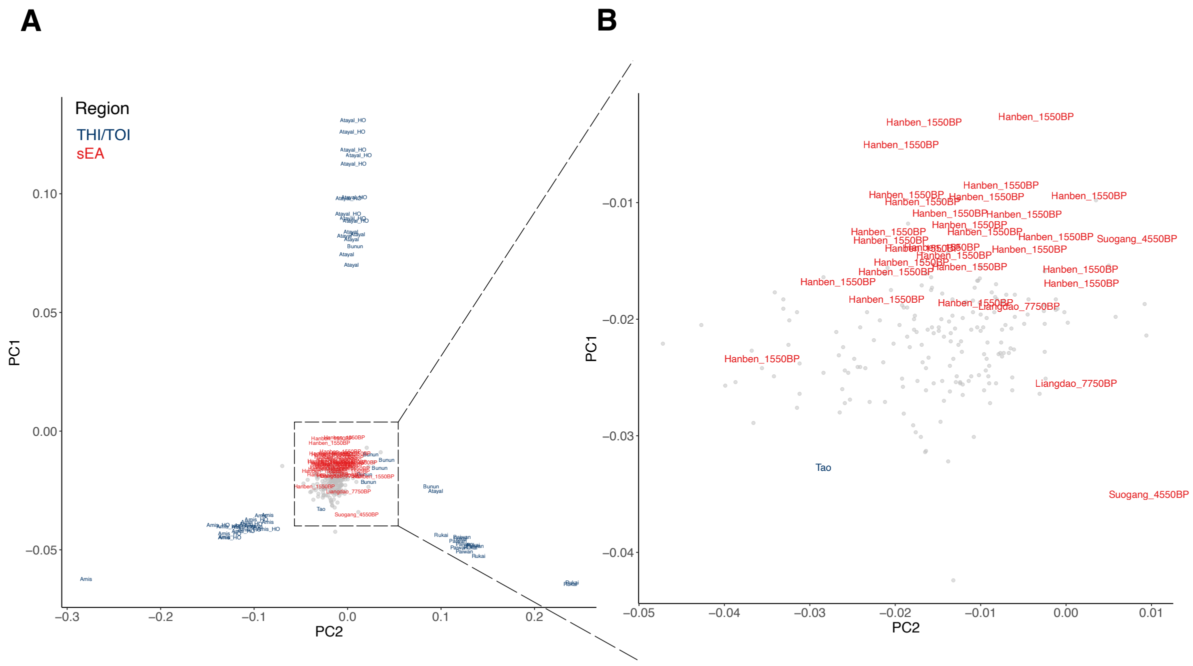

Fig. S7. THI/TOI PCA with a focus on the projected ancient Taiwan-related individuals. (A) the full results and (B) zoom-in of (A) on the projected ancient sEA individuals. Our focusing groups are colored by regions while the other projected ancient individuals are colored in grey to provide an overall background variation.

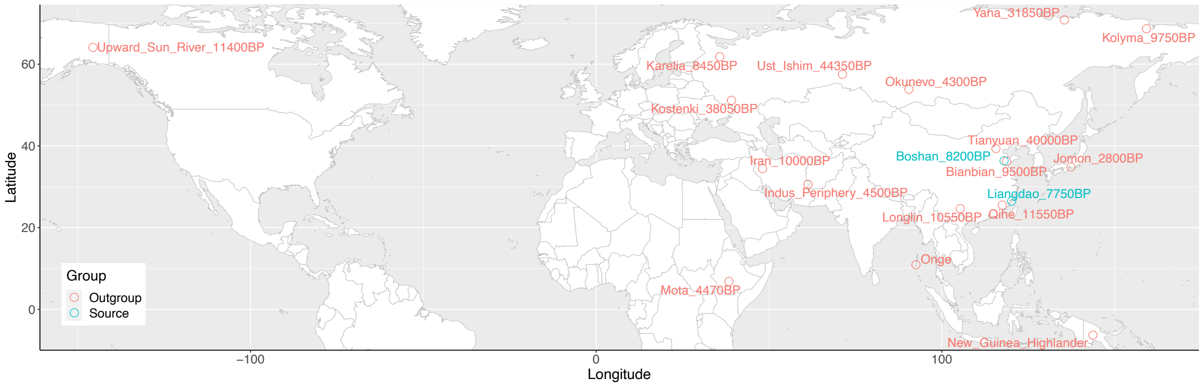

Fig. S8. Map of source groups and outgroups for qpAdm.

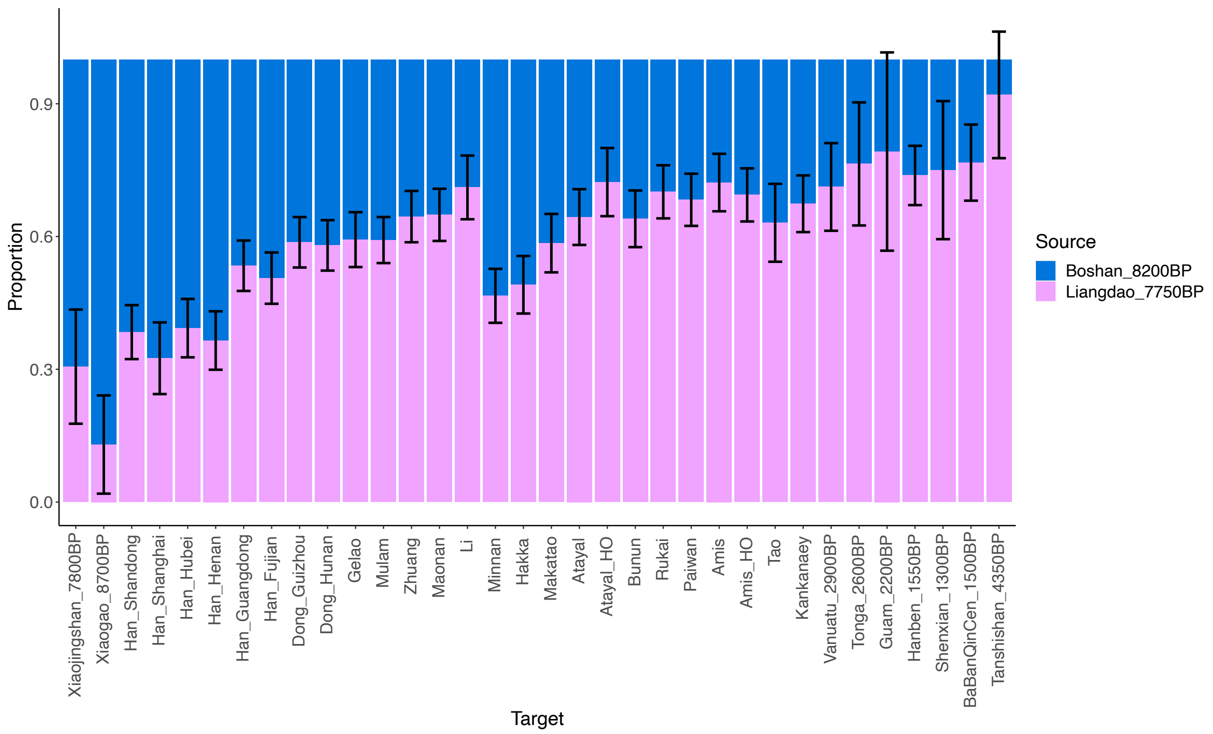

Fig. S9. Bar plot visualization of the qpAdm results. The error bars indicate the mean +/- one standard error.

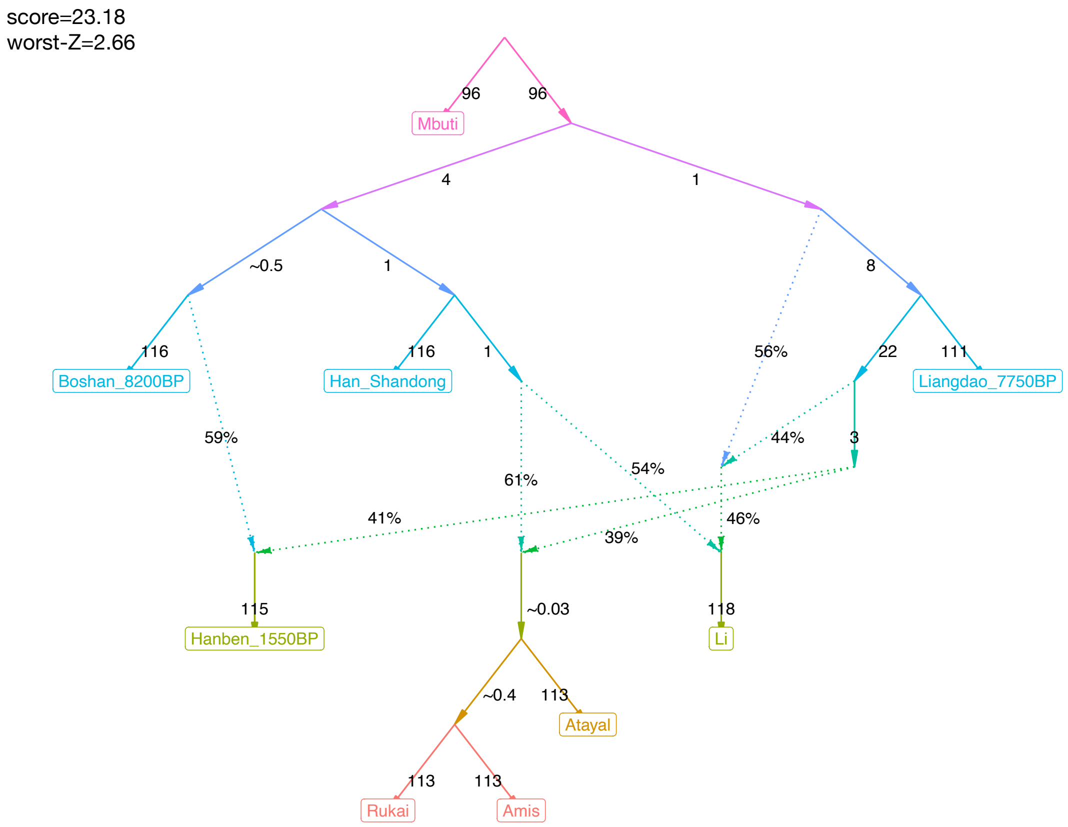

Fig. S10. Best-fitting admixture graph for the Into-Taiwan events. The numbers on the solid lines are in genetic drift units while those on the dashed lines indicate ancestry proportions. The ADMIXTOOLS 2 graph score and the worst-fitting Z score are shown on the top-left.

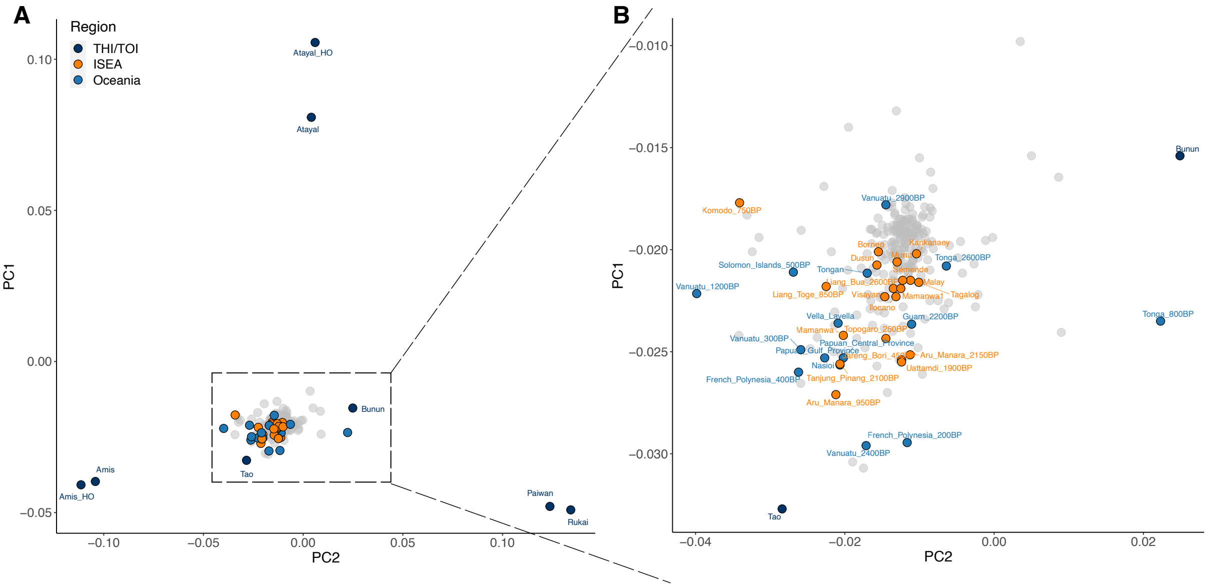

Fig. S11. THI/TOI PCA with a focus on the projected Austronesian-related ISEA/Oceanian groups. The PCA was computed using only the THI/TOI groups. (A) the full results and (B) zoom-in of (A) show the projected modern and ancient groups with a focus on the ISEA/Oceanian groups whose individuals have at least 10% Austronesian-related (purple) component in ADMIXTURE K=9 (Fig. 2; Fig. S3). Dots represent the median position of individuals from a group. Our focusing groups are colored by regions while the other projected groups are colored in grey to provide an overall background variation.

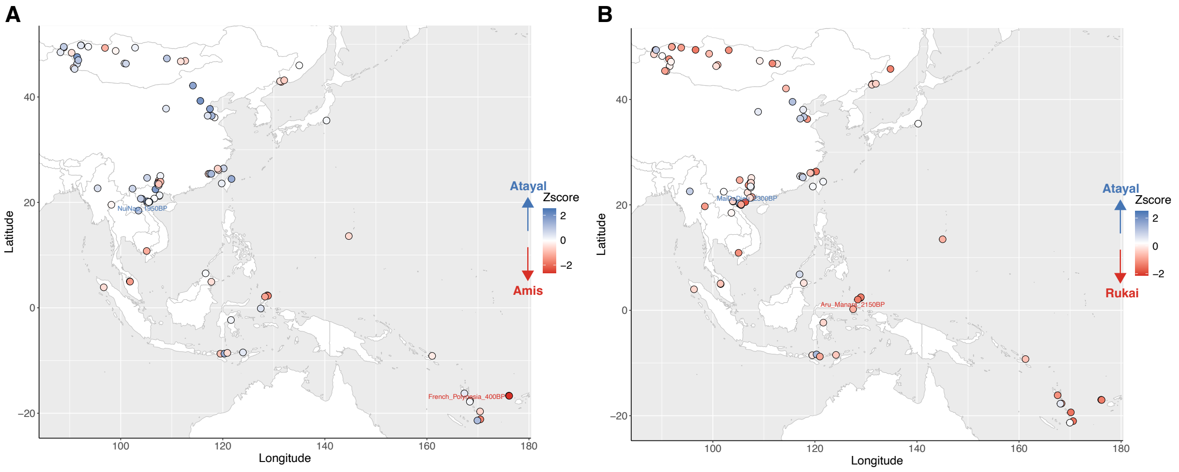

Fig. S12. Differential allelic sharing to the ancient groups from East Asia and Oceania between the Atayal and Rukai/Amis using only transversions and the French as an outgroup. Results of the form f4(Atayal, X; ancient groups from East Asia and Oceania, French) where X is Amis (A) / Rukai (B). The ancient groups are plotted as dots on the map, colored in proportion to Z score. Positive values (in blue) indicate more sharing with the Atayal while negative values (in red) indicate more sharing with the Amis/Rukai. Significant values (absolute Z score value >= 2) are further labelled with the population name.

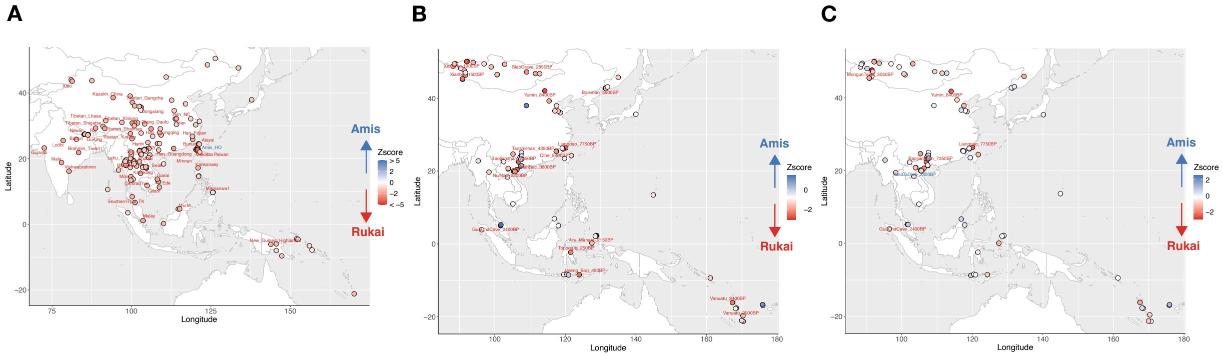

Fig. S13. Differential allelic sharing to the modern/ancient groups from East Asia and Oceania between the Atayal and Rukai/Amis. Results of the form f4(Amis, Rukai; Y, Mbuti) where Y are modern (A) / ancient (B) groups from East Asia and Oceania. Additional tests (C) are results of the form f4(Amis, Rukai; ancient groups, French) using only transversions, which reduces false positives caused by DNA damage and/or attraction to deep outgroups but also the statistical power due to the decreased number of SNPs. The modern/ancient groups are plotted as dots on the map, colored in proportion to Z score. Positive values (in blue) indicate more sharing with the Amis while negative values (in red) indicate more sharing with the Rukai. Significant values (absolute Z score value >= 2) are labelled with the population name.

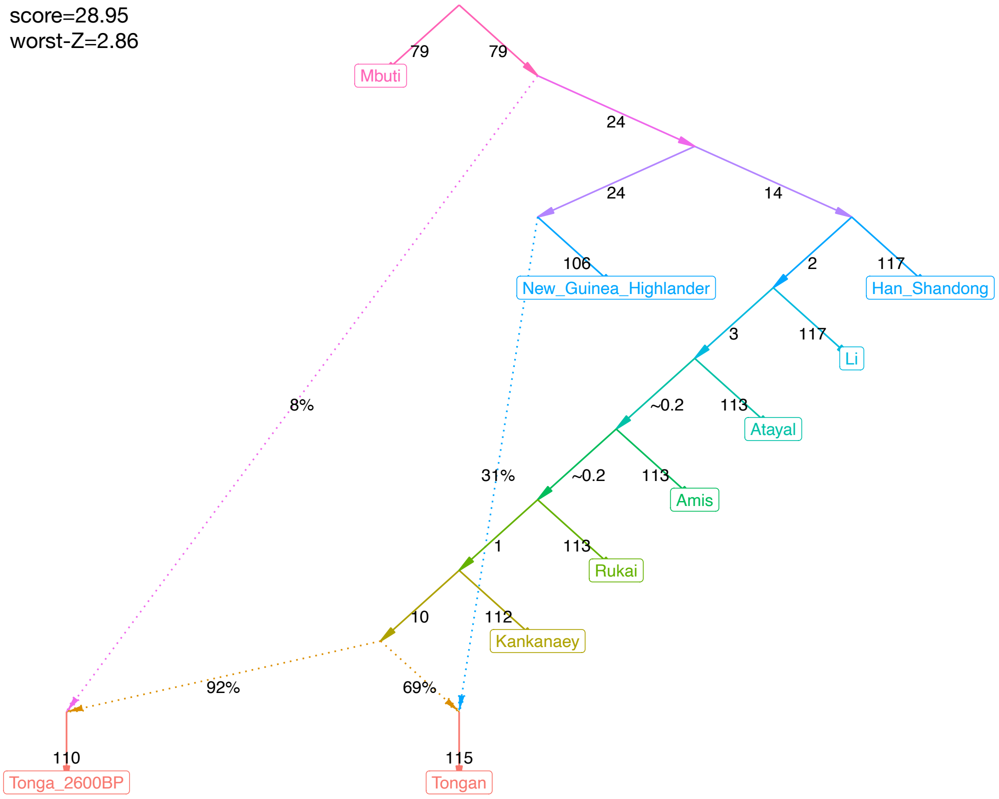

Fig. S14. Best-fitting admixture graph for the Out-of-Taiwan events. The numbers on the solid lines are in genetic drift units while those on the dashed lines indicate the ancestry proportions. The ADMIXTOOLS 2 graph score and the worst-fitting Z score are shown on the top-left.

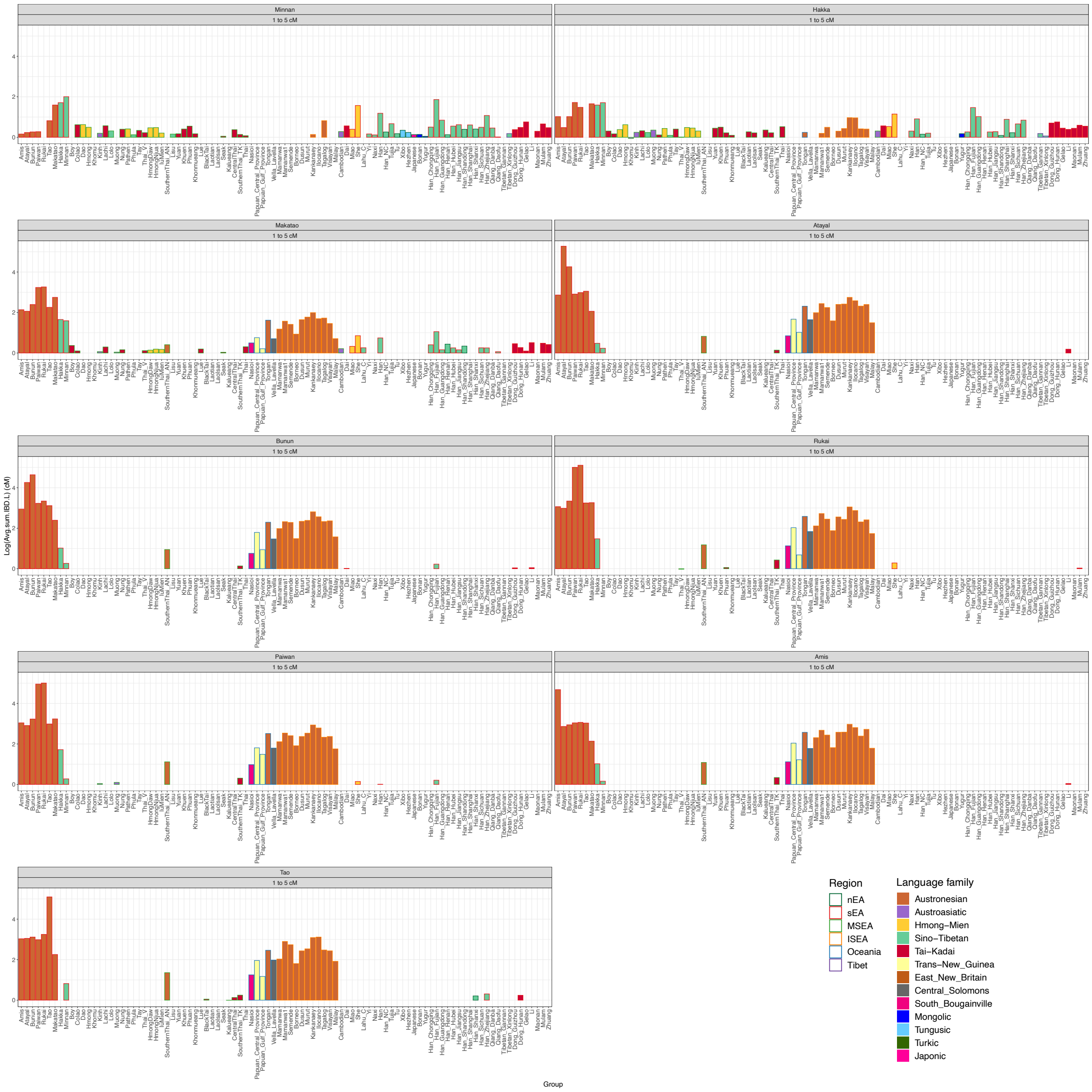

Fig. S15. Quantification of all IBD sharing between each Taiwanese group and East Asian (including other Taiwanese) and Oceanian groups in segment size range of 1 to 5 cM. The average summed IBD length between the Taiwanese groups (in rows) and other East Asian and Oceanian groups is depicted in the bar plots; filled bars are colored according to the languages spoken by the compared East Asian/Oceanian groups shown on the x-axis while the outline color of the empty bars indicates their regions.

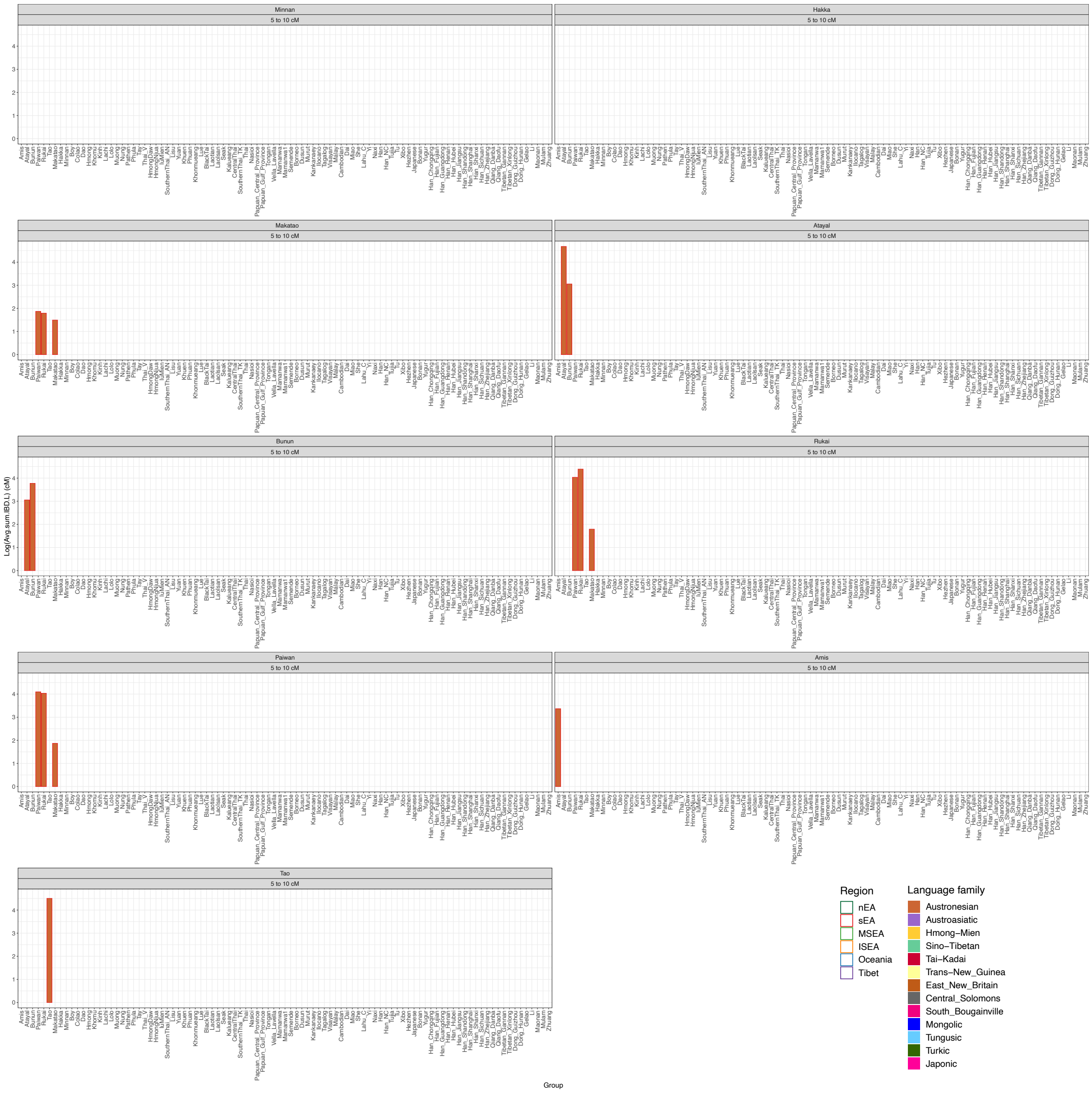

Fig. S16. Quantification of all IBD sharing between each Taiwanese group and East Asian (including other Taiwanese) and Oceanian groups in segment size range of 5 to 10 cM. The average summed IBD length between the Taiwanese groups (in rows) and other East Asian and Oceanian groups is depicted in the bar plots; filled bars are colored according to the languages spoken by the compared East Asian/Oceanian groups shown on the x-axis while the outline color of the empty bars indicates their regions.

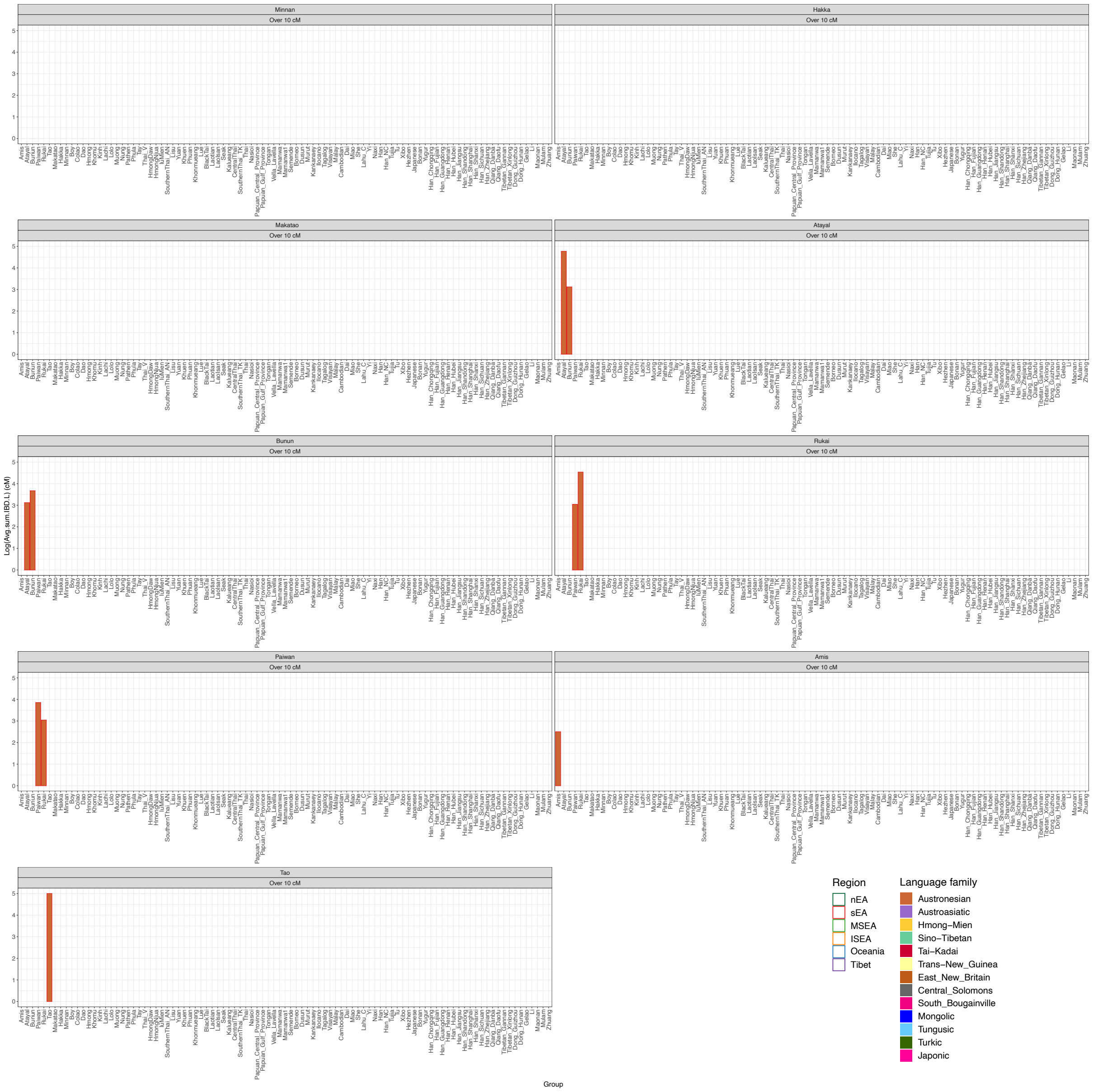

Fig. S17. Quantification of all IBD sharing between each Taiwanese group and East Asian (including other Taiwanese) and Oceanian groups in segment size range of over 10 cM. The average summed IBD length between the Taiwanese groups (in rows) and other East Asian and Oceanian groups is depicted in the bar plots; filled bars are colored according to the languages spoken by the compared East Asian/Oceanian groups shown on the x-axis while the outline color of the empty bars indicates their regions.

Supplementary Tables

Table.S1. Metadata of the individuals in this study. In the column “Region”, sEA is southern East Asia, nEA is northern East Asia, MSEA is Mainland Southeast Asia, and ISEA is Island Southeast Asia. The column “CP_Group” shows the group used for the grouping in the ChromoPainter analyses. In the column “QC”, imiss stands for missingness per individual, kin stands for 1st degree kinship, and ancient individuals only used in qpAdm as outgroups are noted.

Individual_ID	Population	Region	Country	Type	Age	Language	Latitude	Longitude	CP_Group	TW_Group	Reference	QC
TA199	Amis	sEA	Taiwan	modern	0	Austronesian	22.84	121.19	Amis	TW_S	This_study	PASS
TA29	Amis	sEA	Taiwan	modern	0	Austronesian	22.84	121.19	Amis	TW_S	This_study	PASS
TA245	Amis	sEA	Taiwan	modern	0	Austronesian	22.84	121.19	Amis	TW_S	This_study	PASS
TA81	Amis	sEA	Taiwan	modern	0	Austronesian	22.84	121.19	Amis	TW_S	This_study	PASS
TA258	Amis	sEA	Taiwan	modern	0	Austronesian	22.84	121.19	Amis	TW_S	This_study	PASS
TA95	Amis	sEA	Taiwan	modern	0	Austronesian	22.84	121.19	Amis	TW_S	This_study	PASS
TA101	Amis	sEA	Taiwan	modern	0	Austronesian	22.84	121.19	Amis	TW_S	This_study	PASS
TA131	Amis	sEA	Taiwan	modern	0	Austronesian	22.84	121.19	Amis	TW_S	This_study	PASS
TA138	Amis	sEA	Taiwan	modern	0	Austronesian	22.84	121.19	Amis	TW_S	This_study	PASS
ata34	Atayal	sEA	Taiwan	modern	0	Austronesian	24.61	121.3	Atayal	TW_N	This_study	PASS
ata46	Atayal	sEA	Taiwan	modern	0	Austronesian	24.61	121.3	Atayal	TW_N	This_study	PASS
ata30	Atayal	sEA	Taiwan	modern	0	Austronesian	24.61	121.3	Atayal	TW_N	This_study	PASS
ata141	Atayal	sEA	Taiwan	modern	0	Austronesian	24.61	121.3	Atayal	TW_N	This_study	PASS
ata10	Atayal	sEA	Taiwan	modern	0	Austronesian	24.61	121.3	Atayal	TW_N	This_study	PASS
ata4	Atayal	sEA	Taiwan	modern	0	Austronesian	24.61	121.3	Atayal	TW_N	This_study	PASS
ata1	Atayal	sEA	Taiwan	modern	0	Austronesian	24.61	121.3	Atayal	TW_N	This_study	PASS
bun55	Bunun	sEA	Taiwan	modern	0	Austronesian	23.21	120.7	Bunun	TW_C	This_study	PASS
bun34	Bunun	sEA	Taiwan	modern	0	Austronesian	23.21	120.7	Bunun	TW_C	This_study	PASS
bun32	Bunun	sEA	Taiwan	modern	0	Austronesian	23.21	120.7	Bunun	TW_C	This_study	PASS
bun38	Bunun	sEA	Taiwan	modern	0	Austronesian	23.21	120.7	Bunun	TW_C	This_study	PASS
bun30	Bunun	sEA	Taiwan	modern	0	Austronesian	23.21	120.7	Bunun	TW_C	This_study	PASS
bun39	Bunun	sEA	Taiwan	modern	0	Austronesian	23.21	120.7	Bunun	TW_C	This_study	PASS
bun48	Bunun	sEA	Taiwan	modern	0	Austronesian	23.21	120.7	Bunun	TW_C	This_study	PASS
pai10	Paiwan	sEA	Taiwan	modern	0	Austronesian	22.68	120.69	Paiwan	TW_S	This_study	PASS
pai3	Paiwan	sEA	Taiwan	modern	0	Austronesian	22.68	120.69	Paiwan	TW_S	This_study	PASS
pai58	Paiwan	sEA	Taiwan	modern	0	Austronesian	22.68	120.69	Paiwan	TW_S	This_study	PASS
pai20	Paiwan	sEA	Taiwan	modern	0	Austronesian	22.68	120.69	Paiwan	TW_S	This_study	PASS
pai103	Paiwan	sEA	Taiwan	modern	0	Austronesian	22.68	120.69	Paiwan	TW_S	This_study	PASS
pai55	Paiwan	sEA	Taiwan	modern	0	Austronesian	22.68	120.69	Paiwan	TW_S	This_study	PASS
pai25	Paiwan	sEA	Taiwan	modern	0	Austronesian	22.68	120.69	Paiwan	TW_S	This_study	imiss
ruk487	Rukai	sEA	Taiwan	modern	0	Austronesian	22.7	120.61	Rukai	TW_S	This_study	PASS
ruk540	Rukai	sEA	Taiwan	modern	0	Austronesian	22.7	120.61	Rukai	TW_S	This_study	PASS
ruk111	Rukai	sEA	Taiwan	modern	0	Austronesian	22.7	120.61	Rukai	TW_S	This_study	PASS
ruk502	Rukai	sEA	Taiwan	modern	0	Austronesian	22.7	120.61	Rukai	TW_S	This_study	PASS
ruk538	Rukai	sEA	Taiwan	modern	0	Austronesian	22.7	120.61	Rukai	TW_S	This_study	PASS
ruk499	Rukai	sEA	Taiwan	modern	0	Austronesian	22.7	120.61	Rukai	TW_S	This_study	PASS
ruk535	Rukai	sEA	Taiwan	modern	0	Austronesian	22.7	120.61	Rukai	TW_S	This_study	PASS
tao45	Tao	sEA	Taiwan	modern	0	Austronesian	22.03	121.55	Tao	TW_Orchid	This_study	PASS

pin33	Makatao	sEA	Taiwan	modern	0	Austronesian	22.6	120.61	Makatao	TW_Lowland	This_study	PASS
pin29	Makatao	sEA	Taiwan	modern	0	Austronesian	22.6	120.61	Makatao	TW_Lowland	This_study	PASS
pin74	Makatao	sEA	Taiwan	modern	0	Austronesian	22.6	120.61	Makatao	TW_Lowland	This_study	PASS
pin126	Makatao	sEA	Taiwan	modern	0	Austronesian	22.6	120.61	Makatao	TW_Lowland	This_study	PASS
pin81	Makatao	sEA	Taiwan	modern	0	Austronesian	22.6	120.61	Makatao	TW_Lowland	This_study	PASS
hak95	Hakka	sEA	Taiwan	modern	0	Sino-Tibetan	22.67	120.6	Hakka	TW_Han	This_study	PASS
hak19	Hakka	sEA	Taiwan	modern	0	Sino-Tibetan	22.67	120.6	Hakka	TW_Han	This_study	PASS
hak13	Hakka	sEA	Taiwan	modern	0	Sino-Tibetan	22.67	120.6	Hakka	TW_Han	This_study	PASS
hak23	Hakka	sEA	Taiwan	modern	0	Sino-Tibetan	22.67	120.6	Hakka	TW_Han	This_study	PASS
hak33	Hakka	sEA	Taiwan	modern	0	Sino-Tibetan	22.67	120.6	Hakka	TW_Han	This_study	PASS
hak36	Hakka	sEA	Taiwan	modern	0	Sino-Tibetan	22.67	120.6	Hakka	TW_Han	This_study	PASS
min186	Minnan	sEA	Taiwan	modern	0	Sino-Tibetan	22.63	120.31	Minnan	TW_Han	This_study	PASS
min235	Minnan	sEA	Taiwan	modern	0	Sino-Tibetan	22.63	120.31	Minnan	TW_Han	This_study	PASS
min396	Minnan	sEA	Taiwan	modern	0	Sino-Tibetan	22.63	120.31	Minnan	TW_Han	This_study	PASS
min305	Minnan	sEA	Taiwan	modern	0	Sino-Tibetan	22.63	120.31	Minnan	TW_Han	This_study	PASS
min321	Minnan	sEA	Taiwan	modern	0	Sino-Tibetan	22.63	120.31	Minnan	TW_Han	This_study	PASS
min76	Minnan	sEA	Taiwan	modern	0	Sino-Tibetan	22.63	120.31	Minnan	TW_Han	This_study	PASS
BoY180	Boy	MSEA	Vietnam	modern	0	Tai-Kadai	22.7	104.1	Boy	N.A.	Liu_et_al_2020	PASS
BoY181	Boy	MSEA	Vietnam	modern	0	Tai-Kadai	22.7	104.1	Boy	N.A.	Liu_et_al_2020	kin
BoY182	Boy	MSEA	Vietnam	modern	0	Tai-Kadai	22.7	104.1	Boy	N.A.	Liu_et_al_2020	PASS
BoY184	Boy	MSEA	Vietnam	modern	0	Tai-Kadai	22.7	104.1	Boy	N.A.	Liu_et_al_2020	PASS
BoY185	Boy	MSEA	Vietnam	modern	0	Tai-Kadai	22.7	104.1	Boy	N.A.	Liu_et_al_2020	PASS
BoY186	Boy	MSEA	Vietnam	modern	0	Tai-Kadai	22.7	104.1	Boy	N.A.	Liu_et_al_2020	kin
BoY188	Boy	MSEA	Vietnam	modern	0	Tai-Kadai	22.7	104.1	Boy	N.A.	Liu_et_al_2020	kin
BoY189	Boy	MSEA	Vietnam	modern	0	Tai-Kadai	22.7	104.1	Boy	N.A.	Liu_et_al_2020	PASS
BoY190	Boy	MSEA	Vietnam	modern	0	Tai-Kadai	22.7	104.1	Boy	N.A.	Liu_et_al_2020	PASS
BoY191	Boy	MSEA	Vietnam	modern	0	Tai-Kadai	22.7	104.1	Boy	N.A.	Liu_et_al_2020	kin
BoY192_2	Boy	MSEA	Vietnam	modern	0	Tai-Kadai	22.7	104.1	Boy	N.A.	Liu_et_al_2020	kin
BoY193_2	Boy	MSEA	Vietnam	modern	0	Tai-Kadai	22.7	104.1	Boy	N.A.	Liu_et_al_2020	kin
Cham58	Cham	MSEA	Vietnam	modern	0	Austronesian	11.5	108.9	Cham	N.A.	Liu_et_al_2020	PASS
Cham64	Cham	MSEA	Vietnam	modern	0	Austronesian	11.5	108.9	Cham	N.A.	Liu_et_al_2020	PASS
Cham66	Cham	MSEA	Vietnam	modern	0	Austronesian	11.5	108.9	Cham	N.A.	Liu_et_al_2020	PASS
Cham75	Cham	MSEA	Vietnam	modern	0	Austronesian	11.5	108.9	Cham	N.A.	Liu_et_al_2020	PASS
Cham80	Cham	MSEA	Vietnam	modern	0	Austronesian	11.5	108.9	Cham	N.A.	Liu_et_al_2020	PASS
Cham83	Cham	MSEA	Vietnam	modern	0	Austronesian	11.5	108.9	Cham	N.A.	Liu_et_al_2020	PASS
Cham89	Cham	MSEA	Vietnam	modern	0	Austronesian	11.5	108.9	Cham	N.A.	Liu_et_al_2020	PASS
Cham127	Cham	MSEA	Vietnam	modern	0	Austronesian	11.5	108.9	Cham	N.A.	Liu_et_al_2020	PASS
Cham133	Cham	MSEA	Vietnam	modern	0	Austronesian	11.5	108.9	Cham	N.A.	Liu_et_al_2020	PASS
Cham137	Cham	MSEA	Vietnam	modern	0	Austronesian	11.5	108.9	Cham	N.A.	Liu_et_al_2020	PASS
CoLao566	Colao	MSEA	Vietnam	modern	0	Tai-Kadai	22.7	104.7	Colao	N.A.	Liu_et_al_2020	PASS
CoLao567	Colao	MSEA	Vietnam	modern	0	Tai-Kadai	22.7	104.7	Colao	N.A.	Liu_et_al_2020	PASS
CoLao568	Colao	MSEA	Vietnam	modern	0	Tai-Kadai	22.7	104.7	Colao	N.A.	Liu_et_al_2020	PASS
CoLao569	Colao	MSEA	Vietnam	modern	0	Tai-Kadai	22.7	104.7	Colao	N.A.	Liu_et_al_2020	PASS
CoLao570	Colao	MSEA	Vietnam	modern	0	Tai-Kadai	22.7	104.7	Colao	N.A.	Liu_et_al_2020	PASS

CoLao571	Colao	MSEA	Vietnam	modern	0	Tai-Kadai	22.7	104.7	Colao	N.A.	Liu_et_al_2020	PASS
CoLao572	Colao	MSEA	Vietnam	modern	0	Tai-Kadai	22.7	104.7	Colao	N.A.	Liu_et_al_2020	PASS
CoLao573	Colao	MSEA	Vietnam	modern	0	Tai-Kadai	22.7	104.7	Colao	N.A.	Liu_et_al_2020	PASS
CoLao574	Colao	MSEA	Vietnam	modern	0	Tai-Kadai	22.7	104.7	Colao	N.A.	Liu_et_al_2020	PASS
CoLao575	Colao	MSEA	Vietnam	modern	0	Tai-Kadai	22.7	104.7	Colao	N.A.	Liu_et_al_2020	PASS
CoLao576	Colao	MSEA	Vietnam	modern	0	Tai-Kadai	22.7	104.7	Colao	N.A.	Liu_et_al_2020	PASS
CoLao577	Colao	MSEA	Vietnam	modern	0	Tai-Kadai	22.7	104.7	Colao	N.A.	Liu_et_al_2020	PASS
Cong410	Cong	MSEA	Vietnam	modern	0	Sino-Tibetan	22.4	102.7	Cong	N.A.	Liu_et_al_2020	PASS
Cong411	Cong	MSEA	Vietnam	modern	0	Sino-Tibetan	22.4	102.7	Cong	N.A.	Liu_et_al_2020	PASS
Cong412	Cong	MSEA	Vietnam	modern	0	Sino-Tibetan	22.4	102.7	Cong	N.A.	Liu_et_al_2020	kin
Cong413	Cong	MSEA	Vietnam	modern	0	Sino-Tibetan	22.4	102.7	Cong	N.A.	Liu_et_al_2020	PASS
Cong414	Cong	MSEA	Vietnam	modern	0	Sino-Tibetan	22.4	102.7	Cong	N.A.	Liu_et_al_2020	PASS
Cong415	Cong	MSEA	Vietnam	modern	0	Sino-Tibetan	22.4	102.7	Cong	N.A.	Liu_et_al_2020	PASS
Cong416	Cong	MSEA	Vietnam	modern	0	Sino-Tibetan	22.4	102.7	Cong	N.A.	Liu_et_al_2020	PASS
Cong420	Cong	MSEA	Vietnam	modern	0	Sino-Tibetan	22.4	102.7	Cong	N.A.	Liu_et_al_2020	PASS
Cong421	Cong	MSEA	Vietnam	modern	0	Sino-Tibetan	22.4	102.7	Cong	N.A.	Liu_et_al_2020	PASS
Cong423	Cong	MSEA	Vietnam	modern	0	Sino-Tibetan	22.4	102.7	Cong	N.A.	Liu_et_al_2020	PASS
Cong424	Cong	MSEA	Vietnam	modern	0	Sino-Tibetan	22.4	102.7	Cong	N.A.	Liu_et_al_2020	PASS
Cong425	Cong	MSEA	Vietnam	modern	0	Sino-Tibetan	22.4	102.7	Cong	N.A.	Liu_et_al_2020	PASS
Dao50	Dao	MSEA	Vietnam	modern	0	Hmong-Mien	22.7	104.7	N.A.	N.A.	Liu_et_al_2020	PASS
Dao51	Dao	MSEA	Vietnam	modern	0	Hmong-Mien	22.7	104.7	N.A.	N.A.	Liu_et_al_2020	PASS
Dao52	Dao	MSEA	Vietnam	modern	0	Hmong-Mien	22.7	104.7	N.A.	N.A.	Liu_et_al_2020	PASS
Dao623	Dao	MSEA	Vietnam	modern	0	Hmong-Mien	22.7	104.7	N.A.	N.A.	Liu_et_al_2020	PASS
Dao624_2	Dao	MSEA	Vietnam	modern	0	Hmong-Mien	22.7	104.7	N.A.	N.A.	Liu_et_al_2020	PASS
Dao625	Dao	MSEA	Vietnam	modern	0	Hmong-Mien	22.7	104.7	N.A.	N.A.	Liu_et_al_2020	PASS
Dao626	Dao	MSEA	Vietnam	modern	0	Hmong-Mien	22.7	104.7	N.A.	N.A.	Liu_et_al_2020	PASS
Dao627	Dao	MSEA	Vietnam	modern	0	Hmong-Mien	22.7	104.7	N.A.	N.A.	Liu_et_al_2020	PASS
Dao629	Dao	MSEA	Vietnam	modern	0	Hmong-Mien	22.7	104.7	N.A.	N.A.	Liu_et_al_2020	PASS
Dao630	Dao	MSEA	Vietnam	modern	0	Hmong-Mien	22.7	104.7	N.A.	N.A.	Liu_et_al_2020	PASS
Dao631	Dao	MSEA	Vietnam	modern	0	Hmong-Mien	22.7	104.7	N.A.	N.A.	Liu_et_al_2020	PASS
Dao632	Dao	MSEA	Vietnam	modern	0	Hmong-Mien	22.7	104.7	N.A.	N.A.	Liu_et_al_2020	PASS
Ede61	Ede	MSEA	Vietnam	modern	0	Austronesian	12.9	108.1	Ede	N.A.	Liu_et_al_2020	PASS
Ede74	Ede	MSEA	Vietnam	modern	0	Austronesian	12.9	108.2	Ede	N.A.	Liu_et_al_2020	PASS
Ede95	Ede	MSEA	Vietnam	modern	0	Austronesian	12.7	108.8	Ede	N.A.	Liu_et_al_2020	PASS
Ede97	Ede	MSEA	Vietnam	modern	0	Austronesian	12.7	108	Ede	N.A.	Liu_et_al_2020	PASS
Ede102	Ede	MSEA	Vietnam	modern	0	Austronesian	13	108.4	Ede	N.A.	Liu_et_al_2020	PASS
Ede112	Ede	MSEA	Vietnam	modern	0	Austronesian	12.9	108.9	Ede	N.A.	Liu_et_al_2020	PASS
Ede123	Ede	MSEA	Vietnam	modern	0	Austronesian	12.9	108.2	Ede	N.A.	Liu_et_al_2020	PASS
Ede736	Ede	MSEA	Vietnam	modern	0	Austronesian	12.8	108.6	Ede	N.A.	Liu_et_al_2020	PASS
Ede737	Ede	MSEA	Vietnam	modern	0	Austronesian	13	108.4	Ede	N.A.	Liu_et_al_2020	PASS
Ede738	Ede	MSEA	Vietnam	modern	0	Austronesian	12.7	108.8	Ede	N.A.	Liu_et_al_2020	PASS
Ede740	Ede	MSEA	Vietnam	modern	0	Austronesian	12.7	108.8	Ede	N.A.	Liu_et_al_2020	PASS
Giarai60	Giarai	MSEA	Vietnam	modern	0	Austronesian	13.2	108.7	Giarai	N.A.	Liu_et_al_2020	PASS
Giarai67	Giarai	MSEA	Vietnam	modern	0	Austronesian	13.3	108.4	Giarai	N.A.	Liu_et_al_2020	PASS

Giarai91	Giarai	MSEA	Vietnam	modern	0	Austronesian	13.6	108.1	Giarai	N.A.	Liu_et_al_2020	PASS
Giarai100	Giarai	MSEA	Vietnam	modern	0	Austronesian	13.2	108.7	Giarai	N.A.	Liu_et_al_2020	PASS
Giarai107	Giarai	MSEA	Vietnam	modern	0	Austronesian	13.3	108.4	Giarai	N.A.	Liu_et_al_2020	PASS
Giarai124	Giarai	MSEA	Vietnam	modern	0	Austronesian	13.6	107.8	Giarai	N.A.	Liu_et_al_2020	PASS
Giarai134	Giarai	MSEA	Vietnam	modern	0	Austronesian	14.1	107.6	Giarai	N.A.	Liu_et_al_2020	PASS
Giarai716	Giarai	MSEA	Vietnam	modern	0	Austronesian	13.8	107.7	Giarai	N.A.	Liu_et_al_2020	PASS
Giarai718	Giarai	MSEA	Vietnam	modern	0	Austronesian	14.2	108	Giarai	N.A.	Liu_et_al_2020	PASS
Giarai719	Giarai	MSEA	Vietnam	modern	0	Austronesian	14	108	Giarai	N.A.	Liu_et_al_2020	PASS
Giarai722	Giarai	MSEA	Vietnam	modern	0	Austronesian	13.3	108.4	Giarai	N.A.	Liu_et_al_2020	PASS
Giarai723	Giarai	MSEA	Vietnam	modern	0	Austronesian	13.6	107.8	Giarai	N.A.	Liu_et_al_2020	PASS
HaNhi44	Hanhi	MSEA	Vietnam	modern	0	Sino-Tibetan	22.4	102.7	Hanhi	N.A.	Liu_et_al_2020	PASS
HaNhi45	Hanhi	MSEA	Vietnam	modern	0	Sino-Tibetan	22.4	102.7	Hanhi	N.A.	Liu_et_al_2020	PASS
HaNhi46	Hanhi	MSEA	Vietnam	modern	0	Sino-Tibetan	22.4	102.7	Hanhi	N.A.	Liu_et_al_2020	PASS
HaNhi315	Hanhi	MSEA	Vietnam	modern	0	Sino-Tibetan	22.4	102.7	Hanhi	N.A.	Liu_et_al_2020	PASS
HaNhi317	Hanhi	MSEA	Vietnam	modern	0	Sino-Tibetan	22.4	102.7	Hanhi	N.A.	Liu_et_al_2020	PASS
HaNhi319	Hanhi	MSEA	Vietnam	modern	0	Sino-Tibetan	22.4	102.7	Hanhi	N.A.	Liu_et_al_2020	PASS
HaNhi320	Hanhi	MSEA	Vietnam	modern	0	Sino-Tibetan	22.4	102.7	Hanhi	N.A.	Liu_et_al_2020	PASS
HaNhi321	Hanhi	MSEA	Vietnam	modern	0	Sino-Tibetan	22.4	102.7	Hanhi	N.A.	Liu_et_al_2020	PASS
HaNhi322	Hanhi	MSEA	Vietnam	modern	0	Sino-Tibetan	22.4	102.7	Hanhi	N.A.	Liu_et_al_2020	kin
HaNhi323_2	Hanhi	MSEA	Vietnam	modern	0	Sino-Tibetan	22.4	102.7	Hanhi	N.A.	Liu_et_al_2020	PASS
HaNhi328	Hanhi	MSEA	Vietnam	modern	0	Sino-Tibetan	22.4	102.7	Hanhi	N.A.	Liu_et_al_2020	PASS
HaNhi329	Hanhi	MSEA	Vietnam	modern	0	Sino-Tibetan	22.4	102.7	Hanhi	N.A.	Liu_et_al_2020	PASS
Hmong194	Hmong	MSEA	Vietnam	modern	0	Hmong-Mien	21.4	103	N.A.	N.A.	Liu_et_al_2020	PASS
Hmong195	Hmong	MSEA	Vietnam	modern	0	Hmong-Mien	21.4	103	N.A.	N.A.	Liu_et_al_2020	PASS
Hmong196_2	Hmong	MSEA	Vietnam	modern	0	Hmong-Mien	21.4	103	N.A.	N.A.	Liu_et_al_2020	kin
Hmong197_1	Hmong	MSEA	Vietnam	modern	0	Hmong-Mien	21.4	103	N.A.	N.A.	Liu_et_al_2020	kin
Hmong198	Hmong	MSEA	Vietnam	modern	0	Hmong-Mien	21.4	103	N.A.	N.A.	Liu_et_al_2020	PASS
Hmong199	Hmong	MSEA	Vietnam	modern	0	Hmong-Mien	21.4	103	N.A.	N.A.	Liu_et_al_2020	PASS
Hmong200	Hmong	MSEA	Vietnam	modern	0	Hmong-Mien	21.4	103	N.A.	N.A.	Liu_et_al_2020	PASS
Hmong201	Hmong	MSEA	Vietnam	modern	0	Hmong-Mien	21.4	103	N.A.	N.A.	Liu_et_al_2020	PASS
Hmong202	Hmong	MSEA	Vietnam	modern	0	Hmong-Mien	21.4	103	N.A.	N.A.	Liu_et_al_2020	kin
Hmong203	Hmong	MSEA	Vietnam	modern	0	Hmong-Mien	21.4	103	N.A.	N.A.	Liu_et_al_2020	PASS
Hmong204	Hmong	MSEA	Vietnam	modern	0	Hmong-Mien	21.4	103	N.A.	N.A.	Liu_et_al_2020	PASS
Hmong205	Hmong	MSEA	Vietnam	modern	0	Hmong-Mien	21.4	103	N.A.	N.A.	Liu_et_al_2020	PASS
KhoMu235	Khomu	MSEA	Vietnam	modern	0	Austroasiatic	21.4	103	N.A.	N.A.	Liu_et_al_2020	PASS
KhoMu236	Khomu	MSEA	Vietnam	modern	0	Austroasiatic	21.4	103	N.A.	N.A.	Liu_et_al_2020	PASS
KhoMu237	Khomu	MSEA	Vietnam	modern	0	Austroasiatic	21.4	103	N.A.	N.A.	Liu_et_al_2020	PASS
KhoMu238	Khomu	MSEA	Vietnam	modern	0	Austroasiatic	21.4	103	N.A.	N.A.	Liu_et_al_2020	PASS
KhoMu239	Khomu	MSEA	Vietnam	modern	0	Austroasiatic	21.4	103	N.A.	N.A.	Liu_et_al_2020	PASS
KhoMu240	Khomu	MSEA	Vietnam	modern	0	Austroasiatic	21.4	103	N.A.	N.A.	Liu_et_al_2020	PASS
KhoMu241	Khomu	MSEA	Vietnam	modern	0	Austroasiatic	21.4	103	N.A.	N.A.	Liu_et_al_2020	kin
KhoMu242	Khomu	MSEA	Vietnam	modern	0	Austroasiatic	21.4	103	N.A.	N.A.	Liu_et_al_2020	PASS
KhoMu244	Khomu	MSEA	Vietnam	modern	0	Austroasiatic	21.4	103	N.A.	N.A.	Liu_et_al_2020	kin
KhoMu245_2	Khomu	MSEA	Vietnam	modern	0	Austroasiatic	21.4	103	N.A.	N.A.	Liu_et_al_2020	PASS

KhoMu246	Khomu	MSEA	Vietnam	modern	0	Austroasiatic	21.4	103	N.A.	N.A.	Liu_et_al_2020	kin
KhoMu247	Khomu	MSEA	Vietnam	modern	0	Austroasiatic	21.4	103	N.A.	N.A.	Liu_et_al_2020	PASS
Kinh01_1	Kinh	MSEA	Vietnam	modern	0	Austroasiatic	21.4	103	N.A.	N.A.	Liu_et_al_2020	PASS
Kinh03	Kinh	MSEA	Vietnam	modern	0	Austroasiatic	21.2	106	N.A.	N.A.	Liu_et_al_2020	PASS
Kinh05	Kinh	MSEA	Vietnam	modern	0	Austroasiatic	21	105.9	N.A.	N.A.	Liu_et_al_2020	PASS
Kinh06	Kinh	MSEA	Vietnam	modern	0	Austroasiatic	21	105.9	N.A.	N.A.	Liu_et_al_2020	PASS
Kinh07	Kinh	MSEA	Vietnam	modern	0	Austroasiatic	21	105.9	N.A.	N.A.	Liu_et_al_2020	PASS
Kinh08	Kinh	MSEA	Vietnam	modern	0	Austroasiatic	21	105.9	N.A.	N.A.	Liu_et_al_2020	PASS
Kinh09	Kinh	MSEA	Vietnam	modern	0	Austroasiatic	21	105.9	N.A.	N.A.	Liu_et_al_2020	PASS
Kinh10	Kinh	MSEA	Vietnam	modern	0	Austroasiatic	21	105.9	N.A.	N.A.	Liu_et_al_2020	PASS
Kinh11_1	Kinh	MSEA	Vietnam	modern	0	Austroasiatic	21	105.9	N.A.	N.A.	Liu_et_al_2020	PASS
Kinh12	Kinh	MSEA	Vietnam	modern	0	Austroasiatic	21	105.9	N.A.	N.A.	Liu_et_al_2020	PASS
Kinh14	Kinh	MSEA	Vietnam	modern	0	Austroasiatic	21	105.9	N.A.	N.A.	Liu_et_al_2020	PASS
Kinh15	Kinh	MSEA	Vietnam	modern	0	Austroasiatic	22	106.9	N.A.	N.A.	Liu_et_al_2020	PASS
LaChi530	Lachi	MSEA	Vietnam	modern	0	Tai-Kadai	22.7	104.7	Lachi	N.A.	Liu_et_al_2020	PASS
LaChi532	Lachi	MSEA	Vietnam	modern	0	Tai-Kadai	22.7	104.7	Lachi	N.A.	Liu_et_al_2020	PASS
LaChi533	Lachi	MSEA	Vietnam	modern	0	Tai-Kadai	22.7	104.7	Lachi	N.A.	Liu_et_al_2020	PASS
LaChi534	Lachi	MSEA	Vietnam	modern	0	Tai-Kadai	22.7	104.7	Lachi	N.A.	Liu_et_al_2020	PASS
LaChi535	Lachi	MSEA	Vietnam	modern	0	Tai-Kadai	22.7	104.7	Lachi	N.A.	Liu_et_al_2020	PASS
LaChi554	Lachi	MSEA	Vietnam	modern	0	Tai-Kadai	22.7	104.7	Lachi	N.A.	Liu_et_al_2020	PASS
LaChi555	Lachi	MSEA	Vietnam	modern	0	Tai-Kadai	22.7	104.7	Lachi	N.A.	Liu_et_al_2020	PASS
LaChi556	Lachi	MSEA	Vietnam	modern	0	Tai-Kadai	22.7	104.7	Lachi	N.A.	Liu_et_al_2020	PASS
LaChi557	Lachi	MSEA	Vietnam	modern	0	Tai-Kadai	22.7	104.7	Lachi	N.A.	Liu_et_al_2020	PASS
LaChi558	Lachi	MSEA	Vietnam	modern	0	Tai-Kadai	22.7	104.7	Lachi	N.A.	Liu_et_al_2020	PASS
LaChi559	Lachi	MSEA	Vietnam	modern	0	Tai-Kadai	22.7	104.7	Lachi	N.A.	Liu_et_al_2020	PASS
LaChi560	Lachi	MSEA	Vietnam	modern	0	Tai-Kadai	22.7	104.7	Lachi	N.A.	Liu_et_al_2020	PASS
LaHu342	Lahu_V	MSEA	Vietnam	modern	0	Sino-Tibetan	22.4	102.7	Lahu	N.A.	Liu_et_al_2020	PASS
LaHu344	Lahu_V	MSEA	Vietnam	modern	0	Sino-Tibetan	22.4	102.7	Lahu	N.A.	Liu_et_al_2020	PASS
LaHu347	Lahu_V	MSEA	Vietnam	modern	0	Sino-Tibetan	22.4	102.7	Lahu	N.A.	Liu_et_al_2020	PASS
LaHu348	Lahu_V	MSEA	Vietnam	modern	0	Sino-Tibetan	22.4	102.7	Lahu	N.A.	Liu_et_al_2020	PASS
LaHu350	Lahu_V	MSEA	Vietnam	modern	0	Sino-Tibetan	22.4	102.7	Lahu	N.A.	Liu_et_al_2020	PASS
LaHu351	Lahu_V	MSEA	Vietnam	modern	0	Sino-Tibetan	22.4	102.7	Lahu	N.A.	Liu_et_al_2020	PASS
LaHu352	Lahu_V	MSEA	Vietnam	modern	0	Sino-Tibetan	22.4	102.7	Lahu	N.A.	Liu_et_al_2020	kin
LaHu353	Lahu_V	MSEA	Vietnam	modern	0	Sino-Tibetan	22.4	102.7	Lahu	N.A.	Liu_et_al_2020	kin
LaHu354	Lahu_V	MSEA	Vietnam	modern	0	Sino-Tibetan	22.4	102.7	Lahu	N.A.	Liu_et_al_2020	kin
LaHu355	Lahu_V	MSEA	Vietnam	modern	0	Sino-Tibetan	22.4	102.7	Lahu	N.A.	Liu_et_al_2020	PASS
LaHu356	Lahu_V	MSEA	Vietnam	modern	0	Sino-Tibetan	22.4	102.7	Lahu	N.A.	Liu_et_al_2020	PASS
LaHu360	Lahu_V	MSEA	Vietnam	modern	0	Sino-Tibetan	22.4	102.7	Lahu	N.A.	Liu_et_al_2020	kin
LoLo469	Lolo	MSEA	Vietnam	modern	0	Sino-Tibetan	23.1	105.4	Lolo	N.A.	Liu_et_al_2020	PASS
Lolo470	Lolo	MSEA	Vietnam	modern	0	Sino-Tibetan	23.1	105.4	Lolo	N.A.	Liu_et_al_2020	PASS
LoLo471	Lolo	MSEA	Vietnam	modern	0	Sino-Tibetan	23.1	105.4	Lolo	N.A.	Liu_et_al_2020	PASS
LoLo472	Lolo	MSEA	Vietnam	modern	0	Sino-Tibetan	23.1	105.4	Lolo	N.A.	Liu_et_al_2020	kin
LoLo473	Lolo	MSEA	Vietnam	modern	0	Sino-Tibetan	23.1	105.4	Lolo	N.A.	Liu_et_al_2020	PASS
LoLo474	Lolo	MSEA	Vietnam	modern	0	Sino-Tibetan	23.1	105.4	Lolo	N.A.	Liu_et_al_2020	PASS

LoLo475	Lolo	MSEA	Vietnam	modern	0	Sino-Tibetan	23.1	105.4	Lolo	N.A.	Liu_et_al_2020	PASS
LoLo476	Lolo	MSEA	Vietnam	modern	0	Sino-Tibetan	23.1	105.4	Lolo	N.A.	Liu_et_al_2020	PASS
LoLo477	Lolo	MSEA	Vietnam	modern	0	Sino-Tibetan	23.1	105.4	Lolo	N.A.	Liu_et_al_2020	kin
LoLo478	Lolo	MSEA	Vietnam	modern	0	Sino-Tibetan	23.1	105.4	Lolo	N.A.	Liu_et_al_2020	PASS
LoLo479	Lolo	MSEA	Vietnam	modern	0	Sino-Tibetan	23.1	105.4	Lolo	N.A.	Liu_et_al_2020	PASS
LoLo480	Lolo	MSEA	Vietnam	modern	0	Sino-Tibetan	23.1	105.4	Lolo	N.A.	Liu_et_al_2020	PASS
Mang273	Mang	MSEA	Vietnam	modern	0	Austroasiatic	22.4	102.7	N.A.	N.A.	Liu_et_al_2020	PASS
Mang274	Mang	MSEA	Vietnam	modern	0	Austroasiatic	22.4	102.7	N.A.	N.A.	Liu_et_al_2020	PASS
Mang276	Mang	MSEA	Vietnam	modern	0	Austroasiatic	22.4	102.7	N.A.	N.A.	Liu_et_al_2020	PASS
Mang278_2	Mang	MSEA	Vietnam	modern	0	Austroasiatic	22.4	102.7	N.A.	N.A.	Liu_et_al_2020	PASS
Mang284_1	Mang	MSEA	Vietnam	modern	0	Austroasiatic	22.4	102.7	N.A.	N.A.	Liu_et_al_2020	PASS
Mang289	Mang	MSEA	Vietnam	modern	0	Austroasiatic	22.4	102.7	N.A.	N.A.	Liu_et_al_2020	PASS
Mang290	Mang	MSEA	Vietnam	modern	0	Austroasiatic	22.4	102.7	N.A.	N.A.	Liu_et_al_2020	PASS
Mang291	Mang	MSEA	Vietnam	modern	0	Austroasiatic	22.4	102.7	N.A.	N.A.	Liu_et_al_2020	PASS
Mang298	Mang	MSEA	Vietnam	modern	0	Austroasiatic	22.4	102.7	N.A.	N.A.	Liu_et_al_2020	PASS
Mang307_1	Mang	MSEA	Vietnam	modern	0	Austroasiatic	22.4	102.7	N.A.	N.A.	Liu_et_al_2020	PASS
Mang308	Mang	MSEA	Vietnam	modern	0	Austroasiatic	22.4	102.7	N.A.	N.A.	Liu_et_al_2020	PASS
Mang310	Mang	MSEA	Vietnam	modern	0	Austroasiatic	22.4	102.7	N.A.	N.A.	Liu_et_al_2020	kin
Muong35	Muong	MSEA	Vietnam	modern	0	Austroasiatic	20.4	105.6	N.A.	N.A.	Liu_et_al_2020	PASS
Muong36	Muong	MSEA	Vietnam	modern	0	Austroasiatic	20.4	105.6	N.A.	N.A.	Liu_et_al_2020	PASS
Muong38	Muong	MSEA	Vietnam	modern	0	Austroasiatic	20.5	105.4	N.A.	N.A.	Liu_et_al_2020	PASS
Muong39	Muong	MSEA	Vietnam	modern	0	Austroasiatic	20.7	105.6	N.A.	N.A.	Liu_et_al_2020	PASS
Muong40	Muong	MSEA	Vietnam	modern	0	Austroasiatic	20.9	105.5	N.A.	N.A.	Liu_et_al_2020	PASS
Muong41	Muong	MSEA	Vietnam	modern	0	Austroasiatic	20.7	105.6	N.A.	N.A.	Liu_et_al_2020	PASS
Muong42	Muong	MSEA	Vietnam	modern	0	Austroasiatic	20.7	105.6	N.A.	N.A.	Liu_et_al_2020	PASS
Muong43	Muong	MSEA	Vietnam	modern	0	Austroasiatic	20.5	105.4	N.A.	N.A.	Liu_et_al_2020	PASS
Muong121	Muong	MSEA	Vietnam	modern	0	Austroasiatic	20.5	105.4	N.A.	N.A.	Liu_et_al_2020	PASS
Muong132	Muong	MSEA	Vietnam	modern	0	Austroasiatic	20.5	105.4	N.A.	N.A.	Liu_et_al_2020	PASS
Nung47	Nung	MSEA	Vietnam	modern	0	Tai-Kadai	22.8	105	Nung	N.A.	Liu_et_al_2020	PASS
Nung48	Nung	MSEA	Vietnam	modern	0	Tai-Kadai	22.8	105	Nung	N.A.	Liu_et_al_2020	PASS
Nung49	Nung	MSEA	Vietnam	modern	0	Tai-Kadai	22.9	106.1	Nung	N.A.	Liu_et_al_2020	PASS
Nung664	Nung	MSEA	Vietnam	modern	0	Tai-Kadai	22.7	104.7	Nung	N.A.	Liu_et_al_2020	PASS
Nung672_1	Nung	MSEA	Vietnam	modern	0	Tai-Kadai	22.7	104.7	Nung	N.A.	Liu_et_al_2020	PASS
Nung674	Nung	MSEA	Vietnam	modern	0	Tai-Kadai	22.7	104.7	Nung	N.A.	Liu_et_al_2020	PASS
Nung677	Nung	MSEA	Vietnam	modern	0	Tai-Kadai	22.7	104.7	Nung	N.A.	Liu_et_al_2020	PASS
Nung678	Nung	MSEA	Vietnam	modern	0	Tai-Kadai	22.7	104.7	Nung	N.A.	Liu_et_al_2020	PASS
Nung680	Nung	MSEA	Vietnam	modern	0	Tai-Kadai	22.7	104.7	Nung	N.A.	Liu_et_al_2020	PASS
Nung681	Nung	MSEA	Vietnam	modern	0	Tai-Kadai	22.7	104.7	Nung	N.A.	Liu_et_al_2020	PASS
Nung687	Nung	MSEA	Vietnam	modern	0	Tai-Kadai	22.7	104.7	Nung	N.A.	Liu_et_al_2020	PASS
Nung691	Nung	MSEA	Vietnam	modern	0	Tai-Kadai	22.7	104.7	Nung	N.A.	Liu_et_al_2020	PASS
PaThen494	Pathen	MSEA	Vietnam	modern	0	Hmong-Mien	22.4	104.7	N.A.	N.A.	Liu_et_al_2020	PASS
PaThen495	Pathen	MSEA	Vietnam	modern	0	Hmong-Mien	22.4	104.7	N.A.	N.A.	Liu_et_al_2020	PASS
PaThen496	Pathen	MSEA	Vietnam	modern	0	Hmong-Mien	22.4	104.7	N.A.	N.A.	Liu_et_al_2020	PASS
PaThen497	Pathen	MSEA	Vietnam	modern	0	Hmong-Mien	22.4	104.7	N.A.	N.A.	Liu_et_al_2020	PASS

PaThen498	Pathen	MSEA	Vietnam	modern	0	Hmong-Mien	22.4	104.7	N.A.	N.A.	Liu_et_al_2020	PASS
PaThen499	Pathen	MSEA	Vietnam	modern	0	Hmong-Mien	22.4	104.7	N.A.	N.A.	Liu_et_al_2020	PASS
PaThen500	Pathen	MSEA	Vietnam	modern	0	Hmong-Mien	22.4	104.7	N.A.	N.A.	Liu_et_al_2020	PASS
PaThen501	Pathen	MSEA	Vietnam	modern	0	Hmong-Mien	22.4	104.7	N.A.	N.A.	Liu_et_al_2020	PASS
PaThen502	Pathen	MSEA	Vietnam	modern	0	Hmong-Mien	22.4	104.7	N.A.	N.A.	Liu_et_al_2020	kin
PaThen503	Pathen	MSEA	Vietnam	modern	0	Hmong-Mien	22.4	104.7	N.A.	N.A.	Liu_et_al_2020	PASS
PaThen504	Pathen	MSEA	Vietnam	modern	0	Hmong-Mien	22.4	104.7	N.A.	N.A.	Liu_et_al_2020	PASS
PaThen505	Pathen	MSEA	Vietnam	modern	0	Hmong-Mien	22.4	104.7	N.A.	N.A.	Liu_et_al_2020	PASS
Phula141	Phula	MSEA	Vietnam	modern	0	Sino-Tibetan	22.7	104.1	Phula	N.A.	Liu_et_al_2020	PASS
PhuLa142	Phula	MSEA	Vietnam	modern	0	Sino-Tibetan	22.7	104.1	Phula	N.A.	Liu_et_al_2020	PASS
PhuLa143	Phula	MSEA	Vietnam	modern	0	Sino-Tibetan	22.7	104.1	Phula	N.A.	Liu_et_al_2020	PASS
PhuLa144	Phula	MSEA	Vietnam	modern	0	Sino-Tibetan	22.7	104.1	Phula	N.A.	Liu_et_al_2020	PASS
PhuLa145	Phula	MSEA	Vietnam	modern	0	Sino-Tibetan	22.7	104.1	Phula	N.A.	Liu_et_al_2020	PASS
PhuLa146	Phula	MSEA	Vietnam	modern	0	Sino-Tibetan	22.7	104.1	Phula	N.A.	Liu_et_al_2020	PASS
PhuLa147	Phula	MSEA	Vietnam	modern	0	Sino-Tibetan	22.7	104.1	Phula	N.A.	Liu_et_al_2020	PASS
PhuLa148	Phula	MSEA	Vietnam	modern	0	Sino-Tibetan	22.7	104.1	Phula	N.A.	Liu_et_al_2020	PASS
PhuLa149	Phula	MSEA	Vietnam	modern	0	Sino-Tibetan	22.7	104.1	Phula	N.A.	Liu_et_al_2020	PASS
PhuLa152	Phula	MSEA	Vietnam	modern	0	Sino-Tibetan	22.7	104.1	Phula	N.A.	Liu_et_al_2020	PASS
PhuLa153	Phula	MSEA	Vietnam	modern	0	Sino-Tibetan	22.7	104.1	Phula	N.A.	Liu_et_al_2020	PASS
Phula1450	Phula	MSEA	Vietnam	modern	0	Sino-Tibetan	22.7	104.1	Phula	N.A.	Liu_et_al_2020	PASS
Sila427	Sila	MSEA	Vietnam	modern	0	Sino-Tibetan	22.4	102.7	Sila	N.A.	Liu_et_al_2020	PASS
Sila428	Sila	MSEA	Vietnam	modern	0	Sino-Tibetan	22.4	102.7	Sila	N.A.	Liu_et_al_2020	PASS
Sila429	Sila	MSEA	Vietnam	modern	0	Sino-Tibetan	22.4	102.7	Sila	N.A.	Liu_et_al_2020	PASS
Sila433_1	Sila	MSEA	Vietnam	modern	0	Sino-Tibetan	22.4	102.7	Sila	N.A.	Liu_et_al_2020	PASS
Sila434	Sila	MSEA	Vietnam	modern	0	Sino-Tibetan	22.4	102.7	Sila	N.A.	Liu_et_al_2020	PASS
Sila435	Sila	MSEA	Vietnam	modern	0	Sino-Tibetan	22.4	102.7	Sila	N.A.	Liu_et_al_2020	kin
Sila436_2	Sila	MSEA	Vietnam	modern	0	Sino-Tibetan	22.4	102.7	Sila	N.A.	Liu_et_al_2020	PASS
Sila438	Sila	MSEA	Vietnam	modern	0	Sino-Tibetan	22.4	102.7	Sila	N.A.	Liu_et_al_2020	PASS
Sila439_2	Sila	MSEA	Vietnam	modern	0	Sino-Tibetan	22.4	102.7	Sila	N.A.	Liu_et_al_2020	PASS
Sila440	Sila	MSEA	Vietnam	modern	0	Sino-Tibetan	22.4	102.7	Sila	N.A.	Liu_et_al_2020	PASS
Sila441	Sila	MSEA	Vietnam	modern	0	Sino-Tibetan	22.4	102.7	Sila	N.A.	Liu_et_al_2020	PASS
Sila443	Sila	MSEA	Vietnam	modern	0	Sino-Tibetan	22.4	102.7	Sila	N.A.	Liu_et_al_2020	PASS
Tay84	Tay	MSEA	Vietnam	modern	0	Tai-Kadai	11.7	108.4	Tay	N.A.	Liu_et_al_2020	PASS
Tay87	Tay	MSEA	Vietnam	modern	0	Tai-Kadai	21.6	105.6	Tay	N.A.	Liu_et_al_2020	PASS
Tay138	Tay	MSEA	Vietnam	modern	0	Tai-Kadai	22.7	104.1	Tay	N.A.	Liu_et_al_2020	PASS
Tay155	Tay	MSEA	Vietnam	modern	0	Tai-Kadai	22.7	104.1	Tay	N.A.	Liu_et_al_2020	PASS
Tay156	Tay	MSEA	Vietnam	modern	0	Tai-Kadai	22.7	104.1	Tay	N.A.	Liu_et_al_2020	PASS
Tay157	Tay	MSEA	Vietnam	modern	0	Tai-Kadai	22.7	104.1	Tay	N.A.	Liu_et_al_2020	PASS
Tay158	Tay	MSEA	Vietnam	modern	0	Tai-Kadai	22.7	104.1	Tay	N.A.	Liu_et_al_2020	PASS
Tay160	Tay	MSEA	Vietnam	modern	0	Tai-Kadai	22.7	104.1	Tay	N.A.	Liu_et_al_2020	PASS
Tay161	Tay	MSEA	Vietnam	modern	0	Tai-Kadai	22.7	104.1	Tay	N.A.	Liu_et_al_2020	PASS
Tay163	Tay	MSEA	Vietnam	modern	0	Tai-Kadai	22.7	104.1	Tay	N.A.	Liu_et_al_2020	PASS
Tay164	Tay	MSEA	Vietnam	modern	0	Tai-Kadai	22.7	104.1	Tay	N.A.	Liu_et_al_2020	PASS
Tay162_1	Tay	MSEA	Vietnam	modern	0	Tai-Kadai	22.7	104.1	Tay	N.A.	Liu_et_al_2020	PASS

Thai55	Thai_V	MSEA	Vietnam	modern	0	Tai-Kadai	22	103.8	Thai_V	N.A.	Liu_et_al_2020	PASS
Thai56	Thai_V	MSEA	Vietnam	modern	0	Tai-Kadai	22	103.8	Thai_V	N.A.	Liu_et_al_2020	PASS
Thai57	Thai_V	MSEA	Vietnam	modern	0	Tai-Kadai	20.9	105.4	Thai_V	N.A.	Liu_et_al_2020	PASS
Thai248	Thai_V	MSEA	Vietnam	modern	0	Tai-Kadai	21.4	103	Thai_V	N.A.	Liu_et_al_2020	PASS
Thai249_2	Thai_V	MSEA	Vietnam	modern	0	Tai-Kadai	21.4	103	Thai_V	N.A.	Liu_et_al_2020	PASS
Thai250	Thai_V	MSEA	Vietnam	modern	0	Tai-Kadai	21.4	103	Thai_V	N.A.	Liu_et_al_2020	PASS
Thai251	Thai_V	MSEA	Vietnam	modern	0	Tai-Kadai	21.4	103	Thai_V	N.A.	Liu_et_al_2020	PASS
Thai252	Thai_V	MSEA	Vietnam	modern	0	Tai-Kadai	21.4	103	Thai_V	N.A.	Liu_et_al_2020	PASS
Thai253_2	Thai_V	MSEA	Vietnam	modern	0	Tai-Kadai	21.4	103	Thai_V	N.A.	Liu_et_al_2020	PASS
Thai254	Thai_V	MSEA	Vietnam	modern	0	Tai-Kadai	21.4	103	Thai_V	N.A.	Liu_et_al_2020	PASS
Thai255	Thai_V	MSEA	Vietnam	modern	0	Tai-Kadai	21.4	103	Thai_V	N.A.	Liu_et_al_2020	PASS
Thai257	Thai_V	MSEA	Vietnam	modern	0	Tai-Kadai	21.4	103	Thai_V	N.A.	Liu_et_al_2020	PASS
HM401	HmongDaw	MSEA	Thailand	modern	0	Hmong-Mien	19.9	100.11	N.A.	N.A.	Kutanan_et_al_2021	PASS
HM403	HmongDaw	MSEA	Thailand	modern	0	Hmong-Mien	19.9	100.11	N.A.	N.A.	Kutanan_et_al_2021	PASS
HM404	HmongDaw	MSEA	Thailand	modern	0	Hmong-Mien	19.9	100.11	N.A.	N.A.	Kutanan_et_al_2021	PASS
HM405	HmongDaw	MSEA	Thailand	modern	0	Hmong-Mien	19.9	100.11	N.A.	N.A.	Kutanan_et_al_2021	PASS
HM406	HmongDaw	MSEA	Thailand	modern	0	Hmong-Mien	19.9	100.11	N.A.	N.A.	Kutanan_et_al_2021	PASS
HM407	HmongDaw	MSEA	Thailand	modern	0	Hmong-Mien	19.9	100.11	N.A.	N.A.	Kutanan_et_al_2021	PASS
HM409	HmongDaw	MSEA	Thailand	modern	0	Hmong-Mien	19.9	100.11	N.A.	N.A.	Kutanan_et_al_2021	PASS
HM413	HmongDaw	MSEA	Thailand	modern	0	Hmong-Mien	19.9	100.11	N.A.	N.A.	Kutanan_et_al_2021	PASS
HM415	HmongDaw	MSEA	Thailand	modern	0	Hmong-Mien	19.9	100.11	N.A.	N.A.	Kutanan_et_al_2021	PASS
HM101	HmongNjua	MSEA	Thailand	modern	0	Hmong-Mien	18.12	98.23	N.A.	N.A.	Kutanan_et_al_2021	PASS
HM102	HmongNjua	MSEA	Thailand	modern	0	Hmong-Mien	18.12	98.23	N.A.	N.A.	Kutanan_et_al_2021	PASS
HM103	HmongNjua	MSEA	Thailand	modern	0	Hmong-Mien	19.09	100.56	N.A.	N.A.	Kutanan_et_al_2021	PASS
HM104	HmongNjua	MSEA	Thailand	modern	0	Hmong-Mien	18.12	98.23	N.A.	N.A.	Kutanan_et_al_2021	PASS
HM105	HmongNjua	MSEA	Thailand	modern	0	Hmong-Mien	18.12	98.23	N.A.	N.A.	Kutanan_et_al_2021	kin
HM107	HmongNjua	MSEA	Thailand	modern	0	Hmong-Mien	18.12	98.23	N.A.	N.A.	Kutanan_et_al_2021	PASS
HM108	HmongNjua	MSEA	Thailand	modern	0	Hmong-Mien	18.12	98.23	N.A.	N.A.	Kutanan_et_al_2021	PASS
HM109	HmongNjua	MSEA	Thailand	modern	0	Hmong-Mien	18.12	98.23	N.A.	N.A.	Kutanan_et_al_2021	PASS
HM110	HmongNjua	MSEA	Thailand	modern	0	Hmong-Mien	18.12	98.23	N.A.	N.A.	Kutanan_et_al_2021	kin
HM111	HmongNjua	MSEA	Thailand	modern	0	Hmong-Mien	18.12	98.23	N.A.	N.A.	Kutanan_et_al_2021	PASS
Y101	IuMien	MSEA	Thailand	modern	0	Hmong-Mien	19.34	100.43	N.A.	N.A.	Kutanan_et_al_2021	PASS
Y102	IuMien	MSEA	Thailand	modern	0	Hmong-Mien	19.34	100.43	N.A.	N.A.	Kutanan_et_al_2021	PASS
Y104	IuMien	MSEA	Thailand	modern	0	Hmong-Mien	19.34	100.43	N.A.	N.A.	Kutanan_et_al_2021	PASS
Y107	IuMien	MSEA	Thailand	modern	0	Hmong-Mien	19.34	100.43	N.A.	N.A.	Kutanan_et_al_2021	PASS
Y109	IuMien	MSEA	Thailand	modern	0	Hmong-Mien	19.34	100.43	N.A.	N.A.	Kutanan_et_al_2021	PASS
Y215	IuMien	MSEA	Thailand	modern	0	Hmong-Mien	19.34	100.43	N.A.	N.A.	Kutanan_et_al_2021	PASS
Y273	IuMien	MSEA	Thailand	modern	0	Hmong-Mien	19.34	100.43	N.A.	N.A.	Kutanan_et_al_2021	PASS
Y275	IuMien	MSEA	Thailand	modern	0	Hmong-Mien	19.34	100.43	N.A.	N.A.	Kutanan_et_al_2021	PASS
MST101	SouthernThai_AN	MSEA	Thailand	modern	0	Austronesian	6.77	101.08	Southern Thai_AN	N.A.	Kutanan_et_al_2021	PASS
MST104	SouthernThai_AN	MSEA	Thailand	modern	0	Austronesian	6.77	101.08	Southern Thai_AN	N.A.	Kutanan_et_al_2021	PASS
MST106	SouthernThai_AN	MSEA	Thailand	modern	0	Austronesian	6.77	101.08	Southern Thai_AN	N.A.	Kutanan_et_al_2021	PASS
MST108	SouthernThai_AN	MSEA	Thailand	modern	0	Austronesian	6.77	101.08	Southern Thai_AN	N.A.	Kutanan_et_al_2021	PASS
MST202	SouthernThai_AN	MSEA	Thailand	modern	0	Austronesian	6.77	101.08	Southern Thai_AN	N.A.	Kutanan_et_al_2021	PASS

MST204	SouthernThai_AN	MSEA	Thailand	modern	0	Austronesian	6.77	101.08	Southern Thai_AN	N.A.	Kutanan_et_al_2021	PASS
MST206	SouthernThai_AN	MSEA	Thailand	modern	0	Austronesian	6.77	101.08	Southern Thai_AN	N.A.	Kutanan_et_al_2021	PASS
MST208	SouthernThai_AN	MSEA	Thailand	modern	0	Austronesian	6.77	101.08	Southern Thai_AN	N.A.	Kutanan_et_al_2021	PASS
MST302	SouthernThai_AN	MSEA	Thailand	modern	0	Austronesian	6.77	101.08	Southern Thai_AN	N.A.	Kutanan_et_al_2021	PASS
MST304	SouthernThai_AN	MSEA	Thailand	modern	0	Austronesian	6.77	101.08	Southern Thai_AN	N.A.	Kutanan_et_al_2021	PASS
MST306	SouthernThai_AN	MSEA	Thailand	modern	0	Austronesian	6.77	101.08	Southern Thai_AN	N.A.	Kutanan_et_al_2021	PASS
MST308	SouthernThai_AN	MSEA	Thailand	modern	0	Austronesian	6.77	101.08	Southern Thai_AN	N.A.	Kutanan_et_al_2021	PASS
MST401	SouthernThai_AN	MSEA	Thailand	modern	0	Austronesian	6.77	101.08	Southern Thai_AN	N.A.	Kutanan_et_al_2021	PASS
MST402	SouthernThai_AN	MSEA	Thailand	modern	0	Austronesian	6.77	101.08	Southern Thai_AN	N.A.	Kutanan_et_al_2021	PASS
MST403	SouthernThai_AN	MSEA	Thailand	modern	0	Austronesian	6.77	101.08	Southern Thai_AN	N.A.	Kutanan_et_al_2021	PASS
LS01	Lisu	MSEA	Thailand	modern	0	Sino-Tibetan	19.5	98.23	Lisu	N.A.	Kutanan_et_al_2021	PASS
LS02	Lisu	MSEA	Thailand	modern	0	Sino-Tibetan	19.5	98.23	Lisu	N.A.	Kutanan_et_al_2021	PASS
LS03	Lisu	MSEA	Thailand	modern	0	Sino-Tibetan	19.5	98.23	Lisu	N.A.	Kutanan_et_al_2021	PASS
LS04	Lisu	MSEA	Thailand	modern	0	Sino-Tibetan	19.5	98.23	Lisu	N.A.	Kutanan_et_al_2021	PASS
LS05	Lisu	MSEA	Thailand	modern	0	Sino-Tibetan	19.5	98.23	Lisu	N.A.	Kutanan_et_al_2021	PASS
LS06	Lisu	MSEA	Thailand	modern	0	Sino-Tibetan	19.5	98.23	Lisu	N.A.	Kutanan_et_al_2021	PASS
LS07	Lisu	MSEA	Thailand	modern	0	Sino-Tibetan	19.5	98.23	Lisu	N.A.	Kutanan_et_al_2021	PASS
LS09	Lisu	MSEA	Thailand	modern	0	Sino-Tibetan	19.5	98.23	Lisu	N.A.	Kutanan_et_al_2021	PASS
LS10	Lisu	MSEA	Thailand	modern	0	Sino-Tibetan	19.5	98.23	Lisu	N.A.	Kutanan_et_al_2021	PASS
MB01	Lahu_T	MSEA	Thailand	modern	0	Sino-Tibetan	19.58	98.21	Lahu	N.A.	Kutanan_et_al_2021	PASS
MB02	Lahu_T	MSEA	Thailand	modern	0	Sino-Tibetan	19.58	98.21	Lahu	N.A.	Kutanan_et_al_2021	PASS
MB03	Lahu_T	MSEA	Thailand	modern	0	Sino-Tibetan	19.58	98.21	Lahu	N.A.	Kutanan_et_al_2021	PASS
MB04	Lahu_T	MSEA	Thailand	modern	0	Sino-Tibetan	19.58	98.21	Lahu	N.A.	Kutanan_et_al_2021	PASS
MB05	Lahu_T	MSEA	Thailand	modern	0	Sino-Tibetan	19.58	98.21	Lahu	N.A.	Kutanan_et_al_2021	PASS
MB06	Lahu_T	MSEA	Thailand	modern	0	Sino-Tibetan	19.58	98.21	Lahu	N.A.	Kutanan_et_al_2021	PASS
MB07	Lahu_T	MSEA	Thailand	modern	0	Sino-Tibetan	19.58	98.21	Lahu	N.A.	Kutanan_et_al_2021	PASS
MB08	Lahu_T	MSEA	Thailand	modern	0	Sino-Tibetan	19.58	98.21	Lahu	N.A.	Kutanan_et_al_2021	PASS
MB09	Lahu_T	MSEA	Thailand	modern	0	Sino-Tibetan	19.58	98.21	Lahu	N.A.	Kutanan_et_al_2021	kin
MB10	Lahu_T	MSEA	Thailand	modern	0	Sino-Tibetan	19.58	98.21	Lahu	N.A.	Kutanan_et_al_2021	PASS
MR01	Lahu_T	MSEA	Thailand	modern	0	Sino-Tibetan	19.53	98.27	Lahu	N.A.	Kutanan_et_al_2021	PASS
MR02	Lahu_T	MSEA	Thailand	modern	0	Sino-Tibetan	19.53	98.27	Lahu	N.A.	Kutanan_et_al_2021	PASS
MR03	Lahu_T	MSEA	Thailand	modern	0	Sino-Tibetan	19.53	98.27	Lahu	N.A.	Kutanan_et_al_2021	PASS
MR05	Lahu_T	MSEA	Thailand	modern	0	Sino-Tibetan	19.53	98.27	Lahu	N.A.	Kutanan_et_al_2021	PASS
MR06	Lahu_T	MSEA	Thailand	modern	0	Sino-Tibetan	19.53	98.27	Lahu	N.A.	Kutanan_et_al_2021	PASS
MR07	Lahu_T	MSEA	Thailand	modern	0	Sino-Tibetan	19.53	98.27	Lahu	N.A.	Kutanan_et_al_2021	PASS
MR08	Lahu_T	MSEA	Thailand	modern	0	Sino-Tibetan	19.53	98.27	Lahu	N.A.	Kutanan_et_al_2021	PASS
MR09	Lahu_T	MSEA	Thailand	modern	0	Sino-Tibetan	19.53	98.27	Lahu	N.A.	Kutanan_et_al_2021	PASS
MR10	Lahu_T	MSEA	Thailand	modern	0	Sino-Tibetan	19.53	98.27	Lahu	N.A.	Kutanan_et_al_2021	PASS
MR11	Lahu_T	MSEA	Thailand	modern	0	Sino-Tibetan	19.53	98.27	Lahu	N.A.	Kutanan_et_al_2021	PASS
EMS301	KarenPwo	MSEA	Thailand	modern	0	Sino-Tibetan	18.15	97.93	Karen_ST	N.A.	Kutanan_et_al_2021	PASS
EMS305	KarenPwo	MSEA	Thailand	modern	0	Sino-Tibetan	18.15	97.93	Karen_ST	N.A.	Kutanan_et_al_2021	PASS
EMS306	KarenPwo	MSEA	Thailand	modern	0	Sino-Tibetan	18.15	97.93	Karen_ST	N.A.	Kutanan_et_al_2021	PASS
EMS308	KarenPwo	MSEA	Thailand	modern	0	Sino-Tibetan	18.15	97.93	Karen_ST	N.A.	Kutanan_et_al_2021	PASS
EMS309	KarenPwo	MSEA	Thailand	modern	0	Sino-Tibetan	18.15	97.93	Karen_ST	N.A.	Kutanan_et_al_2021	PASS

EMS313	KarenPwo	MSEA	Thailand	modern	0	Sino-Tibetan	18.15	97.93	Karen_ST	N.A.	Kutanan_et_al_2021	PASS
EMS314	KarenPwo	MSEA	Thailand	modern	0	Sino-Tibetan	18.15	97.93	Karen_ST	N.A.	Kutanan_et_al_2021	PASS
EMS316	KarenPwo	MSEA	Thailand	modern	0	Sino-Tibetan	18.15	97.93	Karen_ST	N.A.	Kutanan_et_al_2021	PASS
EMS318	KarenPwo	MSEA	Thailand	modern	0	Sino-Tibetan	18.15	97.93	Karen_ST	N.A.	Kutanan_et_al_2021	PASS
EMS322	KarenPwo	MSEA	Thailand	modern	0	Sino-Tibetan	18.15	97.93	Karen_ST	N.A.	Kutanan_et_al_2021	PASS
EPB501	KarenPadaung	MSEA	Thailand	modern	0	Sino-Tibetan	19.14	97.93	Karen_ST	N.A.	Kutanan_et_al_2021	PASS
EPB504	KarenPadaung	MSEA	Thailand	modern	0	Sino-Tibetan	19.14	97.93	Karen_ST	N.A.	Kutanan_et_al_2021	PASS
EPB507	KarenPadaung	MSEA	Thailand	modern	0	Sino-Tibetan	19.14	97.93	Karen_ST	N.A.	Kutanan_et_al_2021	PASS
EPB508	KarenPadaung	MSEA	Thailand	modern	0	Sino-Tibetan	19.14	97.93	Karen_ST	N.A.	Kutanan_et_al_2021	PASS
EPB511	KarenPadaung	MSEA	Thailand	modern	0	Sino-Tibetan	19.14	97.93	Karen_ST	N.A.	Kutanan_et_al_2021	PASS
EPB516	KarenPadaung	MSEA	Thailand	modern	0	Sino-Tibetan	19.14	97.93	Karen_ST	N.A.	Kutanan_et_al_2021	PASS
EPB522	KarenPadaung	MSEA	Thailand	modern	0	Sino-Tibetan	19.14	97.93	Karen_ST	N.A.	Kutanan_et_al_2021	PASS
EPB523	KarenPadaung	MSEA	Thailand	modern	0	Sino-Tibetan	19.14	97.93	Karen_ST	N.A.	Kutanan_et_al_2021	PASS
EPB525	KarenPadaung	MSEA	Thailand	modern	0	Sino-Tibetan	19.14	97.93	Karen_ST	N.A.	Kutanan_et_al_2021	PASS
EPB528	KarenPadaung	MSEA	Thailand	modern	0	Sino-Tibetan	19.14	97.93	Karen_ST	N.A.	Kutanan_et_al_2021	PASS
KR104	KarenSkaw	MSEA	Thailand	modern	0	Sino-Tibetan	19.58	98.31	Karen_ST	N.A.	Kutanan_et_al_2021	PASS
KR105	KarenSkaw	MSEA	Thailand	modern	0	Sino-Tibetan	19.58	98.31	Karen_ST	N.A.	Kutanan_et_al_2021	PASS
KR106	KarenSkaw	MSEA	Thailand	modern	0	Sino-Tibetan	19.58	98.31	Karen_ST	N.A.	Kutanan_et_al_2021	PASS
KR107	KarenSkaw	MSEA	Thailand	modern	0	Sino-Tibetan	19.58	98.31	Karen_ST	N.A.	Kutanan_et_al_2021	PASS
KR108	KarenSkaw	MSEA	Thailand	modern	0	Sino-Tibetan	19.58	98.31	Karen_ST	N.A.	Kutanan_et_al_2021	PASS
KR111	KarenSkaw	MSEA	Thailand	modern	0	Sino-Tibetan	19.58	98.31	Karen_ST	N.A.	Kutanan_et_al_2021	PASS
KR113	KarenSkaw	MSEA	Thailand	modern	0	Sino-Tibetan	19.58	98.31	Karen_ST	N.A.	Kutanan_et_al_2021	PASS
KR115	KarenSkaw	MSEA	Thailand	modern	0	Sino-Tibetan	19.58	98.31	Karen_ST	N.A.	Kutanan_et_al_2021	PASS
KR117	KarenSkaw	MSEA	Thailand	modern	0	Sino-Tibetan	19.58	98.31	Karen_ST	N.A.	Kutanan_et_al_2021	PASS
KR122	KarenSkaw	MSEA	Thailand	modern	0	Sino-Tibetan	19.58	98.31	Karen_ST	N.A.	Kutanan_et_al_2021	PASS
LW601	Lawa_Western	MSEA	Thailand	modern	0	Austroasiatic	18.08	98.2	N.A.	N.A.	Kutanan_et_al_2021	PASS
LW603	Lawa_Western	MSEA	Thailand	modern	0	Austroasiatic	18.08	98.2	N.A.	N.A.	Kutanan_et_al_2021	PASS
LW609	Lawa_Western	MSEA	Thailand	modern	0	Austroasiatic	18.08	98.2	N.A.	N.A.	Kutanan_et_al_2021	PASS
LW611	Lawa_Western	MSEA	Thailand	modern	0	Austroasiatic	18.08	98.2	N.A.	N.A.	Kutanan_et_al_2021	PASS
LW612	Lawa_Western	MSEA	Thailand	modern	0	Austroasiatic	18.08	98.2	N.A.	N.A.	Kutanan_et_al_2021	PASS
LW615	Lawa_Western	MSEA	Thailand	modern	0	Austroasiatic	18.08	98.2	N.A.	N.A.	Kutanan_et_al_2021	PASS
LW617	Lawa_Western	MSEA	Thailand	modern	0	Austroasiatic	18.08	98.2	N.A.	N.A.	Kutanan_et_al_2021	PASS
LW619	Lawa_Western	MSEA	Thailand	modern	0	Austroasiatic	18.08	98.2	N.A.	N.A.	Kutanan_et_al_2021	PASS
LW625	Lawa_Western	MSEA	Thailand	modern	0	Austroasiatic	18.08	98.2	N.A.	N.A.	Kutanan_et_al_2021	PASS
LW502	Lawa_Eastern	MSEA	Thailand	modern	0	Austroasiatic	18.23	97.56	N.A.	N.A.	Kutanan_et_al_2021	PASS
LW504	Lawa_Eastern	MSEA	Thailand	modern	0	Austroasiatic	18.23	97.56	N.A.	N.A.	Kutanan_et_al_2021	PASS
LW506	Lawa_Eastern	MSEA	Thailand	modern	0	Austroasiatic	18.23	97.56	N.A.	N.A.	Kutanan_et_al_2021	PASS
LW510	Lawa_Eastern	MSEA	Thailand	modern	0	Austroasiatic	18.23	97.56	N.A.	N.A.	Kutanan_et_al_2021	PASS
LW514	Lawa_Eastern	MSEA	Thailand	modern	0	Austroasiatic	18.23	97.56	N.A.	N.A.	Kutanan_et_al_2021	PASS
LW520	Lawa_Eastern	MSEA	Thailand	modern	0	Austroasiatic	18.23	97.56	N.A.	N.A.	Kutanan_et_al_2021	PASS
LW522	Lawa_Eastern	MSEA	Thailand	modern	0	Austroasiatic	18.23	97.56	N.A.	N.A.	Kutanan_et_al_2021	PASS
LW524	Lawa_Eastern	MSEA	Thailand	modern	0	Austroasiatic	18.23	97.56	N.A.	N.A.	Kutanan_et_al_2021	PASS
LW526	Lawa_Eastern	MSEA	Thailand	modern	0	Austroasiatic	18.23	97.56	N.A.	N.A.	Kutanan_et_al_2021	PASS
LW528	Lawa_Eastern	MSEA	Thailand	modern	0	Austroasiatic	18.23	97.56	N.A.	N.A.	Kutanan_et_al_2021	PASS

PL113	Palaung	MSEA	Thailand	modern	0	Austroasiatic	20.25	99.52	N.A.	N.A.	Kutanan_et_al_2021	PASS
PL117	Palaung	MSEA	Thailand	modern	0	Austroasiatic	20.25	99.52	N.A.	N.A.	Kutanan_et_al_2021	PASS
PL119	Palaung	MSEA	Thailand	modern	0	Austroasiatic	20.25	99.52	N.A.	N.A.	Kutanan_et_al_2021	PASS
PL121	Palaung	MSEA	Thailand	modern	0	Austroasiatic	20.25	99.52	N.A.	N.A.	Kutanan_et_al_2021	kin
PL123	Palaung	MSEA	Thailand	modern	0	Austroasiatic	20.25	99.52	N.A.	N.A.	Kutanan_et_al_2021	PASS
PL127	Palaung	MSEA	Thailand	modern	0	Austroasiatic	20.25	99.52	N.A.	N.A.	Kutanan_et_al_2021	PASS
PL129	Palaung	MSEA	Thailand	modern	0	Austroasiatic	20.25	99.52	N.A.	N.A.	Kutanan_et_al_2021	PASS
PL133	Palaung	MSEA	Thailand	modern	0	Austroasiatic	20.25	99.52	N.A.	N.A.	Kutanan_et_al_2021	PASS
PL134	Palaung	MSEA	Thailand	modern	0	Austroasiatic	20.25	99.52	N.A.	N.A.	Kutanan_et_al_2021	PASS
PL145	Palaung	MSEA	Thailand	modern	0	Austroasiatic	20.25	99.52	N.A.	N.A.	Kutanan_et_al_2021	PASS
PP101	Blang	MSEA	Thailand	modern	0	Austroasiatic	19.56	99.09	N.A.	N.A.	Kutanan_et_al_2021	PASS
PP103	Blang	MSEA	Thailand	modern	0	Austroasiatic	19.56	99.09	N.A.	N.A.	Kutanan_et_al_2021	PASS
PP105	Blang	MSEA	Thailand	modern	0	Austroasiatic	19.56	99.09	N.A.	N.A.	Kutanan_et_al_2021	PASS
PP109	Blang	MSEA	Thailand	modern	0	Austroasiatic	19.56	99.09	N.A.	N.A.	Kutanan_et_al_2021	kin
PP119	Blang	MSEA	Thailand	modern	0	Austroasiatic	19.56	99.09	N.A.	N.A.	Kutanan_et_al_2021	PASS
PP211	Blang	MSEA	Thailand	modern	0	Austroasiatic	19.56	99.09	N.A.	N.A.	Kutanan_et_al_2021	PASS
PP257	Blang	MSEA	Thailand	modern	0	Austroasiatic	19.56	99.09	N.A.	N.A.	Kutanan_et_al_2021	PASS
PP259	Blang	MSEA	Thailand	modern	0	Austroasiatic	19.56	99.09	N.A.	N.A.	Kutanan_et_al_2021	PASS
PP263	Blang	MSEA	Thailand	modern	0	Austroasiatic	19.56	99.09	N.A.	N.A.	Kutanan_et_al_2021	PASS
PP265	Blang	MSEA	Thailand	modern	0	Austroasiatic	19.56	99.09	N.A.	N.A.	Kutanan_et_al_2021	PASS
KM102	Khmu	MSEA	Thailand	modern	0	Austroasiatic	19.49	100.89	N.A.	N.A.	Kutanan_et_al_2021	PASS
KM110	Khmu	MSEA	Thailand	modern	0	Austroasiatic	19.49	100.89	N.A.	N.A.	Kutanan_et_al_2021	PASS
KM115	Khmu	MSEA	Thailand	modern	0	Austroasiatic	19.49	100.89	N.A.	N.A.	Kutanan_et_al_2021	PASS
KM117	Khmu	MSEA	Thailand	modern	0	Austroasiatic	19.49	100.89	N.A.	N.A.	Kutanan_et_al_2021	PASS
KM119	Khmu	MSEA	Thailand	modern	0	Austroasiatic	19.49	100.89	N.A.	N.A.	Kutanan_et_al_2021	PASS
KM120	Khmu	MSEA	Thailand	modern	0	Austroasiatic	19.49	100.89	N.A.	N.A.	Kutanan_et_al_2021	PASS
KM121	Khmu	MSEA	Thailand	modern	0	Austroasiatic	19.49	100.89	N.A.	N.A.	Kutanan_et_al_2021	PASS
KM129	Khmu	MSEA	Thailand	modern	0	Austroasiatic	19.49	100.89	N.A.	N.A.	Kutanan_et_al_2021	PASS
KM133	Khmu	MSEA	Thailand	modern	0	Austroasiatic	19.49	100.89	N.A.	N.A.	Kutanan_et_al_2021	PASS
KM137	Khmu	MSEA	Thailand	modern	0	Austroasiatic	19.49	100.89	N.A.	N.A.	Kutanan_et_al_2021	PASS
TN201	HtinPray	MSEA	Thailand	modern	0	Austroasiatic	19.23	100.52	N.A.	N.A.	Kutanan_et_al_2021	PASS
TN207	HtinPray	MSEA	Thailand	modern	0	Austroasiatic	19.23	100.52	N.A.	N.A.	Kutanan_et_al_2021	PASS
TN209	HtinPray	MSEA	Thailand	modern	0	Austroasiatic	19.23	100.52	N.A.	N.A.	Kutanan_et_al_2021	PASS
TN213	HtinPray	MSEA	Thailand	modern	0	Austroasiatic	19.23	100.52	N.A.	N.A.	Kutanan_et_al_2021	PASS
TN219	HtinPray	MSEA	Thailand	modern	0	Austroasiatic	19.23	100.52	N.A.	N.A.	Kutanan_et_al_2021	kin
TN223	HtinPray	MSEA	Thailand	modern	0	Austroasiatic	19.23	100.52	N.A.	N.A.	Kutanan_et_al_2021	kin
TN227	HtinPray	MSEA	Thailand	modern	0	Austroasiatic	19.23	100.52	N.A.	N.A.	Kutanan_et_al_2021	PASS
TN229	HtinPray	MSEA	Thailand	modern	0	Austroasiatic	19.23	100.52	N.A.	N.A.	Kutanan_et_al_2021	PASS
TN231	HtinPray	MSEA	Thailand	modern	0	Austroasiatic	19.23	100.52	N.A.	N.A.	Kutanan_et_al_2021	PASS
TN234	HtinPray	MSEA	Thailand	modern	0	Austroasiatic	19.23	100.52	N.A.	N.A.	Kutanan_et_al_2021	kin
TN236	HtinPray	MSEA	Thailand	modern	0	Austroasiatic	19.23	100.52	N.A.	N.A.	Kutanan_et_al_2021	kin
TN301	HtinPray	MSEA	Thailand	modern	0	Austroasiatic	19.23	100.52	N.A.	N.A.	Kutanan_et_al_2021	PASS
TN303	HtinPray	MSEA	Thailand	modern	0	Austroasiatic	19.23	100.52	N.A.	N.A.	Kutanan_et_al_2021	PASS
TN307	HtinPray	MSEA	Thailand	modern	0	Austroasiatic	19.23	100.52	N.A.	N.A.	Kutanan_et_al_2021	PASS

TN313	HtinPray	MSEA	Thailand	modern	0	Austroasiatic	19.23	100.52	N.A.	N.A.	Kutanan_et_al_2021	PASS
TN102	HtinMal	MSEA	Thailand	modern	0	Austroasiatic	19.08	100.55	N.A.	N.A.	Lipson_et_al_2018	PASS
TN105	HtinMal	MSEA	Thailand	modern	0	Austroasiatic	19.08	100.55	N.A.	N.A.	Lipson_et_al_2018	PASS
TN106	HtinMal	MSEA	Thailand	modern	0	Austroasiatic	19.08	100.55	N.A.	N.A.	Lipson_et_al_2018	PASS
TN110	HtinMal	MSEA	Thailand	modern	0	Austroasiatic	19.08	100.55	N.A.	N.A.	Lipson_et_al_2018	PASS
TN112	HtinMal	MSEA	Thailand	modern	0	Austroasiatic	19.08	100.55	N.A.	N.A.	Lipson_et_al_2018	PASS
TN115	HtinMal	MSEA	Thailand	modern	0	Austroasiatic	19.08	100.55	N.A.	N.A.	Lipson_et_al_2018	PASS
TN122	HtinMal	MSEA	Thailand	modern	0	Austroasiatic	19.08	100.55	N.A.	N.A.	Lipson_et_al_2018	PASS
TN123	HtinMal	MSEA	Thailand	modern	0	Austroasiatic	19.08	100.55	N.A.	N.A.	Lipson_et_al_2018	PASS
TN125	HtinMal	MSEA	Thailand	modern	0	Austroasiatic	19.08	100.55	N.A.	N.A.	Lipson_et_al_2018	PASS
TN147	HtinMal	MSEA	Thailand	modern	0	Austroasiatic	19.08	100.55	N.A.	N.A.	Lipson_et_al_2018	PASS
MA107	Mlabri	MSEA	Thailand	modern	0	Austroasiatic	18.61	100.71	N.A.	N.A.	Lipson_et_al_2018	PASS
MA112	Mlabri	MSEA	Thailand	modern	0	Austroasiatic	18.61	100.71	N.A.	N.A.	Lipson_et_al_2018	PASS
MA113	Mlabri	MSEA	Thailand	modern	0	Austroasiatic	18.61	100.71	N.A.	N.A.	Lipson_et_al_2018	PASS
MA115	Mlabri	MSEA	Thailand	modern	0	Austroasiatic	18.61	100.71	N.A.	N.A.	Lipson_et_al_2018	PASS
MA117	Mlabri	MSEA	Thailand	modern	0	Austroasiatic	18.61	100.71	N.A.	N.A.	Lipson_et_al_2018	PASS
MA119	Mlabri	MSEA	Thailand	modern	0	Austroasiatic	18.61	100.71	N.A.	N.A.	Lipson_et_al_2018	PASS
MA122	Mlabri	MSEA	Thailand	modern	0	Austroasiatic	18.61	100.71	N.A.	N.A.	Lipson_et_al_2018	PASS
MA124	Mlabri	MSEA	Thailand	modern	0	Austroasiatic	18.61	100.71	N.A.	N.A.	Lipson_et_al_2018	PASS
MA126	Mlabri	MSEA	Thailand	modern	0	Austroasiatic	18.61	100.71	N.A.	N.A.	Lipson_et_al_2018	PASS
MA128	Mlabri	MSEA	Thailand	modern	0	Austroasiatic	18.61	100.71	N.A.	N.A.	Lipson_et_al_2018	kin
SOA101	Soa	MSEA	Thailand	modern	0	Austroasiatic	17.37	104.3	N.A.	N.A.	Kutanan_et_al_2021	PASS
SOA105	Soa	MSEA	Thailand	modern	0	Austroasiatic	17.37	104.3	N.A.	N.A.	Kutanan_et_al_2021	PASS
SOA107	Soa	MSEA	Thailand	modern	0	Austroasiatic	17.37	104.3	N.A.	N.A.	Kutanan_et_al_2021	PASS
SOA109	Soa	MSEA	Thailand	modern	0	Austroasiatic	17.37	104.3	N.A.	N.A.	Kutanan_et_al_2021	PASS
SOA111	Soa	MSEA	Thailand	modern	0	Austroasiatic	17.37	104.3	N.A.	N.A.	Kutanan_et_al_2021	PASS
SOA113	Soa	MSEA	Thailand	modern	0	Austroasiatic	17.37	104.3	N.A.	N.A.	Kutanan_et_al_2021	PASS
SOA115	Soa	MSEA	Thailand	modern	0	Austroasiatic	17.37	104.3	N.A.	N.A.	Kutanan_et_al_2021	PASS
SOA117	Soa	MSEA	Thailand	modern	0	Austroasiatic	17.37	104.3	N.A.	N.A.	Kutanan_et_al_2021	PASS
SOA119	Soa	MSEA	Thailand	modern	0	Austroasiatic	17.37	104.3	N.A.	N.A.	Kutanan_et_al_2021	PASS
SOA121	Soa	MSEA	Thailand	modern	0	Austroasiatic	17.37	104.3	N.A.	N.A.	Kutanan_et_al_2021	PASS
SOA123	Soa	MSEA	Thailand	modern	0	Austroasiatic	17.37	104.3	N.A.	N.A.	Kutanan_et_al_2021	PASS
SOA125	Soa	MSEA	Thailand	modern	0	Austroasiatic	17.37	104.3	N.A.	N.A.	Kutanan_et_al_2021	PASS
SOA127	Soa	MSEA	Thailand	modern	0	Austroasiatic	17.37	104.3	N.A.	N.A.	Kutanan_et_al_2021	PASS
SOA129	Soa	MSEA	Thailand	modern	0	Austroasiatic	17.37	104.3	N.A.	N.A.	Kutanan_et_al_2021	PASS
SOA137	Soa	MSEA	Thailand	modern	0	Austroasiatic	17.37	104.3	N.A.	N.A.	Kutanan_et_al_2021	PASS
BRU101	Bru	MSEA	Thailand	modern	0	Austroasiatic	17.39	103.7	N.A.	N.A.	Kutanan_et_al_2021	PASS
BRU103	Bru	MSEA	Thailand	modern	0	Austroasiatic	17.39	103.7	N.A.	N.A.	Kutanan_et_al_2021	PASS
BRU105	Bru	MSEA	Thailand	modern	0	Austroasiatic	17.39	103.7	N.A.	N.A.	Kutanan_et_al_2021	kin
BRU107	Bru	MSEA	Thailand	modern	0	Austroasiatic	17.39	103.7	N.A.	N.A.	Kutanan_et_al_2021	PASS
BRU109	Bru	MSEA	Thailand	modern	0	Austroasiatic	17.39	103.7	N.A.	N.A.	Kutanan_et_al_2021	kin
BRU111	Bru	MSEA	Thailand	modern	0	Austroasiatic	17.39	103.7	N.A.	N.A.	Kutanan_et_al_2021	PASS
BRU113	Bru	MSEA	Thailand	modern	0	Austroasiatic	17.39	103.7	N.A.	N.A.	Kutanan_et_al_2021	kin
BRU115	Bru	MSEA	Thailand	modern	0	Austroasiatic	17.39	103.7	N.A.	N.A.	Kutanan_et_al_2021	PASS

BRU117	Bru	MSEA	Thailand	modern	0	Austroasiatic	17.39	103.7	N.A.	N.A.	Kutanan_et_al_2021	PASS
BRU119	Bru	MSEA	Thailand	modern	0	Austroasiatic	17.39	103.7	N.A.	N.A.	Kutanan_et_al_2021	PASS
BRU121	Bru	MSEA	Thailand	modern	0	Austroasiatic	17.39	103.7	N.A.	N.A.	Kutanan_et_al_2021	PASS
BRU123	Bru	MSEA	Thailand	modern	0	Austroasiatic	17.39	103.7	N.A.	N.A.	Kutanan_et_al_2021	PASS
BRU125	Bru	MSEA	Thailand	modern	0	Austroasiatic	17.39	103.7	N.A.	N.A.	Kutanan_et_al_2021	PASS
BRU127	Bru	MSEA	Thailand	modern	0	Austroasiatic	17.39	103.7	N.A.	N.A.	Kutanan_et_al_2021	PASS
BRU129	Bru	MSEA	Thailand	modern	0	Austroasiatic	17.39	103.7	N.A.	N.A.	Kutanan_et_al_2021	PASS
MO103	Mon	MSEA	Thailand	modern	0	Austroasiatic	14.69	102.06	N.A.	N.A.	Kutanan_et_al_2021	PASS
MO105	Mon	MSEA	Thailand	modern	0	Austroasiatic	14.69	102.06	N.A.	N.A.	Kutanan_et_al_2021	PASS
MO109	Mon	MSEA	Thailand	modern	0	Austroasiatic	14.69	102.06	N.A.	N.A.	Kutanan_et_al_2021	PASS
MO113	Mon	MSEA	Thailand	modern	0	Austroasiatic	14.69	102.06	N.A.	N.A.	Kutanan_et_al_2021	PASS
MO115	Mon	MSEA	Thailand	modern	0	Austroasiatic	14.69	102.06	N.A.	N.A.	Kutanan_et_al_2021	PASS
MO117	Mon	MSEA	Thailand	modern	0	Austroasiatic	14.69	102.06	N.A.	N.A.	Kutanan_et_al_2021	PASS
MO119	Mon	MSEA	Thailand	modern	0	Austroasiatic	14.69	102.06	N.A.	N.A.	Kutanan_et_al_2021	PASS
MO121	Mon	MSEA	Thailand	modern	0	Austroasiatic	14.69	102.06	N.A.	N.A.	Kutanan_et_al_2021	PASS
MO125	Mon	MSEA	Thailand	modern	0	Austroasiatic	14.69	102.06	N.A.	N.A.	Kutanan_et_al_2021	PASS
MO127	Mon	MSEA	Thailand	modern	0	Austroasiatic	14.69	102.06	N.A.	N.A.	Kutanan_et_al_2021	PASS
MO129	Mon	MSEA	Thailand	modern	0	Austroasiatic	14.69	102.06	N.A.	N.A.	Kutanan_et_al_2021	PASS
MO135	Mon	MSEA	Thailand	modern	0	Austroasiatic	14.69	102.06	N.A.	N.A.	Kutanan_et_al_2021	PASS
MO139	Mon	MSEA	Thailand	modern	0	Austroasiatic	14.69	102.06	N.A.	N.A.	Kutanan_et_al_2021	PASS
MO141	Mon	MSEA	Thailand	modern	0	Austroasiatic	14.69	102.06	N.A.	N.A.	Kutanan_et_al_2021	PASS
MO143	Mon	MSEA	Thailand	modern	0	Austroasiatic	14.69	102.06	N.A.	N.A.	Kutanan_et_al_2021	PASS
MO301	Mon	MSEA	Thailand	modern	0	Austroasiatic	15.3	98.38	N.A.	N.A.	Kutanan_et_al_2021	PASS
MO303	Mon	MSEA	Thailand	modern	0	Austroasiatic	15.3	98.38	N.A.	N.A.	Kutanan_et_al_2021	PASS
MO305	Mon	MSEA	Thailand	modern	0	Austroasiatic	15.3	98.38	N.A.	N.A.	Kutanan_et_al_2021	PASS
MO307	Mon	MSEA	Thailand	modern	0	Austroasiatic	15.3	98.38	N.A.	N.A.	Kutanan_et_al_2021	PASS
MO309	Mon	MSEA	Thailand	modern	0	Austroasiatic	15.3	98.38	N.A.	N.A.	Kutanan_et_al_2021	PASS
MO311	Mon	MSEA	Thailand	modern	0	Austroasiatic	15.3	98.38	N.A.	N.A.	Kutanan_et_al_2021	PASS
MO313	Mon	MSEA	Thailand	modern	0	Austroasiatic	15.3	98.38	N.A.	N.A.	Kutanan_et_al_2021	PASS
MO315	Mon	MSEA	Thailand	modern	0	Austroasiatic	15.3	98.38	N.A.	N.A.	Kutanan_et_al_2021	PASS
MO317	Mon	MSEA	Thailand	modern	0	Austroasiatic	15.3	98.38	N.A.	N.A.	Kutanan_et_al_2021	PASS
MO319	Mon	MSEA	Thailand	modern	0	Austroasiatic	15.3	98.38	N.A.	N.A.	Kutanan_et_al_2021	PASS
MO701	Mon	MSEA	Thailand	modern	0	Austroasiatic	13.58	100.1	N.A.	N.A.	Kutanan_et_al_2021	PASS
MO703	Mon	MSEA	Thailand	modern	0	Austroasiatic	13.58	100.1	N.A.	N.A.	Kutanan_et_al_2021	PASS
MO705	Mon	MSEA	Thailand	modern	0	Austroasiatic	13.58	100.1	N.A.	N.A.	Kutanan_et_al_2021	PASS
MO707	Mon	MSEA	Thailand	modern	0	Austroasiatic	13.58	100.1	N.A.	N.A.	Kutanan_et_al_2021	PASS
MO709	Mon	MSEA	Thailand	modern	0	Austroasiatic	13.58	100.1	N.A.	N.A.	Kutanan_et_al_2021	PASS
MO711	Mon	MSEA	Thailand	modern	0	Austroasiatic	13.58	100.1	N.A.	N.A.	Kutanan_et_al_2021	PASS
MO713	Mon	MSEA	Thailand	modern	0	Austroasiatic	13.58	100.1	N.A.	N.A.	Kutanan_et_al_2021	PASS
MO715	Mon	MSEA	Thailand	modern	0	Austroasiatic	13.58	100.1	N.A.	N.A.	Kutanan_et_al_2021	PASS
MO717	Mon	MSEA	Thailand	modern	0	Austroasiatic	13.58	100.1	N.A.	N.A.	Kutanan_et_al_2021	kin
MO719	Mon	MSEA	Thailand	modern	0	Austroasiatic	13.58	100.1	N.A.	N.A.	Kutanan_et_al_2021	PASS
RM101	Mon	MSEA	Thailand	modern	0	Austroasiatic	13.77	99.85	N.A.	N.A.	Kutanan_et_al_2021	PASS
RM103	Mon	MSEA	Thailand	modern	0	Austroasiatic	13.77	99.85	N.A.	N.A.	Kutanan_et_al_2021	PASS

RM105	Mon	MSEA	Thailand	modern	0	Austroasiatic	13.77	99.85	N.A.	N.A.	Kutanan_et_al_2021	PASS
RM107	Mon	MSEA	Thailand	modern	0	Austroasiatic	13.77	99.85	N.A.	N.A.	Kutanan_et_al_2021	PASS
RM109	Mon	MSEA	Thailand	modern	0	Austroasiatic	13.77	99.85	N.A.	N.A.	Kutanan_et_al_2021	PASS
RM110	Mon	MSEA	Thailand	modern	0	Austroasiatic	13.77	99.85	N.A.	N.A.	Kutanan_et_al_2021	PASS
RM111	Mon	MSEA	Thailand	modern	0	Austroasiatic	13.77	99.85	N.A.	N.A.	Kutanan_et_al_2021	PASS
RM112	Mon	MSEA	Thailand	modern	0	Austroasiatic	13.77	99.85	N.A.	N.A.	Kutanan_et_al_2021	PASS
RM115	Mon	MSEA	Thailand	modern	0	Austroasiatic	13.77	99.85	N.A.	N.A.	Kutanan_et_al_2021	PASS
RM117	Mon	MSEA	Thailand	modern	0	Austroasiatic	13.77	99.85	N.A.	N.A.	Kutanan_et_al_2021	PASS
RM119	Mon	MSEA	Thailand	modern	0	Austroasiatic	13.77	99.85	N.A.	N.A.	Kutanan_et_al_2021	PASS
RM121	Mon	MSEA	Thailand	modern	0	Austroasiatic	13.77	99.85	N.A.	N.A.	Kutanan_et_al_2021	PASS
RM123	Mon	MSEA	Thailand	modern	0	Austroasiatic	13.77	99.85	N.A.	N.A.	Kutanan_et_al_2021	PASS
RM127	Mon	MSEA	Thailand	modern	0	Austroasiatic	13.77	99.85	N.A.	N.A.	Kutanan_et_al_2021	PASS
RM129	Mon	MSEA	Thailand	modern	0	Austroasiatic	13.77	99.85	N.A.	N.A.	Kutanan_et_al_2021	PASS
RY112	Yuan	MSEA	Thailand	modern	0	Tai-Kadai	13.54	99.83	N_TK	N.A.	Kutanan_et_al_2021	PASS
RY130	Yuan	MSEA	Thailand	modern	0	Tai-Kadai	13.54	99.83	N_TK	N.A.	Kutanan_et_al_2021	PASS
RY132	Yuan	MSEA	Thailand	modern	0	Tai-Kadai	13.54	99.83	N_TK	N.A.	Kutanan_et_al_2021	PASS
RY136	Yuan	MSEA	Thailand	modern	0	Tai-Kadai	13.54	99.83	N_TK	N.A.	Kutanan_et_al_2021	PASS
RY137	Yuan	MSEA	Thailand	modern	0	Tai-Kadai	13.54	99.83	N_TK	N.A.	Kutanan_et_al_2021	PASS
RY139	Yuan	MSEA	Thailand	modern	0	Tai-Kadai	13.54	99.83	N_TK	N.A.	Kutanan_et_al_2021	PASS
RY141	Yuan	MSEA	Thailand	modern	0	Tai-Kadai	13.54	99.83	N_TK	N.A.	Kutanan_et_al_2021	PASS
RY143	Yuan	MSEA	Thailand	modern	0	Tai-Kadai	13.54	99.83	N_TK	N.A.	Kutanan_et_al_2021	PASS
TK102	Khuen	MSEA	Thailand	modern	0	Tai-Kadai	18.38	98.51	N_TK	N.A.	Kutanan_et_al_2021	PASS
TK104	Khuen	MSEA	Thailand	modern	0	Tai-Kadai	18.38	98.51	N_TK	N.A.	Kutanan_et_al_2021	PASS
TK112	Khuen	MSEA	Thailand	modern	0	Tai-Kadai	18.38	98.51	N_TK	N.A.	Kutanan_et_al_2021	PASS
TK114	Khuen	MSEA	Thailand	modern	0	Tai-Kadai	18.38	98.51	N_TK	N.A.	Kutanan_et_al_2021	kin
TK118	Khuen	MSEA	Thailand	modern	0	Tai-Kadai	18.38	98.51	N_TK	N.A.	Kutanan_et_al_2021	PASS
TK119	Khuen	MSEA	Thailand	modern	0	Tai-Kadai	18.38	98.51	N_TK	N.A.	Kutanan_et_al_2021	PASS
TK120	Khuen	MSEA	Thailand	modern	0	Tai-Kadai	18.38	98.51	N_TK	N.A.	Kutanan_et_al_2021	PASS
TK121	Khuen	MSEA	Thailand	modern	0	Tai-Kadai	18.38	98.51	N_TK	N.A.	Kutanan_et_al_2021	PASS
TK127	Khuen	MSEA	Thailand	modern	0	Tai-Kadai	18.38	98.51	N_TK	N.A.	Kutanan_et_al_2021	PASS
TK139	Khuen	MSEA	Thailand	modern	0	Tai-Kadai	18.38	98.51	N_TK	N.A.	Kutanan_et_al_2021	PASS
STP101	Phuan	MSEA	Thailand	modern	0	Tai-Kadai	17.51	99.77	N_TK	N.A.	Kutanan_et_al_2021	PASS
STP103	Phuan	MSEA	Thailand	modern	0	Tai-Kadai	17.51	99.77	N_TK	N.A.	Kutanan_et_al_2021	PASS
STP106	Phuan	MSEA	Thailand	modern	0	Tai-Kadai	17.51	99.77	N_TK	N.A.	Kutanan_et_al_2021	PASS
STP107	Phuan	MSEA	Thailand	modern	0	Tai-Kadai	17.51	99.77	N_TK	N.A.	Kutanan_et_al_2021	PASS
STP111	Phuan	MSEA	Thailand	modern	0	Tai-Kadai	17.51	99.77	N_TK	N.A.	Kutanan_et_al_2021	PASS
STP113	Phuan	MSEA	Thailand	modern	0	Tai-Kadai	17.51	99.77	N_TK	N.A.	Kutanan_et_al_2021	PASS
STP115	Phuan	MSEA	Thailand	modern	0	Tai-Kadai	17.51	99.77	N_TK	N.A.	Kutanan_et_al_2021	PASS
STP117	Phuan	MSEA	Thailand	modern	0	Tai-Kadai	17.51	99.77	N_TK	N.A.	Kutanan_et_al_2021	PASS
STP121	Phuan	MSEA	Thailand	modern	0	Tai-Kadai	17.51	99.77	N_TK	N.A.	Kutanan_et_al_2021	PASS
STP123	Phuan	MSEA	Thailand	modern	0	Tai-Kadai	17.51	99.77	N_TK	N.A.	Kutanan_et_al_2021	PASS
YA102	Shan	MSEA	Thailand	modern	0	Tai-Kadai	19.37	98.14	N_TK	N.A.	Kutanan_et_al_2021	PASS
YA107	Shan	MSEA	Thailand	modern	0	Tai-Kadai	19.37	98.14	N_TK	N.A.	Kutanan_et_al_2021	PASS
YA108	Shan	MSEA	Thailand	modern	0	Tai-Kadai	19.37	98.14	N_TK	N.A.	Kutanan_et_al_2021	PASS

YA111	Shan	MSEA	Thailand	modern	0	Tai-Kadai	19.37	98.14	N_TK	N.A.	Kutanan_et_al_2021	PASS
YA113	Shan	MSEA	Thailand	modern	0	Tai-Kadai	19.37	98.14	N_TK	N.A.	Kutanan_et_al_2021	PASS
YA115	Shan	MSEA	Thailand	modern	0	Tai-Kadai	19.37	98.14	N_TK	N.A.	Kutanan_et_al_2021	PASS
YA117	Shan	MSEA	Thailand	modern	0	Tai-Kadai	19.37	98.14	N_TK	N.A.	Kutanan_et_al_2021	PASS
YA118	Shan	MSEA	Thailand	modern	0	Tai-Kadai	19.37	98.14	N_TK	N.A.	Kutanan_et_al_2021	kin
YA121	Shan	MSEA	Thailand	modern	0	Tai-Kadai	19.37	98.14	N_TK	N.A.	Kutanan_et_al_2021	PASS
YA122	Shan	MSEA	Thailand	modern	0	Tai-Kadai	19.37	98.14	N_TK	N.A.	Kutanan_et_al_2021	PASS
A1YU101	Khonmueang	MSEA	Thailand	modern	0	Tai-Kadai	19.58	99.51	N_TK	N.A.	Kutanan_et_al_2021	PASS
A1YU105	Khonmueang	MSEA	Thailand	modern	0	Tai-Kadai	19.58	99.51	N_TK	N.A.	Kutanan_et_al_2021	PASS
A1YU107	Khonmueang	MSEA	Thailand	modern	0	Tai-Kadai	19.58	99.51	N_TK	N.A.	Kutanan_et_al_2021	PASS
A1YU111	Khonmueang	MSEA	Thailand	modern	0	Tai-Kadai	19.58	99.51	N_TK	N.A.	Kutanan_et_al_2021	PASS
A1YU113	Khonmueang	MSEA	Thailand	modern	0	Tai-Kadai	19.58	99.51	N_TK	N.A.	Kutanan_et_al_2021	PASS
A1YU115	Khonmueang	MSEA	Thailand	modern	0	Tai-Kadai	19.58	99.51	N_TK	N.A.	Kutanan_et_al_2021	PASS
A1YU125	Khonmueang	MSEA	Thailand	modern	0	Tai-Kadai	19.58	99.51	N_TK	N.A.	Kutanan_et_al_2021	PASS
A1YU129	Khonmueang	MSEA	Thailand	modern	0	Tai-Kadai	19.58	99.51	N_TK	N.A.	Kutanan_et_al_2021	PASS
A1YU131	Khonmueang	MSEA	Thailand	modern	0	Tai-Kadai	19.58	99.51	N_TK	N.A.	Kutanan_et_al_2021	PASS
A1YU132	Khonmueang	MSEA	Thailand	modern	0	Tai-Kadai	19.58	99.51	N_TK	N.A.	Kutanan_et_al_2021	PASS
A2YU301	Khonmueang	MSEA	Thailand	modern	0	Tai-Kadai	19.02	98.58	N_TK	N.A.	Kutanan_et_al_2021	PASS
A2YU303	Khonmueang	MSEA	Thailand	modern	0	Tai-Kadai	19.02	98.58	N_TK	N.A.	Kutanan_et_al_2021	PASS
A2YU305	Khonmueang	MSEA	Thailand	modern	0	Tai-Kadai	19.02	98.58	N_TK	N.A.	Kutanan_et_al_2021	PASS
A2YU307	Khonmueang	MSEA	Thailand	modern	0	Tai-Kadai	19.02	98.58	N_TK	N.A.	Kutanan_et_al_2021	PASS
A2YU313	Khonmueang	MSEA	Thailand	modern	0	Tai-Kadai	19.02	98.58	N_TK	N.A.	Kutanan_et_al_2021	PASS
A2YU321	Khonmueang	MSEA	Thailand	modern	0	Tai-Kadai	19.02	98.58	N_TK	N.A.	Kutanan_et_al_2021	PASS
A2YU329	Khonmueang	MSEA	Thailand	modern	0	Tai-Kadai	19.02	98.58	N_TK	N.A.	Kutanan_et_al_2021	PASS
A2YU331	Khonmueang	MSEA	Thailand	modern	0	Tai-Kadai	19.02	98.58	N_TK	N.A.	Kutanan_et_al_2021	PASS
A2YU337	Khonmueang	MSEA	Thailand	modern	0	Tai-Kadai	19.02	98.58	N_TK	N.A.	Kutanan_et_al_2021	PASS
A2YU353	Khonmueang	MSEA	Thailand	modern	0	Tai-Kadai	19.02	98.58	N_TK	N.A.	Kutanan_et_al_2021	PASS
TL114	Lue	MSEA	Thailand	modern	0	Tai-Kadai	19.09	100.56	N_TK	N.A.	Kutanan_et_al_2021	PASS
TL116	Lue	MSEA	Thailand	modern	0	Tai-Kadai	19.09	100.56	N_TK	N.A.	Kutanan_et_al_2021	PASS
TL118	Lue	MSEA	Thailand	modern	0	Tai-Kadai	19.09	100.56	N_TK	N.A.	Kutanan_et_al_2021	PASS
TL120	Lue	MSEA	Thailand	modern	0	Tai-Kadai	19.09	100.56	N_TK	N.A.	Kutanan_et_al_2021	PASS
TL128	Lue	MSEA	Thailand	modern	0	Tai-Kadai	19.09	100.56	N_TK	N.A.	Kutanan_et_al_2021	PASS
TL148	Lue	MSEA	Thailand	modern	0	Tai-Kadai	19.09	100.56	N_TK	N.A.	Kutanan_et_al_2021	PASS
TL152	Lue	MSEA	Thailand	modern	0	Tai-Kadai	19.09	100.56	N_TK	N.A.	Kutanan_et_al_2021	PASS
TL160	Lue	MSEA	Thailand	modern	0	Tai-Kadai	19.09	100.56	N_TK	N.A.	Kutanan_et_al_2021	PASS
TL166	Lue	MSEA	Thailand	modern	0	Tai-Kadai	19.09	100.56	N_TK	N.A.	Kutanan_et_al_2021	PASS
TL170	Lue	MSEA	Thailand	modern	0	Tai-Kadai	19.09	100.56	N_TK	N.A.	Kutanan_et_al_2021	kin
BTA101	BlackTai	MSEA	Thailand	modern	0	Tai-Kadai	17.79	101.73	NE_TK	N.A.	Kutanan_et_al_2021	PASS
BTA103	BlackTai	MSEA	Thailand	modern	0	Tai-Kadai	17.79	101.73	NE_TK	N.A.	Kutanan_et_al_2021	PASS
BTA105	BlackTai	MSEA	Thailand	modern	0	Tai-Kadai	17.79	101.73	NE_TK	N.A.	Kutanan_et_al_2021	PASS
BTA107	BlackTai	MSEA	Thailand	modern	0	Tai-Kadai	17.79	101.73	NE_TK	N.A.	Kutanan_et_al_2021	PASS
BTA111	BlackTai	MSEA	Thailand	modern	0	Tai-Kadai	17.79	101.73	NE_TK	N.A.	Kutanan_et_al_2021	PASS
BTA113	BlackTai	MSEA	Thailand	modern	0	Tai-Kadai	17.79	101.73	NE_TK	N.A.	Kutanan_et_al_2021	PASS
BTA115	BlackTai	MSEA	Thailand	modern	0	Tai-Kadai	17.79	101.73	NE_TK	N.A.	Kutanan_et_al_2021	PASS

BTA117	BlackTai	MSEA	Thailand	modern	0	Tai-Kadai	17.79	101.73	NE_TK	N.A.	Kutanan_et_al_2021	PASS
BTA119	BlackTai	MSEA	Thailand	modern	0	Tai-Kadai	17.79	101.73	NE_TK	N.A.	Kutanan_et_al_2021	PASS
LUA101	Laotian	MSEA	Laos	modern	0	Tai-Kadai	19.84	102.16	NE_TK	N.A.	Kutanan_et_al_2021	PASS
LUA105	Laotian	MSEA	Laos	modern	0	Tai-Kadai	19.84	102.16	NE_TK	N.A.	Kutanan_et_al_2021	PASS
LUA107	Laotian	MSEA	Laos	modern	0	Tai-Kadai	19.84	102.16	NE_TK	N.A.	Kutanan_et_al_2021	PASS
LUA109	Laotian	MSEA	Laos	modern	0	Tai-Kadai	19.84	102.16	NE_TK	N.A.	Kutanan_et_al_2021	PASS
LUA111	Laotian	MSEA	Laos	modern	0	Tai-Kadai	19.84	102.16	NE_TK	N.A.	Kutanan_et_al_2021	PASS
LUA113	Laotian	MSEA	Laos	modern	0	Tai-Kadai	19.84	102.16	NE_TK	N.A.	Kutanan_et_al_2021	PASS
LUA115	Laotian	MSEA	Laos	modern	0	Tai-Kadai	19.84	102.16	NE_TK	N.A.	Kutanan_et_al_2021	PASS
LUA117	Laotian	MSEA	Laos	modern	0	Tai-Kadai	19.84	102.16	NE_TK	N.A.	Kutanan_et_al_2021	PASS
LUA119	Laotian	MSEA	Laos	modern	0	Tai-Kadai	19.84	102.16	NE_TK	N.A.	Kutanan_et_al_2021	PASS
LUA121	Laotian	MSEA	Laos	modern	0	Tai-Kadai	19.84	102.16	NE_TK	N.A.	Kutanan_et_al_2021	PASS
LUA123	Laotian	MSEA	Laos	modern	0	Tai-Kadai	19.84	102.16	NE_TK	N.A.	Kutanan_et_al_2021	PASS
LUA125	Laotian	MSEA	Laos	modern	0	Tai-Kadai	19.84	102.16	NE_TK	N.A.	Kutanan_et_al_2021	PASS
LUA127	Laotian	MSEA	Laos	modern	0	Tai-Kadai	19.84	102.16	NE_TK	N.A.	Kutanan_et_al_2021	PASS
LUA129	Laotian	MSEA	Laos	modern	0	Tai-Kadai	19.84	102.16	NE_TK	N.A.	Kutanan_et_al_2021	PASS
LUA131	Laotian	MSEA	Laos	modern	0	Tai-Kadai	19.84	102.16	NE_TK	N.A.	Kutanan_et_al_2021	PASS
VIE126	Laotian	MSEA	Laos	modern	0	Tai-Kadai	17.97	102.59	NE_TK	N.A.	Kutanan_et_al_2021	PASS
VIE128	Laotian	MSEA	Laos	modern	0	Tai-Kadai	17.97	102.59	NE_TK	N.A.	Kutanan_et_al_2021	PASS
VIE136	Laotian	MSEA	Laos	modern	0	Tai-Kadai	17.97	102.59	NE_TK	N.A.	Kutanan_et_al_2021	PASS
VIE138	Laotian	MSEA	Laos	modern	0	Tai-Kadai	17.97	102.59	NE_TK	N.A.	Kutanan_et_al_2021	PASS
VIE140	Laotian	MSEA	Laos	modern	0	Tai-Kadai	17.97	102.59	NE_TK	N.A.	Kutanan_et_al_2021	PASS
VIE142	Laotian	MSEA	Laos	modern	0	Tai-Kadai	17.97	102.59	NE_TK	N.A.	Kutanan_et_al_2021	PASS
VIE150	Laotian	MSEA	Laos	modern	0	Tai-Kadai	17.97	102.59	NE_TK	N.A.	Kutanan_et_al_2021	PASS
VIE152	Laotian	MSEA	Laos	modern	0	Tai-Kadai	17.97	102.59	NE_TK	N.A.	Kutanan_et_al_2021	PASS
VIE154	Laotian	MSEA	Laos	modern	0	Tai-Kadai	17.97	102.59	NE_TK	N.A.	Kutanan_et_al_2021	PASS
VIE156	Laotian	MSEA	Laos	modern	0	Tai-Kadai	17.97	102.59	NE_TK	N.A.	Kutanan_et_al_2021	PASS
VIE158	Laotian	MSEA	Laos	modern	0	Tai-Kadai	17.97	102.59	NE_TK	N.A.	Kutanan_et_al_2021	PASS
VIE162	Laotian	MSEA	Laos	modern	0	Tai-Kadai	17.97	102.59	NE_TK	N.A.	Kutanan_et_al_2021	PASS
VIE164	Laotian	MSEA	Laos	modern	0	Tai-Kadai	17.97	102.59	NE_TK	N.A.	Kutanan_et_al_2021	PASS
VIE168	Laotian	MSEA	Laos	modern	0	Tai-Kadai	17.97	102.59	NE_TK	N.A.	Kutanan_et_al_2021	PASS
VIE170	Laotian	MSEA	Laos	modern	0	Tai-Kadai	17.97	102.59	NE_TK	N.A.	Kutanan_et_al_2021	PASS
LAO101	LaoIsan	MSEA	Thailand	modern	0	Tai-Kadai	15.62	103.5	NE_TK	N.A.	Kutanan_et_al_2021	PASS
LAO103	LaoIsan	MSEA	Thailand	modern	0	Tai-Kadai	15.62	103.5	NE_TK	N.A.	Kutanan_et_al_2021	PASS
LAO105	LaoIsan	MSEA	Thailand	modern	0	Tai-Kadai	15.62	103.5	NE_TK	N.A.	Kutanan_et_al_2021	PASS
LAO107	LaoIsan	MSEA	Thailand	modern	0	Tai-Kadai	15.62	103.5	NE_TK	N.A.	Kutanan_et_al_2021	PASS
LAO109	LaoIsan	MSEA	Thailand	modern	0	Tai-Kadai	15.62	103.5	NE_TK	N.A.	Kutanan_et_al_2021	PASS
LAO111	LaoIsan	MSEA	Thailand	modern	0	Tai-Kadai	15.62	103.5	NE_TK	N.A.	Kutanan_et_al_2021	PASS
LAO113	LaoIsan	MSEA	Thailand	modern	0	Tai-Kadai	15.62	103.5	NE_TK	N.A.	Kutanan_et_al_2021	PASS
LAO115	LaoIsan	MSEA	Thailand	modern	0	Tai-Kadai	15.62	103.5	NE_TK	N.A.	Kutanan_et_al_2021	PASS
LAO117	LaoIsan	MSEA	Thailand	modern	0	Tai-Kadai	15.62	103.5	NE_TK	N.A.	Kutanan_et_al_2021	PASS
LAO119	LaoIsan	MSEA	Thailand	modern	0	Tai-Kadai	15.62	103.5	NE_TK	N.A.	Kutanan_et_al_2021	PASS
LAO401	LaoIsan	MSEA	Thailand	modern	0	Tai-Kadai	15.81	102.03	NE_TK	N.A.	Kutanan_et_al_2021	PASS
LAO403	LaoIsan	MSEA	Thailand	modern	0	Tai-Kadai	15.81	102.03	NE_TK	N.A.	Kutanan_et_al_2021	PASS

LAO405	LaoIsan	MSEA	Thailand	modern	0	Tai-Kadai	15.81	102.03	NE_TK	N.A.	Kutanan_et_al_2021	PASS
LAO407	LaoIsan	MSEA	Thailand	modern	0	Tai-Kadai	15.81	102.03	NE_TK	N.A.	Kutanan_et_al_2021	PASS
LAO409	LaoIsan	MSEA	Thailand	modern	0	Tai-Kadai	15.81	102.03	NE_TK	N.A.	Kutanan_et_al_2021	PASS
LAO411	LaoIsan	MSEA	Thailand	modern	0	Tai-Kadai	15.81	102.03	NE_TK	N.A.	Kutanan_et_al_2021	PASS
LAO413	LaoIsan	MSEA	Thailand	modern	0	Tai-Kadai	15.81	102.03	NE_TK	N.A.	Kutanan_et_al_2021	PASS
LAO417	LaoIsan	MSEA	Thailand	modern	0	Tai-Kadai	15.81	102.03	NE_TK	N.A.	Kutanan_et_al_2021	PASS
LAO421	LaoIsan	MSEA	Thailand	modern	0	Tai-Kadai	15.81	102.03	NE_TK	N.A.	Kutanan_et_al_2021	PASS
LAO423	LaoIsan	MSEA	Thailand	modern	0	Tai-Kadai	15.81	102.03	NE_TK	N.A.	Kutanan_et_al_2021	PASS
PUT101	Phutai	MSEA	Thailand	modern	0	Tai-Kadai	17.28	103.65	NE_TK	N.A.	Kutanan_et_al_2021	PASS
PUT102	Phutai	MSEA	Thailand	modern	0	Tai-Kadai	17.28	103.65	NE_TK	N.A.	Kutanan_et_al_2021	PASS
PUT103	Phutai	MSEA	Thailand	modern	0	Tai-Kadai	17.28	103.65	NE_TK	N.A.	Kutanan_et_al_2021	PASS
PUT105	Phutai	MSEA	Thailand	modern	0	Tai-Kadai	17.28	103.65	NE_TK	N.A.	Kutanan_et_al_2021	PASS
PUT109	Phutai	MSEA	Thailand	modern	0	Tai-Kadai	17.28	103.65	NE_TK	N.A.	Kutanan_et_al_2021	PASS
PUT110	Phutai	MSEA	Thailand	modern	0	Tai-Kadai	17.28	103.65	NE_TK	N.A.	Kutanan_et_al_2021	PASS
PUT111	Phutai	MSEA	Thailand	modern	0	Tai-Kadai	17.28	103.65	NE_TK	N.A.	Kutanan_et_al_2021	PASS
PUT112	Phutai	MSEA	Thailand	modern	0	Tai-Kadai	17.28	103.65	NE_TK	N.A.	Kutanan_et_al_2021	PASS
PUT113	Phutai	MSEA	Thailand	modern	0	Tai-Kadai	17.28	103.65	NE_TK	N.A.	Kutanan_et_al_2021	PASS
PUT114	Phutai	MSEA	Thailand	modern	0	Tai-Kadai	17.28	103.65	NE_TK	N.A.	Kutanan_et_al_2021	PASS
YOH103	Nyaw	MSEA	Thailand	modern	0	Tai-Kadai	17.55	104.09	NE_TK	N.A.	Kutanan_et_al_2021	PASS
YOH105	Nyaw	MSEA	Thailand	modern	0	Tai-Kadai	17.55	104.09	NE_TK	N.A.	Kutanan_et_al_2021	PASS
YOH107	Nyaw	MSEA	Thailand	modern	0	Tai-Kadai	17.55	104.09	NE_TK	N.A.	Kutanan_et_al_2021	PASS
YOH109	Nyaw	MSEA	Thailand	modern	0	Tai-Kadai	17.55	104.09	NE_TK	N.A.	Kutanan_et_al_2021	PASS
YOH111	Nyaw	MSEA	Thailand	modern	0	Tai-Kadai	17.55	104.09	NE_TK	N.A.	Kutanan_et_al_2021	PASS
YOH113	Nyaw	MSEA	Thailand	modern	0	Tai-Kadai	17.55	104.09	NE_TK	N.A.	Kutanan_et_al_2021	PASS
YOH115	Nyaw	MSEA	Thailand	modern	0	Tai-Kadai	17.55	104.09	NE_TK	N.A.	Kutanan_et_al_2021	PASS
YOH117	Nyaw	MSEA	Thailand	modern	0	Tai-Kadai	17.55	104.09	NE_TK	N.A.	Kutanan_et_al_2021	PASS
YOH121	Nyaw	MSEA	Thailand	modern	0	Tai-Kadai	17.55	104.09	NE_TK	N.A.	Kutanan_et_al_2021	PASS
YOH123	Nyaw	MSEA	Thailand	modern	0	Tai-Kadai	17.55	104.09	NE_TK	N.A.	Kutanan_et_al_2021	PASS
SAK104	Seak	MSEA	Thailand	modern	0	Tai-Kadai	17.45	104.74	NE_TK	N.A.	Kutanan_et_al_2021	PASS
SAK105	Seak	MSEA	Thailand	modern	0	Tai-Kadai	17.45	104.74	NE_TK	N.A.	Kutanan_et_al_2021	kin
SAK108	Seak	MSEA	Thailand	modern	0	Tai-Kadai	17.45	104.74	NE_TK	N.A.	Kutanan_et_al_2021	PASS
SAK110	Seak	MSEA	Thailand	modern	0	Tai-Kadai	17.45	104.74	NE_TK	N.A.	Kutanan_et_al_2021	PASS
SAK111	Seak	MSEA	Thailand	modern	0	Tai-Kadai	17.45	104.74	NE_TK	N.A.	Kutanan_et_al_2021	PASS
SAK112	Seak	MSEA	Thailand	modern	0	Tai-Kadai	17.45	104.74	NE_TK	N.A.	Kutanan_et_al_2021	PASS
SAK114	Seak	MSEA	Thailand	modern	0	Tai-Kadai	17.45	104.74	NE_TK	N.A.	Kutanan_et_al_2021	PASS
SAK116	Seak	MSEA	Thailand	modern	0	Tai-Kadai	17.45	104.74	NE_TK	N.A.	Kutanan_et_al_2021	PASS
SAK118	Seak	MSEA	Thailand	modern	0	Tai-Kadai	17.45	104.74	NE_TK	N.A.	Kutanan_et_al_2021	PASS
SAK120	Seak	MSEA	Thailand	modern	0	Tai-Kadai	17.45	104.74	NE_TK	N.A.	Kutanan_et_al_2021	PASS
SAK126	Seak	MSEA	Thailand	modern	0	Tai-Kadai	17.45	104.74	NE_TK	N.A.	Kutanan_et_al_2021	PASS
SAK128	Seak	MSEA	Thailand	modern	0	Tai-Kadai	17.45	104.74	NE_TK	N.A.	Kutanan_et_al_2021	PASS
SAK130	Seak	MSEA	Thailand	modern	0	Tai-Kadai	17.45	104.74	NE_TK	N.A.	Kutanan_et_al_2021	PASS
SAK136	Seak	MSEA	Thailand	modern	0	Tai-Kadai	17.45	104.74	NE_TK	N.A.	Kutanan_et_al_2021	PASS
SAK138	Seak	MSEA	Thailand	modern	0	Tai-Kadai	17.45	104.74	NE_TK	N.A.	Kutanan_et_al_2021	PASS
KAL101	Kalueang	MSEA	Thailand	modern	0	Tai-Kadai	17.33	104.59	NE_TK	N.A.	Kutanan_et_al_2021	PASS

KAL102	Kalueang	MSEA	Thailand	modern	0	Tai-Kadai	17.33	104.59	NE_TK	N.A.	Kutanan_et_al_2021	PASS
KAL105	Kalueang	MSEA	Thailand	modern	0	Tai-Kadai	17.33	104.59	NE_TK	N.A.	Kutanan_et_al_2021	PASS
KAL107	Kalueang	MSEA	Thailand	modern	0	Tai-Kadai	17.33	104.59	NE_TK	N.A.	Kutanan_et_al_2021	PASS
KAL110	Kalueang	MSEA	Thailand	modern	0	Tai-Kadai	17.33	104.59	NE_TK	N.A.	Kutanan_et_al_2021	PASS
KAL112	Kalueang	MSEA	Thailand	modern	0	Tai-Kadai	17.33	104.59	NE_TK	N.A.	Kutanan_et_al_2021	PASS
KAL113	Kalueang	MSEA	Thailand	modern	0	Tai-Kadai	17.33	104.59	NE_TK	N.A.	Kutanan_et_al_2021	PASS
KAL115	Kalueang	MSEA	Thailand	modern	0	Tai-Kadai	17.33	104.59	NE_TK	N.A.	Kutanan_et_al_2021	PASS
KAL116	Kalueang	MSEA	Thailand	modern	0	Tai-Kadai	17.33	104.59	NE_TK	N.A.	Kutanan_et_al_2021	PASS
KAL118	Kalueang	MSEA	Thailand	modern	0	Tai-Kadai	17.33	104.59	NE_TK	N.A.	Kutanan_et_al_2021	PASS
CT201	CentralThai	MSEA	Thailand	modern	0	Tai-Kadai	13.71	99.77	C_TK	N.A.	Kutanan_et_al_2021	PASS
CT203	CentralThai	MSEA	Thailand	modern	0	Tai-Kadai	13.71	99.77	C_TK	N.A.	Kutanan_et_al_2021	PASS
CT205	CentralThai	MSEA	Thailand	modern	0	Tai-Kadai	13.71	99.77	C_TK	N.A.	Kutanan_et_al_2021	PASS
CT207	CentralThai	MSEA	Thailand	modern	0	Tai-Kadai	18.12	98.23	C_TK	N.A.	Kutanan_et_al_2021	PASS
CT209	CentralThai	MSEA	Thailand	modern	0	Tai-Kadai	13.71	99.77	C_TK	N.A.	Kutanan_et_al_2021	PASS
CT211	CentralThai	MSEA	Thailand	modern	0	Tai-Kadai	13.71	99.77	C_TK	N.A.	Kutanan_et_al_2021	PASS
CT213	CentralThai	MSEA	Thailand	modern	0	Tai-Kadai	13.71	99.77	C_TK	N.A.	Kutanan_et_al_2021	PASS
CT215	CentralThai	MSEA	Thailand	modern	0	Tai-Kadai	13.71	99.77	C_TK	N.A.	Kutanan_et_al_2021	PASS
CT217	CentralThai	MSEA	Thailand	modern	0	Tai-Kadai	13.71	99.77	C_TK	N.A.	Kutanan_et_al_2021	PASS
CT219	CentralThai	MSEA	Thailand	modern	0	Tai-Kadai	13.71	99.77	C_TK	N.A.	Kutanan_et_al_2021	PASS
CT301	CentralThai	MSEA	Thailand	modern	0	Tai-Kadai	13.74	100.07	C_TK	N.A.	Kutanan_et_al_2021	PASS
CT303	CentralThai	MSEA	Thailand	modern	0	Tai-Kadai	13.71	100.07	C_TK	N.A.	Kutanan_et_al_2021	PASS
CT305	CentralThai	MSEA	Thailand	modern	0	Tai-Kadai	13.74	100.07	C_TK	N.A.	Kutanan_et_al_2021	PASS
CT307	CentralThai	MSEA	Thailand	modern	0	Tai-Kadai	13.74	100.07	C_TK	N.A.	Kutanan_et_al_2021	PASS
CT309	CentralThai	MSEA	Thailand	modern	0	Tai-Kadai	13.74	100.07	C_TK	N.A.	Kutanan_et_al_2021	PASS
CT311	CentralThai	MSEA	Thailand	modern	0	Tai-Kadai	13.74	100.07	C_TK	N.A.	Kutanan_et_al_2021	PASS
CT313	CentralThai	MSEA	Thailand	modern	0	Tai-Kadai	13.74	100.07	C_TK	N.A.	Kutanan_et_al_2021	PASS
CT315	CentralThai	MSEA	Thailand	modern	0	Tai-Kadai	13.74	100.07	C_TK	N.A.	Kutanan_et_al_2021	PASS
CT317	CentralThai	MSEA	Thailand	modern	0	Tai-Kadai	13.74	100.07	C_TK	N.A.	Kutanan_et_al_2021	PASS
CT319	CentralThai	MSEA	Thailand	modern	0	Tai-Kadai	13.74	100.07	C_TK	N.A.	Kutanan_et_al_2021	PASS
BST101	SouthernThai_TK	MSEA	Thailand	modern	0	Tai-Kadai	8.31	100.05	S_TK	N.A.	Kutanan_et_al_2021	PASS
BST103	SouthernThai_TK	MSEA	Thailand	modern	0	Tai-Kadai	8.31	100.05	S_TK	N.A.	Kutanan_et_al_2021	PASS
BST105	SouthernThai_TK	MSEA	Thailand	modern	0	Tai-Kadai	8.31	100.05	S_TK	N.A.	Kutanan_et_al_2021	PASS
BST107	SouthernThai_TK	MSEA	Thailand	modern	0	Tai-Kadai	8.31	100.05	S_TK	N.A.	Kutanan_et_al_2021	PASS
BST109	SouthernThai_TK	MSEA	Thailand	modern	0	Tai-Kadai	8.31	100.05	S_TK	N.A.	Kutanan_et_al_2021	PASS
BST111	SouthernThai_TK	MSEA	Thailand	modern	0	Tai-Kadai	8.31	100.05	S_TK	N.A.	Kutanan_et_al_2021	PASS
BST113	SouthernThai_TK	MSEA	Thailand	modern	0	Tai-Kadai	8.31	100.05	S_TK	N.A.	Kutanan_et_al_2021	PASS
BST115	SouthernThai_TK	MSEA	Thailand	modern	0	Tai-Kadai	8.31	100.05	S_TK	N.A.	Kutanan_et_al_2021	PASS
BST117	SouthernThai_TK	MSEA	Thailand	modern	0	Tai-Kadai	8.31	100.05	S_TK	N.A.	Kutanan_et_al_2021	PASS
BST119	SouthernThai_TK	MSEA	Thailand	modern	0	Tai-Kadai	8.31	100.05	S_TK	N.A.	Kutanan_et_al_2021	PASS
BST121	SouthernThai_TK	MSEA	Thailand	modern	0	Tai-Kadai	8.31	100.05	S_TK	N.A.	Kutanan_et_al_2021	PASS
BST123	SouthernThai_TK	MSEA	Thailand	modern	0	Tai-Kadai	8.31	100.05	S_TK	N.A.	Kutanan_et_al_2021	PASS
BST125	SouthernThai_TK	MSEA	Thailand	modern	0	Tai-Kadai	8.31	100.05	S_TK	N.A.	Kutanan_et_al_2021	PASS
BST129	SouthernThai_TK	MSEA	Thailand	modern	0	Tai-Kadai	8.31	100.05	S_TK	N.A.	Kutanan_et_al_2021	PASS
BST131	SouthernThai_TK	MSEA	Thailand	modern	0	Tai-Kadai	8.31	100.05	S_TK	N.A.	Kutanan_et_al_2021	PASS

CHI007	Thai	MSEA	Thailand	modern	0	Tai-Kadai	13.8	100.5	Thai	N.A.	Lazaridis_et_al_2014	PASS
CHI034	Thai	MSEA	Thailand	modern	0	Tai-Kadai	13.8	100.5	Thai	N.A.	Lazaridis_et_al_2014	PASS
DCH002	Thai	MSEA	Thailand	modern	0	Tai-Kadai	13.8	100.5	Thai	N.A.	Lazaridis_et_al_2014	kin
DCH006	Thai	MSEA	Thailand	modern	0	Tai-Kadai	13.8	100.5	Thai	N.A.	Lazaridis_et_al_2014	PASS
DCH007	Thai	MSEA	Thailand	modern	0	Tai-Kadai	13.8	100.5	Thai	N.A.	Lazaridis_et_al_2014	PASS
DCH008	Thai	MSEA	Thailand	modern	0	Tai-Kadai	13.8	100.5	Thai	N.A.	Lazaridis_et_al_2014	PASS
DCH009	Thai	MSEA	Thailand	modern	0	Tai-Kadai	13.8	100.5	Thai	N.A.	Lazaridis_et_al_2014	PASS
DCH010	Thai	MSEA	Thailand	modern	0	Tai-Kadai	13.8	100.5	Thai	N.A.	Lazaridis_et_al_2014	PASS
DCH011	Thai	MSEA	Thailand	modern	0	Tai-Kadai	13.8	100.5	Thai	N.A.	Lazaridis_et_al_2014	kin
DCH012	Thai	MSEA	Thailand	modern	0	Tai-Kadai	13.8	100.5	Thai	N.A.	Lazaridis_et_al_2014	PASS
HGDP00449	Mbuti	Africa	Congo	modern	0	Central_Sudanic	1	29	N.A.	N.A.	Patterson_et_al_2012	PASS
HGDP00462	Mbuti	Africa	Congo	modern	0	Central_Sudanic	1	29	N.A.	N.A.	Patterson_et_al_2012	PASS
HGDP00463	Mbuti	Africa	Congo	modern	0	Central_Sudanic	1	29	N.A.	N.A.	Patterson_et_al_2012	PASS
HGDP00467	Mbuti	Africa	Congo	modern	0	Central_Sudanic	1	29	N.A.	N.A.	Patterson_et_al_2012	PASS
HGDP00474	Mbuti	Africa	Congo	modern	0	Central_Sudanic	1	29	N.A.	N.A.	Patterson_et_al_2012	PASS
HGDP00476	Mbuti	Africa	Congo	modern	0	Central_Sudanic	1	29	N.A.	N.A.	Patterson_et_al_2012	PASS
HGDP00478	Mbuti	Africa	Congo	modern	0	Central_Sudanic	1	29	N.A.	N.A.	Patterson_et_al_2012	PASS
HGDP00982	Mbuti	Africa	Congo	modern	0	Central_Sudanic	1	29	N.A.	N.A.	Patterson_et_al_2012	PASS
HGDP00984	Mbuti	Africa	Congo	modern	0	Central_Sudanic	1	29	N.A.	N.A.	Patterson_et_al_2012	PASS
HGDP01081	Mbuti	Africa	Congo	modern	0	Central_Sudanic	1	29	N.A.	N.A.	Patterson_et_al_2012	PASS
HGDP00511	French	Europe	France	modern	0	Indo-European	46	2	N.A.	N.A.	Patterson_et_al_2012	PASS
HGDP00512	French	Europe	France	modern	0	Indo-European	46	2	N.A.	N.A.	Patterson_et_al_2012	PASS
HGDP00513	French	Europe	France	modern	0	Indo-European	46	2	N.A.	N.A.	Patterson_et_al_2012	PASS
HGDP00514	French	Europe	France	modern	0	Indo-European	46	2	N.A.	N.A.	Patterson_et_al_2012	PASS
HGDP00515	French	Europe	France	modern	0	Indo-European	46	2	N.A.	N.A.	Patterson_et_al_2012	PASS
HGDP00516	French	Europe	France	modern	0	Indo-European	46	2	N.A.	N.A.	Patterson_et_al_2012	PASS
HGDP00517	French	Europe	France	modern	0	Indo-European	46	2	N.A.	N.A.	Patterson_et_al_2012	PASS
HGDP00518	French	Europe	France	modern	0	Indo-European	46	2	N.A.	N.A.	Patterson_et_al_2012	PASS
HGDP00519	French	Europe	France	modern	0	Indo-European	46	2	N.A.	N.A.	Patterson_et_al_2012	PASS
HGDP00522	French	Europe	France	modern	0	Indo-European	46	2	N.A.	N.A.	Patterson_et_al_2012	PASS
Tiwari1	Brahmin_Tiwari	SouthAsia	India	modern	0	Indo-European	21.9	83.4	N.A.	N.A.	Lazaridis_et_al_2014	PASS
Tiwari2	Brahmin_Tiwari	SouthAsia	India	modern	0	Indo-European	21.9	83.4	N.A.	N.A.	Lazaridis_et_al_2014	PASS
Tiwari3	Brahmin_Tiwari	SouthAsia	India	modern	0	Indo-European	21.9	83.4	N.A.	N.A.	Lazaridis_et_al_2014	PASS
Tiwari4	Brahmin_Tiwari	SouthAsia	India	modern	0	Indo-European	21.9	83.4	N.A.	N.A.	Lazaridis_et_al_2014	PASS
Tiwari5	Brahmin_Tiwari	SouthAsia	India	modern	0	Indo-European	21.9	83.4	N.A.	N.A.	Lazaridis_et_al_2014	PASS
Tiwari6	Brahmin_Tiwari	SouthAsia	India	modern	0	Indo-European	21.9	83.4	N.A.	N.A.	Lazaridis_et_al_2014	PASS
Tiwari7	Brahmin_Tiwari	SouthAsia	India	modern	0	Indo-European	21.9	83.4	N.A.	N.A.	Lazaridis_et_al_2014	PASS
Tiwari8	Brahmin_Tiwari	SouthAsia	India	modern	0	Indo-European	21.9	83.4	N.A.	N.A.	Lazaridis_et_al_2014	PASS
Tiwari9	Brahmin_Tiwari	SouthAsia	India	modern	0	Indo-European	21.9	83.4	N.A.	N.A.	Lazaridis_et_al_2014	PASS
Tiwari10	Brahmin_Tiwari	SouthAsia	India	modern	0	Indo-European	21.9	83.4	N.A.	N.A.	Lazaridis_et_al_2014	PASS
Tiwari11	Brahmin_Tiwari	SouthAsia	India	modern	0	Indo-European	21.9	83.4	N.A.	N.A.	Lazaridis_et_al_2014	PASS
Tiwari12	Brahmin_Tiwari	SouthAsia	India	modern	0	Indo-European	21.9	83.4	N.A.	N.A.	Lazaridis_et_al_2014	PASS
Tiwari13	Brahmin_Tiwari	SouthAsia	India	modern	0	Indo-European	21.9	83.4	N.A.	N.A.	Lazaridis_et_al_2014	PASS
Tiwari14	Brahmin_Tiwari	SouthAsia	India	modern	0	Indo-European	21.9	83.4	N.A.	N.A.	Lazaridis_et_al_2014	PASS

Tiwari15	Brahmin_Tiwari	SouthAsia	India	modern	0	Indo-European	21.9	83.4	N.A.	N.A.	Lazaridis_et_al_2014	PASS
NA20847	Gujarati	SouthAsia	India	modern	0	Indo-European	23.2	72.7	N.A.	N.A.	Lazaridis_et_al_2014	PASS
NA20854	Gujarati	SouthAsia	India	modern	0	Indo-European	23.2	72.7	N.A.	N.A.	Lazaridis_et_al_2014	PASS
NA20881	Gujarati	SouthAsia	India	modern	0	Indo-European	23.2	72.7	N.A.	N.A.	Lazaridis_et_al_2014	PASS
NA20882	Gujarati	SouthAsia	India	modern	0	Indo-European	23.2	72.7	N.A.	N.A.	Lazaridis_et_al_2014	PASS
NA20899	Gujarati	SouthAsia	India	modern	0	Indo-European	23.2	72.7	N.A.	N.A.	Lazaridis_et_al_2014	PASS
NA20910	Gujarati	SouthAsia	India	modern	0	Indo-European	23.2	72.7	N.A.	N.A.	Lazaridis_et_al_2014	PASS
NA21125	Gujarati	SouthAsia	India	modern	0	Indo-European	23.2	72.7	N.A.	N.A.	Lazaridis_et_al_2014	PASS
NA20858	Gujarati	SouthAsia	India	modern	0	Indo-European	23.2	72.7	N.A.	N.A.	Lazaridis_et_al_2014	PASS
NA20866	Gujarati	SouthAsia	India	modern	0	Indo-European	23.2	72.7	N.A.	N.A.	Lazaridis_et_al_2014	PASS
NA20873	Gujarati	SouthAsia	India	modern	0	Indo-European	23.2	72.7	N.A.	N.A.	Lazaridis_et_al_2014	PASS
NA20883	Gujarati	SouthAsia	India	modern	0	Indo-European	23.2	72.7	N.A.	N.A.	Lazaridis_et_al_2014	PASS
NA20885	Gujarati	SouthAsia	India	modern	0	Indo-European	23.2	72.7	N.A.	N.A.	Lazaridis_et_al_2014	PASS
NA20898	Gujarati	SouthAsia	India	modern	0	Indo-European	23.2	72.7	N.A.	N.A.	Lazaridis_et_al_2014	PASS
NA20903	Gujarati	SouthAsia	India	modern	0	Indo-European	23.2	72.7	N.A.	N.A.	Lazaridis_et_al_2014	PASS
NA20904	Gujarati	SouthAsia	India	modern	0	Indo-European	23.2	72.7	N.A.	N.A.	Lazaridis_et_al_2014	PASS
NA20911	Gujarati	SouthAsia	India	modern	0	Indo-European	23.2	72.7	N.A.	N.A.	Lazaridis_et_al_2014	PASS
NA21116	Gujarati	SouthAsia	India	modern	0	Indo-European	23.2	72.7	N.A.	N.A.	Lazaridis_et_al_2014	PASS
NA21123	Gujarati	SouthAsia	India	modern	0	Indo-European	23.2	72.7	N.A.	N.A.	Lazaridis_et_al_2014	PASS
NA21097	Gujarati	SouthAsia	India	modern	0	Indo-European	23.2	72.7	N.A.	N.A.	Lazaridis_et_al_2014	PASS
NA20862	Gujarati	SouthAsia	India	modern	0	Indo-European	23.2	72.7	N.A.	N.A.	Lazaridis_et_al_2014	PASS
Lodhi2	Lodhi	SouthAsia	India	modern	0	Indo-European	25.45	78.62	N.A.	N.A.	Lazaridis_et_al_2014	PASS
Lodhi3	Lodhi	SouthAsia	India	modern	0	Indo-European	25.45	78.62	N.A.	N.A.	Lazaridis_et_al_2014	PASS
Lodhi4	Lodhi	SouthAsia	India	modern	0	Indo-European	25.45	78.62	N.A.	N.A.	Lazaridis_et_al_2014	PASS
Lodhi5	Lodhi	SouthAsia	India	modern	0	Indo-European	25.45	78.62	N.A.	N.A.	Lazaridis_et_al_2014	PASS
Lodhi6	Lodhi	SouthAsia	India	modern	0	Indo-European	25.45	78.62	N.A.	N.A.	Lazaridis_et_al_2014	PASS
Lodhi7	Lodhi	SouthAsia	India	modern	0	Indo-European	25.45	78.62	N.A.	N.A.	Lazaridis_et_al_2014	PASS
Lodhi8	Lodhi	SouthAsia	India	modern	0	Indo-European	25.45	78.62	N.A.	N.A.	Lazaridis_et_al_2014	PASS
Lodhi9	Lodhi	SouthAsia	India	modern	0	Indo-European	25.45	78.62	N.A.	N.A.	Lazaridis_et_al_2014	PASS
Lodhi10	Lodhi	SouthAsia	India	modern	0	Indo-European	25.45	78.62	N.A.	N.A.	Lazaridis_et_al_2014	PASS
Lodhi11	Lodhi	SouthAsia	India	modern	0	Indo-European	25.45	78.62	N.A.	N.A.	Lazaridis_et_al_2014	PASS
Lodhi13	Lodhi	SouthAsia	India	modern	0	Indo-European	25.45	78.62	N.A.	N.A.	Lazaridis_et_al_2014	PASS
Lodhi14	Lodhi	SouthAsia	India	modern	0	Indo-European	25.45	78.62	N.A.	N.A.	Lazaridis_et_al_2014	PASS
Lodhi15	Lodhi	SouthAsia	India	modern	0	Indo-European	25.45	78.62	N.A.	N.A.	Lazaridis_et_al_2014	PASS
Vishwabrahmin1	Vishwabrahmin	SouthAsia	India	modern	0	Dravidian	16.3	80.48	N.A.	N.A.	Lazaridis_et_al_2014	PASS
Vishwabrahmin3	Vishwabrahmin	SouthAsia	India	modern	0	Dravidian	16.3	80.48	N.A.	N.A.	Lazaridis_et_al_2014	PASS
Vishwabrahmin4	Vishwabrahmin	SouthAsia	India	modern	0	Dravidian	16.3	80.48	N.A.	N.A.	Lazaridis_et_al_2014	PASS
Vishwabrahmin5	Vishwabrahmin	SouthAsia	India	modern	0	Dravidian	16.3	80.48	N.A.	N.A.	Lazaridis_et_al_2014	PASS
Vishwabrahmin6	Vishwabrahmin	SouthAsia	India	modern	0	Dravidian	16.3	80.48	N.A.	N.A.	Lazaridis_et_al_2014	PASS
Vishwabrahmin7	Vishwabrahmin	SouthAsia	India	modern	0	Dravidian	16.3	80.48	N.A.	N.A.	Lazaridis_et_al_2014	PASS
Vishwabrahmin8	Vishwabrahmin	SouthAsia	India	modern	0	Dravidian	16.3	80.48	N.A.	N.A.	Lazaridis_et_al_2014	PASS
Vishwabrahmin9	Vishwabrahmin	SouthAsia	India	modern	0	Dravidian	16.3	80.48	N.A.	N.A.	Lazaridis_et_al_2014	PASS
Vishwabrahmin10	Vishwabrahmin	SouthAsia	India	modern	0	Dravidian	16.3	80.48	N.A.	N.A.	Lazaridis_et_al_2014	PASS
Vishwabrahmin11	Vishwabrahmin	SouthAsia	India	modern	0	Dravidian	16.3	80.48	N.A.	N.A.	Lazaridis_et_al_2014	PASS

Vishwabrahmin12	Vishwabrahmin	SouthAsia	India	modern	0	Dravidian	16.3	80.48	N.A.	N.A.	Lazaridis_et_al_2014	PASS
Vishwabrahmin13	Vishwabrahmin	SouthAsia	India	modern	0	Dravidian	16.3	80.48	N.A.	N.A.	Lazaridis_et_al_2014	PASS
Vishwabrahmin14	Vishwabrahmin	SouthAsia	India	modern	0	Dravidian	16.3	80.48	N.A.	N.A.	Lazaridis_et_al_2014	PASS
Vishwabrahmin15	Vishwabrahmin	SouthAsia	India	modern	0	Dravidian	16.3	80.48	N.A.	N.A.	Lazaridis_et_al_2014	PASS
Mala1	Mala	SouthAsia	India	modern	0	Dravidian	18.67	78.17	N.A.	N.A.	Lazaridis_et_al_2014	PASS
Mala2	Mala	SouthAsia	India	modern	0	Dravidian	18.67	78.17	N.A.	N.A.	Lazaridis_et_al_2014	PASS
Mala3	Mala	SouthAsia	India	modern	0	Dravidian	18.67	78.17	N.A.	N.A.	Lazaridis_et_al_2014	PASS
Mala4	Mala	SouthAsia	India	modern	0	Dravidian	18.67	78.17	N.A.	N.A.	Lazaridis_et_al_2014	PASS
Mala5	Mala	SouthAsia	India	modern	0	Dravidian	18.67	78.17	N.A.	N.A.	Lazaridis_et_al_2014	PASS
Mala6	Mala	SouthAsia	India	modern	0	Dravidian	18.67	78.17	N.A.	N.A.	Lazaridis_et_al_2014	PASS
Mala7	Mala	SouthAsia	India	modern	0	Dravidian	18.67	78.17	N.A.	N.A.	Lazaridis_et_al_2014	PASS
Mala8	Mala	SouthAsia	India	modern	0	Dravidian	18.67	78.17	N.A.	N.A.	Lazaridis_et_al_2014	PASS
Mala9	Mala	SouthAsia	India	modern	0	Dravidian	18.67	78.17	N.A.	N.A.	Lazaridis_et_al_2014	PASS
Mala10	Mala	SouthAsia	India	modern	0	Dravidian	18.67	78.17	N.A.	N.A.	Lazaridis_et_al_2014	PASS
Mala11	Mala	SouthAsia	India	modern	0	Dravidian	18.67	78.17	N.A.	N.A.	Lazaridis_et_al_2014	PASS
Mala12	Mala	SouthAsia	India	modern	0	Dravidian	18.67	78.17	N.A.	N.A.	Lazaridis_et_al_2014	PASS
Mala13	Mala	SouthAsia	India	modern	0	Dravidian	18.67	78.17	N.A.	N.A.	Lazaridis_et_al_2014	PASS
Mala14	Mala	SouthAsia	India	modern	0	Dravidian	18.67	78.17	N.A.	N.A.	Lazaridis_et_al_2014	PASS
Mala15	Mala	SouthAsia	India	modern	0	Dravidian	18.67	78.17	N.A.	N.A.	Lazaridis_et_al_2014	PASS
Kharia1	Kharia	SouthAsia	India	modern	0	Austroasiatic	25.77	82.73	N.A.	N.A.	Lazaridis_et_al_2014	PASS
Kharia2	Kharia	SouthAsia	India	modern	0	Austroasiatic	25.77	82.73	N.A.	N.A.	Lazaridis_et_al_2014	PASS
Kharia3	Kharia	SouthAsia	India	modern	0	Austroasiatic	25.77	82.73	N.A.	N.A.	Lazaridis_et_al_2014	PASS
Kharia4	Kharia	SouthAsia	India	modern	0	Austroasiatic	25.77	82.73	N.A.	N.A.	Lazaridis_et_al_2014	PASS
Kharia5	Kharia	SouthAsia	India	modern	0	Austroasiatic	25.77	82.73	N.A.	N.A.	Lazaridis_et_al_2014	PASS
Kharia6	Kharia	SouthAsia	India	modern	0	Austroasiatic	25.77	82.73	N.A.	N.A.	Lazaridis_et_al_2014	PASS
Kharia7	Kharia	SouthAsia	India	modern	0	Austroasiatic	25.77	82.73	N.A.	N.A.	Lazaridis_et_al_2014	PASS
Kharia8	Kharia	SouthAsia	India	modern	0	Austroasiatic	25.77	82.73	N.A.	N.A.	Lazaridis_et_al_2014	PASS
Kharia9	Kharia	SouthAsia	India	modern	0	Austroasiatic	25.77	82.73	N.A.	N.A.	Lazaridis_et_al_2014	PASS
Kharia10	Kharia	SouthAsia	India	modern	0	Austroasiatic	25.77	82.73	N.A.	N.A.	Lazaridis_et_al_2014	PASS
Kharia11	Kharia	SouthAsia	India	modern	0	Austroasiatic	25.77	82.73	N.A.	N.A.	Lazaridis_et_al_2014	PASS
Kharia12	Kharia	SouthAsia	India	modern	0	Austroasiatic	25.77	82.73	N.A.	N.A.	Lazaridis_et_al_2014	PASS
Kharia13	Kharia	SouthAsia	India	modern	0	Austroasiatic	25.77	82.73	N.A.	N.A.	Lazaridis_et_al_2014	PASS
Kharia14	Kharia	SouthAsia	India	modern	0	Austroasiatic	25.77	82.73	N.A.	N.A.	Lazaridis_et_al_2014	PASS
Kharia15	Kharia	SouthAsia	India	modern	0	Austroasiatic	25.77	82.73	N.A.	N.A.	Lazaridis_et_al_2014	PASS
Onge1	Onge	SouthAsia	India	modern	0	Andamanese	10.8	92.5	N.A.	N.A.	Lazaridis_et_al_2014	PASS
Onge2	Onge	SouthAsia	India	modern	0	Andamanese	10.8	92.5	N.A.	N.A.	Lazaridis_et_al_2014	PASS
Onge3	Onge	SouthAsia	India	modern	0	Andamanese	10.8	92.5	N.A.	N.A.	Lazaridis_et_al_2014	PASS
Onge7	Onge	SouthAsia	India	modern	0	Andamanese	10.8	92.5	N.A.	N.A.	Lazaridis_et_al_2014	PASS
Onge9	Onge	SouthAsia	India	modern	0	Andamanese	10.8	92.5	N.A.	N.A.	Lazaridis_et_al_2014	PASS
Onge12	Onge	SouthAsia	India	modern	0	Andamanese	10.8	92.5	N.A.	N.A.	Lazaridis_et_al_2014	PASS
Onge13	Onge	SouthAsia	India	modern	0	Andamanese	10.8	92.5	N.A.	N.A.	Lazaridis_et_al_2014	PASS
Onge14	Onge	SouthAsia	India	modern	0	Andamanese	10.8	92.5	N.A.	N.A.	Lazaridis_et_al_2014	PASS
Onge15	Onge	SouthAsia	India	modern	0	Andamanese	10.8	92.5	N.A.	N.A.	Lazaridis_et_al_2014	PASS
Onge16	Onge	SouthAsia	India	modern	0	Andamanese	10.8	92.5	N.A.	N.A.	Lazaridis_et_al_2014	PASS

Onge17	Onge	SouthAsia	India	modern	0	Andamanese	10.8	92.5	N.A.	N.A.	Lazaridis_et_al_2014	PASS
UV345	Baining_Malasait	Oceania	Papua_New_Guinea	modern	0	East_New_Britain	-4.47	151.9	N.A.	N.A.	Skoglund_et_al_2016	PASS
UV346	Baining_Malasait	Oceania	Papua_New_Guinea	modern	0	East_New_Britain	-4.47	151.9	N.A.	N.A.	Skoglund_et_al_2016	PASS
UV347	Baining_Malasait	Oceania	Papua_New_Guinea	modern	0	East_New_Britain	-4.47	151.9	N.A.	N.A.	Skoglund_et_al_2016	PASS
UV368	Baining_Malasait	Oceania	Papua_New_Guinea	modern	0	East_New_Britain	-4.47	151.9	N.A.	N.A.	Skoglund_et_al_2016	kin
UV370	Baining_Malasait	Oceania	Papua_New_Guinea	modern	0	East_New_Britain	-4.47	151.9	N.A.	N.A.	Skoglund_et_al_2016	PASS
UV001	Baining_Marabu	Oceania	Papua_New_Guinea	modern	0	East_New_Britain	-4.63	152.3	N.A.	N.A.	Skoglund_et_al_2016	PASS
UV076	Baining_Marabu	Oceania	Papua_New_Guinea	modern	0	East_New_Britain	-4.63	152.3	N.A.	N.A.	Skoglund_et_al_2016	PASS
UV005	Baining_Marabu	Oceania	Papua_New_Guinea	modern	0	East_New_Britain	-4.63	152.3	N.A.	N.A.	Skoglund_et_al_2016	PASS
UV02	Baining_Marabu	Oceania	Papua_New_Guinea	modern	0	East_New_Britain	-4.63	152.3	N.A.	N.A.	Skoglund_et_al_2016	PASS
UV08	Baining_Marabu	Oceania	Papua_New_Guinea	modern	0	East_New_Britain	-4.63	152.3	N.A.	N.A.	Skoglund_et_al_2016	PASS
UV11	Baining_Marabu	Oceania	Papua_New_Guinea	modern	0	East_New_Britain	-4.63	152.3	N.A.	N.A.	Skoglund_et_al_2016	PASS
UV23	Baining_Marabu	Oceania	Papua_New_Guinea	modern	0	East_New_Britain	-4.63	152.3	N.A.	N.A.	Skoglund_et_al_2016	PASS
UV24	Baining_Marabu	Oceania	Papua_New_Guinea	modern	0	East_New_Britain	-4.63	152.3	N.A.	N.A.	Skoglund_et_al_2016	PASS
UV48	Baining_Marabu	Oceania	Papua_New_Guinea	modern	0	East_New_Britain	-4.63	152.3	N.A.	N.A.	Skoglund_et_al_2016	PASS
UV73	Baining_Marabu	Oceania	Papua_New_Guinea	modern	0	East_New_Britain	-4.63	152.3	N.A.	N.A.	Skoglund_et_al_2016	PASS
HGDP00655	Nasioi	Oceania	Papua_New_Guinea	modern	0	South_Bougainville	-6.48	155.83	N.A.	N.A.	Patterson_et_al_2012	PASS
HGDP00656	Nasioi	Oceania	Papua_New_Guinea	modern	0	South_Bougainville	-6.48	155.83	N.A.	N.A.	Patterson_et_al_2012	PASS
HGDP00660	Nasioi	Oceania	Papua_New_Guinea	modern	0	South_Bougainville	-6.48	155.83	N.A.	N.A.	Patterson_et_al_2012	PASS
HGDP00661	Nasioi	Oceania	Papua_New_Guinea	modern	0	South_Bougainville	-6.48	155.83	N.A.	N.A.	Patterson_et_al_2012	PASS
HGDP00662	Nasioi	Oceania	Papua_New_Guinea	modern	0	South_Bougainville	-6.48	155.83	N.A.	N.A.	Patterson_et_al_2012	PASS
HGDP00663	Nasioi	Oceania	Papua_New_Guinea	modern	0	South_Bougainville	-6.48	155.83	N.A.	N.A.	Patterson_et_al_2012	PASS
HGDP00664	Nasioi	Oceania	Papua_New_Guinea	modern	0	South_Bougainville	-6.48	155.83	N.A.	N.A.	Patterson_et_al_2012	PASS
HGDP00787	Nasioi	Oceania	Papua_New_Guinea	modern	0	South_Bougainville	-6.48	155.83	N.A.	N.A.	Patterson_et_al_2012	PASS
HGDP00788	Nasioi	Oceania	Papua_New_Guinea	modern	0	South_Bougainville	-6.48	155.83	N.A.	N.A.	Patterson_et_al_2012	PASS
HGDP01027	Nasioi	Oceania	Papua_New_Guinea	modern	0	South_Bougainville	-6.48	155.83	N.A.	N.A.	Patterson_et_al_2012	PASS
NG18	New_Guinea_Highlander	Oceania	Papua_New_Guinea	modern	0	Trans-New_Guinea	-6.15	143.66	N.A.	N.A.	Qin_and_Stoneking_2015	PASS
SH09	New_Guinea_Highlander	Oceania	Papua_New_Guinea	modern	0	Trans-New_Guinea	-6.15	143.66	N.A.	N.A.	Qin_and_Stoneking_2015	PASS
NG20	New_Guinea_Highlander	Oceania	Papua_New_Guinea	modern	0	Trans-New_Guinea	-6.15	143.66	N.A.	N.A.	Qin_and_Stoneking_2015	PASS
SH10	New_Guinea_Highlander	Oceania	Papua_New_Guinea	modern	0	Trans-New_Guinea	-6.15	143.66	N.A.	N.A.	Qin_and_Stoneking_2015	PASS
NG4	New_Guinea_Highlander	Oceania	Papua_New_Guinea	modern	0	Trans-New_Guinea	-6.15	143.66	N.A.	N.A.	Qin_and_Stoneking_2015	PASS
NG22	New_Guinea_Highlander	Oceania	Papua_New_Guinea	modern	0	Trans-New_Guinea	-6.15	143.66	N.A.	N.A.	Qin_and_Stoneking_2015	PASS
SH18	New_Guinea_Highlander	Oceania	Papua_New_Guinea	modern	0	Trans-New_Guinea	-6.15	143.66	N.A.	N.A.	Qin_and_Stoneking_2015	PASS
SH33	New_Guinea_Highlander	Oceania	Papua_New_Guinea	modern	0	Trans-New_Guinea	-6.15	143.66	N.A.	N.A.	Qin_and_Stoneking_2015	PASS
NG25	New_Guinea_Highlander	Oceania	Papua_New_Guinea	modern	0	Trans-New_Guinea	-6.15	143.66	N.A.	N.A.	Qin_and_Stoneking_2015	PASS
SH19	New_Guinea_Highlander	Oceania	Papua_New_Guinea	modern	0	Trans-New_Guinea	-6.15	143.66	N.A.	N.A.	Qin_and_Stoneking_2015	PASS
NG7	New_Guinea_Highlander	Oceania	Papua_New_Guinea	modern	0	Trans-New_Guinea	-6.15	143.66	N.A.	N.A.	Qin_and_Stoneking_2015	PASS
NG26	New_Guinea_Highlander	Oceania	Papua_New_Guinea	modern	0	Trans-New_Guinea	-6.15	143.66	N.A.	N.A.	Qin_and_Stoneking_2015	PASS
SH20	New_Guinea_Highlander	Oceania	Papua_New_Guinea	modern	0	Trans-New_Guinea	-6.15	143.66	N.A.	N.A.	Qin_and_Stoneking_2015	PASS
NG13	New_Guinea_Highlander	Oceania	Papua_New_Guinea	modern	0	Trans-New_Guinea	-6.15	143.66	N.A.	N.A.	Qin_and_Stoneking_2015	PASS
SH05	New_Guinea_Highlander	Oceania	Papua_New_Guinea	modern	0	Trans-New_Guinea	-6.15	143.66	N.A.	N.A.	Qin_and_Stoneking_2015	PASS
SH31	New_Guinea_Highlander	Oceania	Papua_New_Guinea	modern	0	Trans-New_Guinea	-6.15	143.66	N.A.	N.A.	Qin_and_Stoneking_2015	PASS

SH07	New_Guinea_Highlander	Oceania	Papua_New_Guinea	modern	0	Trans-New_Guinea	-6.15	143.66	N.A.	N.A.	Qin_and_Stoneking_2015	PASS
NG17	New_Guinea_Highlander	Oceania	Papua_New_Guinea	modern	0	Trans-New_Guinea	-6.15	143.66	N.A.	N.A.	Qin_and_Stoneking_2015	PASS
SH08	New_Guinea_Highlander	Oceania	Papua_New_Guinea	modern	0	Trans-New_Guinea	-6.15	143.66	N.A.	N.A.	Qin_and_Stoneking_2015	PASS
NG24	Papuan_Eastern_Highlands	Oceania	Papua_New_Guinea	modern	0	Trans-New_Guinea	-6.1	145.4	N.A.	N.A.	Qin_and_Stoneking_2015	PASS
NG10	Papuan_Eastern_Highlands	Oceania	Papua_New_Guinea	modern	0	Trans-New_Guinea	-6.1	145.4	N.A.	N.A.	Qin_and_Stoneking_2015	PASS
NG11	Papuan_Eastern_Highlands	Oceania	Papua_New_Guinea	modern	0	Trans-New_Guinea	-6.1	145.4	N.A.	N.A.	Qin_and_Stoneking_2015	PASS
NG28	Papuan_Eastern_Highlands	Oceania	Papua_New_Guinea	modern	0	Trans-New_Guinea	-6.1	145.4	N.A.	N.A.	Qin_and_Stoneking_2015	PASS
CP3	Papuan_Central_Province	Oceania	Papua_New_Guinea	modern	0	Trans-New_Guinea	-9.48	147.2	N.A.	N.A.	Qin_and_Stoneking_2015	PASS
CP7	Papuan_Central_Province	Oceania	Papua_New_Guinea	modern	0	Trans-New_Guinea	-9.48	147.2	N.A.	N.A.	Qin_and_Stoneking_2015	PASS
CP10	Papuan_Central_Province	Oceania	Papua_New_Guinea	modern	0	Trans-New_Guinea	-9.48	147.2	N.A.	N.A.	Qin_and_Stoneking_2015	PASS
CP13	Papuan_Central_Province	Oceania	Papua_New_Guinea	modern	0	Trans-New_Guinea	-9.48	147.2	N.A.	N.A.	Qin_and_Stoneking_2015	PASS
CP14	Papuan_Central_Province	Oceania	Papua_New_Guinea	modern	0	Trans-New_Guinea	-9.48	147.2	N.A.	N.A.	Qin_and_Stoneking_2015	PASS
GP3	Papuan_Gulf_Province	Oceania	Papua_New_Guinea	modern	0	Trans-New_Guinea	-7.88	145.69	N.A.	N.A.	Qin_and_Stoneking_2015	PASS
GP4	Papuan_Gulf_Province	Oceania	Papua_New_Guinea	modern	0	Trans-New_Guinea	-7.88	145.69	N.A.	N.A.	Qin_and_Stoneking_2015	PASS
GP6	Papuan_Gulf_Province	Oceania	Papua_New_Guinea	modern	0	Trans-New_Guinea	-7.88	145.69	N.A.	N.A.	Qin_and_Stoneking_2015	PASS
Y4349	Tongan	Oceania	Kingdom_of_Tonga	modern	0	Austronesian	-21.19	-175.18	Tongan	N.A.	Qin_and_Stoneking_2015	PASS
Y4383	Tongan	Oceania	Kingdom_of_Tonga	modern	0	Austronesian	-21.19	-175.18	Tongan	N.A.	Qin_and_Stoneking_2015	PASS
Y4914	Tongan	Oceania	Kingdom_of_Tonga	modern	0	Austronesian	-21.19	-175.18	Tongan	N.A.	Qin_and_Stoneking_2015	PASS
Y6254	Tongan	Oceania	Kingdom_of_Tonga	modern	0	Austronesian	-21.19	-175.18	Tongan	N.A.	Qin_and_Stoneking_2015	PASS
Y5707	Tongan	Oceania	Kingdom_of_Tonga	modern	0	Austronesian	-21.19	-175.18	Tongan	N.A.	Qin_and_Stoneking_2015	PASS
Y5718	Tongan	Oceania	Kingdom_of_Tonga	modern	0	Austronesian	-21.19	-175.18	Tongan	N.A.	Qin_and_Stoneking_2015	PASS
VL43	Vella_Lavella	Oceania	Solomon_Islands	modern	0	Central_Solomons	-7.9	156.7	N.A.	N.A.	Qin_and_Stoneking_2015	PASS
KO39	Vella_Lavella	Oceania	Solomon_Islands	modern	0	Central_Solomons	-7.9	156.7	N.A.	N.A.	Qin_and_Stoneking_2015	PASS
VL02	Vella_Lavella	Oceania	Solomon_Islands	modern	0	Central_Solomons	-7.9	156.7	N.A.	N.A.	Qin_and_Stoneking_2015	PASS
VL08	Vella_Lavella	Oceania	Solomon_Islands	modern	0	Central_Solomons	-7.9	156.7	N.A.	N.A.	Qin_and_Stoneking_2015	PASS
VL09	Vella_Lavella	Oceania	Solomon_Islands	modern	0	Central_Solomons	-7.9	156.7	N.A.	N.A.	Qin_and_Stoneking_2015	PASS
VL11	Vella_Lavella	Oceania	Solomon_Islands	modern	0	Central_Solomons	-7.9	156.7	N.A.	N.A.	Qin_and_Stoneking_2015	PASS
PH10_1	Mamanwa	ISEA	Philippines	modern	0	Austronesian	9.76	125.51	Mamanwa	N.A.	Qin_and_Stoneking_2015	PASS
PH65_1	Mamanwa	ISEA	Philippines	modern	0	Austronesian	9.76	125.51	Mamanwa	N.A.	Qin_and_Stoneking_2015	PASS
PH2	Mamanwa	ISEA	Philippines	modern	0	Austronesian	9.76	125.51	Mamanwa	N.A.	Qin_and_Stoneking_2015	PASS
PH35_1	Mamanwa	ISEA	Philippines	modern	0	Austronesian	9.76	125.51	Mamanwa	N.A.	Qin_and_Stoneking_2015	PASS
PH38_1	Mamanwa	ISEA	Philippines	modern	0	Austronesian	9.76	125.51	Mamanwa	N.A.	Qin_and_Stoneking_2015	PASS
PH4	Mamanwa	ISEA	Philippines	modern	0	Austronesian	9.76	125.51	Mamanwa	N.A.	Qin_and_Stoneking_2015	PASS
PH45_1	Mamanwa	ISEA	Philippines	modern	0	Austronesian	9.76	125.51	Mamanwa	N.A.	Qin_and_Stoneking_2015	PASS
PH66_2	Mamanwa	ISEA	Philippines	modern	0	Austronesian	9.76	125.51	Mamanwa	N.A.	Qin_and_Stoneking_2015	PASS
PH67_2	Mamanwa	ISEA	Philippines	modern	0	Austronesian	9.76	125.51	Mamanwa	N.A.	Qin_and_Stoneking_2015	PASS
PH72_3	Mamanwa	ISEA	Philippines	modern	0	Austronesian	9.76	125.51	Mamanwa	N.A.	Qin_and_Stoneking_2015	PASS
PH77_1	Mamanwa	ISEA	Philippines	modern	0	Austronesian	9.76	125.51	Mamanwa	N.A.	Qin_and_Stoneking_2015	PASS
PH8	Mamanwa	ISEA	Philippines	modern	0	Austronesian	9.76	125.51	Mamanwa	N.A.	Qin_and_Stoneking_2015	PASS
PH9	Mamanwa	ISEA	Philippines	modern	0	Austronesian	9.76	125.51	Mamanwa	N.A.	Qin_and_Stoneking_2015	PASS
PH50_1	Mamanwa1	ISEA	Philippines	modern	0	Austronesian	9.76	125.51	Mamanwa	N.A.	Qin_and_Stoneking_2015	PASS
PH52_3	Mamanwa1	ISEA	Philippines	modern	0	Austronesian	9.76	125.51	Mamanwa	N.A.	Qin_and_Stoneking_2015	PASS

PH53_1	Mamanwa1	ISEA	Philippines	modern	0	Austronesian	9.76	125.51	Mamanwa	N.A.	Qin_and_Stoneking_2015	PASS
PH55_2	Mamanwa1	ISEA	Philippines	modern	0	Austronesian	9.76	125.51	Mamanwa	N.A.	Qin_and_Stoneking_2015	PASS
PH56_1	Mamanwa1	ISEA	Philippines	modern	0	Austronesian	9.76	125.51	Mamanwa	N.A.	Qin_and_Stoneking_2015	PASS
Smd1_1	Semende	ISEA	Indonesia	modern	0	Austronesian	3.58	98.67	Semende	N.A.	Qin_and_Stoneking_2015	PASS
Smd10_1	Semende	ISEA	Indonesia	modern	0	Austronesian	3.58	98.67	Semende	N.A.	Qin_and_Stoneking_2015	PASS
Smd14_1	Semende	ISEA	Indonesia	modern	0	Austronesian	3.58	98.67	Semende	N.A.	Qin_and_Stoneking_2015	PASS
Smd15_1	Semende	ISEA	Indonesia	modern	0	Austronesian	3.58	98.67	Semende	N.A.	Qin_and_Stoneking_2015	PASS
Smd18_1	Semende	ISEA	Indonesia	modern	0	Austronesian	3.58	98.67	Semende	N.A.	Qin_and_Stoneking_2015	PASS
Smd19_1	Semende	ISEA	Indonesia	modern	0	Austronesian	3.58	98.67	Semende	N.A.	Qin_and_Stoneking_2015	PASS
Smd3_1	Semende	ISEA	Indonesia	modern	0	Austronesian	3.58	98.67	Semende	N.A.	Qin_and_Stoneking_2015	PASS
Smd5_1	Semende	ISEA	Indonesia	modern	0	Austronesian	3.58	98.67	Semende	N.A.	Qin_and_Stoneking_2015	PASS
Smd6_1	Semende	ISEA	Indonesia	modern	0	Austronesian	3.58	98.67	Semende	N.A.	Qin_and_Stoneking_2015	PASS
Smd9_1	Semende	ISEA	Indonesia	modern	0	Austronesian	3.58	98.67	Semende	N.A.	Qin_and_Stoneking_2015	PASS
Bor1_5	Borneo	ISEA	Indonesia	modern	0	Austronesian	0.18	110.16	Borneo	N.A.	Qin_and_Stoneking_2015	PASS
Bor16_1	Borneo	ISEA	Indonesia	modern	0	Austronesian	0.18	110.16	Borneo	N.A.	Qin_and_Stoneking_2015	PASS
Bor2_3	Borneo	ISEA	Indonesia	modern	0	Austronesian	0.18	110.16	Borneo	N.A.	Qin_and_Stoneking_2015	PASS
Bor20_1	Borneo	ISEA	Indonesia	modern	0	Austronesian	0.18	110.16	Borneo	N.A.	Qin_and_Stoneking_2015	PASS
Bor3_3	Borneo	ISEA	Indonesia	modern	0	Austronesian	0.18	110.16	Borneo	N.A.	Qin_and_Stoneking_2015	PASS
Bor4_5	Borneo	ISEA	Indonesia	modern	0	Austronesian	0.18	110.16	Borneo	N.A.	Qin_and_Stoneking_2015	PASS
Bor6_1	Borneo	ISEA	Indonesia	modern	0	Austronesian	0.18	110.16	Borneo	N.A.	Qin_and_Stoneking_2015	PASS
Bor7_2	Borneo	ISEA	Indonesia	modern	0	Austronesian	0.18	110.16	Borneo	N.A.	Qin_and_Stoneking_2015	PASS
Bor9_3	Borneo	ISEA	Indonesia	modern	0	Austronesian	0.18	110.16	Borneo	N.A.	Qin_and_Stoneking_2015	PASS
Dusun4	Dusun	ISEA	Brunei	modern	0	Austronesian	4.71	114.67	Dusun	N.A.	Skoglund_et_al_2016	PASS
Dusun7	Dusun	ISEA	Brunei	modern	0	Austronesian	4.71	114.67	Dusun	N.A.	Skoglund_et_al_2016	PASS
Dusun8	Dusun	ISEA	Brunei	modern	0	Austronesian	4.71	114.67	Dusun	N.A.	Skoglund_et_al_2016	PASS
Dusun10	Dusun	ISEA	Brunei	modern	0	Austronesian	4.71	114.67	Dusun	N.A.	Skoglund_et_al_2016	PASS
Dusun11	Dusun	ISEA	Brunei	modern	0	Austronesian	4.71	114.67	Dusun	N.A.	Skoglund_et_al_2016	PASS
Dusun12	Dusun	ISEA	Brunei	modern	0	Austronesian	4.71	114.67	Dusun	N.A.	Skoglund_et_al_2016	PASS
Dusun14	Dusun	ISEA	Brunei	modern	0	Austronesian	4.71	114.67	Dusun	N.A.	Skoglund_et_al_2016	PASS
Dusun21	Dusun	ISEA	Brunei	modern	0	Austronesian	4.71	114.67	Dusun	N.A.	Skoglund_et_al_2016	PASS
Dusun22	Dusun	ISEA	Brunei	modern	0	Austronesian	4.71	114.67	Dusun	N.A.	Skoglund_et_al_2016	PASS
Dusun23	Dusun	ISEA	Brunei	modern	0	Austronesian	4.71	114.67	Dusun	N.A.	Skoglund_et_al_2016	PASS
Murut2	Murut	ISEA	Brunei	modern	0	Austronesian	4.62	115.14	Murut	N.A.	Skoglund_et_al_2016	PASS
Murut3	Murut	ISEA	Brunei	modern	0	Austronesian	4.62	115.14	Murut	N.A.	Skoglund_et_al_2016	PASS
Murut4	Murut	ISEA	Brunei	modern	0	Austronesian	4.62	115.14	Murut	N.A.	Skoglund_et_al_2016	PASS
Murut5	Murut	ISEA	Brunei	modern	0	Austronesian	4.62	115.14	Murut	N.A.	Skoglund_et_al_2016	PASS
Murut6	Murut	ISEA	Brunei	modern	0	Austronesian	4.62	115.14	Murut	N.A.	Skoglund_et_al_2016	PASS
Murut9	Murut	ISEA	Brunei	modern	0	Austronesian	4.62	115.14	Murut	N.A.	Skoglund_et_al_2016	PASS
Murut11	Murut	ISEA	Brunei	modern	0	Austronesian	4.62	115.14	Murut	N.A.	Skoglund_et_al_2016	PASS
Murut13	Murut	ISEA	Brunei	modern	0	Austronesian	4.62	115.14	Murut	N.A.	Skoglund_et_al_2016	PASS
Murut15	Murut	ISEA	Brunei	modern	0	Austronesian	4.62	115.14	Murut	N.A.	Skoglund_et_al_2016	PASS
Murut17	Murut	ISEA	Brunei	modern	0	Austronesian	4.62	115.14	Murut	N.A.	Skoglund_et_al_2016	PASS
Burm4	Burmese	MSEA	Myanmar	modern	0	Sino-Tibetan	16.41	95.89	Burmese	N.A.	Skoglund_et_al_2016	PASS
Burm7	Burmese	MSEA	Myanmar	modern	0	Sino-Tibetan	16.41	95.89	Burmese	N.A.	Skoglund_et_al_2016	PASS

Burm8	Burmese	MSEA	Myanmar	modern	0	Sino-Tibetan	16.41	95.89	Burmese	N.A.	Skoglund_et_al_2016	PASS
Burm9	Burmese	MSEA	Myanmar	modern	0	Sino-Tibetan	16.41	95.89	Burmese	N.A.	Skoglund_et_al_2016	PASS
Burm10	Burmese	MSEA	Myanmar	modern	0	Sino-Tibetan	16.41	95.89	Burmese	N.A.	Skoglund_et_al_2016	PASS
Burm12	Burmese	MSEA	Myanmar	modern	0	Sino-Tibetan	16.41	95.89	Burmese	N.A.	Skoglund_et_al_2016	PASS
Burm13	Burmese	MSEA	Myanmar	modern	0	Sino-Tibetan	16.41	95.89	Burmese	N.A.	Skoglund_et_al_2016	PASS
Burm14	Burmese	MSEA	Myanmar	modern	0	Sino-Tibetan	16.41	95.89	Burmese	N.A.	Skoglund_et_al_2016	PASS
Burm15	Burmese	MSEA	Myanmar	modern	0	Sino-Tibetan	16.41	95.89	Burmese	N.A.	Skoglund_et_al_2016	PASS
Burm16	Burmese	MSEA	Myanmar	modern	0	Sino-Tibetan	16.41	95.89	Burmese	N.A.	Skoglund_et_al_2016	PASS
Igorot3	Kankanaey	ISEA	Philippines	modern	0	Austronesian	17.07	121.03	Kankanaey	N.A.	Skoglund_et_al_2016	PASS
Igorot4	Kankanaey	ISEA	Philippines	modern	0	Austronesian	17.07	121.03	Kankanaey	N.A.	Skoglund_et_al_2016	PASS
Igorot5	Kankanaey	ISEA	Philippines	modern	0	Austronesian	17.07	121.03	Kankanaey	N.A.	Skoglund_et_al_2016	PASS
Igorot6	Kankanaey	ISEA	Philippines	modern	0	Austronesian	17.07	121.03	Kankanaey	N.A.	Skoglund_et_al_2016	PASS
Igorot10	Kankanaey	ISEA	Philippines	modern	0	Austronesian	17.07	121.03	Kankanaey	N.A.	Skoglund_et_al_2016	PASS
Igorot11	Kankanaey	ISEA	Philippines	modern	0	Austronesian	17.07	121.03	Kankanaey	N.A.	Skoglund_et_al_2016	PASS
Igorot13	Kankanaey	ISEA	Philippines	modern	0	Austronesian	17.07	121.03	Kankanaey	N.A.	Skoglund_et_al_2016	PASS
Igorot17	Kankanaey	ISEA	Philippines	modern	0	Austronesian	17.07	121.03	Kankanaey	N.A.	Skoglund_et_al_2016	PASS
Igorot21	Kankanaey	ISEA	Philippines	modern	0	Austronesian	17.07	121.03	Kankanaey	N.A.	Skoglund_et_al_2016	PASS
Igorot23	Kankanaey	ISEA	Philippines	modern	0	Austronesian	17.07	121.03	Kankanaey	N.A.	Skoglund_et_al_2016	PASS
Luz2	Ilocano	ISEA	Philippines	modern	0	Austronesian	14.6	120.98	Ilocano	N.A.	Skoglund_et_al_2016	PASS
Luz6	Ilocano	ISEA	Philippines	modern	0	Austronesian	14.6	120.98	Ilocano	N.A.	Skoglund_et_al_2016	PASS
Luz5	Tagalog	ISEA	Philippines	modern	0	Austronesian	14.6	120.98	Tagalog	N.A.	Skoglund_et_al_2016	PASS
Luz8	Tagalog	ISEA	Philippines	modern	0	Austronesian	14.6	120.98	Tagalog	N.A.	Skoglund_et_al_2016	PASS
Luz9	Tagalog	ISEA	Philippines	modern	0	Austronesian	14.6	120.98	Tagalog	N.A.	Skoglund_et_al_2016	PASS
Luz10	Tagalog	ISEA	Philippines	modern	0	Austronesian	14.6	120.98	Tagalog	N.A.	Skoglund_et_al_2016	PASS
Luz11	Tagalog	ISEA	Philippines	modern	0	Austronesian	14.6	120.98	Tagalog	N.A.	Skoglund_et_al_2016	PASS
Vizaya1	Visayan	ISEA	Philippines	modern	0	Austronesian	9.76	125.51	Visayan	N.A.	Skoglund_et_al_2016	PASS
Vizaya2	Visayan	ISEA	Philippines	modern	0	Austronesian	9.76	125.51	Visayan	N.A.	Skoglund_et_al_2016	PASS
Vizaya3	Visayan	ISEA	Philippines	modern	0	Austronesian	9.76	125.51	Visayan	N.A.	Skoglund_et_al_2016	PASS
Vizaya4	Visayan	ISEA	Philippines	modern	0	Austronesian	9.76	125.51	Visayan	N.A.	Skoglund_et_al_2016	PASS
Malay2	Malay	ISEA	Singapore	modern	0	Austronesian	1.35	103.82	Malay	N.A.	Skoglund_et_al_2016	PASS
Malay5	Malay	ISEA	Singapore	modern	0	Austronesian	1.35	103.82	Malay	N.A.	Skoglund_et_al_2016	PASS
Malay6	Malay	ISEA	Singapore	modern	0	Austronesian	1.35	103.82	Malay	N.A.	Skoglund_et_al_2016	PASS
Malay10	Malay	ISEA	Singapore	modern	0	Austronesian	1.35	103.82	Malay	N.A.	Skoglund_et_al_2016	PASS
Malay11	Malay	ISEA	Singapore	modern	0	Austronesian	1.35	103.82	Malay	N.A.	Skoglund_et_al_2016	PASS
Malay13	Malay	ISEA	Singapore	modern	0	Austronesian	1.35	103.82	Malay	N.A.	Skoglund_et_al_2016	PASS
Malay14	Malay	ISEA	Singapore	modern	0	Austronesian	1.35	103.82	Malay	N.A.	Skoglund_et_al_2016	PASS
Malay20	Malay	ISEA	Singapore	modern	0	Austronesian	1.35	103.82	Malay	N.A.	Skoglund_et_al_2016	PASS
Malay21	Malay	ISEA	Singapore	modern	0	Austronesian	1.35	103.82	Malay	N.A.	Skoglund_et_al_2016	PASS
NA13607	Amis_HO	sEA	Taiwan	modern	0	Austronesian	22.8	121.2	Amis	TW_S	Lazaridis_et_al_2014	PASS
NA13608	Amis_HO	sEA	Taiwan	modern	0	Austronesian	22.8	121.2	Amis	TW_S	Lazaridis_et_al_2014	PASS
NA13609	Amis_HO	sEA	Taiwan	modern	0	Austronesian	22.8	121.2	Amis	TW_S	Lazaridis_et_al_2014	PASS
NA13610	Amis_HO	sEA	Taiwan	modern	0	Austronesian	22.8	121.2	Amis	TW_S	Lazaridis_et_al_2014	PASS
NA13611	Amis_HO	sEA	Taiwan	modern	0	Austronesian	22.8	121.2	Amis	TW_S	Lazaridis_et_al_2014	PASS
NA13612	Amis_HO	sEA	Taiwan	modern	0	Austronesian	22.8	121.2	Amis	TW_S	Lazaridis_et_al_2014	PASS

NA13613	Amis_HO	sEA	Taiwan	modern	0	Austronesian	22.8	121.2	Amis	TW_S	Lazaridis_et_al_2014	PASS
NA13614	Amis_HO	sEA	Taiwan	modern	0	Austronesian	22.8	121.2	Amis	TW_S	Lazaridis_et_al_2014	PASS
NA13615	Amis_HO	sEA	Taiwan	modern	0	Austronesian	22.8	121.2	Amis	TW_S	Lazaridis_et_al_2014	PASS
NA13616	Amis_HO	sEA	Taiwan	modern	0	Austronesian	22.8	121.2	Amis	TW_S	Lazaridis_et_al_2014	PASS
NA13597	Atayal_HO	sEA	Taiwan	modern	0	Austronesian	24.6	121.3	Atayal	TW_N	Lazaridis_et_al_2014	PASS
NA13598	Atayal_HO	sEA	Taiwan	modern	0	Austronesian	24.6	121.3	Atayal	TW_N	Lazaridis_et_al_2014	PASS
NA13599	Atayal_HO	sEA	Taiwan	modern	0	Austronesian	24.6	121.3	Atayal	TW_N	Lazaridis_et_al_2014	PASS
NA13600	Atayal_HO	sEA	Taiwan	modern	0	Austronesian	24.6	121.3	Atayal	TW_N	Lazaridis_et_al_2014	PASS
NA13601	Atayal_HO	sEA	Taiwan	modern	0	Austronesian	24.6	121.3	Atayal	TW_N	Lazaridis_et_al_2014	PASS
NA13602	Atayal_HO	sEA	Taiwan	modern	0	Austronesian	24.6	121.3	Atayal	TW_N	Lazaridis_et_al_2014	PASS
NA13603	Atayal_HO	sEA	Taiwan	modern	0	Austronesian	24.6	121.3	Atayal	TW_N	Lazaridis_et_al_2014	PASS
NA13604	Atayal_HO	sEA	Taiwan	modern	0	Austronesian	24.6	121.3	Atayal	TW_N	Lazaridis_et_al_2014	PASS
NA13605	Atayal_HO	sEA	Taiwan	modern	0	Austronesian	24.6	121.3	Atayal	TW_N	Lazaridis_et_al_2014	PASS
NA13606	Atayal_HO	sEA	Taiwan	modern	0	Austronesian	24.6	121.3	Atayal	TW_N	Lazaridis_et_al_2014	PASS
HGDP00711	Cambodian	MSEA	Cambodia	modern	0	Austroasiatic	12	105	N.A.	N.A.	Patterson_et_al_2012	PASS
HGDP00712	Cambodian	MSEA	Cambodia	modern	0	Austroasiatic	12	105	N.A.	N.A.	Patterson_et_al_2012	PASS
HGDP00713	Cambodian	MSEA	Cambodia	modern	0	Austroasiatic	12	105	N.A.	N.A.	Patterson_et_al_2012	PASS
HGDP00714	Cambodian	MSEA	Cambodia	modern	0	Austroasiatic	12	105	N.A.	N.A.	Patterson_et_al_2012	PASS
HGDP00715	Cambodian	MSEA	Cambodia	modern	0	Austroasiatic	12	105	N.A.	N.A.	Patterson_et_al_2012	PASS
HGDP00716	Cambodian	MSEA	Cambodia	modern	0	Austroasiatic	12	105	N.A.	N.A.	Patterson_et_al_2012	PASS
HGDP00717	Cambodian	MSEA	Cambodia	modern	0	Austroasiatic	12	105	N.A.	N.A.	Patterson_et_al_2012	PASS
HGDP00719	Cambodian	MSEA	Cambodia	modern	0	Austroasiatic	12	105	N.A.	N.A.	Patterson_et_al_2012	PASS
HGDP00720	Cambodian	MSEA	Cambodia	modern	0	Austroasiatic	12	105	N.A.	N.A.	Patterson_et_al_2012	PASS
HGDP00721	Cambodian	MSEA	Cambodia	modern	0	Austroasiatic	12	105	N.A.	N.A.	Patterson_et_al_2012	PASS
HG01842	Kinh	MSEA	Vietnam	modern	0	Austroasiatic	21	105.9	N.A.	N.A.	Patterson_et_al_2012	PASS
HG01844	Kinh	MSEA	Vietnam	modern	0	Austroasiatic	21	105.9	N.A.	N.A.	Patterson_et_al_2012	PASS
HG01846	Kinh	MSEA	Vietnam	modern	0	Austroasiatic	21	105.9	N.A.	N.A.	Patterson_et_al_2012	PASS
HG01600	Kinh	MSEA	Vietnam	modern	0	Austroasiatic	21	105.9	N.A.	N.A.	Patterson_et_al_2012	PASS
HG01595	Kinh	MSEA	Vietnam	modern	0	Austroasiatic	21	105.9	N.A.	N.A.	Patterson_et_al_2012	PASS
HG01596	Kinh	MSEA	Vietnam	modern	0	Austroasiatic	21	105.9	N.A.	N.A.	Patterson_et_al_2012	PASS
HG01597	Kinh	MSEA	Vietnam	modern	0	Austroasiatic	21	105.9	N.A.	N.A.	Patterson_et_al_2012	PASS
HG01598	Kinh	MSEA	Vietnam	modern	0	Austroasiatic	21	105.9	N.A.	N.A.	Patterson_et_al_2012	PASS
HGDP01307	Dai	sEA	China	modern	0	Tai-Kadai	21	100	Dai	N.A.	Patterson_et_al_2012	PASS
HGDP01308	Dai	sEA	China	modern	0	Tai-Kadai	21	100	Dai	N.A.	Patterson_et_al_2012	PASS
HGDP01309	Dai	sEA	China	modern	0	Tai-Kadai	21	100	Dai	N.A.	Patterson_et_al_2012	PASS
HGDP01310	Dai	sEA	China	modern	0	Tai-Kadai	21	100	Dai	N.A.	Patterson_et_al_2012	PASS
HGDP01311	Dai	sEA	China	modern	0	Tai-Kadai	21	100	Dai	N.A.	Patterson_et_al_2012	PASS
HGDP01312	Dai	sEA	China	modern	0	Tai-Kadai	21	100	Dai	N.A.	Patterson_et_al_2012	PASS
HGDP01313	Dai	sEA	China	modern	0	Tai-Kadai	21	100	Dai	N.A.	Patterson_et_al_2012	PASS
HGDP01314	Dai	sEA	China	modern	0	Tai-Kadai	21	100	Dai	N.A.	Patterson_et_al_2012	PASS
HGDP01315	Dai	sEA	China	modern	0	Tai-Kadai	21	100	Dai	N.A.	Patterson_et_al_2012	PASS
HGDP01316	Dai	sEA	China	modern	0	Tai-Kadai	21	100	Dai	N.A.	Patterson_et_al_2012	PASS
HGDP01189	Miao	sEA	China	modern	0	Hmong-Mien	28	109	N.A.	N.A.	Patterson_et_al_2012	PASS
HGDP01190	Miao	sEA	China	modern	0	Hmong-Mien	28	109	N.A.	N.A.	Patterson_et_al_2012	PASS

HGDP01191	Miao	sEA	China	modern	0	Hmong-Mien	28	109	N.A.	N.A.	Patterson_et_al_2012	PASS
HGDP01192	Miao	sEA	China	modern	0	Hmong-Mien	28	109	N.A.	N.A.	Patterson_et_al_2012	PASS
HGDP01193	Miao	sEA	China	modern	0	Hmong-Mien	28	109	N.A.	N.A.	Patterson_et_al_2012	PASS
HGDP01194	Miao	sEA	China	modern	0	Hmong-Mien	28	109	N.A.	N.A.	Patterson_et_al_2012	PASS
HGDP01195	Miao	sEA	China	modern	0	Hmong-Mien	28	109	N.A.	N.A.	Patterson_et_al_2012	PASS
HGDP01196	Miao	sEA	China	modern	0	Hmong-Mien	28	109	N.A.	N.A.	Patterson_et_al_2012	PASS
HGDP01197	Miao	sEA	China	modern	0	Hmong-Mien	28	109	N.A.	N.A.	Patterson_et_al_2012	PASS
HGDP01198	Miao	sEA	China	modern	0	Hmong-Mien	28	109	N.A.	N.A.	Patterson_et_al_2012	PASS
HGDP01327	She	sEA	China	modern	0	Hmong-Mien	27	119	N.A.	N.A.	Patterson_et_al_2012	PASS
HGDP01328	She	sEA	China	modern	0	Hmong-Mien	27	119	N.A.	N.A.	Patterson_et_al_2012	PASS
HGDP01329	She	sEA	China	modern	0	Hmong-Mien	27	119	N.A.	N.A.	Patterson_et_al_2012	PASS
HGDP01330	She	sEA	China	modern	0	Hmong-Mien	27	119	N.A.	N.A.	Patterson_et_al_2012	PASS
HGDP01331	She	sEA	China	modern	0	Hmong-Mien	27	119	N.A.	N.A.	Patterson_et_al_2012	PASS
HGDP01332	She	sEA	China	modern	0	Hmong-Mien	27	119	N.A.	N.A.	Patterson_et_al_2012	PASS
HGDP01333	She	sEA	China	modern	0	Hmong-Mien	27	119	N.A.	N.A.	Patterson_et_al_2012	PASS
HGDP01334	She	sEA	China	modern	0	Hmong-Mien	27	119	N.A.	N.A.	Patterson_et_al_2012	PASS
HGDP01335	She	sEA	China	modern	0	Hmong-Mien	27	119	N.A.	N.A.	Patterson_et_al_2012	PASS
HGDP01336	She	sEA	China	modern	0	Hmong-Mien	27	119	N.A.	N.A.	Patterson_et_al_2012	PASS
HGDP01317	Lahu_C	sEA	China	modern	0	Sino-Tibetan	22	100	Lahu	N.A.	Patterson_et_al_2012	PASS
HGDP01318	Lahu_C	sEA	China	modern	0	Sino-Tibetan	22	100	Lahu	N.A.	Patterson_et_al_2012	PASS
HGDP01319	Lahu_C	sEA	China	modern	0	Sino-Tibetan	22	100	Lahu	N.A.	Patterson_et_al_2012	PASS
HGDP01320	Lahu_C	sEA	China	modern	0	Sino-Tibetan	22	100	Lahu	N.A.	Patterson_et_al_2012	PASS
HGDP01321	Lahu_C	sEA	China	modern	0	Sino-Tibetan	22	100	Lahu	N.A.	Patterson_et_al_2012	PASS
HGDP01322	Lahu_C	sEA	China	modern	0	Sino-Tibetan	22	100	Lahu	N.A.	Patterson_et_al_2012	PASS
HGDP01323	Lahu_C	sEA	China	modern	0	Sino-Tibetan	22	100	Lahu	N.A.	Patterson_et_al_2012	PASS
HGDP01326	Lahu_C	sEA	China	modern	0	Sino-Tibetan	22	100	Lahu	N.A.	Patterson_et_al_2012	PASS
HGDP01179	Yi	sEA	China	modern	0	Sino-Tibetan	28	103	Yi	N.A.	Patterson_et_al_2012	PASS
HGDP01180	Yi	sEA	China	modern	0	Sino-Tibetan	28	103	Yi	N.A.	Patterson_et_al_2012	PASS
HGDP01181	Yi	sEA	China	modern	0	Sino-Tibetan	28	103	Yi	N.A.	Patterson_et_al_2012	PASS
HGDP01182	Yi	sEA	China	modern	0	Sino-Tibetan	28	103	Yi	N.A.	Patterson_et_al_2012	PASS
HGDP01183	Yi	sEA	China	modern	0	Sino-Tibetan	28	103	Yi	N.A.	Patterson_et_al_2012	PASS
HGDP01184	Yi	sEA	China	modern	0	Sino-Tibetan	28	103	Yi	N.A.	Patterson_et_al_2012	PASS
HGDP01185	Yi	sEA	China	modern	0	Sino-Tibetan	28	103	Yi	N.A.	Patterson_et_al_2012	PASS
HGDP01186	Yi	sEA	China	modern	0	Sino-Tibetan	28	103	Yi	N.A.	Patterson_et_al_2012	PASS
HGDP01187	Yi	sEA	China	modern	0	Sino-Tibetan	28	103	Yi	N.A.	Patterson_et_al_2012	PASS
HGDP01188	Yi	sEA	China	modern	0	Sino-Tibetan	28	103	Yi	N.A.	Patterson_et_al_2012	PASS
HGDP01337	Naxi	sEA	China	modern	0	Sino-Tibetan	26	100	Naxi	N.A.	Patterson_et_al_2012	PASS
HGDP01338	Naxi	sEA	China	modern	0	Sino-Tibetan	26	100	Naxi	N.A.	Patterson_et_al_2012	PASS
HGDP01339	Naxi	sEA	China	modern	0	Sino-Tibetan	26	100	Naxi	N.A.	Patterson_et_al_2012	PASS
HGDP01340	Naxi	sEA	China	modern	0	Sino-Tibetan	26	100	Naxi	N.A.	Patterson_et_al_2012	PASS
HGDP01341	Naxi	sEA	China	modern	0	Sino-Tibetan	26	100	Naxi	N.A.	Patterson_et_al_2012	PASS
HGDP01342	Naxi	sEA	China	modern	0	Sino-Tibetan	26	100	Naxi	N.A.	Patterson_et_al_2012	PASS
HGDP01344	Naxi	sEA	China	modern	0	Sino-Tibetan	26	100	Naxi	N.A.	Patterson_et_al_2012	PASS
HGDP01345	Naxi	sEA	China	modern	0	Sino-Tibetan	26	100	Naxi	N.A.	Patterson_et_al_2012	PASS

HGDP01346	Naxi	sEA	China	modern	0	Sino-Tibetan	26	100	Naxi	N.A.	Patterson_et_al_2012	PASS
HGDP00774	Han	sEA	China	modern	0	Sino-Tibetan	32.27	114	Han	N.A.	Patterson_et_al_2012	PASS
HGDP00775	Han	sEA	China	modern	0	Sino-Tibetan	32.27	114	Han	N.A.	Patterson_et_al_2012	PASS
HGDP00776	Han	sEA	China	modern	0	Sino-Tibetan	32.27	114	Han	N.A.	Patterson_et_al_2012	PASS
HGDP00777	Han	sEA	China	modern	0	Sino-Tibetan	32.27	114	Han	N.A.	Patterson_et_al_2012	PASS
HGDP00779	Han	sEA	China	modern	0	Sino-Tibetan	32.27	114	Han	N.A.	Patterson_et_al_2012	PASS
HGDP00780	Han	sEA	China	modern	0	Sino-Tibetan	32.27	114	Han	N.A.	Patterson_et_al_2012	PASS
HGDP00781	Han	sEA	China	modern	0	Sino-Tibetan	32.27	114	Han	N.A.	Patterson_et_al_2012	PASS
HGDP00782	Han	sEA	China	modern	0	Sino-Tibetan	32.27	114	Han	N.A.	Patterson_et_al_2012	PASS
HGDP00783	Han	sEA	China	modern	0	Sino-Tibetan	32.27	114	Han	N.A.	Patterson_et_al_2012	PASS
HGDP00784	Han	sEA	China	modern	0	Sino-Tibetan	32.27	114	Han	N.A.	Patterson_et_al_2012	PASS
HGDP00785	Han	sEA	China	modern	0	Sino-Tibetan	32.27	114	Han	N.A.	Patterson_et_al_2012	PASS
HGDP00786	Han	sEA	China	modern	0	Sino-Tibetan	32.27	114	Han	N.A.	Patterson_et_al_2012	PASS
HGDP00811	Han	sEA	China	modern	0	Sino-Tibetan	32.27	114	Han	N.A.	Patterson_et_al_2012	PASS
HGDP00812	Han	sEA	China	modern	0	Sino-Tibetan	32.27	114	Han	N.A.	Patterson_et_al_2012	PASS
HGDP00813	Han	sEA	China	modern	0	Sino-Tibetan	32.27	114	Han	N.A.	Patterson_et_al_2012	PASS
HGDP00814	Han	sEA	China	modern	0	Sino-Tibetan	32.27	114	Han	N.A.	Patterson_et_al_2012	PASS
HGDP00815	Han	sEA	China	modern	0	Sino-Tibetan	32.27	114	Han	N.A.	Patterson_et_al_2012	PASS
HGDP00817	Han	sEA	China	modern	0	Sino-Tibetan	32.27	114	Han	N.A.	Patterson_et_al_2012	PASS
HGDP00818	Han	sEA	China	modern	0	Sino-Tibetan	32.27	114	Han	N.A.	Patterson_et_al_2012	PASS
HGDP00819	Han	sEA	China	modern	0	Sino-Tibetan	32.27	114	Han	N.A.	Patterson_et_al_2012	PASS
HGDP00820	Han	sEA	China	modern	0	Sino-Tibetan	32.27	114	Han	N.A.	Patterson_et_al_2012	PASS
HGDP00821	Han	sEA	China	modern	0	Sino-Tibetan	32.27	114	Han	N.A.	Patterson_et_al_2012	PASS
HGDP00822	Han	sEA	China	modern	0	Sino-Tibetan	32.27	114	Han	N.A.	Patterson_et_al_2012	PASS
HGDP00971	Han	sEA	China	modern	0	Sino-Tibetan	32.27	114	Han	N.A.	Patterson_et_al_2012	PASS
HGDP00972	Han	sEA	China	modern	0	Sino-Tibetan	32.27	114	Han	N.A.	Patterson_et_al_2012	PASS
HGDP00973	Han	sEA	China	modern	0	Sino-Tibetan	32.27	114	Han	N.A.	Patterson_et_al_2012	PASS
HGDP00974	Han	sEA	China	modern	0	Sino-Tibetan	32.27	114	Han	N.A.	Patterson_et_al_2012	PASS
HGDP00975	Han	sEA	China	modern	0	Sino-Tibetan	32.27	114	Han	N.A.	Patterson_et_al_2012	PASS
HGDP00976	Han	sEA	China	modern	0	Sino-Tibetan	32.27	114	Han	N.A.	Patterson_et_al_2012	PASS
HGDP00977	Han	sEA	China	modern	0	Sino-Tibetan	32.27	114	Han	N.A.	Patterson_et_al_2012	PASS
HGDP01021	Han	sEA	China	modern	0	Sino-Tibetan	32.27	114	Han	N.A.	Patterson_et_al_2012	PASS
HGDP01023	Han	sEA	China	modern	0	Sino-Tibetan	32.27	114	Han	N.A.	Patterson_et_al_2012	PASS
HGDP01024	Han	sEA	China	modern	0	Sino-Tibetan	32.27	114	Han	N.A.	Patterson_et_al_2012	PASS
HGDP01287	Han_NC	nEA	China	modern	0	Sino-Tibetan	32.27	114	Han	N.A.	Patterson_et_al_2012	PASS
HGDP01288	Han_NC	nEA	China	modern	0	Sino-Tibetan	32.27	114	Han	N.A.	Patterson_et_al_2012	PASS
HGDP01289	Han_NC	nEA	China	modern	0	Sino-Tibetan	32.27	114	Han	N.A.	Patterson_et_al_2012	PASS
HGDP01290	Han_NC	nEA	China	modern	0	Sino-Tibetan	32.27	114	Han	N.A.	Patterson_et_al_2012	PASS
HGDP01291	Han_NC	nEA	China	modern	0	Sino-Tibetan	32.27	114	Han	N.A.	Patterson_et_al_2012	PASS
HGDP01292	Han_NC	nEA	China	modern	0	Sino-Tibetan	32.27	114	Han	N.A.	Patterson_et_al_2012	PASS
HGDP01293	Han_NC	nEA	China	modern	0	Sino-Tibetan	32.27	114	Han	N.A.	Patterson_et_al_2012	PASS
HGDP01294	Han_NC	nEA	China	modern	0	Sino-Tibetan	32.27	114	Han	N.A.	Patterson_et_al_2012	PASS
HGDP01295	Han_NC	nEA	China	modern	0	Sino-Tibetan	32.27	114	Han	N.A.	Patterson_et_al_2012	PASS
HGDP01296	Han_NC	nEA	China	modern	0	Sino-Tibetan	32.27	114	Han	N.A.	Patterson_et_al_2012	PASS

HGDP01095	Tujia	sEA	China	modern	0	Sino-Tibetan	29	109	Tujia	N.A.	Patterson_et_al_2012	PASS
HGDP01096	Tujia	sEA	China	modern	0	Sino-Tibetan	29	109	Tujia	N.A.	Patterson_et_al_2012	PASS
HGDP01097	Tujia	sEA	China	modern	0	Sino-Tibetan	29	109	Tujia	N.A.	Patterson_et_al_2012	PASS
HGDP01098	Tujia	sEA	China	modern	0	Sino-Tibetan	29	109	Tujia	N.A.	Patterson_et_al_2012	PASS
HGDP01099	Tujia	sEA	China	modern	0	Sino-Tibetan	29	109	Tujia	N.A.	Patterson_et_al_2012	PASS
HGDP01100	Tujia	sEA	China	modern	0	Sino-Tibetan	29	109	Tujia	N.A.	Patterson_et_al_2012	PASS
HGDP01101	Tujia	sEA	China	modern	0	Sino-Tibetan	29	109	Tujia	N.A.	Patterson_et_al_2012	PASS
HGDP01102	Tujia	sEA	China	modern	0	Sino-Tibetan	29	109	Tujia	N.A.	Patterson_et_al_2012	PASS
HGDP01103	Tujia	sEA	China	modern	0	Sino-Tibetan	29	109	Tujia	N.A.	Patterson_et_al_2012	PASS
HGDP01104	Tujia	sEA	China	modern	0	Sino-Tibetan	29	109	Tujia	N.A.	Patterson_et_al_2012	PASS
HGDP01347	Tu	nEA	China	modern	0	Sino-Tibetan	36	101	Tu	N.A.	Patterson_et_al_2012	PASS
HGDP01348	Tu	nEA	China	modern	0	Sino-Tibetan	36	101	Tu	N.A.	Patterson_et_al_2012	PASS
HGDP01349	Tu	nEA	China	modern	0	Sino-Tibetan	36	101	Tu	N.A.	Patterson_et_al_2012	PASS
HGDP01350	Tu	nEA	China	modern	0	Sino-Tibetan	36	101	Tu	N.A.	Patterson_et_al_2012	PASS
HGDP01351	Tu	nEA	China	modern	0	Sino-Tibetan	36	101	Tu	N.A.	Patterson_et_al_2012	PASS
HGDP01352	Tu	nEA	China	modern	0	Sino-Tibetan	36	101	Tu	N.A.	Patterson_et_al_2012	PASS
HGDP01353	Tu	nEA	China	modern	0	Sino-Tibetan	36	101	Tu	N.A.	Patterson_et_al_2012	PASS
HGDP01354	Tu	nEA	China	modern	0	Sino-Tibetan	36	101	Tu	N.A.	Patterson_et_al_2012	PASS
HGDP01355	Tu	nEA	China	modern	0	Sino-Tibetan	36	101	Tu	N.A.	Patterson_et_al_2012	PASS
HGDP01356	Tu	nEA	China	modern	0	Sino-Tibetan	36	101	Tu	N.A.	Patterson_et_al_2012	PASS
HGDP01243	Xibo	nEA	China	modern	0	Tungusic	43.5	81.5	N.A.	N.A.	Patterson_et_al_2012	PASS
HGDP01244	Xibo	nEA	China	modern	0	Tungusic	43.5	81.5	N.A.	N.A.	Patterson_et_al_2012	PASS
HGDP01245	Xibo	nEA	China	modern	0	Tungusic	43.5	81.5	N.A.	N.A.	Patterson_et_al_2012	PASS
HGDP01246	Xibo	nEA	China	modern	0	Tungusic	43.5	81.5	N.A.	N.A.	Patterson_et_al_2012	PASS
HGDP01247	Xibo	nEA	China	modern	0	Tungusic	43.5	81.5	N.A.	N.A.	Patterson_et_al_2012	PASS
HGDP01248	Xibo	nEA	China	modern	0	Tungusic	43.5	81.5	N.A.	N.A.	Patterson_et_al_2012	PASS
HGDP01249	Xibo	nEA	China	modern	0	Tungusic	43.5	81.5	N.A.	N.A.	Patterson_et_al_2012	PASS
HGDP01250	Xibo	nEA	China	modern	0	Tungusic	43.5	81.5	N.A.	N.A.	Patterson_et_al_2012	PASS
HGDP01251	Xibo	nEA	China	modern	0	Tungusic	43.5	81.5	N.A.	N.A.	Patterson_et_al_2012	PASS
HGDP01233	Hezhen	nEA	China	modern	0	Tungusic	47.5	133.5	N.A.	N.A.	Patterson_et_al_2012	PASS
HGDP01234	Hezhen	nEA	China	modern	0	Tungusic	47.5	133.5	N.A.	N.A.	Patterson_et_al_2012	PASS
HGDP01236	Hezhen	nEA	China	modern	0	Tungusic	47.5	133.5	N.A.	N.A.	Patterson_et_al_2012	PASS
HGDP01237	Hezhen	nEA	China	modern	0	Tungusic	47.5	133.5	N.A.	N.A.	Patterson_et_al_2012	PASS
HGDP01238	Hezhen	nEA	China	modern	0	Tungusic	47.5	133.5	N.A.	N.A.	Patterson_et_al_2012	PASS
HGDP01239	Hezhen	nEA	China	modern	0	Tungusic	47.5	133.5	N.A.	N.A.	Patterson_et_al_2012	PASS
HGDP01240	Hezhen	nEA	China	modern	0	Tungusic	47.5	133.5	N.A.	N.A.	Patterson_et_al_2012	PASS
HGDP01241	Hezhen	nEA	China	modern	0	Tungusic	47.5	133.5	N.A.	N.A.	Patterson_et_al_2012	PASS
HGDP01242	Hezhen	nEA	China	modern	0	Tungusic	47.5	133.5	N.A.	N.A.	Patterson_et_al_2012	PASS
HGDP01203	Oroqen	nEA	China	modern	0	Tungusic	50.43	126.5	N.A.	N.A.	Patterson_et_al_2012	PASS
HGDP01204	Oroqen	nEA	China	modern	0	Tungusic	50.43	126.5	N.A.	N.A.	Patterson_et_al_2012	PASS
HGDP01205	Oroqen	nEA	China	modern	0	Tungusic	50.43	126.5	N.A.	N.A.	Patterson_et_al_2012	PASS
HGDP01206	Oroqen	nEA	China	modern	0	Tungusic	50.43	126.5	N.A.	N.A.	Patterson_et_al_2012	PASS
HGDP01207	Oroqen	nEA	China	modern	0	Tungusic	50.43	126.5	N.A.	N.A.	Patterson_et_al_2012	PASS
HGDP01208	Oroqen	nEA	China	modern	0	Tungusic	50.43	126.5	N.A.	N.A.	Patterson_et_al_2012	PASS

HGDP01209	Oroqen	nEA	China	modern	0	Tungusic	50.43	126.5	N.A.	N.A.	Patterson_et_al_ 2012	PASS
HGDP01211	Oroqen	nEA	China	modern	0	Tungusic	50.43	126.5	N.A.	N.A.	Patterson_et_al_ 2012	PASS
HGDP01212	Oroqen	nEA	China	modern	0	Tungusic	50.43	126.5	N.A.	N.A.	Patterson_et_al_ 2012	PASS
HGDP01297	Uygur	nEA	China	modern	0	Turkic	44	81	N.A.	N.A.	Patterson_et_al_ 2012	PASS
HGDP01298	Uygur	nEA	China	modern	0	Turkic	44	81	N.A.	N.A.	Patterson_et_al_ 2012	PASS
HGDP01299	Uygur	nEA	China	modern	0	Turkic	44	81	N.A.	N.A.	Patterson_et_al_ 2012	PASS
HGDP01300	Uygur	nEA	China	modern	0	Turkic	44	81	N.A.	N.A.	Patterson_et_al_ 2012	PASS
HGDP01301	Uygur	nEA	China	modern	0	Turkic	44	81	N.A.	N.A.	Patterson_et_al_ 2012	PASS
HGDP01302	Uygur	nEA	China	modern	0	Turkic	44	81	N.A.	N.A.	Patterson_et_al_ 2012	PASS
HGDP01303	Uygur	nEA	China	modern	0	Turkic	44	81	N.A.	N.A.	Patterson_et_al_ 2012	PASS
HGDP01304	Uygur	nEA	China	modern	0	Turkic	44	81	N.A.	N.A.	Patterson_et_al_ 2012	PASS
HGDP01305	Uygur	nEA	China	modern	0	Turkic	44	81	N.A.	N.A.	Patterson_et_al_ 2012	PASS
HGDP01306	Uygur	nEA	China	modern	0	Turkic	44	81	N.A.	N.A.	Patterson_et_al_ 2012	PASS
HGDP01213	Daur	nEA	China	modern	0	Mongolic	48.5	124	N.A.	N.A.	Patterson_et_al_ 2012	PASS
HGDP01214	Daur	nEA	China	modern	0	Mongolic	48.5	124	N.A.	N.A.	Patterson_et_al_ 2012	PASS
HGDP01215	Daur	nEA	China	modern	0	Mongolic	48.5	124	N.A.	N.A.	Patterson_et_al_ 2012	PASS
HGDP01216	Daur	nEA	China	modern	0	Mongolic	48.5	124	N.A.	N.A.	Patterson_et_al_ 2012	PASS
HGDP01217	Daur	nEA	China	modern	0	Mongolic	48.5	124	N.A.	N.A.	Patterson_et_al_ 2012	PASS
HGDP01218	Daur	nEA	China	modern	0	Mongolic	48.5	124	N.A.	N.A.	Patterson_et_al_ 2012	PASS
HGDP01220	Daur	nEA	China	modern	0	Mongolic	48.5	124	N.A.	N.A.	Patterson_et_al_ 2012	PASS
HGDP01221	Daur	nEA	China	modern	0	Mongolic	48.5	124	N.A.	N.A.	Patterson_et_al_ 2012	PASS
HGDP01222	Daur	nEA	China	modern	0	Mongolic	48.5	124	N.A.	N.A.	Patterson_et_al_ 2012	PASS
HGDP01223	Mongola	nEA	Mongolia	modern	0	Mongolic	45	111	N.A.	N.A.	Patterson_et_al_ 2012	PASS
HGDP01224	Mongola	nEA	Mongolia	modern	0	Mongolic	45	111	N.A.	N.A.	Patterson_et_al_ 2012	PASS
HGDP01225	Mongola	nEA	Mongolia	modern	0	Mongolic	45	111	N.A.	N.A.	Patterson_et_al_ 2012	PASS
HGDP01226	Mongola	nEA	Mongolia	modern	0	Mongolic	45	111	N.A.	N.A.	Patterson_et_al_ 2012	PASS
HGDP01227	Mongola	nEA	Mongolia	modern	0	Mongolic	45	111	N.A.	N.A.	Patterson_et_al_ 2012	PASS
HGDP01228	Mongola	nEA	Mongolia	modern	0	Mongolic	45	111	N.A.	N.A.	Patterson_et_al_ 2012	PASS
HGDP01229	Mongola	nEA	Mongolia	modern	0	Mongolic	45	111	N.A.	N.A.	Patterson_et_al_ 2012	PASS
HGDP01230	Mongola	nEA	Mongolia	modern	0	Mongolic	45	111	N.A.	N.A.	Patterson_et_al_ 2012	PASS
HGDP01231	Mongola	nEA	Mongolia	modern	0	Mongolic	45	111	N.A.	N.A.	Patterson_et_al_ 2012	PASS
HGDP01232	Mongola	nEA	Mongolia	modern	0	Mongolic	45	111	N.A.	N.A.	Patterson_et_al_ 2012	PASS
HGDP00747	Japanese	nEA	Japan	modern	0	Japonic	38	138	N.A.	N.A.	Patterson_et_al_ 2012	PASS
HGDP00748	Japanese	nEA	Japan	modern	0	Japonic	38	138	N.A.	N.A.	Patterson_et_al_ 2012	PASS
HGDP00749	Japanese	nEA	Japan	modern	0	Japonic	38	138	N.A.	N.A.	Patterson_et_al_ 2012	PASS
HGDP00750	Japanese	nEA	Japan	modern	0	Japonic	38	138	N.A.	N.A.	Patterson_et_al_ 2012	PASS
HGDP00751	Japanese	nEA	Japan	modern	0	Japonic	38	138	N.A.	N.A.	Patterson_et_al_ 2012	PASS
HGDP00752	Japanese	nEA	Japan	modern	0	Japonic	38	138	N.A.	N.A.	Patterson_et_al_ 2012	PASS
HGDP00753	Japanese	nEA	Japan	modern	0	Japonic	38	138	N.A.	N.A.	Patterson_et_al_ 2012	PASS
HGDP00754	Japanese	nEA	Japan	modern	0	Japonic	38	138	N.A.	N.A.	Patterson_et_al_ 2012	PASS
HGDP00755	Japanese	nEA	Japan	modern	0	Japonic	38	138	N.A.	N.A.	Patterson_et_al_ 2012	PASS
HGDP00756	Japanese	nEA	Japan	modern	0	Japonic	38	138	N.A.	N.A.	Patterson_et_al_ 2012	PASS
HGDP00757	Japanese	nEA	Japan	modern	0	Japonic	38	138	N.A.	N.A.	Patterson_et_al_ 2012	PASS
HGDP00758	Japanese	nEA	Japan	modern	0	Japonic	38	138	N.A.	N.A.	Patterson_et_al_ 2012	PASS

HGDP00759	Japanese	nEA	Japan	modern	0	Japonic	38	138	N.A.	N.A.	Patterson_et_al_2012	PASS
HGDP00760	Japanese	nEA	Japan	modern	0	Japonic	38	138	N.A.	N.A.	Patterson_et_al_2012	PASS
HGDP00761	Japanese	nEA	Japan	modern	0	Japonic	38	138	N.A.	N.A.	Patterson_et_al_2012	PASS
HGDP00762	Japanese	nEA	Japan	modern	0	Japonic	38	138	N.A.	N.A.	Patterson_et_al_2012	PASS
HGDP00763	Japanese	nEA	Japan	modern	0	Japonic	38	138	N.A.	N.A.	Patterson_et_al_2012	PASS
HGDP00764	Japanese	nEA	Japan	modern	0	Japonic	38	138	N.A.	N.A.	Patterson_et_al_2012	PASS
HGDP00765	Japanese	nEA	Japan	modern	0	Japonic	38	138	N.A.	N.A.	Patterson_et_al_2012	PASS
HGDP00766	Japanese	nEA	Japan	modern	0	Japonic	38	138	N.A.	N.A.	Patterson_et_al_2012	PASS
HGDP00767	Japanese	nEA	Japan	modern	0	Japonic	38	138	N.A.	N.A.	Patterson_et_al_2012	PASS
HGDP00768	Japanese	nEA	Japan	modern	0	Japonic	38	138	N.A.	N.A.	Patterson_et_al_2012	PASS
HGDP00769	Japanese	nEA	Japan	modern	0	Japonic	38	138	N.A.	N.A.	Patterson_et_al_2012	PASS
HGDP00771	Japanese	nEA	Japan	modern	0	Japonic	38	138	N.A.	N.A.	Patterson_et_al_2012	PASS
HGDP00772	Japanese	nEA	Japan	modern	0	Japonic	38	138	N.A.	N.A.	Patterson_et_al_2012	PASS
HGDP00773	Japanese	nEA	Japan	modern	0	Japonic	38	138	N.A.	N.A.	Patterson_et_al_2012	PASS
HGDP00790	Japanese	nEA	Japan	modern	0	Japonic	38	138	N.A.	N.A.	Patterson_et_al_2012	PASS
HGDP00791	Japanese	nEA	Japan	modern	0	Japonic	38	138	N.A.	N.A.	Patterson_et_al_2012	PASS
HGDP00828	Japanese	nEA	Japan	modern	0	Japonic	38	138	N.A.	N.A.	Patterson_et_al_2012	PASS
BAO60	Bonan	nEA	China	modern	0	Mongolic	35.7	102.8	N.A.	N.A.	Wang_C_et_al_2021	PASS
BAO53	Bonan	nEA	China	modern	0	Mongolic	35.7	102.8	N.A.	N.A.	Wang_C_et_al_2021	PASS
BAO15	Bonan	nEA	China	modern	0	Mongolic	35.7	102.8	N.A.	N.A.	Wang_C_et_al_2021	PASS
BAO12	Bonan	nEA	China	modern	0	Mongolic	35.7	102.8	N.A.	N.A.	Wang_C_et_al_2021	PASS
BAO26	Bonan	nEA	China	modern	0	Mongolic	35.7	102.8	N.A.	N.A.	Wang_C_et_al_2021	PASS
BAO47	Bonan	nEA	China	modern	0	Mongolic	35.7	102.8	N.A.	N.A.	Wang_C_et_al_2021	PASS
BAO54	Bonan	nEA	China	modern	0	Mongolic	35.7	102.8	N.A.	N.A.	Wang_C_et_al_2021	PASS
BAO22	Bonan	nEA	China	modern	0	Mongolic	35.7	102.8	N.A.	N.A.	Wang_C_et_al_2021	PASS
BAO11	Bonan	nEA	China	modern	0	Mongolic	35.7	102.8	N.A.	N.A.	Wang_C_et_al_2021	PASS
BAO03	Bonan	nEA	China	modern	0	Mongolic	35.7	102.8	N.A.	N.A.	Wang_C_et_al_2021	kin
DX1157	Dongxiang	nEA	China	modern	0	Mongolic	35.6	103.2	N.A.	N.A.	Wang_C_et_al_2021	PASS
DX1175	Dongxiang	nEA	China	modern	0	Mongolic	35.6	103.2	N.A.	N.A.	Wang_C_et_al_2021	PASS
DX1170	Dongxiang	nEA	China	modern	0	Mongolic	35.6	103.2	N.A.	N.A.	Wang_C_et_al_2021	PASS
DX1047	Dongxiang	nEA	China	modern	0	Mongolic	35.6	103.2	N.A.	N.A.	Wang_C_et_al_2021	PASS
DX1146	Dongxiang	nEA	China	modern	0	Mongolic	35.6	103.2	N.A.	N.A.	Wang_C_et_al_2021	PASS
DX1058	Dongxiang	nEA	China	modern	0	Mongolic	35.6	103.2	N.A.	N.A.	Wang_C_et_al_2021	PASS
DX1131	Dongxiang	nEA	China	modern	0	Mongolic	35.6	103.2	N.A.	N.A.	Wang_C_et_al_2021	PASS
EYG48	Yugur	nEA	China	modern	0	Mongolic	38.9	99.6	N.A.	N.A.	Wang_C_et_al_2021	PASS
EYG28	Yugur	nEA	China	modern	0	Mongolic	38.9	99.6	N.A.	N.A.	Wang_C_et_al_2021	PASS
EYG13	Yugur	nEA	China	modern	0	Mongolic	38.9	99.6	N.A.	N.A.	Wang_C_et_al_2021	PASS
EYG18	Yugur	nEA	China	modern	0	Mongolic	38.9	99.6	N.A.	N.A.	Wang_C_et_al_2021	PASS
EYG31	Yugur	nEA	China	modern	0	Mongolic	38.9	99.6	N.A.	N.A.	Wang_C_et_al_2021	PASS
XYG19	Yugur	nEA	China	modern	0	Mongolic	38.9	99.6	N.A.	N.A.	Wang_C_et_al_2021	PASS
EYG8	Yugur	nEA	China	modern	0	Mongolic	38.9	99.6	N.A.	N.A.	Wang_C_et_al_2021	PASS
EYG04	Yugur	nEA	China	modern	0	Mongolic	38.9	99.6	N.A.	N.A.	Wang_C_et_al_2021	PASS
EYG45	Yugur	nEA	China	modern	0	Mongolic	38.9	99.6	N.A.	N.A.	Wang_C_et_al_2021	PASS
EYG44	Yugur	nEA	China	modern	0	Mongolic	38.9	99.6	N.A.	N.A.	Wang_C_et_al_2021	PASS

EYG40	Yugur	nEA	China	modern	0	Mongolic	38.9	99.6	N.A.	N.A.	Wang_C_et_al_2021	PASS
EYG06	Yugur	nEA	China	modern	0	Mongolic	38.9	99.6	N.A.	N.A.	Wang_C_et_al_2021	PASS
EYG25	Yugur	nEA	China	modern	0	Mongolic	38.9	99.6	N.A.	N.A.	Wang_C_et_al_2021	PASS
EYG42	Yugur	nEA	China	modern	0	Mongolic	38.9	99.6	N.A.	N.A.	Wang_C_et_al_2021	PASS
EYG46	Yugur	nEA	China	modern	0	Mongolic	38.9	99.6	N.A.	N.A.	Wang_C_et_al_2021	PASS
EYG24	Yugur	nEA	China	modern	0	Mongolic	38.9	99.6	N.A.	N.A.	Wang_C_et_al_2021	PASS
Han516	Han_Chongqing	sEA	China	modern	0	Sino-Tibetan	29.3	106.3	Han	N.A.	Wang_C_et_al_2021	PASS
Han1968	Han_Chongqing	sEA	China	modern	0	Sino-Tibetan	29.3	106.3	Han	N.A.	Wang_C_et_al_2021	PASS
Han2150	Han_Chongqing	sEA	China	modern	0	Sino-Tibetan	29.3	106.3	Han	N.A.	Wang_C_et_al_2021	PASS
Han1994	Han_Fujian	sEA	China	modern	0	Sino-Tibetan	26.1	119.3	Han	N.A.	Wang_C_et_al_2021	PASS
Han1467	Han_Fujian	sEA	China	modern	0	Sino-Tibetan	26.1	119.3	Han	N.A.	Wang_C_et_al_2021	PASS
Han1619	Han_Fujian	sEA	China	modern	0	Sino-Tibetan	26.1	119.3	Han	N.A.	Wang_C_et_al_2021	PASS
Han1900	Han_Fujian	sEA	China	modern	0	Sino-Tibetan	26.1	119.3	Han	N.A.	Wang_C_et_al_2021	PASS
Han1934	Han_Fujian	sEA	China	modern	0	Sino-Tibetan	26.1	119.3	Han	N.A.	Wang_C_et_al_2021	PASS
Han1207	Han_Guangdong	sEA	China	modern	0	Sino-Tibetan	23.2	113.2	Han	N.A.	Wang_C_et_al_2021	PASS
Han1235	Han_Guangdong	sEA	China	modern	0	Sino-Tibetan	23.2	113.2	Han	N.A.	Wang_C_et_al_2021	PASS
Han1415	Han_Guangdong	sEA	China	modern	0	Sino-Tibetan	23.2	113.2	Han	N.A.	Wang_C_et_al_2021	PASS
Han1432	Han_Guangdong	sEA	China	modern	0	Sino-Tibetan	23.2	113.2	Han	N.A.	Wang_C_et_al_2021	PASS
Han1434	Han_Guangdong	sEA	China	modern	0	Sino-Tibetan	23.2	113.2	Han	N.A.	Wang_C_et_al_2021	PASS
Han2087	Han_Guangdong	sEA	China	modern	0	Sino-Tibetan	23.2	113.2	Han	N.A.	Wang_C_et_al_2021	PASS
Han2088	Han_Guangdong	sEA	China	modern	0	Sino-Tibetan	23.2	113.2	Han	N.A.	Wang_C_et_al_2021	PASS
Han894	Han_Henan	nEA	China	modern	0	Sino-Tibetan	34.8	113.6	Han	N.A.	Wang_C_et_al_2021	PASS
Han1226	Han_Henan	nEA	China	modern	0	Sino-Tibetan	34.8	113.6	Han	N.A.	Wang_C_et_al_2021	PASS
Han1243	Han_Henan	nEA	China	modern	0	Sino-Tibetan	34.8	113.6	Han	N.A.	Wang_C_et_al_2021	PASS
Han1713	Han_Henan	nEA	China	modern	0	Sino-Tibetan	34.8	113.6	Han	N.A.	Wang_C_et_al_2021	PASS
Han1714	Han_Henan	nEA	China	modern	0	Sino-Tibetan	34.8	113.6	Han	N.A.	Wang_C_et_al_2021	PASS
Han788	Han_Hubei	sEA	China	modern	0	Sino-Tibetan	30.5	114.3	Han	N.A.	Wang_C_et_al_2021	PASS
Han789	Han_Hubei	sEA	China	modern	0	Sino-Tibetan	30.5	114.3	Han	N.A.	Wang_C_et_al_2021	PASS
Han874	Han_Hubei	sEA	China	modern	0	Sino-Tibetan	30.5	114.3	Han	N.A.	Wang_C_et_al_2021	PASS
Han2161	Han_Hubei	sEA	China	modern	0	Sino-Tibetan	30.5	114.3	Han	N.A.	Wang_C_et_al_2021	PASS
Han2162	Han_Hubei	sEA	China	modern	0	Sino-Tibetan	30.5	114.3	Han	N.A.	Wang_C_et_al_2021	PASS
Han1452	Han_Jiangsu	sEA	China	modern	0	Sino-Tibetan	32.1	118.8	Han	N.A.	Wang_C_et_al_2021	PASS
Han1962	Han_Jiangsu	sEA	China	modern	0	Sino-Tibetan	32.1	118.8	Han	N.A.	Wang_C_et_al_2021	PASS
Han2054	Han_Jiangsu	sEA	China	modern	0	Sino-Tibetan	32.1	118.8	Han	N.A.	Wang_C_et_al_2021	PASS
Han2057	Han_Jiangsu	sEA	China	modern	0	Sino-Tibetan	32.1	118.8	Han	N.A.	Wang_C_et_al_2021	PASS
Han2058	Han_Jiangsu	sEA	China	modern	0	Sino-Tibetan	32.1	118.8	Han	N.A.	Wang_C_et_al_2021	PASS
Han2076	Han_Jiangsu	sEA	China	modern	0	Sino-Tibetan	32.1	118.8	Han	N.A.	Wang_C_et_al_2021	PASS
Han2090	Han_Jiangsu	sEA	China	modern	0	Sino-Tibetan	32.1	118.8	Han	N.A.	Wang_C_et_al_2021	PASS
Han4	Han_Shandong	nEA	China	modern	0	Sino-Tibetan	36.6	117	Han	N.A.	Wang_C_et_al_2021	PASS
Han25	Han_Shandong	nEA	China	modern	0	Sino-Tibetan	36.6	117	Han	N.A.	Wang_C_et_al_2021	PASS
Han853	Han_Shandong	nEA	China	modern	0	Sino-Tibetan	36.6	117	Han	N.A.	Wang_C_et_al_2021	PASS
Han1329	Han_Shandong	nEA	China	modern	0	Sino-Tibetan	36.6	117	Han	N.A.	Wang_C_et_al_2021	PASS
Han1616	Han_Shandong	nEA	China	modern	0	Sino-Tibetan	36.6	117	Han	N.A.	Wang_C_et_al_2021	PASS
Han1840	Han_Shandong	nEA	China	modern	0	Sino-Tibetan	36.6	117	Han	N.A.	Wang_C_et_al_2021	PASS

Han1896	Han_Shandong	nEA	China	modern	0	Sino-Tibetan	36.6	117	Han	N.A.	Wang_C_et_al_2021	PASS
Han1916	Han_Shandong	nEA	China	modern	0	Sino-Tibetan	36.6	117	Han	N.A.	Wang_C_et_al_2021	PASS
Han1978	Han_Shandong	nEA	China	modern	0	Sino-Tibetan	36.6	117	Han	N.A.	Wang_C_et_al_2021	PASS
Han220	Han_Shandong	nEA	China	modern	0	Sino-Tibetan	36.6	117	Han	N.A.	Wang_C_et_al_2021	PASS
Han24	Han_Shanghai	sEA	China	modern	0	Sino-Tibetan	31.2	121.5	Han	N.A.	Wang_C_et_al_2021	PASS
Han496	Han_Shanghai	sEA	China	modern	0	Sino-Tibetan	31.2	121.5	Han	N.A.	Wang_C_et_al_2021	PASS
Han115	Han_Shanxi	nEA	China	modern	0	Sino-Tibetan	37.9	112.5	Han	N.A.	Wang_C_et_al_2021	PASS
Han790	Han_Shanxi	nEA	China	modern	0	Sino-Tibetan	37.9	112.5	Han	N.A.	Wang_C_et_al_2021	PASS
Han855	Han_Shanxi	nEA	China	modern	0	Sino-Tibetan	37.9	112.5	Han	N.A.	Wang_C_et_al_2021	PASS
Han1603	Han_Shanxi	nEA	China	modern	0	Sino-Tibetan	37.9	112.5	Han	N.A.	Wang_C_et_al_2021	PASS
Han1843	Han_Shanxi	nEA	China	modern	0	Sino-Tibetan	37.9	112.5	Han	N.A.	Wang_C_et_al_2021	PASS
Han1897	Han_Shanxi	nEA	China	modern	0	Sino-Tibetan	37.9	112.5	Han	N.A.	Wang_C_et_al_2021	PASS
Han1899	Han_Shanxi	nEA	China	modern	0	Sino-Tibetan	37.9	112.5	Han	N.A.	Wang_C_et_al_2021	PASS
Han1970	Han_Shanxi	nEA	China	modern	0	Sino-Tibetan	37.9	112.5	Han	N.A.	Wang_C_et_al_2021	PASS
Han1709	Han_Sichuan	sEA	China	modern	0	Sino-Tibetan	30.7	104.1	Han	N.A.	Wang_C_et_al_2021	PASS
Han1917	Han_Sichuan	sEA	China	modern	0	Sino-Tibetan	30.7	104.1	Han	N.A.	Wang_C_et_al_2021	PASS
Han2044	Han_Sichuan	sEA	China	modern	0	Sino-Tibetan	30.7	104.1	Han	N.A.	Wang_C_et_al_2021	PASS
Han2145	Han_Sichuan	sEA	China	modern	0	Sino-Tibetan	30.7	104.1	Han	N.A.	Wang_C_et_al_2021	PASS
Han2147	Han_Sichuan	sEA	China	modern	0	Sino-Tibetan	30.7	104.1	Han	N.A.	Wang_C_et_al_2021	PASS
Han2151	Han_Sichuan	sEA	China	modern	0	Sino-Tibetan	30.7	104.1	Han	N.A.	Wang_C_et_al_2021	PASS
Han2155	Han_Sichuan	sEA	China	modern	0	Sino-Tibetan	30.7	104.1	Han	N.A.	Wang_C_et_al_2021	PASS
Han53	Han_Zhejiang	sEA	China	modern	0	Sino-Tibetan	30.3	120.2	Han	N.A.	Wang_C_et_al_2021	PASS
Han192	Han_Zhejiang	sEA	China	modern	0	Sino-Tibetan	30.3	120.2	Han	N.A.	Wang_C_et_al_2021	PASS
Han278	Han_Zhejiang	sEA	China	modern	0	Sino-Tibetan	30.3	120.2	Han	N.A.	Wang_C_et_al_2021	PASS
Han605	Han_Zhejiang	sEA	China	modern	0	Sino-Tibetan	30.3	120.2	Han	N.A.	Wang_C_et_al_2021	PASS
Han993	Han_Zhejiang	sEA	China	modern	0	Sino-Tibetan	30.3	120.2	Han	N.A.	Wang_C_et_al_2021	PASS
DBB006	Qiang_Danba	sEA	China	modern	0	Sino-Tibetan	30.8	101.9	Qiang	N.A.	Wang_C_et_al_2021	PASS
DBA19	Qiang_Danba	sEA	China	modern	0	Sino-Tibetan	30.8	101.9	Qiang	N.A.	Wang_C_et_al_2021	PASS
DBA021	Qiang_Danba	sEA	China	modern	0	Sino-Tibetan	30.8	101.9	Qiang	N.A.	Wang_C_et_al_2021	PASS
DBB007	Qiang_Danba	sEA	China	modern	0	Sino-Tibetan	30.8	101.9	Qiang	N.A.	Wang_C_et_al_2021	PASS
DBA017	Qiang_Danba	sEA	China	modern	0	Sino-Tibetan	30.8	101.9	Qiang	N.A.	Wang_C_et_al_2021	PASS
DBA018	Qiang_Danba	sEA	China	modern	0	Sino-Tibetan	30.8	101.9	Qiang	N.A.	Wang_C_et_al_2021	PASS
DBB001	Qiang_Danba	sEA	China	modern	0	Sino-Tibetan	30.8	101.9	Qiang	N.A.	Wang_C_et_al_2021	PASS
DBA01	Qiang_Danba	sEA	China	modern	0	Sino-Tibetan	30.8	101.9	Qiang	N.A.	Wang_C_et_al_2021	PASS
DBB002	Qiang_Danba	sEA	China	modern	0	Sino-Tibetan	30.8	101.9	Qiang	N.A.	Wang_C_et_al_2021	PASS
BM32	Qiang_Daofu	sEA	China	modern	0	Sino-Tibetan	30.9	101.1	Qiang	N.A.	Wang_C_et_al_2021	PASS
BM10	Qiang_Daofu	sEA	China	modern	0	Sino-Tibetan	30.9	101.1	Qiang	N.A.	Wang_C_et_al_2021	PASS
BM43	Qiang_Daofu	sEA	China	modern	0	Sino-Tibetan	30.9	101.1	Qiang	N.A.	Wang_C_et_al_2021	PASS
BM24	Qiang_Daofu	sEA	China	modern	0	Sino-Tibetan	30.9	101.1	Qiang	N.A.	Wang_C_et_al_2021	PASS
BM26	Qiang_Daofu	sEA	China	modern	0	Sino-Tibetan	30.9	101.1	Qiang	N.A.	Wang_C_et_al_2021	PASS
BM21	Qiang_Daofu	sEA	China	modern	0	Sino-Tibetan	30.9	101.1	Qiang	N.A.	Wang_C_et_al_2021	PASS
BM28	Qiang_Daofu	sEA	China	modern	0	Sino-Tibetan	30.9	101.1	Qiang	N.A.	Wang_C_et_al_2021	PASS
BM37	Qiang_Daofu	sEA	China	modern	0	Sino-Tibetan	30.9	101.1	Qiang	N.A.	Wang_C_et_al_2021	PASS
BM27	Qiang_Daofu	sEA	China	modern	0	Sino-Tibetan	30.9	101.1	Qiang	N.A.	Wang_C_et_al_2021	PASS

BM02	Qiang_Daofu	sEA	China	modern	0	Sino-Tibetan	30.9	101.1	Qiang	N.A.	Wang_C_et_al_2021	PASS
BM18	Qiang_Daofu	sEA	China	modern	0	Sino-Tibetan	30.9	101.1	Qiang	N.A.	Wang_C_et_al_2021	PASS
T141	Tibetan_Chamdo	Tibet	China	modern	0	Sino-Tibetan	31.1	97.2	Tibetan	N.A.	Wang_C_et_al_2021	PASS
T173	Tibetan_Chamdo	Tibet	China	modern	0	Sino-Tibetan	31.1	97.2	Tibetan	N.A.	Wang_C_et_al_2021	PASS
T274	Tibetan_Chamdo	Tibet	China	modern	0	Sino-Tibetan	31.1	97.2	Tibetan	N.A.	Wang_C_et_al_2021	PASS
T363	Tibetan_Chamdo	Tibet	China	modern	0	Sino-Tibetan	31.1	97.2	Tibetan	N.A.	Wang_C_et_al_2021	PASS
T247	Tibetan_Chamdo	Tibet	China	modern	0	Sino-Tibetan	31.1	97.2	Tibetan	N.A.	Wang_C_et_al_2021	PASS
T74	Tibetan_Chamdo	Tibet	China	modern	0	Sino-Tibetan	31.1	97.2	Tibetan	N.A.	Wang_C_et_al_2021	PASS
T144	Tibetan_Chamdo	Tibet	China	modern	0	Sino-Tibetan	31.1	97.2	Tibetan	N.A.	Wang_C_et_al_2021	PASS
T194	Tibetan_Chamdo	Tibet	China	modern	0	Sino-Tibetan	31.1	97.2	Tibetan	N.A.	Wang_C_et_al_2021	PASS
T196	Tibetan_Chamdo	Tibet	China	modern	0	Sino-Tibetan	31.1	97.2	Tibetan	N.A.	Wang_C_et_al_2021	PASS
T273	Tibetan_Chamdo	Tibet	China	modern	0	Sino-Tibetan	31.1	97.2	Tibetan	N.A.	Wang_C_et_al_2021	PASS
T282	Tibetan_Chamdo	Tibet	China	modern	0	Sino-Tibetan	31.1	97.2	Tibetan	N.A.	Wang_C_et_al_2021	PASS
T283	Tibetan_Chamdo	Tibet	China	modern	0	Sino-Tibetan	31.1	97.2	Tibetan	N.A.	Wang_C_et_al_2021	PASS
QH1	Tibetan_Gangcha	Tibet	China	modern	0	Sino-Tibetan	37.3	100.2	Tibetan	N.A.	Wang_C_et_al_2021	PASS
QH5	Tibetan_Gangcha	Tibet	China	modern	0	Sino-Tibetan	37.3	100.2	Tibetan	N.A.	Wang_C_et_al_2021	PASS
QH7	Tibetan_Gangcha	Tibet	China	modern	0	Sino-Tibetan	37.3	100.2	Tibetan	N.A.	Wang_C_et_al_2021	PASS
QH18	Tibetan_Gangcha	Tibet	China	modern	0	Sino-Tibetan	37.3	100.2	Tibetan	N.A.	Wang_C_et_al_2021	PASS
QH10	Tibetan_Gangcha	Tibet	China	modern	0	Sino-Tibetan	37.3	100.2	Tibetan	N.A.	Wang_C_et_al_2021	PASS
QH9	Tibetan_Gangcha	Tibet	China	modern	0	Sino-Tibetan	37.3	100.2	Tibetan	N.A.	Wang_C_et_al_2021	PASS
QH14	Tibetan_Gangcha	Tibet	China	modern	0	Sino-Tibetan	37.3	100.2	Tibetan	N.A.	Wang_C_et_al_2021	PASS
QH17	Tibetan_Gangcha	Tibet	China	modern	0	Sino-Tibetan	37.3	100.2	Tibetan	N.A.	Wang_C_et_al_2021	PASS
QH2	Tibetan_Gangcha	Tibet	China	modern	0	Sino-Tibetan	37.3	100.2	Tibetan	N.A.	Wang_C_et_al_2021	PASS
QH3	Tibetan_Gangcha	Tibet	China	modern	0	Sino-Tibetan	37.3	100.2	Tibetan	N.A.	Wang_C_et_al_2021	PASS
QH4	Tibetan_Gangcha	Tibet	China	modern	0	Sino-Tibetan	37.3	100.2	Tibetan	N.A.	Wang_C_et_al_2021	PASS
QH8	Tibetan_Gangcha	Tibet	China	modern	0	Sino-Tibetan	37.3	100.2	Tibetan	N.A.	Wang_C_et_al_2021	PASS
QH11	Tibetan_Gangcha	Tibet	China	modern	0	Sino-Tibetan	37.3	100.2	Tibetan	N.A.	Wang_C_et_al_2021	PASS
QH13	Tibetan_Gangcha	Tibet	China	modern	0	Sino-Tibetan	37.3	100.2	Tibetan	N.A.	Wang_C_et_al_2021	PASS
QH15	Tibetan_Gangcha	Tibet	China	modern	0	Sino-Tibetan	37.3	100.2	Tibetan	N.A.	Wang_C_et_al_2021	PASS
QH16	Tibetan_Gangcha	Tibet	China	modern	0	Sino-Tibetan	37.3	100.2	Tibetan	N.A.	Wang_C_et_al_2021	PASS
QH19	Tibetan_Gangcha	Tibet	China	modern	0	Sino-Tibetan	37.3	100.2	Tibetan	N.A.	Wang_C_et_al_2021	PASS
QH20	Tibetan_Gangcha	Tibet	China	modern	0	Sino-Tibetan	37.3	100.2	Tibetan	N.A.	Wang_C_et_al_2021	PASS
QH6	Tibetan_Gangcha	Tibet	China	modern	0	Sino-Tibetan	37.3	100.2	Tibetan	N.A.	Wang_C_et_al_2021	PASS
QH12	Tibetan_Gangcha	Tibet	China	modern	0	Sino-Tibetan	37.3	100.2	Tibetan	N.A.	Wang_C_et_al_2021	PASS
GN01	Tibetan_Gannan	Tibet	China	modern	0	Sino-Tibetan	35	102.9	Tibetan	N.A.	Wang_C_et_al_2021	PASS
GN03	Tibetan_Gannan	Tibet	China	modern	0	Sino-Tibetan	35	102.9	Tibetan	N.A.	Wang_C_et_al_2021	PASS
GN10	Tibetan_Gannan	Tibet	China	modern	0	Sino-Tibetan	35	102.9	Tibetan	N.A.	Wang_C_et_al_2021	PASS
GN17	Tibetan_Gannan	Tibet	China	modern	0	Sino-Tibetan	35	102.9	Tibetan	N.A.	Wang_C_et_al_2021	PASS
GN19	Tibetan_Gannan	Tibet	China	modern	0	Sino-Tibetan	35	102.9	Tibetan	N.A.	Wang_C_et_al_2021	PASS
T21	Tibetan_Lhasa	Tibet	China	modern	0	Sino-Tibetan	30	91.1	Tibetan	N.A.	Wang_C_et_al_2021	PASS
T165	Tibetan_Lhasa	Tibet	China	modern	0	Sino-Tibetan	30	91.1	Tibetan	N.A.	Wang_C_et_al_2021	PASS
T170	Tibetan_Lhasa	Tibet	China	modern	0	Sino-Tibetan	30	91.1	Tibetan	N.A.	Wang_C_et_al_2021	PASS
T181	Tibetan_Lhasa	Tibet	China	modern	0	Sino-Tibetan	30	91.1	Tibetan	N.A.	Wang_C_et_al_2021	PASS
T183	Tibetan_Lhasa	Tibet	China	modern	0	Sino-Tibetan	30	91.1	Tibetan	N.A.	Wang_C_et_al_2021	PASS

T76	Tibetan_Lhasa	Tibet	China	modern	0	Sino-Tibetan	30	91.1	Tibetan	N.A.	Wang_C_et_al_2021	PASS
T125	Tibetan_Lhasa	Tibet	China	modern	0	Sino-Tibetan	30	91.1	Tibetan	N.A.	Wang_C_et_al_2021	PASS
T185	Tibetan_Lhasa	Tibet	China	modern	0	Sino-Tibetan	30	91.1	Tibetan	N.A.	Wang_C_et_al_2021	PASS
T18	Tibetan_Lhasa	Tibet	China	modern	0	Sino-Tibetan	30	91.1	Tibetan	N.A.	Wang_C_et_al_2021	PASS
T67	Tibetan_Nagqu	Tibet	China	modern	0	Sino-Tibetan	31.5	92.1	Tibetan	N.A.	Wang_C_et_al_2021	PASS
T91	Tibetan_Nagqu	Tibet	China	modern	0	Sino-Tibetan	31.5	92.1	Tibetan	N.A.	Wang_C_et_al_2021	PASS
T121	Tibetan_Nagqu	Tibet	China	modern	0	Sino-Tibetan	31.5	92.1	Tibetan	N.A.	Wang_C_et_al_2021	PASS
T187	Tibetan_Nagqu	Tibet	China	modern	0	Sino-Tibetan	31.5	92.1	Tibetan	N.A.	Wang_C_et_al_2021	PASS
T238	Tibetan_Nagqu	Tibet	China	modern	0	Sino-Tibetan	31.5	92.1	Tibetan	N.A.	Wang_C_et_al_2021	PASS
T278	Tibetan_Nagqu	Tibet	China	modern	0	Sino-Tibetan	31.5	92.1	Tibetan	N.A.	Wang_C_et_al_2021	PASS
T312	Tibetan_Nagqu	Tibet	China	modern	0	Sino-Tibetan	31.5	92.1	Tibetan	N.A.	Wang_C_et_al_2021	PASS
T320	Tibetan_Nagqu	Tibet	China	modern	0	Sino-Tibetan	31.5	92.1	Tibetan	N.A.	Wang_C_et_al_2021	PASS
T188	Tibetan_Shannan	Tibet	China	modern	0	Sino-Tibetan	29.2	91.8	Tibetan	N.A.	Wang_C_et_al_2021	PASS
T190	Tibetan_Shannan	Tibet	China	modern	0	Sino-Tibetan	29.2	91.8	Tibetan	N.A.	Wang_C_et_al_2021	PASS
T242	Tibetan_Shannan	Tibet	China	modern	0	Sino-Tibetan	29.2	91.8	Tibetan	N.A.	Wang_C_et_al_2021	PASS
T248	Tibetan_Shannan	Tibet	China	modern	0	Sino-Tibetan	29.2	91.8	Tibetan	N.A.	Wang_C_et_al_2021	PASS
T126	Tibetan_Shannan	Tibet	China	modern	0	Sino-Tibetan	29.2	91.8	Tibetan	N.A.	Wang_C_et_al_2021	PASS
T127	Tibetan_Shannan	Tibet	China	modern	0	Sino-Tibetan	29.2	91.8	Tibetan	N.A.	Wang_C_et_al_2021	PASS
T199	Tibetan_Shannan	Tibet	China	modern	0	Sino-Tibetan	29.2	91.8	Tibetan	N.A.	Wang_C_et_al_2021	PASS
T200	Tibetan_Shannan	Tibet	China	modern	0	Sino-Tibetan	29.2	91.8	Tibetan	N.A.	Wang_C_et_al_2021	PASS
T289	Tibetan_Shannan	Tibet	China	modern	0	Sino-Tibetan	29.2	91.8	Tibetan	N.A.	Wang_C_et_al_2021	PASS
T22	Tibetan_Shigatse	Tibet	China	modern	0	Sino-Tibetan	29.3	88.9	Tibetan	N.A.	Wang_C_et_al_2021	PASS
T41	Tibetan_Shigatse	Tibet	China	modern	0	Sino-Tibetan	29.3	88.9	Tibetan	N.A.	Wang_C_et_al_2021	PASS
T77	Tibetan_Shigatse	Tibet	China	modern	0	Sino-Tibetan	29.3	88.9	Tibetan	N.A.	Wang_C_et_al_2021	PASS
T78	Tibetan_Shigatse	Tibet	China	modern	0	Sino-Tibetan	29.3	88.9	Tibetan	N.A.	Wang_C_et_al_2021	PASS
T80	Tibetan_Shigatse	Tibet	China	modern	0	Sino-Tibetan	29.3	88.9	Tibetan	N.A.	Wang_C_et_al_2021	PASS
T189	Tibetan_Shigatse	Tibet	China	modern	0	Sino-Tibetan	29.3	88.9	Tibetan	N.A.	Wang_C_et_al_2021	PASS
T192	Tibetan_Shigatse	Tibet	China	modern	0	Sino-Tibetan	29.3	88.9	Tibetan	N.A.	Wang_C_et_al_2021	PASS
T291	Tibetan_Shigatse	Tibet	China	modern	0	Sino-Tibetan	29.3	88.9	Tibetan	N.A.	Wang_C_et_al_2021	PASS
T297	Tibetan_Shigatse	Tibet	China	modern	0	Sino-Tibetan	29.3	88.9	Tibetan	N.A.	Wang_C_et_al_2021	PASS
T215	Tibetan_Shigatse	Tibet	China	modern	0	Sino-Tibetan	29.3	88.9	Tibetan	N.A.	Wang_C_et_al_2021	PASS
XL120	Tibetan_Xinlong	Tibet	China	modern	0	Sino-Tibetan	31	100.3	Tibetan	N.A.	Wang_C_et_al_2021	PASS
XL61	Tibetan_Xinlong	Tibet	China	modern	0	Sino-Tibetan	31	100.3	Tibetan	N.A.	Wang_C_et_al_2021	PASS
XL20	Tibetan_Xinlong	Tibet	China	modern	0	Sino-Tibetan	31	100.3	Tibetan	N.A.	Wang_C_et_al_2021	PASS
XL92	Tibetan_Xinlong	Tibet	China	modern	0	Sino-Tibetan	31	100.3	Tibetan	N.A.	Wang_C_et_al_2021	PASS
XL60	Tibetan_Xinlong	Tibet	China	modern	0	Sino-Tibetan	31	100.3	Tibetan	N.A.	Wang_C_et_al_2021	PASS
XL47	Tibetan_Xinlong	Tibet	China	modern	0	Sino-Tibetan	31	100.3	Tibetan	N.A.	Wang_C_et_al_2021	PASS
XL76	Tibetan_Xinlong	Tibet	China	modern	0	Sino-Tibetan	31	100.3	Tibetan	N.A.	Wang_C_et_al_2021	PASS
XL22	Tibetan_Xinlong	Tibet	China	modern	0	Sino-Tibetan	31	100.3	Tibetan	N.A.	Wang_C_et_al_2021	PASS
XL37	Tibetan_Xinlong	Tibet	China	modern	0	Sino-Tibetan	31	100.3	Tibetan	N.A.	Wang_C_et_al_2021	PASS
XL05	Tibetan_Xinlong	Tibet	China	modern	0	Sino-Tibetan	31	100.3	Tibetan	N.A.	Wang_C_et_al_2021	PASS
XHTB7	Tibetan_Xunhua	Tibet	China	modern	0	Sino-Tibetan	35.8	102.5	Tibetan	N.A.	Wang_C_et_al_2021	PASS
XHTB17	Tibetan_Xunhua	Tibet	China	modern	0	Sino-Tibetan	35.8	102.5	Tibetan	N.A.	Wang_C_et_al_2021	PASS
XHTB20	Tibetan_Xunhua	Tibet	China	modern	0	Sino-Tibetan	35.8	102.5	Tibetan	N.A.	Wang_C_et_al_2021	PASS

XHTB5	Tibetan_Xunhua	Tibet	China	modern	0	Sino-Tibetan	35.8	102.5	Tibetan	N.A.	Wang_C_et_al_2021	PASS
YJ86	Tibetan_Yajiang	Tibet	China	modern	0	Sino-Tibetan	30	101	Tibetan	N.A.	Wang_C_et_al_2021	PASS
YJ14	Tibetan_Yajiang	Tibet	China	modern	0	Sino-Tibetan	30	101	Tibetan	N.A.	Wang_C_et_al_2021	PASS
YJ39	Tibetan_Yajiang	Tibet	China	modern	0	Sino-Tibetan	30	101	Tibetan	N.A.	Wang_C_et_al_2021	PASS
YJ01	Tibetan_Yajiang	Tibet	China	modern	0	Sino-Tibetan	30	101	Tibetan	N.A.	Wang_C_et_al_2021	PASS
YJ140	Tibetan_Yajiang	Tibet	China	modern	0	Sino-Tibetan	30	101	Tibetan	N.A.	Wang_C_et_al_2021	PASS
YJ37	Tibetan_Yajiang	Tibet	China	modern	0	Sino-Tibetan	30	101	Tibetan	N.A.	Wang_C_et_al_2021	PASS
YJ12	Tibetan_Yajiang	Tibet	China	modern	0	Sino-Tibetan	30	101	Tibetan	N.A.	Wang_C_et_al_2021	PASS
YJ111	Tibetan_Yajiang	Tibet	China	modern	0	Sino-Tibetan	30	101	Tibetan	N.A.	Wang_C_et_al_2021	PASS
YJ25	Tibetan_Yajiang	Tibet	China	modern	0	Sino-Tibetan	30	101	Tibetan	N.A.	Wang_C_et_al_2021	PASS
YJ104	Tibetan_Yajiang	Tibet	China	modern	0	Sino-Tibetan	30	101	Tibetan	N.A.	Wang_C_et_al_2021	PASS
Tibetan870	Tibetan_Yunnan	Tibet	China	modern	0	Sino-Tibetan	27.8	99.7	Tibetan	N.A.	Wang_C_et_al_2021	PASS
Tibetan871	Tibetan_Yunnan	Tibet	China	modern	0	Sino-Tibetan	27.8	99.7	Tibetan	N.A.	Wang_C_et_al_2021	PASS
Tibetan872	Tibetan_Yunnan	Tibet	China	modern	0	Sino-Tibetan	27.8	99.7	Tibetan	N.A.	Wang_C_et_al_2021	PASS
Tibetan937	Tibetan_Yunnan	Tibet	China	modern	0	Sino-Tibetan	27.8	99.7	Tibetan	N.A.	Wang_C_et_al_2021	PASS
Dong02	Dong_Guizhou	sEA	China	modern	0	Tai-Kadai	26.7	106.6	Dong	N.A.	Wang_C_et_al_2021	PASS
Dong03	Dong_Guizhou	sEA	China	modern	0	Tai-Kadai	26.7	106.6	Dong	N.A.	Wang_C_et_al_2021	PASS
Dong05	Dong_Guizhou	sEA	China	modern	0	Tai-Kadai	26.7	106.6	Dong	N.A.	Wang_C_et_al_2021	PASS
Dong08	Dong_Guizhou	sEA	China	modern	0	Tai-Kadai	26.7	106.6	Dong	N.A.	Wang_C_et_al_2021	PASS
Dong09	Dong_Guizhou	sEA	China	modern	0	Tai-Kadai	26.7	106.6	Dong	N.A.	Wang_C_et_al_2021	PASS
Dong10	Dong_Guizhou	sEA	China	modern	0	Tai-Kadai	26.7	106.6	Dong	N.A.	Wang_C_et_al_2021	PASS
Dong11	Dong_Guizhou	sEA	China	modern	0	Tai-Kadai	26.7	106.6	Dong	N.A.	Wang_C_et_al_2021	PASS
Dong14	Dong_Guizhou	sEA	China	modern	0	Tai-Kadai	26.7	106.6	Dong	N.A.	Wang_C_et_al_2021	PASS
Dong13	Dong_Guizhou	sEA	China	modern	0	Tai-Kadai	26.7	106.6	Dong	N.A.	Wang_C_et_al_2021	PASS
Dong01	Dong_Guizhou	sEA	China	modern	0	Tai-Kadai	26.7	106.6	Dong	N.A.	Wang_C_et_al_2021	PASS
Dong007	Dong_Guizhou	sEA	China	modern	0	Tai-Kadai	26.7	106.6	Dong	N.A.	Wang_C_et_al_2021	PASS
HN1-243	Dong_Hunan	sEA	China	modern	0	Tai-Kadai	27.4	109.2	Dong	N.A.	Wang_C_et_al_2021	PASS
HN1-273	Dong_Hunan	sEA	China	modern	0	Tai-Kadai	27.4	109.2	Dong	N.A.	Wang_C_et_al_2021	PASS
HN1-232	Dong_Hunan	sEA	China	modern	0	Tai-Kadai	27.4	109.2	Dong	N.A.	Wang_C_et_al_2021	PASS
HN1-218	Dong_Hunan	sEA	China	modern	0	Tai-Kadai	27.4	109.2	Dong	N.A.	Wang_C_et_al_2021	PASS
HN1-206	Dong_Hunan	sEA	China	modern	0	Tai-Kadai	27.4	109.2	Dong	N.A.	Wang_C_et_al_2021	PASS
Dong04	Dong_Hunan	sEA	China	modern	0	Tai-Kadai	27.4	109.2	Dong	N.A.	Wang_C_et_al_2021	PASS
HN1-260	Dong_Hunan	sEA	China	modern	0	Tai-Kadai	27.4	109.2	Dong	N.A.	Wang_C_et_al_2021	PASS
Dong12	Dong_Hunan	sEA	China	modern	0	Tai-Kadai	27.4	109.2	Dong	N.A.	Wang_C_et_al_2021	PASS
HN1-106	Dong_Hunan	sEA	China	modern	0	Tai-Kadai	27.4	109.2	Dong	N.A.	Wang_C_et_al_2021	PASS
GL01	Gelao	sEA	China	modern	0	Tai-Kadai	24.8	105.3	Gelao	N.A.	Wang_C_et_al_2021	PASS
GL02	Gelao	sEA	China	modern	0	Tai-Kadai	24.8	105.3	Gelao	N.A.	Wang_C_et_al_2021	PASS
GL09	Gelao	sEA	China	modern	0	Tai-Kadai	24.8	105.3	Gelao	N.A.	Wang_C_et_al_2021	PASS
GL07	Gelao	sEA	China	modern	0	Tai-Kadai	24.8	105.3	Gelao	N.A.	Wang_C_et_al_2021	PASS
GL10	Gelao	sEA	China	modern	0	Tai-Kadai	24.8	105.3	Gelao	N.A.	Wang_C_et_al_2021	PASS
GL23	Gelao	sEA	China	modern	0	Tai-Kadai	24.8	105.3	Gelao	N.A.	Wang_C_et_al_2021	PASS
GL13	Gelao	sEA	China	modern	0	Tai-Kadai	24.8	105.3	Gelao	N.A.	Wang_C_et_al_2021	PASS
GL16	Gelao	sEA	China	modern	0	Tai-Kadai	24.8	105.3	Gelao	N.A.	Wang_C_et_al_2021	PASS
GL20	Gelao	sEA	China	modern	0	Tai-Kadai	24.8	105.3	Gelao	N.A.	Wang_C_et_al_2021	PASS

GL24	Gelao	sEA	China	modern	0	Tai-Kadai	24.8	105.3	Gelao	N.A.	Wang_C_et_al_ 2021	PASS
CS411	Li	sEA	China	modern	0	Tai-Kadai	18.5	110	Li	N.A.	Wang_C_et_al_ 2021	PASS
LS001	Li	sEA	China	modern	0	Tai-Kadai	18.5	110	Li	N.A.	Wang_C_et_al_ 2021	PASS
SY071	Li	sEA	China	modern	0	Tai-Kadai	18.5	110	Li	N.A.	Wang_C_et_al_ 2021	PASS
WZS058	Li	sEA	China	modern	0	Tai-Kadai	18.5	110	Li	N.A.	Wang_C_et_al_ 2021	PASS
W14	Maonan	sEA	China	modern	0	Tai-Kadai	24.8	108.3	Maonan	N.A.	Wang_C_et_al_ 2021	PASS
W10	Maonan	sEA	China	modern	0	Tai-Kadai	24.8	108.3	Maonan	N.A.	Wang_C_et_al_ 2021	PASS
J6	Maonan	sEA	China	modern	0	Tai-Kadai	24.8	108.3	Maonan	N.A.	Wang_C_et_al_ 2021	PASS
J58	Maonan	sEA	China	modern	0	Tai-Kadai	24.8	108.3	Maonan	N.A.	Wang_C_et_al_ 2021	PASS
J17	Maonan	sEA	China	modern	0	Tai-Kadai	24.8	108.3	Maonan	N.A.	Wang_C_et_al_ 2021	PASS
J9	Maonan	sEA	China	modern	0	Tai-Kadai	24.8	108.3	Maonan	N.A.	Wang_C_et_al_ 2021	PASS
J29	Maonan	sEA	China	modern	0	Tai-Kadai	24.8	108.3	Maonan	N.A.	Wang_C_et_al_ 2021	PASS
W29	Maonan	sEA	China	modern	0	Tai-Kadai	24.8	108.3	Maonan	N.A.	Wang_C_et_al_ 2021	PASS
W3	Maonan	sEA	China	modern	0	Tai-Kadai	24.8	108.3	Maonan	N.A.	Wang_C_et_al_ 2021	PASS
W11	Maonan	sEA	China	modern	0	Tai-Kadai	24.8	108.3	Maonan	N.A.	Wang_C_et_al_ 2021	PASS
J32	Maonan	sEA	China	modern	0	Tai-Kadai	24.8	108.3	Maonan	N.A.	Wang_C_et_al_ 2021	PASS
J33	Maonan	sEA	China	modern	0	Tai-Kadai	24.8	108.3	Maonan	N.A.	Wang_C_et_al_ 2021	PASS
J36	Maonan	sEA	China	modern	0	Tai-Kadai	24.8	108.3	Maonan	N.A.	Wang_C_et_al_ 2021	PASS
W13	Maonan	sEA	China	modern	0	Tai-Kadai	24.8	108.3	Maonan	N.A.	Wang_C_et_al_ 2021	PASS
W26	Maonan	sEA	China	modern	0	Tai-Kadai	24.8	108.3	Maonan	N.A.	Wang_C_et_al_ 2021	PASS
W15	Maonan	sEA	China	modern	0	Tai-Kadai	24.8	108.3	Maonan	N.A.	Wang_C_et_al_ 2021	PASS
W41	Maonan	sEA	China	modern	0	Tai-Kadai	24.8	108.3	Maonan	N.A.	Wang_C_et_al_ 2021	PASS
B87	Mulam	sEA	China	modern	0	Tai-Kadai	24.8	108.9	Mulam	N.A.	Wang_C_et_al_ 2021	PASS
B26	Mulam	sEA	China	modern	0	Tai-Kadai	24.8	108.9	Mulam	N.A.	Wang_C_et_al_ 2021	PASS
A114	Mulam	sEA	China	modern	0	Tai-Kadai	24.8	108.9	Mulam	N.A.	Wang_C_et_al_ 2021	PASS
B3	Mulam	sEA	China	modern	0	Tai-Kadai	24.8	108.9	Mulam	N.A.	Wang_C_et_al_ 2021	PASS
B81	Mulam	sEA	China	modern	0	Tai-Kadai	24.8	108.9	Mulam	N.A.	Wang_C_et_al_ 2021	PASS
B48	Mulam	sEA	China	modern	0	Tai-Kadai	24.8	108.9	Mulam	N.A.	Wang_C_et_al_ 2021	PASS
B49	Mulam	sEA	China	modern	0	Tai-Kadai	24.8	108.9	Mulam	N.A.	Wang_C_et_al_ 2021	PASS
B8	Mulam	sEA	China	modern	0	Tai-Kadai	24.8	108.9	Mulam	N.A.	Wang_C_et_al_ 2021	PASS
B14	Mulam	sEA	China	modern	0	Tai-Kadai	24.8	108.9	Mulam	N.A.	Wang_C_et_al_ 2021	PASS
B43	Mulam	sEA	China	modern	0	Tai-Kadai	24.8	108.9	Mulam	N.A.	Wang_C_et_al_ 2021	PASS
B18	Mulam	sEA	China	modern	0	Tai-Kadai	24.8	108.9	Mulam	N.A.	Wang_C_et_al_ 2021	PASS
B32	Mulam	sEA	China	modern	0	Tai-Kadai	24.8	108.9	Mulam	N.A.	Wang_C_et_al_ 2021	PASS
A019	Mulam	sEA	China	modern	0	Tai-Kadai	24.8	108.9	Mulam	N.A.	Wang_C_et_al_ 2021	PASS
A015	Mulam	sEA	China	modern	0	Tai-Kadai	24.8	108.9	Mulam	N.A.	Wang_C_et_al_ 2021	PASS
A005	Mulam	sEA	China	modern	0	Tai-Kadai	24.8	108.9	Mulam	N.A.	Wang_C_et_al_ 2021	PASS
A016	Mulam	sEA	China	modern	0	Tai-Kadai	24.8	108.9	Mulam	N.A.	Wang_C_et_al_ 2021	PASS
A123	Mulam	sEA	China	modern	0	Tai-Kadai	24.8	108.9	Mulam	N.A.	Wang_C_et_al_ 2021	PASS
Zhuang01	Zhuang	sEA	China	modern	0	Tai-Kadai	22.8	108.4	Zhuang	N.A.	Wang_C_et_al_ 2021	PASS
Zhuang012	Zhuang	sEA	China	modern	0	Tai-Kadai	22.8	108.4	Zhuang	N.A.	Wang_C_et_al_ 2021	PASS
Zhuang019	Zhuang	sEA	China	modern	0	Tai-Kadai	22.8	108.4	Zhuang	N.A.	Wang_C_et_al_ 2021	PASS
Zhuang02	Zhuang	sEA	China	modern	0	Tai-Kadai	22.8	108.4	Zhuang	N.A.	Wang_C_et_al_ 2021	PASS
Zhuang04	Zhuang	sEA	China	modern	0	Tai-Kadai	22.8	108.4	Zhuang	N.A.	Wang_C_et_al_ 2021	PASS

Zhuang013	Zhuang	sEA	China	modern	0	Tai-Kadai	22.8	108.4	Zhuang	N.A.	Wang_C_et_al_ 2021	PASS
Zhuang09	Zhuang	sEA	China	modern	0	Tai-Kadai	22.8	108.4	Zhuang	N.A.	Wang_C_et_al_ 2021	PASS
Zhuang08	Zhuang	sEA	China	modern	0	Tai-Kadai	22.8	108.4	Zhuang	N.A.	Wang_C_et_al_ 2021	PASS
Zhuang03	Zhuang	sEA	China	modern	0	Tai-Kadai	22.8	108.4	Zhuang	N.A.	Wang_C_et_al_ 2021	PASS
Zhuang015	Zhuang	sEA	China	modern	0	Tai-Kadai	22.8	108.4	Zhuang	N.A.	Wang_C_et_al_ 2021	PASS
Zhuang018	Zhuang	sEA	China	modern	0	Tai-Kadai	22.8	108.4	Zhuang	N.A.	Wang_C_et_al_ 2021	PASS
Zhuang11	Zhuang	sEA	China	modern	0	Tai-Kadai	22.8	108.4	Zhuang	N.A.	Wang_C_et_al_ 2021	PASS
Zhuang017	Zhuang	sEA	China	modern	0	Tai-Kadai	22.8	108.4	Zhuang	N.A.	Wang_C_et_al_ 2021	PASS
Zhuang020	Zhuang	sEA	China	modern	0	Tai-Kadai	22.8	108.4	Zhuang	N.A.	Wang_C_et_al_ 2021	PASS
Zhuang16	Zhuang	sEA	China	modern	0	Tai-Kadai	22.8	108.4	Zhuang	N.A.	Wang_C_et_al_ 2021	PASS
Zhuang10	Zhuang	sEA	China	modern	0	Tai-Kadai	22.8	108.4	Zhuang	N.A.	Wang_C_et_al_ 2021	PASS
Zhuang06	Zhuang	sEA	China	modern	0	Tai-Kadai	22.8	108.4	Zhuang	N.A.	Wang_C_et_al_ 2021	PASS
Zhuang05	Zhuang	sEA	China	modern	0	Tai-Kadai	22.8	108.4	Zhuang	N.A.	Wang_C_et_al_ 2021	PASS
Zhuang07	Zhuang	sEA	China	modern	0	Tai-Kadai	22.8	108.4	Zhuang	N.A.	Wang_C_et_al_ 2021	PASS
Z01	Zhuang	sEA	China	modern	0	Tai-Kadai	22.8	108.4	Zhuang	N.A.	Wang_C_et_al_ 2021	PASS
Z02	Zhuang	sEA	China	modern	0	Tai-Kadai	22.8	108.4	Zhuang	N.A.	Wang_C_et_al_ 2021	PASS
Z05	Zhuang	sEA	China	modern	0	Tai-Kadai	22.8	108.4	Zhuang	N.A.	Wang_C_et_al_ 2021	PASS
HK03	Kazakh_China	nEA	China	modern	0	Turkic	38.5	94.3	N.A.	N.A.	Wang_C_et_al_ 2021	PASS
HK4	Kazakh_China	nEA	China	modern	0	Turkic	38.5	94.3	N.A.	N.A.	Wang_C_et_al_ 2021	PASS
HK14	Kazakh_China	nEA	China	modern	0	Turkic	38.5	94.3	N.A.	N.A.	Wang_C_et_al_ 2021	PASS
HK15	Kazakh_China	nEA	China	modern	0	Turkic	38.5	94.3	N.A.	N.A.	Wang_C_et_al_ 2021	PASS
HKC90	Kazakh_China	nEA	China	modern	0	Turkic	38.5	94.3	N.A.	N.A.	Wang_C_et_al_ 2021	PASS
HKC29	Kazakh_China	nEA	China	modern	0	Turkic	38.5	94.3	N.A.	N.A.	Wang_C_et_al_ 2021	PASS
HKC109	Kazakh_China	nEA	China	modern	0	Turkic	38.5	94.3	N.A.	N.A.	Wang_C_et_al_ 2021	PASS
HKC140	Kazakh_China	nEA	China	modern	0	Turkic	38.5	94.3	N.A.	N.A.	Wang_C_et_al_ 2021	PASS
KZ78	Kyrgyz_China	nEA	China	modern	0	Turkic	43.8	87.7	N.A.	N.A.	Wang_C_et_al_ 2021	PASS
KZ35	Kyrgyz_China	nEA	China	modern	0	Turkic	43.8	87.7	N.A.	N.A.	Wang_C_et_al_ 2021	PASS
KZ15	Kyrgyz_China	nEA	China	modern	0	Turkic	43.8	87.7	N.A.	N.A.	Wang_C_et_al_ 2021	PASS
KZ77	Kyrgyz_China	nEA	China	modern	0	Turkic	43.8	87.7	N.A.	N.A.	Wang_C_et_al_ 2021	PASS
KZ52	Kyrgyz_China	nEA	China	modern	0	Turkic	43.8	87.7	N.A.	N.A.	Wang_C_et_al_ 2021	PASS
KZ12	Kyrgyz_China	nEA	China	modern	0	Turkic	43.8	87.7	N.A.	N.A.	Wang_C_et_al_ 2021	PASS
KZ83	Kyrgyz_China	nEA	China	modern	0	Turkic	43.8	87.7	N.A.	N.A.	Wang_C_et_al_ 2021	PASS
KZ46	Kyrgyz_China	nEA	China	modern	0	Turkic	43.8	87.7	N.A.	N.A.	Wang_C_et_al_ 2021	PASS
KZ81	Kyrgyz_China	nEA	China	modern	0	Turkic	43.8	87.7	N.A.	N.A.	Wang_C_et_al_ 2021	PASS
KZ87	Kyrgyz_China	nEA	China	modern	0	Turkic	43.8	87.7	N.A.	N.A.	Wang_C_et_al_ 2021	PASS
KZ65	Kyrgyz_China	nEA	China	modern	0	Turkic	43.8	87.7	N.A.	N.A.	Wang_C_et_al_ 2021	PASS
KZ22	Kyrgyz_China	nEA	China	modern	0	Turkic	43.8	87.7	N.A.	N.A.	Wang_C_et_al_ 2021	PASS
KZ73	Kyrgyz_China	nEA	China	modern	0	Turkic	43.8	87.7	N.A.	N.A.	Wang_C_et_al_ 2021	PASS
SL1	Salar	nEA	China	modern	0	Turkic	35.8	102.5	N.A.	N.A.	Wang_C_et_al_ 2021	PASS
SL2	Salar	nEA	China	modern	0	Turkic	35.8	102.5	N.A.	N.A.	Wang_C_et_al_ 2021	PASS
SL3	Salar	nEA	China	modern	0	Turkic	35.8	102.5	N.A.	N.A.	Wang_C_et_al_ 2021	PASS
SL6	Salar	nEA	China	modern	0	Turkic	35.8	102.5	N.A.	N.A.	Wang_C_et_al_ 2021	PASS
SL7	Salar	nEA	China	modern	0	Turkic	35.8	102.5	N.A.	N.A.	Wang_C_et_al_ 2021	PASS
SL9	Salar	nEA	China	modern	0	Turkic	35.8	102.5	N.A.	N.A.	Wang_C_et_al_ 2021	PASS

SL10	Salar	nEA	China	modern	0	Turkic	35.8	102.5	N.A.	N.A.	Wang_C_et_al_2021	PASS
SL15	Salar	nEA	China	modern	0	Turkic	35.8	102.5	N.A.	N.A.	Wang_C_et_al_2021	PASS
Ba37	Bahun	SouthAsia	Nepal	modern	0	Indo-European	27.4	85.3	N.A.	N.A.	Wang_C_et_al_2021	PASS
Ba48	Bahun	SouthAsia	Nepal	modern	0	Indo-European	27.4	85.3	N.A.	N.A.	Wang_C_et_al_2021	PASS
Ba15	Bahun	SouthAsia	Nepal	modern	0	Indo-European	27.4	85.3	N.A.	N.A.	Wang_C_et_al_2021	PASS
Ba25	Bahun	SouthAsia	Nepal	modern	0	Indo-European	27.4	85.3	N.A.	N.A.	Wang_C_et_al_2021	PASS
Ba02	Bahun	SouthAsia	Nepal	modern	0	Indo-European	27.4	85.3	N.A.	N.A.	Wang_C_et_al_2021	PASS
Rai16	Rai	SouthAsia	Nepal	modern	0	Indo-European	27.4	85.3	N.A.	N.A.	Wang_C_et_al_2021	PASS
Rai12	Rai	SouthAsia	Nepal	modern	0	Indo-European	27.4	85.3	N.A.	N.A.	Wang_C_et_al_2021	PASS
Rai09	Rai	SouthAsia	Nepal	modern	0	Indo-European	27.4	85.3	N.A.	N.A.	Wang_C_et_al_2021	PASS
Rai14	Rai	SouthAsia	Nepal	modern	0	Indo-European	27.4	85.3	N.A.	N.A.	Wang_C_et_al_2021	PASS
Rai04	Rai	SouthAsia	Nepal	modern	0	Indo-European	27.4	85.3	N.A.	N.A.	Wang_C_et_al_2021	PASS
Te18	Tharu	SouthAsia	Nepal	modern	0	Indo-European	27.4	86.2	N.A.	N.A.	Wang_C_et_al_2021	PASS
Te16	Tharu	SouthAsia	Nepal	modern	0	Indo-European	27.4	86.2	N.A.	N.A.	Wang_C_et_al_2021	PASS
Te09	Tharu	SouthAsia	Nepal	modern	0	Indo-European	27.4	86.2	N.A.	N.A.	Wang_C_et_al_2021	PASS
Te15	Tharu	SouthAsia	Nepal	modern	0	Indo-European	27.4	86.2	N.A.	N.A.	Wang_C_et_al_2021	PASS
Te06	Tharu	SouthAsia	Nepal	modern	0	Indo-European	27.4	86.2	N.A.	N.A.	Wang_C_et_al_2021	PASS
Gu18	Gurung	SouthAsia	Nepal	modern	0	Sino-Tibetan	27.4	86.2	Gurung	N.A.	Wang_C_et_al_2021	PASS
Gu22	Gurung	SouthAsia	Nepal	modern	0	Sino-Tibetan	27.4	86.2	Gurung	N.A.	Wang_C_et_al_2021	PASS
Gu24	Gurung	SouthAsia	Nepal	modern	0	Sino-Tibetan	27.4	86.2	Gurung	N.A.	Wang_C_et_al_2021	PASS
Gu26	Gurung	SouthAsia	Nepal	modern	0	Sino-Tibetan	27.4	86.2	Gurung	N.A.	Wang_C_et_al_2021	PASS
Gu27	Gurung	SouthAsia	Nepal	modern	0	Sino-Tibetan	27.4	86.2	Gurung	N.A.	Wang_C_et_al_2021	PASS
Ma41	Magar	SouthAsia	Nepal	modern	0	Sino-Tibetan	27.4	86.2	Magar	N.A.	Wang_C_et_al_2021	PASS
Ma11	Magar	SouthAsia	Nepal	modern	0	Sino-Tibetan	27.4	86.2	Magar	N.A.	Wang_C_et_al_2021	PASS
Ma30	Magar	SouthAsia	Nepal	modern	0	Sino-Tibetan	27.4	86.2	Magar	N.A.	Wang_C_et_al_2021	PASS
Ma23	Magar	SouthAsia	Nepal	modern	0	Sino-Tibetan	27.4	86.2	Magar	N.A.	Wang_C_et_al_2021	PASS
Ma07	Magar	SouthAsia	Nepal	modern	0	Sino-Tibetan	27.4	86.2	Magar	N.A.	Wang_C_et_al_2021	PASS
Ma28	Magar	SouthAsia	Nepal	modern	0	Sino-Tibetan	27.4	86.2	Magar	N.A.	Wang_C_et_al_2021	PASS
Ne41	Newar	SouthAsia	Nepal	modern	0	Sino-Tibetan	27.4	85.3	Newar	N.A.	Wang_C_et_al_2021	PASS
Ne72	Newar	SouthAsia	Nepal	modern	0	Sino-Tibetan	27.4	85.3	Newar	N.A.	Wang_C_et_al_2021	PASS
Ne30	Newar	SouthAsia	Nepal	modern	0	Sino-Tibetan	27.4	85.3	Newar	N.A.	Wang_C_et_al_2021	PASS
Ne08	Newar	SouthAsia	Nepal	modern	0	Sino-Tibetan	27.4	85.3	Newar	N.A.	Wang_C_et_al_2021	PASS
Ne66	Newar	SouthAsia	Nepal	modern	0	Sino-Tibetan	27.4	85.3	Newar	N.A.	Wang_C_et_al_2021	PASS
Ne61	Newar	SouthAsia	Nepal	modern	0	Sino-Tibetan	27.4	85.3	Newar	N.A.	Wang_C_et_al_2021	PASS
Ne35	Newar	SouthAsia	Nepal	modern	0	Sino-Tibetan	27.4	85.3	Newar	N.A.	Wang_C_et_al_2021	PASS
Ne01	Newar	SouthAsia	Nepal	modern	0	Sino-Tibetan	27.4	85.3	Newar	N.A.	Wang_C_et_al_2021	PASS
Sherpa08	Sherpa	SouthAsia	Nepal	modern	0	Sino-Tibetan	27.4	85.3	Sherpa	N.A.	Wang_C_et_al_2021	PASS
Sherpa04	Sherpa	SouthAsia	Nepal	modern	0	Sino-Tibetan	27.4	85.3	Sherpa	N.A.	Wang_C_et_al_2021	PASS
Sherpa13	Sherpa	SouthAsia	Nepal	modern	0	Sino-Tibetan	27.4	85.3	Sherpa	N.A.	Wang_C_et_al_2021	PASS
Sherpa09	Sherpa	SouthAsia	Nepal	modern	0	Sino-Tibetan	27.4	85.3	Sherpa	N.A.	Wang_C_et_al_2021	PASS
Ta39	Tamang	SouthAsia	Nepal	modern	0	Sino-Tibetan	27.4	86.2	Tamang	N.A.	Wang_C_et_al_2021	PASS
Ta43	Tamang	SouthAsia	Nepal	modern	0	Sino-Tibetan	27.4	86.2	Tamang	N.A.	Wang_C_et_al_2021	PASS
Ta24	Tamang	SouthAsia	Nepal	modern	0	Sino-Tibetan	27.4	86.2	Tamang	N.A.	Wang_C_et_al_2021	PASS
Ta64	Tamang	SouthAsia	Nepal	modern	0	Sino-Tibetan	27.4	86.2	Tamang	N.A.	Wang_C_et_al_2021	PASS

Ta80	Tamang	SouthAsia	Nepal	modern	0	Sino-Tibetan	27.4	86.2	Tamang	N.A.	Wang_C_et_al_2021	PASS
Ta52	Tamang	SouthAsia	Nepal	modern	0	Sino-Tibetan	27.4	86.2	Tamang	N.A.	Wang_C_et_al_2021	PASS
Ta05	Tamang	SouthAsia	Nepal	modern	0	Sino-Tibetan	27.4	86.2	Tamang	N.A.	Wang_C_et_al_2021	PASS
Ta03	Tamang	SouthAsia	Nepal	modern	0	Sino-Tibetan	27.4	86.2	Tamang	N.A.	Wang_C_et_al_2021	PASS
TY	Tianyuan_40000BP	nEA	China	ancient	40000	N.A.	39.4	115.5	N.A.	N.A.	Yang_et_al_2017	PASS
Yumin	Yumin_8400BP	nEA	Mongolia	ancient	8375	N.A.	42	114.2	N.A.	N.A.	Yang_et_al_2020	PASS
Bianbian	Bianbian_9500BP	nEA	China	ancient	9513	N.A.	36.1	118.5	N.A.	N.A.	Yang_et_al_2020	PASS
BS	Boshan_8200BP	nEA	China	ancient	8180	N.A.	36.5	117.9	N.A.	N.A.	Yang_et_al_2020	PASS
XJS1309_M7	Xiaojingshan_7800BP	nEA	China	ancient	7797	N.A.	36.5	117.9	N.A.	N.A.	Yang_et_al_2020	PASS
XJS1311_M16	Xiaojingshan_7800BP	nEA	China	ancient	7861	N.A.	36.5	117	N.A.	N.A.	Yang_et_al_2020	PASS
XJS1309_M4	Xiaojingshan_7800BP	nEA	China	ancient	7806	N.A.	36.5	117	N.A.	N.A.	Yang_et_al_2020	PASS
Xiaogao	Xiaogao_8700BP	nEA	China	ancient	8684	N.A.	37.9	117.6	N.A.	N.A.	Yang_et_al_2020	PASS
Qihe2_d	Qihe_8400BP	sEA	China	ancient	8394	N.A.	25.4	117.6	N.A.	N.A.	Yang_et_al_2020	PASS
LD1	Liangdao_7750BP	sEA	Taiwan	ancient	8190	N.A.	26.3	120.2	N.A.	N.A.	Yang_et_al_2020	PASS
LD2	Liangdao_7750BP	sEA	Taiwan	ancient	7575	N.A.	26.3	120.2	N.A.	N.A.	Yang_et_al_2020	PASS
SuogangB1_d	Suogang_4550BP	sEA	Taiwan	ancient	4550	N.A.	23.5	119.6	N.A.	N.A.	Yang_et_al_2020	PASS
SuogangB3_d	Suogang_4550BP	sEA	Taiwan	ancient	4550	N.A.	23.5	119.6	N.A.	N.A.	Yang_et_al_2020	PASS
L5705	Xitoucun_4500BP	sEA	China	ancient	4333	N.A.	26.2	119.1	N.A.	N.A.	Yang_et_al_2020	PASS
L5700	Xitoucun_4500BP	sEA	China	ancient	4474	N.A.	26.2	119.1	N.A.	N.A.	Yang_et_al_2020	PASS
L5692_d	Xitoucun_4500BP	sEA	China	ancient	4474	N.A.	26.2	119.1	N.A.	N.A.	Yang_et_al_2020	imiss
L5706_d	Xitoucun_4500BP	sEA	China	ancient	4467	N.A.	26.2	119.1	N.A.	N.A.	Yang_et_al_2020	PASS
L5704_d	Xitoucun_4500BP	sEA	China	ancient	4502	N.A.	26.2	119.1	N.A.	N.A.	Yang_et_al_2020	PASS
L5703_d	Xitoucun_4500BP	sEA	China	ancient	4572	N.A.	26.2	119.1	N.A.	N.A.	Yang_et_al_2020	imiss
L5701_d	Xitoucun_4500BP	sEA	China	ancient	4329	N.A.	26.2	119.1	N.A.	N.A.	Yang_et_al_2020	PASS
L7415	Tanshishan_4350BP	sEA	China	ancient	4333	N.A.	26.1	119.2	N.A.	N.A.	Yang_et_al_2020	PASS
L7417_d	Tanshishan_4350BP	sEA	China	ancient	4472	N.A.	26.1	119.2	N.A.	N.A.	Yang_et_al_2020	PASS
L5698_d	Tanshishan_4350BP	sEA	China	ancient	4318	N.A.	26.2	119.1	N.A.	N.A.	Yang_et_al_2020	imiss
L5696_d	Tanshishan_4350BP	sEA	China	ancient	NA	N.A.	26.2	119.1	N.A.	N.A.	Yang_et_al_2020	imiss
L5694	Chuanyun_300BP	sEA	China	ancient	308	N.A.	25.6	117.3	N.A.	N.A.	Yang_et_al_2020	PASS
I3354	Boisman_6900BP	nEA	Russia	ancient	7237	N.A.	42.79	131.28	N.A.	N.A.	Wang_C_et_al_2021	PASS
I1196	Boisman_6900BP	nEA	Russia	ancient	7058	N.A.	42.79	131.28	N.A.	N.A.	Wang_C_et_al_2021	PASS
I1198	Boisman_6900BP	nEA	Russia	ancient	6872	N.A.	42.79	131.28	N.A.	N.A.	Wang_C_et_al_2021	PASS
I1193	Boisman_6900BP	nEA	Russia	ancient	6832	N.A.	42.79	131.28	N.A.	N.A.	Wang_C_et_al_2021	PASS
I3355	Boisman_6900BP	nEA	Russia	ancient	6821	N.A.	42.79	131.28	N.A.	N.A.	Wang_C_et_al_2021	PASS
I1197	Boisman_6900BP	nEA	Russia	ancient	6804	N.A.	42.79	131.28	N.A.	N.A.	Wang_C_et_al_2021	PASS
I1190	Boisman_6900BP	nEA	Russia	ancient	6675	N.A.	42.79	131.28	N.A.	N.A.	Wang_C_et_al_2021	PASS
I1194	Boisman_6900BP	nEA	Russia	ancient	6652	N.A.	42.79	131.28	N.A.	N.A.	Wang_C_et_al_2021	PASS
I14819	Boisman_5600BP	nEA	Russia	ancient	5625	N.A.	42.79	131.28	N.A.	N.A.	Wang_C_et_al_2021	PASS
I3356	Boisman_5600BP	nEA	Russia	ancient	5605	N.A.	42.79	131.28	N.A.	N.A.	Wang_C_et_al_2021	PASS
I1206	Boisman_6900BP	nEA	Russia	ancient	6954	N.A.	42.79	131.28	N.A.	N.A.	Wang_C_et_al_2021	PASS
I13886	Jomon_3500BP	nEA	Japan	ancient	4003	N.A.	35.55	140.16	N.A.	N.A.	Wang_C_et_al_2021	PASS
I13884	Jomon_3500BP	nEA	Japan	ancient	4351	N.A.	35.55	140.16	N.A.	N.A.	Wang_C_et_al_2021	PASS
I6341	Jomon_3500BP	nEA	Japan	ancient	4200	N.A.	45.37	141.03	N.A.	N.A.	Wang_C_et_al_2021	PASS
I13882	Jomon_3500BP	nEA	Japan	ancient	3234	N.A.	35.55	140.16	N.A.	N.A.	Wang_C_et_al_2021	PASS

I13885	Jomon_3500BP	nEA	Japan	ancient	3216	N.A.	35.55	140.16	N.A.	N.A.	Wang_C_et_al_2021	PASS
I13883	Jomon_3500BP	nEA	Japan	ancient	2859	N.A.	35.55	140.16	N.A.	N.A.	Wang_C_et_al_2021	PASS
I6358	Mongolia_7300BP	nEA	Mongolia	ancient	7167	N.A.	48.29	115.1	N.A.	N.A.	Wang_C_et_al_2021	PASS
I7021	Mongolia_7300BP	nEA	Mongolia	ancient	7050	N.A.	48	113.93	N.A.	N.A.	Wang_C_et_al_2021	PASS
I11696	Mongolia_7300BP	nEA	Mongolia	ancient	7528	N.A.	49.39	102.7	N.A.	N.A.	Wang_C_et_al_2021	PASS
I11698	Mongolia_7300BP	nEA	Mongolia	ancient	7520	N.A.	49.39	102.7	N.A.	N.A.	Wang_C_et_al_2021	PASS
I11697	Mongolia_7300BP	nEA	Mongolia	ancient	7500	N.A.	49.39	102.7	N.A.	N.A.	Wang_C_et_al_2021	PASS
I13179	Mongolia_7300BP	nEA	Mongolia	ancient	7515	N.A.	49.39	102.7	N.A.	N.A.	Wang_C_et_al_2021	PASS
I13698	Mongolia_7300BP	nEA	Mongolia	ancient	7504	N.A.	49.53	103.28	N.A.	N.A.	Wang_C_et_al_2021	PASS
I14000	Mongolia_7300BP	nEA	Mongolia	ancient	7480	N.A.	49.53	103.28	N.A.	N.A.	Wang_C_et_al_2021	PASS
S97.EC	Wuzhuangguoliang_5000BP	nEA	China	ancient	5050	N.A.	37.82	109.05	N.A.	N.A.	Wang_C_et_al_2021	PASS
S123.EC	Wuzhuangguoliang_5000BP	nEA	China	ancient	5050	N.A.	37.82	109.05	N.A.	N.A.	Wang_C_et_al_2021	PASS
18R21262.EC	Wuzhuangguoliang_5000BP	nEA	China	ancient	5050	N.A.	37.82	109.05	N.A.	N.A.	Wang_C_et_al_2021	PASS
S91.EC	Wuzhuangguoliang_5000BP	nEA	China	ancient	5050	N.A.	37.82	109.05	N.A.	N.A.	Wang_C_et_al_2021	imiss
S118.EC	Wuzhuangguoliang_5000BP	nEA	China	ancient	5050	N.A.	37.82	109.05	N.A.	N.A.	Wang_C_et_al_2021	imiss
S120.EC	Wuzhuangguoliang_5000BP	nEA	China	ancient	5050	N.A.	37.82	109.05	N.A.	N.A.	Wang_C_et_al_2021	imiss
18R21265.EC	Wuzhuangguoliang_5000BP	nEA	China	ancient	5050	N.A.	37.82	109.05	N.A.	N.A.	Wang_C_et_al_2021	imiss
18R21266.EC	Wuzhuangguoliang_5000BP	nEA	China	ancient	4785	N.A.	37.82	109.05	N.A.	N.A.	Wang_C_et_al_2021	PASS
I6221	Afanasievo_5000BP	nEA	Mongolia	ancient	4996	N.A.	46.4	100.82	N.A.	N.A.	Wang_C_et_al_2021	PASS
I13957	Afanasievo_4600BP	nEA	Mongolia	ancient	4625	N.A.	49.34	88.71	N.A.	N.A.	Wang_C_et_al_2021	PASS
I6347	CenterWest_3100BP	nEA	Mongolia	ancient	3295	N.A.	49.31	95.45	N.A.	N.A.	Wang_C_et_al_2021	PASS
I6264	CenterWest_3100BP	nEA	Mongolia	ancient	3275	N.A.	45.92	100.83	N.A.	N.A.	Wang_C_et_al_2021	PASS
I6262	CenterWest_3100BP	nEA	Mongolia	ancient	3270	N.A.	45.92	100.83	N.A.	N.A.	Wang_C_et_al_2021	PASS
I13766	CenterWest_3100BP	nEA	Mongolia	ancient	3208	N.A.	46.43	100.82	N.A.	N.A.	Wang_C_et_al_2021	PASS
I13767	CenterWest_3100BP	nEA	Mongolia	ancient	3198	N.A.	48.11	102.55	N.A.	N.A.	Wang_C_et_al_2021	PASS
I12975	CenterWest_3100BP	nEA	Mongolia	ancient	3110	N.A.	51.5	100.67	N.A.	N.A.	Wang_C_et_al_2021	PASS
I7039	CenterWest_3100BP	nEA	Mongolia	ancient	3066	N.A.	47.42	92.23	N.A.	N.A.	Wang_C_et_al_2021	PASS
I13505	CenterWest_3100BP	nEA	Mongolia	ancient	3001	N.A.	46.43	100.82	N.A.	N.A.	Wang_C_et_al_2021	PASS
I6362	CenterWest_3100BP	nEA	Mongolia	ancient	2921	N.A.	46.06	92.03	N.A.	N.A.	Wang_C_et_al_2021	PASS
I6351	CenterWest_2900BP	nEA	Mongolia	ancient	2885	N.A.	49.7	93.8	N.A.	N.A.	Wang_C_et_al_2021	PASS
I12978	Chemurchek_4450BP	nEA	Mongolia	ancient	4472	N.A.	46.12	91.57	N.A.	N.A.	Wang_C_et_al_2021	PASS
I12957	Chemurchek_4450BP	nEA	Mongolia	ancient	4462	N.A.	46.12	91.57	N.A.	N.A.	Wang_C_et_al_2021	PASS
I3620	Hanben_1550BP	sEA	Taiwan	ancient	1857	N.A.	24.33	121.77	N.A.	N.A.	Wang_C_et_al_2021	PASS
I3615	Hanben_1550BP	sEA	Taiwan	ancient	1833	N.A.	24.33	121.77	N.A.	N.A.	Wang_C_et_al_2021	PASS
I3617	Hanben_1550BP	sEA	Taiwan	ancient	1644	N.A.	24.33	121.77	N.A.	N.A.	Wang_C_et_al_2021	PASS
I3616	Hanben_1550BP	sEA	Taiwan	ancient	1641	N.A.	24.33	121.77	N.A.	N.A.	Wang_C_et_al_2021	PASS
I3621	Hanben_1550BP	sEA	Taiwan	ancient	1595	N.A.	24.33	121.77	N.A.	N.A.	Wang_C_et_al_2021	PASS
I3618	Hanben_1550BP	sEA	Taiwan	ancient	1550	N.A.	24.33	121.77	N.A.	N.A.	Wang_C_et_al_2021	PASS
I3731	Hanben_1550BP	sEA	Taiwan	ancient	1550	N.A.	24.33	121.77	N.A.	N.A.	Wang_C_et_al_2021	PASS
I3727	Hanben_1550BP	sEA	Taiwan	ancient	1550	N.A.	24.33	121.77	N.A.	N.A.	Wang_C_et_al_2021	PASS
I3736	Hanben_1550BP	sEA	Taiwan	ancient	1550	N.A.	24.33	121.77	N.A.	N.A.	Wang_C_et_al_2021	PASS
I13692	Hanben_1550BP	sEA	Taiwan	ancient	1550	N.A.	24.33	121.77	N.A.	N.A.	Wang_C_et_al_2021	PASS
I8074	Hanben_1550BP	sEA	Taiwan	ancient	1550	N.A.	24.33	121.77	N.A.	N.A.	Wang_C_et_al_2021	PASS
I14929	Hanben_1550BP	sEA	Taiwan	ancient	1550	N.A.	24.33	121.77	N.A.	N.A.	Wang_C_et_al_2021	PASS

I14931	Hanben_1550BP	sEA	Taiwan	ancient	1550	N.A.	24.33	121.77	N.A.	N.A.	Wang_C_et_al_2021	PASS
I8080	Hanben_1550BP	sEA	Taiwan	ancient	1550	N.A.	24.33	121.77	N.A.	N.A.	Wang_C_et_al_2021	PASS
I14933	Hanben_1550BP	sEA	Taiwan	ancient	1550	N.A.	24.33	121.77	N.A.	N.A.	Wang_C_et_al_2021	PASS
I14934	Hanben_1550BP	sEA	Taiwan	ancient	1550	N.A.	24.33	121.77	N.A.	N.A.	Wang_C_et_al_2021	PASS
I8076	Hanben_1550BP	sEA	Taiwan	ancient	1550	N.A.	24.33	121.77	N.A.	N.A.	Wang_C_et_al_2021	PASS
I14925	Hanben_1550BP	sEA	Taiwan	ancient	1550	N.A.	24.33	121.77	N.A.	N.A.	Wang_C_et_al_2021	imiss
I3614	Hanben_1550BP	sEA	Taiwan	ancient	1550	N.A.	24.33	121.77	N.A.	N.A.	Wang_C_et_al_2021	PASS
I3728	Hanben_1550BP	sEA	Taiwan	ancient	1496	N.A.	24.33	121.77	N.A.	N.A.	Wang_C_et_al_2021	PASS
I3619	Hanben_1550BP	sEA	Taiwan	ancient	1473	N.A.	24.33	121.77	N.A.	N.A.	Wang_C_et_al_2021	PASS
I8075	Hanben_1550BP	sEA	Taiwan	ancient	1459	N.A.	24.33	121.77	N.A.	N.A.	Wang_C_et_al_2021	PASS
I13695	Hanben_1550BP	sEA	Taiwan	ancient	1459	N.A.	24.33	121.77	N.A.	N.A.	Wang_C_et_al_2021	PASS
I8081	Hanben_1550BP	sEA	Taiwan	ancient	1409	N.A.	24.33	121.77	N.A.	N.A.	Wang_C_et_al_2021	PASS
I3732	Hanben_1550BP	sEA	Taiwan	ancient	1235	N.A.	24.33	121.77	N.A.	N.A.	Wang_C_et_al_2021	PASS
I8072	Hanben_1550BP	sEA	Taiwan	ancient	1550	N.A.	24.33	121.77	N.A.	N.A.	Wang_C_et_al_2021	PASS
I3734	Hanben_1550BP	sEA	Taiwan	ancient	1575	N.A.	24.33	121.77	N.A.	N.A.	Wang_C_et_al_2021	PASS
I3735	Hanben_1550BP	sEA	Taiwan	ancient	1532	N.A.	24.33	121.77	N.A.	N.A.	Wang_C_et_al_2021	PASS
I3612	Hanben_1550BP	sEA	Taiwan	ancient	1800	N.A.	24.33	121.77	N.A.	N.A.	Wang_C_et_al_2021	PASS
I3611	Hanben_1550BP	sEA	Taiwan	ancient	1715	N.A.	24.33	121.77	N.A.	N.A.	Wang_C_et_al_2021	PASS
I12973	Mongolia_2500BP	nEA	Mongolia	ancient	3226	N.A.	49.41	102.69	N.A.	N.A.	Wang_C_et_al_2021	PASS
I13174	MongunTaiga_3350BP	nEA	Mongolia	ancient	3410	N.A.	47.07	91.83	N.A.	N.A.	Wang_C_et_al_2021	PASS
I12976	MongunTaiga_3350BP	nEA	Mongolia	ancient	3332	N.A.	49.36	88.71	N.A.	N.A.	Wang_C_et_al_2021	PASS
I6363	MongunTaiga_3050BP	nEA	Mongolia	ancient	3095	N.A.	45.35	90.85	N.A.	N.A.	Wang_C_et_al_2021	PASS
I7033	MongunTaiga_3050BP	nEA	Mongolia	ancient	3066	N.A.	49.96	92.05	N.A.	N.A.	Wang_C_et_al_2021	PASS
I12955	Munkhkhairkhan_3700BP	nEA	Mongolia	ancient	3824	N.A.	49.7	96.8	N.A.	N.A.	Wang_C_et_al_2021	PASS
I13173	Munkhkhairkhan_3700BP	nEA	Mongolia	ancient	3722	N.A.	47.07	91.83	N.A.	N.A.	Wang_C_et_al_2021	PASS
I6348	Munkhkhairkhan_3700BP	nEA	Mongolia	ancient	3632	N.A.	49.39	102.7	N.A.	N.A.	Wang_C_et_al_2021	PASS
I12958	Munkhkhairkhan_3500BP	nEA	Mongolia	ancient	3483	N.A.	47.07	91.83	N.A.	N.A.	Wang_C_et_al_2021	PASS
I13964	Ulaanzukh_3350BP	nEA	Mongolia	ancient	3350	N.A.	46.77	111.6	N.A.	N.A.	Wang_C_et_al_2021	PASS
I12972	Ulaanzukh_3350BP	nEA	Mongolia	ancient	3347	N.A.	46.77	111.6	N.A.	N.A.	Wang_C_et_al_2021	PASS
I14037	Ulaanzukh_3350BP	nEA	Mongolia	ancient	3306	N.A.	46.77	111.6	N.A.	N.A.	Wang_C_et_al_2021	PASS
I13180	Ulgii_4700BP	nEA	Mongolia	ancient	4862	N.A.	49.3	88.83	N.A.	N.A.	Wang_C_et_al_2021	PASS
I12977	Ulgii_4700BP	nEA	Mongolia	ancient	4822	N.A.	49.36	88.71	N.A.	N.A.	Wang_C_et_al_2021	PASS
I6361	Ulgii_4700BP	nEA	Mongolia	ancient	4483	N.A.	49.34	88.71	N.A.	N.A.	Wang_C_et_al_2021	PASS
I6357	SlabGrave_2850BP	nEA	Mongolia	ancient	2200	N.A.	47.7	106.4	N.A.	N.A.	Wang_C_et_al_2021	PASS
I6364	Mongolia_2500BP	nEA	Mongolia	ancient	2927	N.A.	45.39	90.8	N.A.	N.A.	Wang_C_et_al_2021	PASS
I14194	Mongolia_2500BP	nEA	Mongolia	ancient	2834	N.A.	46.77	111.6	N.A.	N.A.	Wang_C_et_al_2021	PASS
I13504	Mongolia_2500BP	nEA	Mongolia	ancient	2368	N.A.	50.7	99.2	N.A.	N.A.	Wang_C_et_al_2021	PASS
I13965	Mongolia_2500BP	nEA	Mongolia	ancient	2270	N.A.	48.68	88.38	N.A.	N.A.	Wang_C_et_al_2021	PASS
I6263	Pazyryk_2200BP	nEA	Mongolia	ancient	2221	N.A.	48.68	88.38	N.A.	N.A.	Wang_C_et_al_2021	PASS
I6224	Sagly_2250BP	nEA	Mongolia	ancient	2225	N.A.	49.96	92.05	N.A.	N.A.	Wang_C_et_al_2021	PASS
I6232	Sagly_2250BP	nEA	Mongolia	ancient	2239	N.A.	49.96	92.05	N.A.	N.A.	Wang_C_et_al_2021	PASS
I12970	Sagly_2250BP	nEA	Mongolia	ancient	2286	N.A.	49.96	92.05	N.A.	N.A.	Wang_C_et_al_2021	PASS
I7029	Sagly_2250BP	nEA	Mongolia	ancient	2225	N.A.	49.96	92.05	N.A.	N.A.	Wang_C_et_al_2021	PASS
I7027	Sagly_2250BP	nEA	Mongolia	ancient	2278	N.A.	49.96	92.05	N.A.	N.A.	Wang_C_et_al_2021	PASS

I6356	Sagly_2250BP	nEA	Mongolia	ancient	2250	N.A.	49.96	92.05	N.A.	N.A.	Wang_C_et_al_2021	PASS
I7030	Sagly_2250BP	nEA	Mongolia	ancient	2244	N.A.	49.96	92.05	N.A.	N.A.	Wang_C_et_al_2021	PASS
I7024	Sagly_2250BP	nEA	Mongolia	ancient	2244	N.A.	49.96	92.05	N.A.	N.A.	Wang_C_et_al_2021	PASS
I7022	Sagly_2250BP	nEA	Mongolia	ancient	2244	N.A.	49.96	92.05	N.A.	N.A.	Wang_C_et_al_2021	PASS
I7023	Sagly_2250BP	nEA	Mongolia	ancient	2226	N.A.	49.96	92.05	N.A.	N.A.	Wang_C_et_al_2021	PASS
I6233	Sagly_2250BP	nEA	Mongolia	ancient	2225	N.A.	49.96	92.05	N.A.	N.A.	Wang_C_et_al_2021	PASS
I6226	Sagly_2250BP	nEA	Mongolia	ancient	2220	N.A.	49.96	92.05	N.A.	N.A.	Wang_C_et_al_2021	PASS
I12960	SlabGrave_2850BP	nEA	Mongolia	ancient	3332	N.A.	46.92	102.76	N.A.	N.A.	Wang_C_et_al_2021	PASS
I12969	SlabGrave_2850BP	nEA	Mongolia	ancient	3001	N.A.	47.18	109.19	N.A.	N.A.	Wang_C_et_al_2021	PASS
I6352	SlabGrave_2850BP	nEA	Mongolia	ancient	2949	N.A.	46.9	102.77	N.A.	N.A.	Wang_C_et_al_2021	PASS
I7032	SlabGrave_2850BP	nEA	Mongolia	ancient	2849	N.A.	48.69	110.19	N.A.	N.A.	Wang_C_et_al_2021	PASS
I12971	SlabGrave_2850BP	nEA	Mongolia	ancient	2849	N.A.	49.15	114.87	N.A.	N.A.	Wang_C_et_al_2021	PASS
I13963	SlabGrave_2850BP	nEA	Mongolia	ancient	2829	N.A.	47.19	109.19	N.A.	N.A.	Wang_C_et_al_2021	PASS
I6349	SlabGrave_2850BP	nEA	Mongolia	ancient	2781	N.A.	45.3	113.85	N.A.	N.A.	Wang_C_et_al_2021	PASS
I6365	SlabGrave_2850BP	nEA	Mongolia	ancient	2744	N.A.	49.66	99.93	N.A.	N.A.	Wang_C_et_al_2021	PASS
I6359	SlabGrave_2850BP	nEA	Mongolia	ancient	2700	N.A.	50.12	100.05	N.A.	N.A.	Wang_C_et_al_2021	PASS
I6369	SlabGrave_2850BP	nEA	Mongolia	ancient	2554	N.A.	47.38	110.32	N.A.	N.A.	Wang_C_et_al_2021	PASS
I13175	Xianbei_1500BP	nEA	Mongolia	ancient	1483	N.A.	45.39	90.8	N.A.	N.A.	Wang_C_et_al_2021	PASS
I6228	Xiongnu_1900BP	nEA	Mongolia	ancient	1919	N.A.	49.96	92.05	N.A.	N.A.	Wang_C_et_al_2021	PASS
I1202	Yankovsky_2850BP	nEA	Russia	ancient	2850	N.A.	43.06	131.89	N.A.	N.A.	Wang_C_et_al_2021	PASS
I3358	Heishui_Mohe_750BP	nEA	Russia	ancient	769	N.A.	45.91	134.88	N.A.	N.A.	Wang_C_et_al_2021	PASS
I12974	Mongolia_650BP	nEA	Mongolia	ancient	682	N.A.	47.38	110.32	N.A.	N.A.	Wang_C_et_al_2021	PASS
I13961	Mongolia_650BP	nEA	Mongolia	ancient	609	N.A.	46.15	114.87	N.A.	N.A.	Wang_C_et_al_2021	PASS
I13176	Mongolia_1000BP	nEA	Mongolia	ancient	1011	N.A.	45.35	90.85	N.A.	N.A.	Wang_C_et_al_2021	PASS
Qihe3	Qihe_11550BP	sEA	China	ancient	11552	N.A.	25.4	117.6	N.A.	N.A.	Wang_T_et_al_2021	PASS
Longlin_1	Longlin_10550BP	sEA	China	ancient	10563	N.A.	24.64	105.17	N.A.	N.A.	Wang_T_et_al_2021	PASS
Dushan4_1	Dushan_8800BP	sEA	China	ancient	8784	N.A.	23.6	107.13	N.A.	N.A.	Wang_T_et_al_2021	PASS
Baojianshan5_M2	Baojianshan_7350BP	sEA	China	ancient	7368	N.A.	22.34	106.85	N.A.	N.A.	Wang_T_et_al_2021	PASS
Baojianshan5_M1	Baojianshan_7350BP	sEA	China	ancient	7368	N.A.	22.34	106.85	N.A.	N.A.	Wang_T_et_al_2021	PASS
BalongKD07	BaBanQinCen_1550BP	sEA	China	ancient	1552	N.A.	24.15	107.51	N.A.	N.A.	Wang_T_et_al_2021	PASS
BalongKD10	BaBanQinCen_1500BP	sEA	China	ancient	1489	N.A.	24.15	107.51	N.A.	N.A.	Wang_T_et_al_2021	PASS
QinchangKD14	BaBanQinCen_1500BP	sEA	China	ancient	1476	N.A.	24.1	107.51	N.A.	N.A.	Wang_T_et_al_2021	PASS
QinchangKD13	BaBanQinCen_1500BP	sEA	China	ancient	1442	N.A.	24.1	107.51	N.A.	N.A.	Wang_T_et_al_2021	PASS
BandaKD15	BaBanQinCen_1500BP	sEA	China	ancient	1435	N.A.	24.05	107.5	N.A.	N.A.	Wang_T_et_al_2021	PASS
CenxunKP05	BaBanQinCen_1500BP	sEA	China	ancient	1387	N.A.	23.4	107.4	N.A.	N.A.	Wang_T_et_al_2021	PASS
BandaKD11	BaBanQinCen_1500BP	sEA	China	ancient	1387	N.A.	24.05	107.5	N.A.	N.A.	Wang_T_et_al_2021	PASS
BalongKD06	BaBanQinCen_1500BP	sEA	China	ancient	1500	N.A.	24.15	107.51	N.A.	N.A.	Wang_T_et_al_2021	imiss
BalongKD08	BaBanQinCen_1500BP	sEA	China	ancient	1500	N.A.	24.15	107.51	N.A.	N.A.	Wang_T_et_al_2021	imiss
CenxunKP13	LaCen_1350BP	sEA	China	ancient	1411	N.A.	23.4	107.4	N.A.	N.A.	Wang_T_et_al_2021	PASS
LadaKH01	LaCen_1350BP	sEA	China	ancient	1388	N.A.	24	107.6	N.A.	N.A.	Wang_T_et_al_2021	PASS
CenxunKP07	LaCen_1350BP	sEA	China	ancient	1330	N.A.	23.4	107.4	N.A.	N.A.	Wang_T_et_al_2021	PASS
LayiKD01	Layi_1450BP	sEA	China	ancient	1468	N.A.	24.08	107.62	N.A.	N.A.	Wang_T_et_al_2021	PASS
ShenxianKP09	Shenxian_1300BP	sEA	China	ancient	1315	N.A.	23.31	107.59	N.A.	N.A.	Wang_T_et_al_2021	PASS
YiyangKP17	Yiyang_1350BP	sEA	China	ancient	1387	N.A.	23.33	107.5	N.A.	N.A.	Wang_T_et_al_2021	PASS

HuatuyanNL06	GaoHuaHua_450BP	sEA	China	ancient	500	N.A.	25.1	107.66	N.A.	N.A.	Wang_T_et_al_2021	PASS
HuatuyanNL18	GaoHuaHua_450BP	sEA	China	ancient	500	N.A.	25.1	107.66	N.A.	N.A.	Wang_T_et_al_2021	PASS
HuatuyanNL04	GaoHuaHua_450BP	sEA	China	ancient	500	N.A.	25.1	107.66	N.A.	N.A.	Wang_T_et_al_2021	imiss
Yinwang	GaoHuaHua_450BP	sEA	China	ancient	500	N.A.	23.82	107.52	N.A.	N.A.	Wang_T_et_al_2021	imiss
HuatuyanNL11	GaoHuaHua_450BP	sEA	China	ancient	446	N.A.	25.1	107.66	N.A.	N.A.	Wang_T_et_al_2021	PASS
HuatuyanNL17	GaoHuaHua_450BP	sEA	China	ancient	434	N.A.	25.1	107.66	N.A.	N.A.	Wang_T_et_al_2021	PASS
GaofengNL23	GaoHuaHua_450BP	sEA	China	ancient	421	N.A.	25.11	107.7	N.A.	N.A.	Wang_T_et_al_2021	imiss
HuaqiaoNL26	GaoHuaHua_450BP	sEA	China	ancient	419	N.A.	25.11	107.66	N.A.	N.A.	Wang_T_et_al_2021	PASS
HuatuyanNL21	GaoHuaHua_450BP	sEA	China	ancient	405	N.A.	25.1	107.66	N.A.	N.A.	Wang_T_et_al_2021	PASS
HuatuyanNL02	GaoHuaHua_450BP	sEA	China	ancient	386	N.A.	25.1	107.66	N.A.	N.A.	Wang_T_et_al_2021	PASS
HuatuyanNL19	GaoHuaHua_450BP	sEA	China	ancient	375	N.A.	25.1	107.66	N.A.	N.A.	Wang_T_et_al_2021	PASS
La368	PhaFaen_7000BP	MSEA	Laos	ancient	7040	N.A.	18.55	103.52	N.A.	N.A.	McColl_et_al_2018	PASS
Ma911	GuaChaCave_3900BP	MSEA	Malaysia	ancient	3872	N.A.	5.15	101.69	N.A.	N.A.	McColl_et_al_2018	PASS
Ma912	GuaChaCave_2400BP	MSEA	Malaysia	ancient	2409	N.A.	5.15	101.69	N.A.	N.A.	McColl_et_al_2018	PASS
I0627	ManBac_3800BP	MSEA	Vietnam	ancient	3963	N.A.	20.54	106.41	N.A.	N.A.	Lipson_et_al_2018	PASS
I1137	ManBac_3800BP	MSEA	Vietnam	ancient	3763	N.A.	20.54	106.41	N.A.	N.A.	Lipson_et_al_2018	PASS
I1859	ManBac_3800BP	MSEA	Vietnam	ancient	3765	N.A.	20.54	106.41	N.A.	N.A.	Lipson_et_al_2018	PASS
I2731	ManBac_3800BP	MSEA	Vietnam	ancient	3718	N.A.	20.54	106.41	N.A.	N.A.	Lipson_et_al_2018	PASS
I2947_new	ManBac_3800BP	MSEA	Vietnam	ancient	3750	N.A.	20.54	106.41	N.A.	N.A.	Lipson_et_al_2018	PASS
Vt778	NamTun_2550BP	MSEA	Vietnam	ancient	2549	N.A.	22.42	102.32	N.A.	N.A.	McColl_et_al_2018	PASS
Vt777	MaiDaDieu_2300BP	MSEA	Vietnam	ancient	2275	N.A.	20.13	105.32	N.A.	N.A.	McColl_et_al_2018	PASS
Vt833	MaiDaDieu_3800BP	MSEA	Vietnam	ancient	3788	N.A.	20.13	105.32	N.A.	N.A.	McColl_et_al_2018	PASS
Vt880	HonHaiCoTien_4000BP	MSEA	Vietnam	ancient	4000	N.A.	21.4	107.47	N.A.	N.A.	McColl_et_al_2018	PASS
La364	TamPaLing_2850BP	MSEA	Laos	ancient	2865	N.A.	20.77	104.01	N.A.	N.A.	McColl_et_al_2018	PASS
La727	TamHang_2200BP	MSEA	Laos	ancient	2320	N.A.	20.77	104.01	N.A.	N.A.	McColl_et_al_2018	PASS
La898	TamHang_2200BP	MSEA	Laos	ancient	2000	N.A.	20.77	104.01	N.A.	N.A.	McColl_et_al_2018	PASS
I4011_new	Oakaie_2950BP	MSEA	Myanmar	ancient	2950	N.A.	22.76	95.39	N.A.	N.A.	Lipson_et_al_2018	PASS
In661	LoyangUjungCave_2000BP	MSEA	Indonesia	ancient	1917	N.A.	3.85	96.47	N.A.	N.A.	McColl_et_al_2018	PASS
In662	LoyangUjungCave_2000BP	MSEA	Indonesia	ancient	2152	N.A.	3.85	96.47	N.A.	N.A.	McColl_et_al_2018	PASS
Vt779	NuiNap_2200BP	MSEA	Vietnam	ancient	2242	N.A.	19.98	105.69	N.A.	N.A.	McColl_et_al_2018	PASS
Vt781	NuiNap_2200BP	MSEA	Vietnam	ancient	2248	N.A.	19.98	105.69	N.A.	N.A.	McColl_et_al_2018	PASS
Vt796	NuiNap_2200BP	MSEA	Vietnam	ancient	2143	N.A.	19.98	105.69	N.A.	N.A.	McColl_et_al_2018	PASS
Vt808	NuiNap_2200BP	MSEA	Vietnam	ancient	2255	N.A.	19.98	105.69	N.A.	N.A.	McColl_et_al_2018	PASS
I2497	NuiNap_2200BP	MSEA	Vietnam	ancient	2000	N.A.	19.98	105.69	N.A.	N.A.	Lipson_et_al_2018	PASS
I2948_new	NuiNap_1950BP	MSEA	Vietnam	ancient	1948	N.A.	19.98	105.69	N.A.	N.A.	Lipson_et_al_2018	PASS
I1680_new	VatKomnou_1800BP	MSEA	Cambodia	ancient	1810	N.A.	10.98	104.97	N.A.	N.A.	Lipson_et_al_2018	PASS
Th519	LongLongRak_1750BP	MSEA	Thailand	ancient	1792	N.A.	19.55	98.27	N.A.	N.A.	McColl_et_al_2018	PASS
Th521	LongLongRak_1750BP	MSEA	Thailand	ancient	1785	N.A.	19.55	98.27	N.A.	N.A.	McColl_et_al_2018	PASS
Th530	LongLongRak_1750BP	MSEA	Thailand	ancient	1756	N.A.	19.55	98.27	N.A.	N.A.	McColl_et_al_2018	PASS
Th531	LongLongRak_1750BP	MSEA	Thailand	ancient	1687	N.A.	19.55	98.27	N.A.	N.A.	McColl_et_al_2018	PASS
Th703	LongLongRak_1750BP	MSEA	Thailand	ancient	1758	N.A.	19.55	98.27	N.A.	N.A.	McColl_et_al_2018	PASS
Vt719	HonHaiCoTien_200BP	MSEA	Vietnam	ancient	223	N.A.	21.4	107.47	N.A.	N.A.	McColl_et_al_2018	PASS
Ma554	SupuHujung_400BP	MSEA	Malaysia	ancient	383	N.A.	6.7	116.8	N.A.	N.A.	McColl_et_al_2018	PASS
Ma555	Kinabatagan_300BP	MSEA	Malaysia	ancient	299	N.A.	5.08	117.87	N.A.	N.A.	McColl_et_al_2018	PASS

LIA001002.TF1.1	Liang_Bua_2600BP	ISEA	Indonesia	ancient	2600	N.A.	-8.53	120.44	N.A.	N.A.	Oliveira_et_al_2021	PASS
TanjungPinang1.TF	Tanjung_Pinang_2100BP	ISEA	Indonesia	ancient	2100	N.A.	2	128.42	N.A.	N.A.	Oliveira_et_al_2021	PASS
TanjungPinang2.TF	Tanjung_Pinang_2100BP	ISEA	Indonesia	ancient	2100	N.A.	2	128.42	N.A.	N.A.	Oliveira_et_al_2021	PASS
TanjungPinang4.TF	Tanjung_Pinang_2100BP	ISEA	Indonesia	ancient	2100	N.A.	2	128.42	N.A.	N.A.	Oliveira_et_al_2021	PASS
TanjungPinang6.TF	Tanjung_Pinang_2100BP	ISEA	Indonesia	ancient	2100	N.A.	2	128.42	N.A.	N.A.	Oliveira_et_al_2021	PASS
Uattamdi1.TF	Uattamdi_1900BP	ISEA	Indonesia	ancient	1900	N.A.	0.05	127.41	N.A.	N.A.	Oliveira_et_al_2021	PASS
AMA001.B0101.TF1.1	Aru_Manara_2150BP	ISEA	Indonesia	ancient	2150	N.A.	2.3	128.8	N.A.	N.A.	Oliveira_et_al_2021	PASS
AMA0038.A0101.TF1.1	Aru_Manara_2150BP	ISEA	Indonesia	ancient	2150	N.A.	2.3	128.8	N.A.	N.A.	Oliveira_et_al_2021	PASS
AMA004.AB0101.TF1.1	Aru_Manara_2150BP	ISEA	Indonesia	ancient	2150	N.A.	2.3	128.8	N.A.	N.A.	Oliveira_et_al_2021	PASS
AMA005.A0101.TF1.1	Aru_Manara_2150BP	ISEA	Indonesia	ancient	2150	N.A.	2.3	128.8	N.A.	N.A.	Oliveira_et_al_2021	PASS
AMA009.AB0101.TF1.1	Aru_Manara_950BP	ISEA	Indonesia	ancient	950	N.A.	2.3	128.8	N.A.	N.A.	Oliveira_et_al_2021	PASS
TOP002.A0101.TF1.1	Topogaro_250BP	ISEA	Indonesia	ancient	250	N.A.	-2.2	121.66	N.A.	N.A.	Oliveira_et_al_2021	PASS
TOP004.A0101.TF1.1	Topogaro_250BP	ISEA	Indonesia	ancient	250	N.A.	-2.2	121.66	N.A.	N.A.	Oliveira_et_al_2021	PASS
JAB001.A0101.TF1.1	Jareng_Bori_450BP	ISEA	Indonesia	ancient	450	N.A.	-8.42	124.12	N.A.	N.A.	Oliveira_et_al_2021	PASS
KMO001.A0101.TF1.1	Komodo_750BP	ISEA	Indonesia	ancient	750	N.A.	-8.6	119.44	N.A.	N.A.	Oliveira_et_al_2021	PASS
LIT001.A0101.TF1.1	Liang_Toge_850BP	ISEA	Indonesia	ancient	850	N.A.	-8.65	120.99	N.A.	N.A.	Oliveira_et_al_2021	PASS
Lapita_I1368	Vanuatu_2900BP	Oceania	Vanuatu	ancient	2983	N.A.	-17.79	168.37	N.A.	N.A.	Skoglund_et_al_2016	PASS
Lapita_I1369	Vanuatu_2900BP	Oceania	Vanuatu	ancient	3045	N.A.	-17.79	168.37	N.A.	N.A.	Skoglund_et_al_2016	PASS
Lapita_I1370	Vanuatu_2900BP	Oceania	Vanuatu	ancient	3083	N.A.	-17.79	168.37	N.A.	N.A.	Skoglund_et_al_2016	PASS
Lapita_Sk10	Tonga_2600BP	Oceania	Tonga	ancient	2594	N.A.	-21.18	-175.12	N.A.	N.A.	Skoglund_et_al_2016	PASS
TON001	Tonga_2600BP	Oceania	Tonga	ancient	2625	N.A.	-21.18	175.11	N.A.	N.A.	Posth_et_al_2018	PASS
TON002	Tonga_2600BP	Oceania	Tonga	ancient	2625	N.A.	-21.18	175.11	N.A.	N.A.	Posth_et_al_2018	PASS
MAL006	Vanuatu_2400BP	Oceania	Vanuatu	ancient	2665	N.A.	-16.07	167.45	N.A.	N.A.	Posth_et_al_2018	imiss
MAL004	Vanuatu_2400BP	Oceania	Vanuatu	ancient	2567	N.A.	-16.07	167.45	N.A.	N.A.	Posth_et_al_2018	PASS
MAL002	Vanuatu_2400BP	Oceania	Vanuatu	ancient	2525	N.A.	-16.07	167.45	N.A.	N.A.	Posth_et_al_2018	PASS
TAN002	Vanuatu_2400BP	Oceania	Vanuatu	ancient	2490	N.A.	-19.33	169.34	N.A.	N.A.	Posth_et_al_2018	PASS
MAL001	Vanuatu_2400BP	Oceania	Vanuatu	ancient	2240	N.A.	-15.9	167.3	N.A.	N.A.	Posth_et_al_2018	PASS
MAL008	Vanuatu_2400BP	Oceania	Vanuatu	ancient	2238	N.A.	-16.07	167.45	N.A.	N.A.	Posth_et_al_2018	imiss
MAL007	Vanuatu_2400BP	Oceania	Vanuatu	ancient	2038	N.A.	-16.07	167.45	N.A.	N.A.	Posth_et_al_2018	PASS
FUT002	Vanuatu_1200BP	Oceania	Vanuatu	ancient	1238	N.A.	-19.52	170.23	N.A.	N.A.	Posth_et_al_2018	PASS
FUT006	Vanuatu_1200BP	Oceania	Vanuatu	ancient	1209	N.A.	-19.52	170.23	N.A.	N.A.	Posth_et_al_2018	PASS
FUT007	Vanuatu_1200BP	Oceania	Vanuatu	ancient	1204	N.A.	-19.52	170.23	N.A.	N.A.	Posth_et_al_2018	PASS
FUT001	Vanuatu_1200BP	Oceania	Vanuatu	ancient	1159	N.A.	-19.52	170.23	N.A.	N.A.	Posth_et_al_2018	PASS
LHA001	Tonga_800BP	Oceania	Tonga	ancient	844	N.A.	-21.18	-175.12	N.A.	N.A.	Posth_et_al_2018	PASS
MAI002	Solomon_Islands_500BP	Oceania	Solomon_Islands	ancient	467	N.A.	-9.25	161.22	N.A.	N.A.	Posth_et_al_2018	PASS
TAP003	French_Polynesia_400BP	Oceania	French_Polynesia	ancient	366	N.A.	-16.84	-151.36	N.A.	N.A.	Posth_et_al_2018	PASS
TAP004	French_Polynesia_200BP	Oceania	French_Polynesia	ancient	228	N.A.	-16.84	-151.36	N.A.	N.A.	Posth_et_al_2018	PASS
TAP002	French_Polynesia_200BP	Oceania	French_Polynesia	ancient	203	N.A.	-16.84	-151.36	N.A.	N.A.	Posth_et_al_2018	PASS
TAN001	Vanuatu_300BP	Oceania	Vanuatu	ancient	202	N.A.	-19.56	169.28	N.A.	N.A.	Posth_et_al_2018	PASS
I1370	Vanuatu_2900BP	Oceania	Vanuatu	ancient	2945	N.A.	-17.79	168.37	N.A.	N.A.	Lipson_et_al_2018_b	imiss
I1369	Vanuatu_2900BP	Oceania	Vanuatu	ancient	2885	N.A.	-17.79	168.37	N.A.	N.A.	Lipson_et_al_2018_b	imiss
I1368	Vanuatu_2900BP	Oceania	Vanuatu	ancient	2870	N.A.	-17.79	168.37	N.A.	N.A.	Lipson_et_al_2018_b	imiss
I5951	Vanuatu_2900BP	Oceania	Vanuatu	ancient	2820	N.A.	-17.79	168.37	N.A.	N.A.	Lipson_et_al_2018_b	PASS
I4451	Vanuatu_2400BP	Oceania	Vanuatu	ancient	2260	N.A.	-17.69	168.29	N.A.	N.A.	Lipson_et_al_2018_b	PASS

I3921	Vanuatu_1200BP	Oceania	Vanuatu	ancient	1260	N.A.	-16.71	168.13	N.A.	N.A.	Lipson_et_al_2018_b	PASS
I5259_published	Vanuatu_300BP	Oceania	Vanuatu	ancient	480	N.A.	-17.64	168.21	N.A.	N.A.	Lipson_et_al_2018_b	PASS
I4450	Vanuatu_300BP	Oceania	Vanuatu	ancient	215	N.A.	-17.68	168.53	N.A.	N.A.	Lipson_et_al_2018_b	PASS
I4105	Vanuatu_300BP	Oceania	Vanuatu	ancient	150	N.A.	-16.89	168.3	N.A.	N.A.	Lipson_et_al_2018_b	PASS
I4419	Vanuatu_300BP	Oceania	Vanuatu	ancient	140	N.A.	-17.8	168.52	N.A.	N.A.	Lipson_et_al_2018_b	PASS
I4424	Vanuatu_300BP	Oceania	Vanuatu	ancient	140	N.A.	-17.77	168.29	N.A.	N.A.	Lipson_et_al_2018_b	PASS
I4106	Vanuatu_300BP	Oceania	Vanuatu	ancient	140	N.A.	-16.89	168.3	N.A.	N.A.	Lipson_et_al_2018_b	PASS
I4425	Vanuatu_300BP	Oceania	Vanuatu	ancient	135	N.A.	-17.65	168.4	N.A.	N.A.	Lipson_et_al_2018_b	PASS
I5265	Vanuatu_2900BP	Oceania	Vanuatu	ancient	2875	N.A.	-17.79	168.39	N.A.	N.A.	Lipson_et_al_2020	imiss
I5266	Vanuatu_2900BP	Oceania	Vanuatu	ancient	2875	N.A.	-17.79	168.39	N.A.	N.A.	Lipson_et_al_2020	PASS
I5268	Vanuatu_2900BP	Oceania	Vanuatu	ancient	2995	N.A.	-17.79	168.39	N.A.	N.A.	Lipson_et_al_2020	imiss
I5267	Vanuatu_2900BP	Oceania	Vanuatu	ancient	3050	N.A.	-17.79	168.39	N.A.	N.A.	Lipson_et_al_2020	imiss
I6188	Vanuatu_2400BP	Oceania	Vanuatu	ancient	2400	N.A.	-17.7	168.27	N.A.	N.A.	Lipson_et_al_2020	imiss
I14493	Vanuatu_300BP	Oceania	Vanuatu	ancient	350	N.A.	-17.64	168.15	N.A.	N.A.	Lipson_et_al_2020	PASS
I10969	Vanuatu_300BP	Oceania	Vanuatu	ancient	350	N.A.	-17.64	168.15	N.A.	N.A.	Lipson_et_al_2020	PASS
I10968	Vanuatu_300BP	Oceania	Vanuatu	ancient	350	N.A.	-17.64	168.15	N.A.	N.A.	Lipson_et_al_2020	PASS
I10967	Vanuatu_300BP	Oceania	Vanuatu	ancient	180	N.A.	-17.64	168.2	N.A.	N.A.	Lipson_et_al_2020	PASS
I10966	Vanuatu_300BP	Oceania	Vanuatu	ancient	350	N.A.	-17.64	168.2	N.A.	N.A.	Lipson_et_al_2020	PASS
EFE005	Vanuatu_300BP	Oceania	Vanuatu	ancient	234	N.A.	-17.81	168.52	N.A.	N.A.	Lipson_et_al_2020	PASS
SP4210.all	Guam_2200BP	Oceania	US	ancient	2200	N.A.	13.62	144.87	N.A.	N.A.	Pugach_et_al_2021	PASS
SP4211.all	Guam_2200BP	Oceania	US	ancient	2200	N.A.	13.62	144.87	N.A.	N.A.	Pugach_et_al_2021	PASS
mota.SG	Mota_4470BP	Africa	Ethiopia	ancient	4470	N.A.	6.797495	38.207852	N.A.	N.A.	Llorente_et_al_2015	PASS (qpAdm)
Ust_Ishim_published.DG	Ust_Ishim_44350BP	nEA	Russia	ancient	44366	N.A.	57.7	71.1	N.A.	N.A.	Fu_et_al_2014	PASS (qpAdm)
Kostenki14.SG	Kostenki_38050BP	nEA	Russia	ancient	38052	N.A.	51.23	39.3	N.A.	N.A.	Seguin-Orlando_et_al_2014	PASS (qpAdm)
I1290	Iran_10000BP	WestAsia	Iran	ancient	9806	N.A.	34.45	48.116	N.A.	N.A.	Lazaridis_et_al_2016	PASS (qpAdm)
I1944	Iran_10000BP	WestAsia	Iran	ancient	9800	N.A.	34.45	48.116	N.A.	N.A.	Narasimhan_et_al_2019	PASS (qpAdm)
I1945	Iran_10000BP	WestAsia	Iran	ancient	9800	N.A.	34.45	48.116	N.A.	N.A.	Narasimhan_et_al_2019	PASS (qpAdm)
I1947	Iran_10000BP	WestAsia	Iran	ancient	9992	N.A.	34.45	48.116	N.A.	N.A.	Narasimhan_et_al_2019	PASS (qpAdm)
I1949	Iran_10000BP	WestAsia	Iran	ancient	10042	N.A.	34.45	48.116	N.A.	N.A.	Narasimhan_et_al_2019	PASS (qpAdm)
I1954	Iran_10000BP	WestAsia	Iran	ancient	10162	N.A.	34.45	48.116	N.A.	N.A.	Narasimhan_et_al_2019	PASS (qpAdm)
I1951	Iran_10000BP	WestAsia	Iran	ancient	9848	N.A.	34.45	48.116	N.A.	N.A.	Narasimhan_et_al_2019	PASS (qpAdm)
I7527	Iran_10000BP	WestAsia	Iran	ancient	9900	N.A.	34.45	48.116	N.A.	N.A.	Narasimhan_et_al_2019	PASS (qpAdm)
Yana_old.SG	Yana_31850BP	nEA	Russia	ancient	31850	N.A.	70.72	135.42	N.A.	N.A.	Sikora_et_al_2019	PASS (qpAdm)
I0061.SG	Karelia_8450BP	nEA	Russia	ancient	8450	N.A.	61.65	35.65	N.A.	N.A.	Fu_et_al_2016	PASS (qpAdm)
I2123	Indus_Periphery_4500BP	WestAsia	Turkmenistan	ancient	4211	N.A.	38.21228	62.03443	N.A.	N.A.	Narasimhan_et_al_2019	PASS (qpAdm)
I11456_published	Indus_Periphery_4500BP	WestAsia	Iran	ancient	4500	N.A.	30.649857	61.400311	N.A.	N.A.	Narasimhan_et_al_2019	PASS (qpAdm)
I11459_published	Indus_Periphery_4500BP	WestAsia	Iran	ancient	4699	N.A.	30.649857	61.400311	N.A.	N.A.	Narasimhan_et_al_2019	PASS (qpAdm)
I11466_published	Indus_Periphery_4500BP	WestAsia	Iran	ancient	4200	N.A.	30.649857	61.400311	N.A.	N.A.	Narasimhan_et_al_2019	PASS (qpAdm)
I8726	Indus_Periphery_4500BP	WestAsia	Iran	ancient	5000	N.A.	30.649857	61.400311	N.A.	N.A.	Narasimhan_et_al_2019	PASS (qpAdm)
I8728_published	Indus_Periphery_4500BP	WestAsia	Iran	ancient	4500	N.A.	30.649857	61.400311	N.A.	N.A.	Narasimhan_et_al_2019	PASS (qpAdm)
I11458_published	Indus_Periphery_4500BP	WestAsia	Iran	ancient	4600	N.A.	30.649857	61.400311	N.A.	N.A.	Narasimhan_et_al_2019	imiss
USR1.SG	Upward_Sun_River_11400BP	America	US	ancient	11425	N.A.	64.22	-145.7	N.A.	N.A.	Moreno-Mayar_et_al_2017	PASS (qpAdm)

Kolyma_River.SG	Kolyma_9750BP	nEA	Russia	ancient	9775	N.A.	68.6	159.1	N.A.	N.A.	Sikora_et_al_2019	PASS (qpAdm)
IK002.SG	Jomon_2800BP	nEA	Japan	ancient	2782	N.A.	35.0183	137.294	N.A.	N.A.	McColl_et_al_2018	PASS (qpAdm)
RISE515.SG	Okunevo_4300BP	nEA	Russia	ancient	4196	N.A.	53.156486	90.207811	N.A.	N.A.	Damgaard_et_al_2018	PASS (qpAdm)
RISE667.SG	Okunevo_4300BP	nEA	Russia	ancient	4300	N.A.	53.156486	90.207811	N.A.	N.A.	Damgaard_et_al_2018	PASS (qpAdm)
RISE670.SG	Okunevo_4300BP	nEA	Russia	ancient	3959	N.A.	53.156486	90.207811	N.A.	N.A.	Damgaard_et_al_2018	PASS (qpAdm)
RISE671.SG	Okunevo_4300BP	nEA	Russia	ancient	4300	N.A.	53.156486	90.207811	N.A.	N.A.	Damgaard_et_al_2018	PASS (qpAdm)
RISE672.SG	Okunevo_4300BP	nEA	Russia	ancient	4300	N.A.	53.156486	90.207811	N.A.	N.A.	Damgaard_et_al_2018	PASS (qpAdm)
RISE674.SG	Okunevo_4300BP	nEA	Russia	ancient	4064	N.A.	53.156486	90.207811	N.A.	N.A.	Damgaard_et_al_2018	PASS (qpAdm)
RISE675.SG	Okunevo_4300BP	nEA	Russia	ancient	4517	N.A.	53.708561	90.359808	N.A.	N.A.	Damgaard_et_al_2018	PASS (qpAdm)
RISE677.SG	Okunevo_4300BP	nEA	Russia	ancient	4409	N.A.	53.708561	90.359808	N.A.	N.A.	Damgaard_et_al_2018	PASS (qpAdm)
RISE680.SG	Okunevo_4300BP	nEA	Russia	ancient	4300	N.A.	53.708561	90.359808	N.A.	N.A.	Damgaard_et_al_2018	PASS (qpAdm)
RISE681.SG	Okunevo_4300BP	nEA	Russia	ancient	4300	N.A.	53.708561	90.359808	N.A.	N.A.	Damgaard_et_al_2018	PASS (qpAdm)
RISE683.SG	Okunevo_4300BP	nEA	Russia	ancient	3973	N.A.	53.708561	90.359808	N.A.	N.A.	Damgaard_et_al_2018	PASS (qpAdm)
RISE684.SG	Okunevo_4300BP	nEA	Russia	ancient	4239	N.A.	53.708561	90.359808	N.A.	N.A.	Damgaard_et_al_2018	PASS (qpAdm)
RISE685.SG	Okunevo_4300BP	nEA	Russia	ancient	4300	N.A.	53.708561	90.359808	N.A.	N.A.	Damgaard_et_al_2018	PASS (qpAdm)
RISE719.SG	Okunevo_4300BP	nEA	Russia	ancient	4300	N.A.	54.371767	91.506856	N.A.	N.A.	Damgaard_et_al_2018	PASS (qpAdm)
